# Mapping development and health effects of cooking with solid fuels in low-income and middle-income countries, 2000–18: a geospatial modelling study

**DOI:** 10.1016/S2214-109X(22)00332-1

**Published:** 2022-09-13

**Authors:** Local Burden of Disease Household Air Pollution Collaborators, Joseph Jon Frostad, QuynhAnh P Nguyen, Mathew M Baumann, Brigette F Blacker, Laurie B Marczak, Aniruddha Deshpande, Kirsten E Wiens, Kate E LeGrand, Kimberly B Johnson, Mohsen Abbasi-Kangevari, Amir Abdoli, Hassan Abolhassani, Lucas Guimarães Abreu, Michael R M Abrigo, Niveen ME Abu-Rmeileh, Victor Adekanmbi, Anurag Agrawal, Muktar Beshir Ahmed, Ziyad Al-Aly, Fahad Mashhour Alanezi, Jacqueline Elizabeth Alcalde-Rabanal, Vahid Alipour, Khalid A Altirkawi, Nelson Alvis-Guzman, Nelson J Alvis-Zakzuk, Adeladza Kofi Amegah, Saeed Amini, Fatemeh Amiri, Dickson A Amugsi, Robert Ancuceanu, Catalina Liliana Andrei, Tudorel Andrei, Ernoiz Antriyandarti, Davood Anvari, Jalal Arabloo, Morteza Arab-Zozani, Seyyed Shamsadin Athari, Marcel Ausloos, Getinet Ayano, Yared Asmare Aynalem, Samad Azari, Ashish D Badiye, Atif Amin Baig, Kalpana Balakrishnan, Maciej Banach, Sanjay Basu, Neeraj Bedi, Michelle L Bell, Derrick A Bennett, Krittika Bhattacharyya, Zulfiqar A Bhutta, Sadia Bibi, Somayeh Bohlouli, Soufiane Boufous, Nicola Luigi Bragazzi, Dejana Braithwaite, Sharath Burugina Nagaraja, Zahid A Butt, Florentino Luciano Caetano dos Santos, Josip Car, Rosario Cárdenas, Felix Carvalho, Joao Mauricio Castaldelli-Maia, Carlos A Castañeda-Orjuela, Ester Cerin, Soosanna Kumary Chattu, Vijay Kumar Chattu, Pankaj Chaturvedi, Sarika Chaturvedi, Simiao Chen, Dinh-Toi Chu, Sheng-Chia Chung, Saad M A Dahlawi, Giovanni Damiani, Lalit Dandona, Rakhi Dandona, Aso Mohammad Darwesh, Jai K Das, Aditya Prasad Dash, Claudio Alberto Dávila-Cervantes, Diego De Leo, Jan-Walter De Neve, Getu Debalkie Demissie, Edgar Denova-Gutiérrez, Sagnik Dey, Samath Dhamminda Dharmaratne, Meghnath Dhimal, Govinda Prasad Dhungana, Daniel Diaz, Isaac Oluwafemi Dipeolu, Fariba Dorostkar, Leila Doshmangir, Andre Rodrigues Duraes, Hisham Atan Edinur, Ferry Efendi, Maha El Tantawi, Sharareh Eskandarieh, Ibtihal Fadhil, Nazir Fattahi, Nelsensius Klau Fauk, Seyed-Mohammad Fereshtehnejad, Morenike Oluwatoyin Folayan, Masoud Foroutan, Takeshi Fukumoto, Abhay Motiramji Gaidhane, Mansour Ghafourifard, Ahmad Ghashghaee, Syed Amir Gilani, Tiffany K Gill, Alessandra C Goulart, Bárbara Niegia Garcia Goulart, Ayman Grada, Mohammed Ibrahim Mohialdeen Gubari, Davide Guido, Yuming Guo, Rajat Das Gupta, Rajeev Gupta, Reyna Alma Gutiérrez, Nima Hafezi-Nejad, Randah R Hamadeh, Ahmed I Hasaballah, Soheil Hassanipour, Khezar Hayat, Behzad Heibati, Reza Heidari-Soureshjani, Nathaniel J Henry, Claudiu Herteliu, Mehdi Hosseinzadeh, Mohamed Hsairi, Guoqing Hu, Segun Emmanuel Ibitoye, Olayinka Stephen Ilesanmi, Irena M Ilic, Milena D Ilic, Seyed Sina Naghibi Irvani, Sheikh Mohammed Shariful Islam, Chidozie C D Iwu, Jalil Jaafari, Mihajlo Jakovljevic, Tahereh Javaheri, Ravi Prakash Jha, John S Ji, Jost B Jonas, Ali Kabir, Zubair Kabir, Rohollah Kalhor, Naser Kamyari, Tanuj Kanchan, Umesh Kapil, Neeti Kapoor, Gbenga A Kayode, Peter Njenga Keiyoro, Yousef Saleh Khader, Nauman Khalid, Ejaz Ahmad Khan, Maseer Khan, Md Nuruzzaman Khan, Khaled Khatab, Mona M Khater, Mahalaqua Nazli Khatib, Maryam Khayamzadeh, Jagdish Khubchandani, Gyu Ri Kim, Yun Jin Kim, Ruth W Kimokoti, Adnan Kisa, Sezer Kisa, Luke D Knibbs, Parvaiz A Koul, Ai Koyanagi, Kewal Krishan, G Anil Kumar, Manasi Kumar, Dian Kusuma, Carlo La Vecchia, Ben Lacey, Faris Hasan Lami, Qing Lan, Savita Lasrado, Paolo Lauriola, Paul H Lee, Sonia Lewycka, Shanshan Li, Daiane Borges Machado, Phetole Walter Mahasha, Mina Maheri, Azeem Majeed, Afshin Maleki, Reza Malekzadeh, Deborah Carvalho Malta, Borhan Mansouri, Mohammad Ali Mansournia, Natalie Maria Martinez, Santi Martini, Francisco Rogerlândio Martins-Melo, Benjamin K Mayala, Man Mohan Mehndiratta, Walter Mendoza, Ritesh G Menezes, Endalkachew Worku Mengesha, Tuomo J Meretoja, Tomislav Mestrovic, Irmina Maria Michalek, Erkin M Mirrakhimov, Maryam Mirzaei, Roya Mirzaei, Babak Moazen, Yousef Mohammad, Abdollah Mohammadian-Hafshejani, Shafiu Mohammed, Ali H Mokdad, Lorenzo Monasta, Maziar Moradi-Lakeh, Paula Moraga, Lidia Morawska, Abbas Mosapour, Simin Mouodi, Amin Mousavi Khaneghah, Satinath Mukhopadhyay, Sandra B Munro, Christopher J L Murray, Ahamarshan Jayaraman Nagarajan, Mohsen Naghavi, Sanjeev Nair, Vinay Nangia, Bruno Ramos Nascimento, Javad Nazari, Ionut Negoi, Henok Biresaw Netsere, Josephine W Ngunjiri, Huong Lan Thi Nguyen, Jean Jacques Noubiap, Bogdan Oancea, Felix Akpojene Ogbo, In-Hwan Oh, Andrew T Olagunju, Bolajoko Olubukunola Olusanya, Jacob Olusegun Olusanya, Ahmed Omar Bali, Obinna E Onwujekwe, Nikita Otstavnov, Stanislav S Otstavnov, Mayowa O Owolabi, Mahesh P A, Anamika Pandey, Eun-Cheol Park, Eun-Kee Park, Sangram Kishor Patel, Hai Quang Pham, Thomas Pilgrim, Meghdad Pirsaheb, Khem Narayan Pokhrel, Maarten J Postma, Zahiruddin Quazi Syed, Navid Rabiee, Amir Radfar, Fakher Rahim, Mohammad Hifz Ur Rahman, Muhammad Aziz Rahman, Amir Masoud Rahmani, Chhabi Lal Ranabhat, Sowmya J Rao, Davide Rasella, Prateek Rastogi, Goura Kishor Rath, David Laith Rawaf, Salman Rawaf, Lal Rawal, Reza Rawassizadeh, Andre M N Renzaho, Bhageerathy Reshmi, Negar Rezaei, Nima Rezaei, Aziz Rezapour, Jennifer Rickard, Leonardo Roever, Luca Ronfani, Morteza Rostamian, Enrico Rubagotti, Godfrey M Rwegerera, Basema Saddik, Ehsan Sadeghi, Sahar Saeedi Moghaddam, Rajesh Sagar, Amirhossein Sahebkar, Biniyam Sahiledengle, Marwa Rashad Salem, Abdallah M Samy, Milena M Santric-Milicevic, Sivan Yegnanarayana Iyer Saraswathy, Brijesh Sathian, Thirunavukkarasu Sathish, David C Schwebel, Sadaf G Sepanlou, Saeed Shahabi, Amira A Shaheen, Izza Shahid, Masood Ali Shaikh, Ali S Shalash, Mehran Shams-Beyranvand, Mohammed Shannawaz, Kiomars Sharafi, Aziz Sheikh, Sara Sheikhbahaei, Ranjitha S Shetty, Wondimeneh Shibabaw Shiferaw, Mika Shigematsu, Jae Il Shin, K M Shivakumar, Soraya Siabani, Tariq Jamal Siddiqi, Balbir Bagicha Singh, Jasvinder A Singh, Yitagesu Sintayehu, Muluken Bekele Sorrie, Ireneous N Soyiri, Emma Elizabeth Spurlock, Chandrashekhar T Sreeramareddy, Leo Stockfelt, Mu'awiyyah Babale Sufiyan, Rizwan Suliankatchi Abdulkader, Rafael Tabarés-Seisdedos, Takahiro Tabuchi, Amir Taherkhani, Mohamad-Hani Temsah, Kavumpurathu Raman Thankappan, Marcos Roberto Tovani-Palone, Eugenio Traini, Saif Ullah, Bhaskaran Unnikrishnan, Era Upadhyay, Sahel Valadan Tahbaz, Santosh Varughese, Francesco S Violante, Bay Vo, Giang Thu Vu, Yasir Waheed, Yuan-Pang Wang, Catherine A Welgan, Andrea Werdecker, Seyed Hossein Yahyazadeh Jabbari, Sanni Yaya, Vahid Yazdi-Feyzabadi, Mekdes Tigistu Yilma, Naohiro Yonemoto, Mustafa Z Younis, Taraneh Yousefinezhadi, Chuanhua Yu, Yong Yu, Sojib Bin Zaman, Yunquan Zhang, Zhi-Jiang Zhang, Michael Brauer, Simon I Hay, Robert C Reiner

## Abstract

**Background:**

More than 3 billion people do not have access to clean energy and primarily use solid fuels to cook. Use of solid fuels generates household air pollution, which was associated with more than 2 million deaths in 2019. Although local patterns in cooking vary systematically, subnational trends in use of solid fuels have yet to be comprehensively analysed. We estimated the prevalence of solid-fuel use with high spatial resolution to explore subnational inequalities, assess local progress, and assess the effects on health in low-income and middle-income countries (LMICs) without universal access to clean fuels.

**Methods:**

We did a geospatial modelling study to map the prevalence of solid-fuel use for cooking at a 5 km × 5 km resolution in 98 LMICs based on 2·1 million household observations of the primary cooking fuel used from 663 population-based household surveys over the years 2000 to 2018. We use observed temporal patterns to forecast household air pollution in 2030 and to assess the probability of attaining the Sustainable Development Goal (SDG) target indicator for clean cooking. We aligned our estimates of household air pollution to geospatial estimates of ambient air pollution to establish the risk transition occurring in LMICs. Finally, we quantified the effect of residual primary solid-fuel use for cooking on child health by doing a counterfactual risk assessment to estimate the proportion of deaths from lower respiratory tract infections in children younger than 5 years that could be associated with household air pollution.

**Findings:**

Although primary reliance on solid-fuel use for cooking has declined globally, it remains widespread. 593 million people live in districts where the prevalence of solid-fuel use for cooking exceeds 95%. 66% of people in LMICs live in districts that are not on track to meet the SDG target for universal access to clean energy by 2030. Household air pollution continues to be a major contributor to particulate exposure in LMICs, and rising ambient air pollution is undermining potential gains from reductions in the prevalence of solid-fuel use for cooking in many countries. We estimated that, in 2018, 205 000 (95% uncertainty interval 147 000–257 000) children younger than 5 years died from lower respiratory tract infections that could be attributed to household air pollution.

**Interpretation:**

Efforts to accelerate the adoption of clean cooking fuels need to be substantially increased and recalibrated to account for subnational inequalities, because there are substantial opportunities to improve air quality and avert child mortality associated with household air pollution.

**Funding:**

Bill & Melinda Gates Foundation.

## Introduction

The deleterious health effects of household air pollution are long established: solid-fuel use, defined by WHO as primary reliance on wood, crop residue, coal, or dung for cooking, heating, and lighting,^1^ was first associated with increased risk of respiratory infections in children in Papua New Guinea almost 50 years ago.^2^ The fine particulate matter smaller than 2·5 μm (PM_2·5_) generated by solid-fuel use is a complex mixture that causes harm to health through multiple pathways, including mucociliary dysfunction (which increases susceptibility to infection) and hyperinflammation or immunodeficiency (which can worsen disease prognosis).^3^ Solid-fuel use results in PM_2·5_ exposure both within the home and more broadly through emissions that contribute substantially to ambient air pollution.^4^, ^5^

High-income countries have almost fully transitioned to clean fuels (ie, the prevalence of solid-fuel use is less than 5%).^6^, ^7^ Across low-income and middle-income countries (LMICs), the net effects of household air pollution—including health effects (US$1·4 trillion), lost productivity ($0·8 trillion), and environmental degradation ($0·4 trillion)—represent an immense annual cost, and thus access to clean and sustainable energy needs to be an essential part of the development agenda.^8^ Clean cooking is core to proposed indicators for monitoring Sustainable Development Goal (SDG) 7 (target 7.1: universal access to clean fuels and technology), and has important synergies with goals related to health (SDG 3), education (SDG 4), gender (SDG 5), urban development (SDG 11), climate change (SDG 13), and terrestrial ecology (SDG 15).^9^ Prevention strategies targeting household air pollution are shifting towards supplying households with technology or fuels for clean cooking, such as liquefied petroleum gas or electricity.^10^ Clean-fuel campaigns are often targeted subnationally, and even country-level programmes have shown heterogeneous patterns of adoption.^11^, ^12^ Previously, descriptive analyses of household air pollution and solid-fuel use have focused on a subset of relevant countries^13^, ^14^ or have been done globally but constrained by their spatial scale,^6^, ^15^ and were of little use for highlighting local patterns or identifying subnational inequality.

**Research in context**

**Evidence before this study**

We did not do a formal systematic search of the literature. Previous analyses have quantified the cause-specific disease burden associated with household air pollution globally, including studies using integrated exposure–response curves and pooled meta-analysis from a systematic review. These efforts showed the substantial health effects associated with cooking with solid fuels but did not examine trends in the underlying prevalence of the use of solid fuel in depth. A study estimated the prevalence of primary reliance on specific fuel types at the global and national level over the past 30 years, including forecasts to 2030. Evidence that community-level drivers are the strongest predictors of clean fuel adoption implies that the operational scale of solid-fuel use is more granular and that failure to account for local patterns could obscure inequalities.

**Added value of this study**

We used geostatistical methods to estimate the prevalence of primary use of solid fuels for cooking and household air pollution at substantially higher resolutions than previous studies, which allowed us to do subnational trend analysis in 98 low-income and middle-income countries from 2000 to 2018. By aggregating these geospatial estimates to second-level administrative boundaries (districts), we were able to provide actionable insights aligned to the scale of precision public health. We also made projections of progress to 2030, which suggested that few countries are on track to reach the Sustainable Development Goal of universal access to clean fuels within the coming decade. Finally, we combined our high-resolution estimates of the prevalence of solid-fuel use for cooking with exposure–response curves and equivalently resolved estimates of under-5 mortality from lower respiratory tract infections to quantify effects on child health.

**Implications of all the available evidence**

Although some regions exhibited substantial progress from 2000, in many regions in low-income and middle-income countries, primary reliance on solid fuels for cooking was still ubiquitous in 2018. We noted substantial subnational disparities, leading to health inequalities. Local estimates highlight the outstanding challenge of attaining universal access to clean cooking fuels, and risk assessments showed that hundreds of thousands of children still die annually from lower respiratory tract infections associated with household air pollution. The economic downturn and increased public health strain associated with the ongoing COVID-19 pandemic suggest that our forecasts are likely optimistic, and that the transition to clean and modern fuels must be broadly accelerated to fulfil the bold ambitions of the Sustainable Development Goals.

In this study, we generate the first high-resolution geospatial estimates of the prevalence of solid-fuel use (as indicated by primary fuel type) and the resulting household concentrations of PM_2·5_ in 98 LMICS. Our aim was to assess growth in access to clean cooking fuels over the past two decades. We also use temporal trends from 2000 to 2018 to forecast the likelihood of achieving SDG target 7.1 by 2030. We further quantify the household-level relationship between household and ambient air pollution by juxtaposing our estimates with data for ambient exposure to PM_2·5_ with equivalent spatial resolution to establish a robust indicator of total personal exposure to PM_2·5_ air pollution. Finally, we combine our results with population data, PM_2·5_ exposure–response functions, and equivalently resolved geospatial estimates of mortality from lower respiratory tract infections (LRTIs) in children younger than 5 years—a case study designed to assess the health effects of residual solid-fuel use in this vulnerable population.

## Methods

### Data sources

Solid-fuel use was estimated on the basis of population-based household survey data, in which respondents indicated the primary cooking fuel being used in the household. These responses were mapped to one of eight categories: no cooking in household, electricity, gas, kerosene, coal, wood, crop waste, and dung. Coal, wood, crop waste, and dung were considered solid fuels, whereas the others were deemed clean fuels. We then constructed a binary indicator of solid-fuel use (primary reliance on solid fuels *vs* primary reliance on clean fuels).

98 LMICs were included in the analysis based on their Socio-demographic Index scores (a development index derived from education, fertility, and poverty estimates), which were calculated using values from the low, low-middle, and middle quintiles from the Global Burden of Disease 2019.^16^ Sources of input data were only included for modelling if they were representative of the entire population during the time period and across the geographical area of measurement. Furthermore, certain sources were excluded if the associated estimates seemed implausible based on expert review of estimates and comparison with other sources in the same country and time period. We excluded LMICs with populations of less than 1 million and those that did not have household survey data available (appendix p 2). In total, 663 household surveys were compiled and extracted (appendix p 84). The full database represented 2·1 million people from 2000 to 2018 and included geocoded information from 181 556 coordinates (points) and 417 650 subnational administrative boundaries (polygons). Further details about the data-extraction and data-processing sequence are in the appendix (p 4).

### Definitions

In this study, we defined solid-fuel use as the household-level prevalence of primary reliance on solid fuels for cooking, which was described by the adminstrators of the included surveys as the fuel used most often for cooking in a household. In accordance with the Global Burden of Disease (GBD) 2019 study,^16^ household air pollution was defined as the incremental concentration of PM_2·5_ generated from cooking with solid fuels. We used the estimated ambient concentration of PM_2·5_ in a location as the baseline exposure (appendix p 6). We subtracted this value from the total personal PM_2·5_ exposure estimated for a solid fuel user: the difference represented the additional contribution of household air pollution to PM_2·5_ exposure.

### Statistical analysis

Available geospatial covariates with plausible a priori relationships with solid-fuel use were compiled for use in the prediction model (appendix p 4). We included seven indicators of urbanicity or development, which could be associated with increased access to clean-fuel technologies,^17^ such as travel and night-time lights. We also included 16 environmental indicators that might be associated with access to fuelwood or other solid fuels,^18^ including diurnal temperature range, elevation, and the normalised difference vegetation index (an indicator of whether a given observation contains live green material, which is calculated by comparing satellite images generated from visible and near-infrared light to estimate plant mass in the pixel). To account for potential multicollinearity, we used the variance inflation factor to analyse these covariates and filtered for each modelling region using a threshold of 5 (which was chosen to prioritise predictive over explanatory power).

We used a Bayesian hierarchical modelling framework to model household exposure to solid-fuel use through a generalised linear mixed-effects model that was spatially explicit. Prevalence of exposure to solid-fuel sources was modelled using the observed number of household members exposed as binomial count data (*C*_d_) among a sample size (*N*_d_). Annual prevalence in each primary sampling unit (cluster; *d*) for each survey was the modelled quantity, which was mapped to a geospatial raster location (*i*) for every year (*t*):

$$C_{d}|p_{i(d)}N_{d}\sim Binomial(p_{i(d)}N_{d})\forall obs.clustersd$$

$$\text{logit}(p_{i,t})=\beta_{0}+X_{i,\beta }+{ɛ}_{c(i)}+{ɛ}_{n(i)}+{ɛ}_{i}-Z_{i}$$

$$\sum_{n=1}^{3}\beta_{h}=1$$

$${ɛ}_{c}\overset{iid}{\sim }N(0,\gamma_{c}^{2})$$

$${ɛ}_{n}\overset{iid}{\sim }N(0,\gamma_{n}^{2})$$

$${ɛ}_{i}\overset{iid}{\sim }N(0,\sigma^{2})$$

$$Z\sim GP(0,\sum^{space})$$

$$\sum^{space}=\frac{\omega^{2}}{\Gamma (v)2^{v-1}}*{\left(kD\right)}^{v}*K_{v}(kD)$$

The appendix (p 5) contains a detailed explanation of these calculations, including definitions of all other included variables.

Prevalence of solid-fuel use was modelled as a linear combination of three submodels (generalised additive models, gradient boosted decision trees, and lasso regression; appendix p 4), rasterised spatiotemporal covariate values, a correlated spatial random effect term (*Z*_i_), country random effects (ɛ_c_), survey-specific random effects (ɛ_n_), and an independent nugget random effect (ɛ_i_). The coefficient of each submodel (β), represented the predictive weighting within the logit link. A key strength of this approach is the ability to leverage residual correlation structures within the predictions to make more accurate estimates for data-sparse locations, while simultaneously propagating this dependence through to estimates of uncertainty in all indicators (appendix p 6). The posterior distributions were fit based on approximations in integrated nested Laplace approximation (R-INLA), with approximation of the stochastic partial differential equations to the Gaussian process residuals done in R (version 3.6.1).

Models were assessed on the basis of a five-fold out-of-sample cross-validation strategy that was stratified over space (appendix p 6). Estimates of bias (mean error), variance (root-mean-square error), coverage of data by 95% prediction intervals (appendix p 6), and correlations between predictions and observed data were used to assess the models (appendix p 6). In-sample and out-of-sample model validation plots were also produced comparing every country and first and second administrative unit estimated with the observed data for those units (appendix p 6).

Pixel-level estimates of solid-fuel use were calibrated to estimates from the GBD 2019 study using a previously described method^19^ to preserve relative spatial patterns while ensuring comparability and incorporating information from national-level reports that could not be used within geospatial models. We defined calibration factors on the basis of comparison of draws from GBD outputs to population-weighted aggregations of our estimates at the highest level of spatial granularity available (either national or the first administrative level for select countries for which GBD-produced subnational estimates were available).

We combined the model output (*P(SFU)*) with geospatial estimates of population (*pop*) and ambient PM_2·5_ exposure (*APM*_2·5_) from the GBD 2019 study^16^ to calculate personal total exposure to PM_2·5_ pollution (*TAP*) (the sum of household and ambient air pollution in each 5 km × 5 km grid cell *i* [pixel]):

$$HAP_{i,c,t}=(P{\left(SFU\right)}_{i,c,t}*pop_{i,c,t})*HPM_{2.5_{c,t}}$$

$$TAP_{i,c,t}=pop_{i,c,t}*APM_{2.5_{c,t}}+HPM_{i,c,t}$$

$$TAPRR_{i,c,t,o}=IER{\left(\frac{TAP_{i,c,t}}{pop_{i,c,t}}\right)}_{o}$$

$$TAPPAF_{i,c,t,o}=\frac{TAPRR_{i,c,t,o}}{TAPRR_{i,c,t,o}-1}$$

$$HAPN_{i,c,t,o}=TAPPAF_{i,c,t,o}*N_{i,c,t,o}*HAP_{i,c,t}/TAP_{i,c,t}$$

Estimates of the expected incremental PM_2·5_ concentration generated in a household using solid fuels (*HPM*_2·5_) for a given country (*c*) and year (*t*) from GBD 2019 were used to calculate the concentration of household air pollution in the exposed population.^20^ The per-person annual average ambient PM_2·5_ estimate from GBD 2019 (*APM*_2·5_) was summed with the household air pollution concentration to calculate the total air pollution concentration. The fraction of total personal exposure to PM_2·5_ air pollution contributed by household air pollution in each pixel was estimated to provide the household air pollution share (*HAP%*). Finally, the per-person air pollution concentration in each pixel was used as an input to the GBD 2019 risk (*IER*).^16^ curve for LRTIs to estimate a relative risk (*RR*) and population attributable fraction (*PAF*) for every PM_2·5_-associated outcome (*o*) in each pixel. The population attributable fraction is a counterfactual estimate of the disease burden explained by a given risk factor, based on the level of exposure and corresponding excess relative risk of disease.^16^ The population attributable fraction for LRTIs was combined with pixel-level estimates^21^ of under-5 LRTI mortality counts(*N*_i,c,t,o_) to estimate the count (*TAP N*) and rate of deaths from LRTIs that were attributable to total air pollution and specifically to household air pollution and ambient air pollution in each district.

All pixel-level indicators were population-weighted and aggregated to first (regions) and second (districts) administrative levels using shapefile boundaries from the Database of Global Administrative Areas shapefiles. We quantified within-country inequalities by using the range between the best-performing and worst-performing district for a given year and the average interpersonal difference, which estimates the average difference between any two districts in a country-year.^22^ Absolute and annualised rates of change from 2000 to 2018 were computed to quantify temporal trends over the study period. This annualised rate of change was applied to 2018 values to project the summary exposure value (a risk-weighted summary measure of exposure prevalence)^16^ to 2030 for assessment of the attainment of SDG indicator 7.1.2 (the proportion of population with primary reliance on clean fuels and technology, which is used as a tracking benchmark for SDG 7). We chose this target indicator because it corresponds most closely to our modelled proportion on the basis of previously published^23^ methods that were consistent with the GBD 2019 study.^16^ The annualised rate of change for solid-fuel use (*P*) was calculated at the draw level (*i*) for each pixel (*m*) by estimating the rate between each pair of adjacent years (*t*):

$$Annualisedrateofchange_{i,m,t}=\text{logit}(\frac{P_{i,m,t}}{P_{i,m,t-1}})$$

The annualised rate of change was then weighted across all years, such that more recent rates were additionally weighted. Weights (*W*) were defined as:

$$W_{t}={\left(t-200+1\right)}^{\omega }$$

For this analysis, ω was defined using draws from a distribution generated empirically for this indicator in the GBD 2019 study, with a mean value of 2·3 and an SD of 0·41. The weighted annualised rate of change for each pixel was generated for each unit as:

$$\begin{array}{c} Annualisedrateofchange=\text{logit}(\sum_{\text{2000}}^{\text{2018}}{\text{W}}_{\text{t}}*Annualised\\ rateofchange_{i,m}) \end{array}$$

Annualised rate of change estimates for 2018 were used to predict for 2030:

$$\begin{array}{c} {\text{Proj}}_{\text{i},\text{m},2030}{=\text{logit}}^{-1}(\text{logit}(P_{i,m,2018})+Annualisedrateof\\ change_{i,m}*(2030-2018)) \end{array}$$

Attainment probabilities for SDG indicator 7.1.2 were derived from the percentage of simulations in 2030 with summary exposure below 5%, a threshold chosen on the basis of estimates of solid-fuel use in high-income countries.^24^ We used the R-INLA package in R (version 4.1.3) for our analyses. All code used in the analysis is available online.

### Role of the funding source

The funder of the study had no role in the study design, data collection, data analysis, data interpretation, or the writing of the report.

## Results

**Figure 1:**
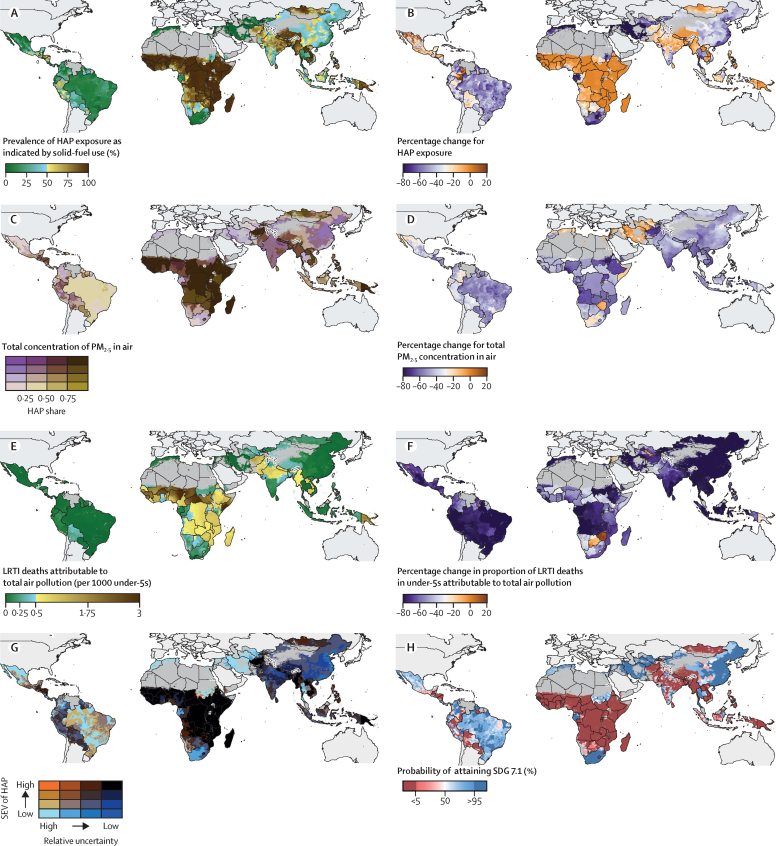
Prevalence of, and change from 2000 to 2018, in solid-fuel use, household air pollution, total air pollution, and deaths attributable to LRTI in children younger than 5 years in low-income and middle-income countries (A) Mean prevalence of solid-fuel use for cooking, 2018 (as indicated by primary fuel type). (B) Total concentration of PM_2·5_ in air by source, 2018. (C) Mean number of deaths from LRTIs (per 1000 children younger than 5 years) attributable to total concentration of PM_2·5_ in air, 2018. (D) Overlapping terciles of mean risk-weighted prevalence (SEV) of HAP in 2018 and relative uncertainty. These data were used for our projections of SDG attainment in 2030. (E) Percentage change in mean proportion of solid-fuel use for cooking, 2000 to 2018. (F) Percentage change in total concentration of PM_2·5_ in air, 2000 to 2018. (G) Percentage change in the proportion of LRTI deaths (per 1000 children younger than 5 years) attributable to total concentration of PM_2·5_ in air, 2000 to 2018. (H) Probability of attaining SDG 7.1 by 2030. All panels are aggregated to the second administrative-level unit. Maps reflect administrative boundaries, land cover, lakes, and population. Grey-coloured grid cells had fewer than ten people per 1 km × 1 km grid cell and were classified as barren or sparsely vegetated, or not included in this analysis. HAP=household air pollution. PM_2·5_=particulate matter of less than 2·5 μm in diameter. SEV=summary exposure value. LRTI=lower respiratory tract infection. SDG=Sustainable Development Goal.

**Figure 2:**
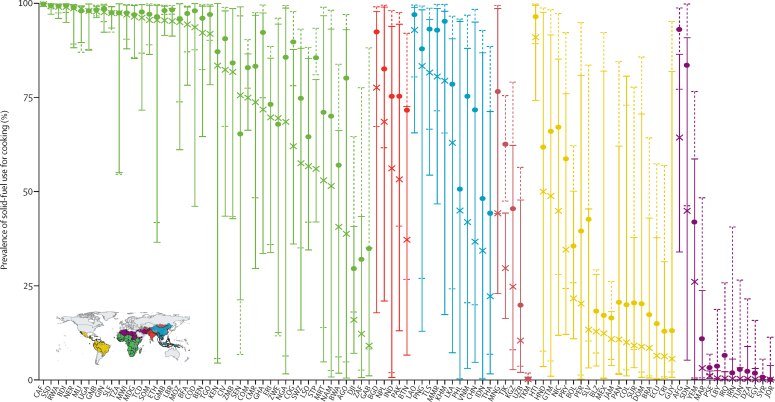
Geographical variations in the prevalence of solid-fuel use for cooking in 98 low-income and middle-income countries Each bar represents the range of the prevalence of solid-fuel use for cooking (as represented by primary fuel type) across all districts within each country. The Xs and dashed bars represent the mean and range in 2000, whereas the dots and solid bars represent the mean and range in 2018. Countries are grouped according to geographical region and coloured according to the Global Burden of Disease 2019 super-regions (see inset thumbnail).^16^ Each country is labelled by its ISO-3 code.

Subnational solid-fuel use prevalence was notably spatiotemporally heterogeneous (figure 1A), underscoring the importance of subnationally tracking cooking behaviours and corresponding measures of pollution. In 2018, 10·0% (95% uncertainty interval [UI] 7·4–13·9; appendix p 6) of the population in LMICs (593 million people) lived in districts where the prevalence of solid-fuel use exceeded 95%. National prevalence estimates masked substantial within-country heterogeneity (figure 2). For example, the average solid-fuel use prevalence in Guatemala was 52·0% (95% UI 40·0–63·2), but across just 140 km, prevalence ranged from 1·0% (0–3·3) in Zona 22, Guatemala City, to 91·6% (79·4–97·8) in Santa Eulalia, Huehuetenango. During the study period, the average interpersonal difference increased by 43·0% across all LMICs.

Prevalence of solid-fuel use fell across LMICs (figure 1E) from 67·8% (95% UI 67·1–69·0) in 2000 to 56·5% (53·8–57·9) in 2018, a relative decrease of 16·7% (14·3–21·6). Due to population growth, however, an additional 359 million (234 million–388 million) people were exposed to solid-fuel use in 2018. We estimated that, in 2018, 56·5% (53·9–57·9) of people in LMICs, roughly 3·38 billion (3·21 billion–3·45 billion) of the 5·96 billion in the study area (which rose from 4·4 billion in 2000) relied primarily on solid fuels for cooking. Projections for the year 2030 (figure 1D) suggest that only five countries (Iraq, Iran, Jordan, Syria, and Turkmenistan) across three regions, representing 3% of the 2018 study population, have a greater than 95% probability of achieving universal access to clean cooking fuels (figure 1H) in every district. District-level forecasting highlights the importance of using subnational estimation to monitor progress towards universal access. National trends suggest that Mexico has a 98% probability of meeting the threshold for universal access by 2030. However, this figure obscures local inequalities, because 14% of the population (nearly 19 million people in 2018) live in districts where the probability is less than 50%. In India, solid-fuel use prevalence has substantially decreased, and the national attainment probability is up to 33%. Locally, however, more than 35% of people (482 million people in 2018) lived in districts where the forecasted probability of attainment is less than 5%.

**Figure 3:**
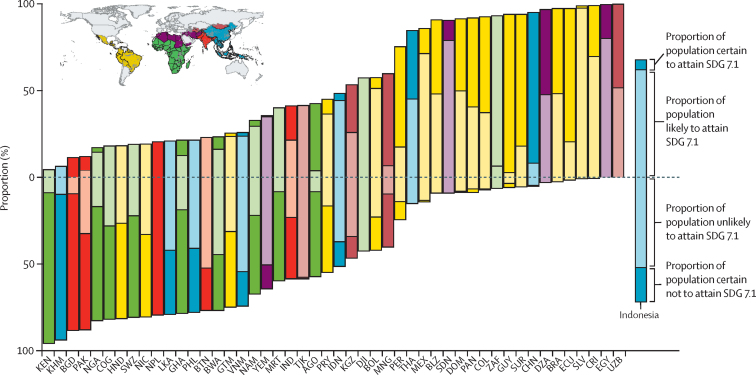
Projections of the probability of attainment of SDG 7.1 by 2030 Progress towards SDG 7.1 was monitored by estimating access to clean cooking fuel across the study period, calculating the annualised rate of change and forecasting to 2030. The bars represent the share of population living in districts that were projected (with 95% certainty) to be above (negative values) or below (positive values) a threshold of 5% for attainment in 2030. The translucent, lighter-coloured sections of the bars represent projections in districts with less certainty (greater than 50%, but less than 95%). The bars are coloured according to Global Burden of Disease 2019 super-regions (see inset thumbnail).^16^ Only the 48 countries where at least one district was projected to be above the threshold and at least one was projected to be below the threshold were included. Each country is labeled by its ISO-3 code. SDG=Sustainable Development Goal.

Moderate rates of change and the shortening timeline for achievement of the SDGs suggest that few districts that had not already achieved the target of universal access to clean cooking fuels in 2018 will do so by 2030. In 2018, 3·8 billion people in LMICs (ie, 65·7% of the population) lived in districts that did not meet the threshold for universal access to clean cooking fuel. Our projections suggest that only 22·0% (835 million) of these people live in a district that is forecasted to meet the threshold by 2030. Countries with the largest share of districts that were above the threshold in 2018 but on track to meet it by 2030 included Gabon (where 93·9% of the population that has yet to meet the goal lives in districts that are projected to meet it by 2030), China (82·9%), South Africa (66·5%), Mongolia (52·7%), Suriname (40·5%), Ecuador (27·6%), and Uzbekistan (24·6%). In 27 countries, every district is projected to remain above the 5% threshold by 2030 with high certainty (figure 1H). In the 48 countries containing both districts that are projected to meet the threshold and districts projected to fail to meet it, the proportion of the population living in a district that is not on track (as of 2018) ranged from less than 1% (14 459 people) in Uzbekistan to 95% (49 million people) in Kenya (figure 3).

**Figure 4:**
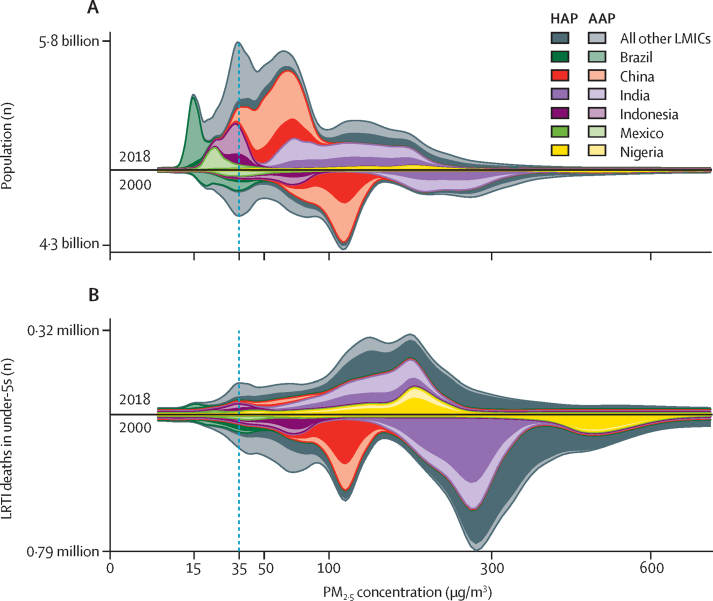
All-age population (A) and LRTI deaths attributable to total PM_2·5_ concentrations in air in children younger than 5 years (B) in 2000 and 2018, distributed as a function of PM_2·5_ Within each distribution, the darker shading represents the portion of air pollution contributed by household sources, whereas the lighter shading represents the portion contributed by ambient sources. The plotted data represent local smoothing of normalised distributions that were computed over 400 logarithmically spaced bins. The dashed vertical line indicates WHO's interim threshold for PM_2·5_ air pollution (35 μg/m^3^). The y-axis labels provide the total area under the curve. Data for all other LMICs included in the study are broken down by country in the appendix (p 42). LRTI=lower respiratory tract infection. HAP=household air pollution. AAP=ambient air pollution. PM_2·5_=particulate matter of less than 2·5 μm in diameter. LMICs=low-income and middle-income countries.

The total concentration of PM_2·5_ air pollution to which people were exposed was nearly halved (mean decrease 47·2% [95% UI 46·4–47·7]) from 2000 to 2018 (figure 1F). However, in 2018, only 14·9% (10·9–18·4) of people in LMICs lived in districts that met WHO's air quality interim target^25^ of 35 μg/m^3^ (figure 4A), and 80% of people were exposed to annual concentrations greater than 44 μg/m^3^ (for comparison, in 2000, 80% of the population lived in pixels with concentrations that were almost double that value, at 82 μg/m^3^ or more). Globally, the proportion of the total particulate matter concentration contributed by household air pollution decreased from 66·3% (64·8–70·0) in 2000 to 46·5% (43·2–47·8) in 2018. Household air pollution contributed most of the total PM_2·5_ air pollution in 69 of 98 countries in 2000, and 50 of 98 in 2018, suggesting a risk transition for the main source of particulate matter exposure in LMICs.

We focused our case study on the effects of particulate matter on child health, given the availability of analogous high-resolution geospatial estimates for mortality attributable to LRTIs in children younger than 5 years.^21^ Over the study period, the population attributable fraction of fatal LRTIs associated with total exposure to PM_2·5_ air pollution in all LMICs fell from 45·7% (95% UI 32·2–57·6) in 2000 to 38·4% (24·9–51·9) in 2018, a relative reduction of 16·5% (95% UI 9·0–26·5). Nearly half of fatal LRTIs in 2018 were attributable to total exposure to PM_2·5_ air pollution in sub-Saharan Africa (49·8% [36·4–61·4]). Across LMICs, most fatal LRTIs were attributable to total exposure to PM_2·5_ air pollution in 10·0% (0·0–22·3) of districts. We estimated that, across LMICs in 2018, 320 000 (218 000–408 000) children died from LRTIs attributable to air pollution (figure 1D) compared with 747 000 (533 000–932 000) in 2000. This reduction was driven largely by reductions in solid-fuel use: the number of LRTI deaths attributable to household air pollution in children younger than 5 years fell by 65·5% (62·3–68·6). However, an estimated 205 000 (147 000–257 000) children still died from LRTIs attributable to household air pollution in 2018. These deaths are now associated with lower pollution concentrations (figure 4B): the median was 202 μg/m^3^ in 2018, compared with 326 μg/m^3^ in 2000. Across LMICs, the share of attributable under-5 LRTI mortality that was driven by household versus ambient particulates fell from 79·6% (77·4–81·6) in 2000 to 64·3% (61·6–67·9) in 2018, signalling that household air pollution is still a crucial factor in under-5 LRTI deaths.^25^

**Figure 5:**
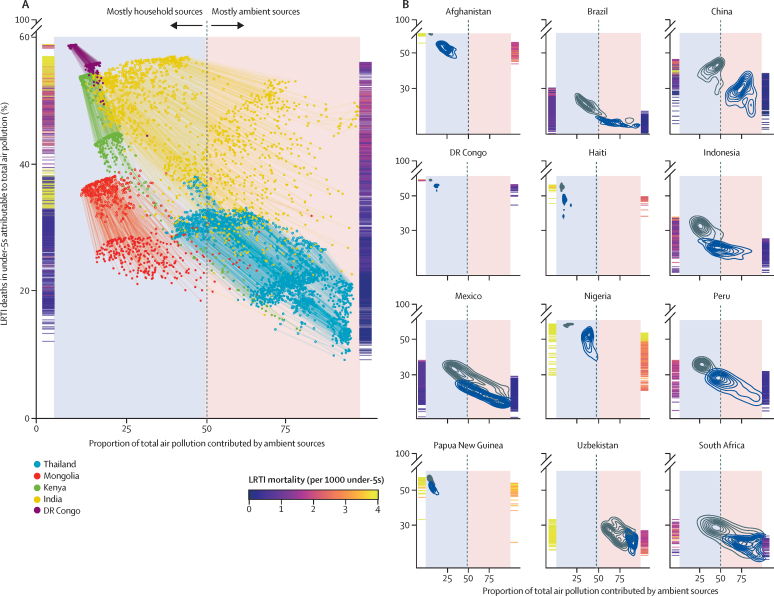
Air pollution risk transition, 2000–18 (A) Trends in the proportion of LRTI deaths attributed to total air pollution at the second administrative unit (district) level in five low-income and middle-income countries. These countries were chosen to exemplify different stages of air-pollution risk transition (all other countries included in the study are illustrated in the appendix [p 63]). The y-axis rugs indicate the gradient of background LRTI mortality rates for 2000 (left) and 2018 (right), illustrating the correlation between LRTI rates and the fraction attributable to total ambient air pollution. The lines connect a district to its preceding timepoint across the series. (B) Countries with the highest LRTI mortality in 2000 for each Global Burden of Disease subregion. The grey contours represent district-level distributions in 2000, whereas the navy represents distributions in 2018. In Thailand and South Africa, for example, reductions in household air pollution have resulted in less than a quarter of LRTIs being attributable to air pollution, whereas in China and India, similar reductions have been counteracted by rising ambient air pollution concentrations, which means that a larger share of LRTI deaths in children younger than 5 years continue to be attributable to total air pollution. LRTI=lower respiratory tract infection.

District-level burden estimates further underscored the risk transition occurring in LMICs: the concentrations of particulate matter to which populations were exposed decreased, and were increasingly driven by ambient sources (figure 5). This transition generally decreased the share of LRTIs attributable to particulate air pollution (figure 1E). However, as experienced in China, India, and Nigeria, sharply rising outdoor pollution superseded clean cooking adoption and offset potential burden reductions (figure 5). In some countries, the prevalence of fatal LRTIs (figure 1G) fell without a corresponding decline in household air pollution: in Laos, for example, the rate of fatal LRTIs per 1000 children younger than 5 years fell 73·8% (95% UI 66·3–80·9) between 2000 and 2018, while solid-fuel use prevalence decreased by only 3·5% (1·0–6·9). In the 19 countries where under-5 LRTI death rates still exceed two per 1000 children, however, the proportion of LRTI deaths attributable to household air pollution ranged from 21·4% (12·8–30·0) in Lesotho to 60·9% (44·8–75·7) in Somalia, suggesting that reduction of household air pollution remains fundamental to the elimination of preventable child mortality.

## Discussion

The results of our geospatial modelling study suggest that, despite progress since 2000, solid-fuel use continues to be widespread in LMICs, with uneven improvements driving regional and within-country inequalities. Our projections indicate that attaining the SDG target of universal access to clean cooking fuels by 2030 is improbable for many countries. Total particulate matter exposure fell between 2000 and 2018, but most people in LMICs continue to be exposed to PM_2·5_ concentrations far above the interim air quality target of 35 μg/m^3^. The fraction of total PM_2·5_ contributed by ambient air pollution rose, as growing outdoor concentrations in many geographies offset the health gains of cleaner cooking. The combination of a slow transition from solid-fuel use and displacement by ambient pollution sources supports a mandate for strengthening efforts to drive decreases in exposure to realise crucial health gains. The health effects of inaction are particularly relevant to vulnerable populations, such as children younger than 5 years, among whom more than a third of deaths from LRTIs are still attributable to air pollution.

The inclusion of clean energy in multinational initiatives such as the Global Forum on Child Pneumonia,^26^ climate change mitigation,^27^ and a web of interlinked SDG targets^9^ indicates that promotion of clean fuels will be crucial to the global development agenda in the coming decade. The energy-ladder hypothesis initially asserted that adoption of clean fuel sources was primarily driven by rising income, but subsequent research suggests that residential energy choices are influenced by a variety of complex socioeconomic forces, including community factors, agricultural practices, and dietary preferences.^14^, ^28^ Cooking is social, and campaigns to develop new energy markets by reducing prices and strengthening supply chains should be integrated with marketing and educational outreach activities to build knowledge and change household norms.^29^ Policies that include rural electrification and fuel subsidies have been efficacious, but our estimates support critiques that these campaigns need to be accelerated and redirected to target rural households that are continuing to fall behind in the adoption of clean cooking fuels.^30^

Ambitious programmes, such as India's Pradhan Mantri Ujjwala Yojana, have substantially increased adoption of clean fuels, but beneficiaries struggle to sustain regular use.^12^ Our estimates were derived from data for primary fuel type, and the widespread nature of stacking (ie, parallel use of multiple fuel types^31^) supports the value of a more specific proxy for clean cooking than that used by us and in most household surveys. The Multi-Tier Framework surveys for some countries show that the extent of stacking depends on local context: for example, most urban households in Cambodia stack,^32^ whereas more than 90% of households in Rwanda use one stove to meet their energy needs.^33^ Air-monitoring data help to clarify the direction and magnitude of the bias introduced by exposure misclassification as a result of stove stacking: compared with households that exclusively use their primary fuel for cooking, households that primarily use clean fuels but stack with a solid fuel have a much higher particulate matter differential than households who stack with a solid primary and clean secondary fuel, suggesting that primary fuel data underestimate the true burden of household air pollution.^34^

Ultimately, switching of primary fuel sources is an inadequate target for the reduction of household air pollution to levels that are acceptable for human health. While our analysis of primary fuel data shows the tremendous scale of residual solid-fuel use, it represents a narrow interpretation of SDG 7.1, and attainment of universal access to modern energy should incorporate solutions that acknowledge the contextual challenges of LMIC households (eg, power outages, fuel shortages, device malfunctions). Expanding the spatiotemporal coverage of nuanced surveys with more specific assessments of household energy behaviours would allow for future analyses to maintain our global scope while transitioning to more sophisticated indicators, like the Multi-Tier Framework data, to enable calculation of adequate proxies for total personal exposure to PM_2·5_. Likewise, other indicators relevant to the immediate housing environment^35^ should be integrated with these estimates to account for the synergistic effects they have on health outcomes.

Our study had several limitations. Although data^6^ suggest that use of kerosene as a fuel for cooking has substantially diminished globally during the study period, our exclusion of kerosene in calculations of household air pollution probably underestimates the burden in countries where kerosene is still commonly used,^36^ such as Equatorial Guinea, Djibouti, and urban areas in some Oceanian nations.^6^ Furthermore, the lack of comprehensive data for fuels used for heating and lighting restricted our analyses to cooking. Solid-fuel use for heating especially contributes substantially to seasonal concentrations of PM_2·5_ in LMICs with cooler climates.^37^ Thus our burden estimates are probably too low in important cases, such as China^38^ and South Africa.^39^ Restricting our epidemiological case study to LRTI deaths in children younger than 5 years is an additional limitation, because LRTIs represent only a fraction of the deleterious health effects of air pollution. Aside from the robust evidence for effects on other child health endpoints, including adverse birth outcomes^40^ and neurodevelopmental effects,^41^ PM_2·5_ pollution substantially affects adult health and is associated with chronic diseases like cardiovascular disease, chronic obstructive pulmonary disease, lung cancer, and diabetes.^7^ Finally, the accuracy of our estimates is a function of the quality and volume of survey data available, and uncertainty is substantial in areas where data are missing or less reliable. Our projections are based on a simplified method for applying annual trends to estimates for 2018, and are dependent on countries maintaining this rate of progress. Accordingly, they do not reflect the potential effect of new technologies or investments in clean energy, nor do they capture how widespread economic and societal disruptions—such as those associated with the COVID-19 pandemic—could negatively affect immediate and long-term trajectories. Pandemic-related effects on pollution are likely to be multifaceted and nuanced across settings, and could vary in relation to a combination of public health measures taken against COVID-19.^42^

There have been notable triumphs in the push for global transition to clean cooking fuels, yet our estimates clearly show the breadth of residual exposure to household air pollution and the substantial challenges faced in addressing the relevant SDG targets before 2030. Unless global and national investments in clean energy access and adoption increase substantially this decade, household particulate concentrations will remain far above acceptable levels and the bold SDG ambitions for clean energy will remain unmet. Heterogeneity in reductions in the prevalence of solid-fuel use emphasises growing inequality in much of the world, and the changing relationship between solid-fuel use and ambient air pollution underscores the importance of continuing to push for universal access to clean fuels. Collectively, our results emphasise the need to support access to clean cooking fuels to achieve development goals and can help to inform the design of geospatially targeted campaigns that combat areas of enduring deprivation by proactively targeting inequality.

## Data sharing

Data inputs and metadata (or, for inputs that cannot be shared due to data-use restrictions, relevant contact information) are available through the Global Health Data Exchange.

## Affiliations

Institute for Health Metrics and Evaluation (J J Frostad MPH, Q P Nguyen BS, M M Baumann BS, B F Blacker MPH, L B Marczak PhD, K E LeGrand MPH, K B Johnson MS, Prof L Dandona MD, Prof R Dandona PhD, Prof S D Dharmaratne MD, N M Martinez MPSA, B K Mayala PhD, T Mestrovic PhD, A H Mokdad PhD, Prof C J L Murray DPhil, Prof M Naghavi PhD, E E Spurlock MPH, C A Welgan BS, Prof M Brauer DSc, Prof S I Hay FMedSci, R C Reiner Jr PhD), Department of Health Metrics Sciences, School of Medicine (Prof R Dandona, Prof S D Dharmaratne, A H Mokdad, Prof C J L Murray, Prof M Naghavi, Prof S I Hay, R C Reiner Jr), University of Washington, Seattle, WA, USA; Department of Epidemiology, Emory University, Atlanta, GA, USA (A Deshpande MPH); Department of Epidemiology (K E Wiens PhD), Department of Radiology and Radiological Science (N Hafezi-Nejad MD, S Sheikhbahaei MD), Johns Hopkins University, Baltimore, MD, USA; Social Determinants of Health Research Center (M Abbasi-Kangevari MD), Injury Prevention and Safety Promotion Research Center (T Yousefinezhadi PhD), Shahid Beheshti University of Medical Sciences, Tehran, Iran (M Khayamzadeh MD); Zoonoses Research Center, Jahrom University of Medical Sciences, Jahrom, Iran (A Abdoli PhD); Research Center for Immunodeficiencies (H Abolhassani PhD, Prof N Rezaei PhD), Multiple Sclerosis Research Center (S Eskandarieh PhD), School of Medicine (N Hafezi-Nejad MD), Department of Environmental Health Engineering (Prof A Maleki PhD), Digestive Diseases Research Institute (Prof R Malekzadeh MD, S G Sepanlou MD), Department of Epidemiology and Biostatistics (M Mansournia PhD), Water Quality Research Center (R Mirzaei PhD), Metabolomics and Genomics Research Center (F Rahim PhD), Non-communicable Diseases Research Center (N Rezaei PhD, S Saeedi Moghaddam MSc), Endocrinology and Metabolism Research Institute (N Rezaei PhD), Tehran University of Medical Sciences, Tehran, Iran; Department of Biosciences and Nutrition, Karolinska University Hospital, Huddinge, Sweden (H Abolhassani PhD); Department of Pediatric Dentistry (Prof L G Abreu PhD), Department of Maternal and Child Nursing and Public Health (Prof D C Malta PhD), Department of Clinical Medicine (Prof B R Nascimento PhD), Clinical Hospital (Prof B R Nascimento PhD), Federal University of Minas Gerais, Belo Horizonte, Brazil; Department of Research, Philippine Institute for Development Studies, Quezon City, Philippines (M R M Abrigo PhD); Institute of Community and Public Health, Birzeit University, Ramallah, Palestine (Prof N M Abu-Rmeileh PhD); Department of Obstetrics & Gynecology, University of Texas, Galveston, TX, USA (V Adekanmbi PhD); Trivedi School of Biosciences, Ashoka University, Sonipat, India (Prof A Agrawal PhD); Section of General Internal Medicine, Baylor College of Medicine, Houston, TX, USA (Prof A Agrawal); Department of Epidemiology, Jimma University, Jimma, Ethiopia (M B Ahmed MPH); Australian Center for Precision Health, University of South Australia, Adelaide, SA, Australia (M B Ahmed); John T Milliken Department of Internal Medicine, Washington University in St Louis, St Louis, MO, USA (Z Al-Aly MD); Clinical Epidemiology Center, US Department of Veterans Affairs, St Louis, MO, USA (Z Al-Aly); Environmental Health Department (S M A Dahlawi PhD), Forensic Medicine Division (Prof R G Menezes MD), Imam Abdulrahman Bin Faisal University, Dammam, Saudi Arabia (F M Alanezi PhD); Center for Health Systems Research (J E Alcalde-Rabanal PhD), Center for Nutrition and Health Research (E Denova-Gutiérrez DSc), National Institute of Public Health, Cuernavaca, Mexico; Health Management and Economics Research Center (V Alipour PhD, J Arabloo PhD, A Rezapour PhD), Department of Health Economics (V Alipour), Hospital Management Research Center (S Azari PhD), Department of Medical Laboratory Sciences (F Dorostkar PhD), Minimally Invasive Surgery Research Center (A Kabir MD), Comprehensive Research Laboratory (R Mirzaei PhD), Preventive Medicine and Public Health Research Center (M Moradi-Lakeh MD), Iran University of Medical Sciences, Tehran, Iran; Pediatric Intensive Care Unit (K A Altirkawi MD, M Temsah MD), Internal Medicine Department (Y Mohammad MD), King Saud University, Riyadh, Saudi Arabia; Research Group in Hospital Management and Health Policies (Prof N Alvis-Guzman PhD), Department of Economic Sciences (N J Alvis-Zakzuk MSc), Universidad de la Costa (University of the Coast), Barranquilla, Colombia; Research Group in Health Economics, University of Cartagena, Cartagena, Colombia (Prof N Alvis-Guzman PhD); National Health Observatory (N J Alvis-Zakzuk MSc), Colombian National Health Observatory (C A Castañeda-Orjuela MD), National Institute of Health, Bogota, Colombia; Department of Biomedical Science, University of Cape Coast, Cape Coast, Ghana (A K Amegah PhD); Department of Health Services Management, Khomein University of Medical Sciences, Khomein, Iran (S Amini PhD); Department of Radiology and Nuclear Medicine (F Amiri MSc), Research Center for Environmental Determinants of Health (N Fattahi PhD, Prof M Pirsaheb PhD, Prof E Sadeghi PhD, K Sharafi PhD), Substance Abuse Prevention Research Center (B Mansouri PhD), Department of Rehabilitation and Sports Medicine (M Mirzaei MSc), Department of Health Education and Health Promotion (S Siabani PhD), Kermanshah University of Medical Sciences, Kermanshah, Iran; Maternal and Child Wellbeing, African Population and Health Research Center, Nairobi, Kenya (D A Amugsi PhD); Department of Pharmacy (Prof R Ancuceanu PhD), Department of Cardiology (C Andrei PhD), Department of General Surgery (I Negoi PhD), Carol Davila University of Medicine and Pharmacy, Bucharest, Romania; Department of Statistics and Econometrics (Prof T Andrei PhD, Prof M Ausloos PhD, Prof C Herteliu PhD), Bucharest University of Economic Studies, Bucharest, Romania; Agribusiness Study Program, Sebelas Maret University, Surakarta, Indonesia (E Antriyandarti DrAgrSc); Department of Parasitology, Mazandaran University of Medical Sciences, Sari, Iran (D Anvari PhD); Department of Parasitology, Iranshahr University of Medical Sciences, Iranshahr, Iran (D Anvari); Social Determinants of Health Research Center, Birjand University of Medical Sciences, Birjand, Iran (M Arab-Zozani PhD); Department of Immunology, Zanjan University of Medical Sciences, Zanjan, Iran (S Athari PhD); School of Business (Prof M Ausloos PhD), Department of Health Sciences (P H Lee PhD), University of Leicester, Leicester, UK; School of Indigenous Studies, University of Western Australia, Perth, WA, Australia (G Ayano MSc); School of Public Health, Curtin University, Perth, WA, Australia (G Ayano); Department of Nursing, Debre Berhan University, Debre Berhan, Ethiopia (Y A Aynalem MSc, W S Shiferaw MSc); Department of Forensic Science, Government Institute of Forensic Science, Nagpur, India (A D Badiye MSc, N Kapoor MSc); Unit of Biochemistry (A A Baig PhD), Universiti Sultan Zainal Abidin (Sultan Zainal Abidin University), Kuala Terengganu, Malaysia; Department of Environmental Health Engineering, Sri Ramachandra Medical College and Research Institute, Chennai, India (Prof K Balakrishnan PhD); Department of Hypertension, Medical University of Lodz, Lodz, Poland (Prof M Banach PhD); Polish Mothers’ Memorial Hospital Research Institute, Lodz, Poland (Prof M Banach); Center for Primary Care (S Basu PhD), Division of General Internal Medicine (Prof A Sheikh MD), Harvard University, Boston, MA, USA; School of Public Health (S Basu PhD), Department of Primary Care and Public Health (J Car PhD, Prof A Majeed MD, Prof S Rawaf MD), Imperial College Business School (D Kusuma DSc), WHO Collaborating Centre for Public Health Education and Training (D L Rawaf MD), Imperial College London, London, UK; School of Public Health, Dr D Y Patil University, Mumbai, India (Prof N Bedi MD); Department of Epidemiology (M Khan MD), Jazan University, Jazan, Saudi Arabia (Prof N Bedi MD); School of the Environment (Prof M L Bell PhD), Yale School of Public Health—Social and Behavioral Sciences (E E Spurlock MPH), Yale University, New Haven, CT, USA; Nuffield Department of Population Health (D A Bennett PhD, B Lacey PhD), Nuffield Department of Clinical Medicine (N J Henry BS), Centre for Tropical Medicine and Global Health (S Lewycka PhD), The George Institute for Global Health (Prof S Yaya PhD), University of Oxford, Oxford, UK; Department of Statistical and Computational Genomics, National Institute of Biomedical Genomics, Kalyani, India (K Bhattacharyya MSc); Department of Statistics, University of Calcutta, Kolkata, India (K Bhattacharyya); Centre for Global Child Health, University of Toronto, Toronto, ON, Canada (Prof Z A Bhutta PhD); Centre of Excellence in Women & Child Health (Prof Z A Bhutta), Division of Women and Child Health (J K Das MD), Aga Khan University, Karachi, Pakistan; Institute of Soil and Environmental Sciences, University of Agriculture, Faisalabad, Faisalabad, Pakistan (S Bibi PhD, S Ullah PhD); Department of Veterinary Medicine, Islamic Azad University, Kermanshah, Iran (S Bohlouli PhD); Transport and Road Safety Research Centre, University of New South Wales, Sydney, NSW, Australia (S Boufous PhD); University of Genoa, Genoa, Italy (N L Bragazzi PhD); Department of Epidemiology, University of Florida, Gainesville, FL, USA (D Braithwaite PhD); Cancer Population Sciences Program, University of Florida Health Cancer Center, Gainesville, FL, USA (D Braithwaite); Department of Community Medicine, Employee State Insurance Post Graduate Institute of Medical Sciences and Research, Bangalore, India (Prof S Burugina Nagaraja MD); School of Public Health and Health Systems, University of Waterloo, Waterloo, ON, Canada (Z A Butt PhD); Al Shifa School of Public Health, Al Shifa Trust Eye Hospital, Rawalpindi, Pakistan (Z A Butt); Institute of Microengineering, Federal Polytechnic School of Lausanne, Lausanne, Switzerland (F Caetano dos Santos PhD); Centre for Population Health Sciences, Nanyang Technological University, Singapore, Singapore (J Car PhD); Department of Health Care, Metropolitan Autonomous University, Mexico City, Mexico (Prof R Cárdenas DSc); Research Unit on Applied Molecular Biosciences (UCIBIO), University of Porto, Porto, Portugal (Prof F Carvalho PhD); Department of Psychiatry (Prof J Castaldelli-Maia PhD, Y Wang PhD), Center for Clinical and Epidemiological Research (A C Goulart PhD), Department of Internal Medicine (A C Goulart PhD), Department of Pathology and Legal Medicine (M R Tovani-Palone PhD), University of São Paulo, São Paulo, Brazil; Epidemiology and Public Health Evaluation Group, National University of Colombia, Bogota, Colombia (C A Castañeda-Orjuela MD); Mary MacKillop Institute for Health Research, Australian Catholic University, Melbourne, VIC, Australia (Prof E Cerin PhD); School of Public Health, University of Hong Kong, Hong Kong, China (Prof E Cerin); Department of Public Health, Texila American University, Georgetown, Guyana (S Chattu PhD); Department of Community Medicine, Datta Meghe Institute of Medical Sciences, Sawangi, India (V Chattu MD); Saveetha Medical College, Saveetha University, Chennai, India (V Chattu); Center for Cancer Epidemiology, Tata Memorial Hospital, Navi Mumbai, India (Prof P Chaturvedi MD); Department of Head Neck Surgery, Tata Memorial Hospital, Mumbai, India (Prof P Chaturvedi); Department of Research, Dr D Y Patil University, Pune, India (S Chaturvedi PhD); Heidelberg Institute of Global Health, Heidelberg University, Heidelberg, Germany (S Chen DSc, J De Neve MD, B Moazen MSc); Center for Biomedicine and Community Health, VNU-International School, Hanoi, Viet Nam (D Chu PhD); Department of Health Informatics (S Chung PhD), Division of Psychology and Language Sciences (M Kumar PhD), University College London, London, UK; Health Data Research UK, London, UK (S Chung PhD); IRCCS Istituto Ortopedico Galeazzi (G Damiani MD), Department of Clinical Sciences and Community Health (Prof C La Vecchia MD), University of Milan, Milan, Italy; Department of Dermatology, Case Western Reserve University, Cleveland, OH, USA (G Damiani); Department of Research (A Pandey PhD), Public Health Foundation of India, Gurugram, India (Prof L Dandona MD, Prof R Dandona PhD, G Kumar PhD); Indian Council of Medical Research, New Delhi, India (Prof L Dandona MD); Department of Information Technology (A M Darwesh PhD), Department of Computer Science (M Hosseinzadeh PhD), Diplomacy and Public Relations Department (A Omar Bali PhD), University of Human Development, Sulaymaniyah, Iraq; Asian Institute of Public Health University, Bhubaneswar, India (Prof A P Dash DSc); Department of Population and Development, Latin American Faculty of Social Sciences Mexico, Mexico City, Mexico (C A Dávila-Cervantes PhD); Australian Institute for Suicide Research and Prevention, Griffith University, Mount Gravatt, QLD, Australia (Prof D De Leo DSc); Institute of Public health (G D Demissie MPH), School of Nursing (H B Netsere MS), University of Gondar, Gondar, Ethiopia; Centre for Atmospheric Sciences, Indian Institute of Technology Delhi, New Delhi, India (S Dey PhD); Department of Community Medicine, University of Peradeniya, Peradeniya, Sri Lanka (Prof S D Dharmaratne MD); Health Research Section, Nepal Health Research Council, Kathmandu, Nepal (M Dhimal PhD); Department of Microbiology, Far Western University, Mahendranagar, Nepal (G P Dhungana MSc); Center of Complexity Sciences, National Autonomous University of Mexico, Mexico City, Mexico (Prof D Diaz PhD); Faculty of Veterinary Medicine and Zootechnics, Autonomous University of Sinaloa, Culiacán Rosales, Mexico (Prof D Diaz PhD); Health Promotion and Education (I O Dipeolu PhD), Department of Health Promotion and Education (S E Ibitoye MPH), Department of Community Medicine (O S Ilesanmi PhD), Department of Medicine (Prof M O Owolabi DrM), University of Ibadan, Ibadan, Nigeria; Department of Health Policy and Management (L Doshmangir PhD), Department of Medical Surgical Nursing (M Ghafourifard PhD), Tabriz University of Medical Sciences, Tabriz, Iran; School of Medicine (Prof A R Duraes PhD), Institute of Collective Health (Prof D Rasella PhD), Federal University of Bahia, Salvador, Brazil; Department of Internal Medicine (Prof A R Duraes PhD), Escola Bahiana de Medicina e Saúde Pública (Bahiana School of Medicine and Public Health), Salvador, Brazil; School of Health Sciences (H A Edinur PhD), Universiti Sains Malaysia (University of Science Malaysia), Kubang Kerian, Malaysia; Community Health Nursing (F Efendi PhD), Faculty of Public Health (S Martini PhD), Universitas Airlangga (Airlangga University), Surabaya, Indonesia; School of Nursing and Midwifery, La Trobe University, Melbourne, VIC, Australia (F Efendi PhD, M Rahman PhD); Pediatric Dentistry and Dental Public Health Department, Alexandria University, Alexandria, Egypt (Prof M El Tantawi PhD); Division of Non-communicable Diseases, Ministry of Public Health and Population, Dubai, United Arab Emirates (I Fadhil PhD); Torrens University Australia, Adelaide, SA, Australia (N K Fauk MSc); Institute of Resource Governance and Social Change, Kupang, Indonesia (N K Fauk MSc); Department of Neurobiology, Karolinska Institute, Stockholm, Sweden (S Fereshtehnejad PhD); Division of Neurology (S Fereshtehnejad), School of International Development and Global Studies (Prof S Yaya PhD), University of Ottawa, Ottawa, ON, Canada; Department of Child Dental Health, Obafemi Awolowo University, Ile-Ife, Nigeria (Prof M O Folayan FWACS); Department of Medical Parasitology (M Foroutan PhD), Faculty of Medicine (M Foroutan PhD), Department of Biostatistics (N Kamyari PhD), Abadan University of Medical Sciences, Abadan, Iran; Department of Dermatology, Kobe University, Kobe, Japan (T Fukumoto PhD); Department of Community Medicine (Prof A M Gaidhane MD, Prof Z Quazi Syed PhD), Global Evidence Synthesis Initiative (Prof M Khatib PhD), Datta Meghe Institute of Medical Sciences, Wardha, India; School of Public Health (A Ghashghaee BSc), Institute for Prevention of Non-communicable Diseases (R Kalhor PhD), Health Services Management Department (R Kalhor PhD), Qazvin University of Medical Sciences, Qazvin, Iran; Faculty of Allied Health Sciences, University of Lahore, Lahore, Pakistan (Prof S Gilani PhD); Afro-Asian Institute, Lahore, Pakistan (Prof S Gilani); Adelaide Medical School (T K Gill PhD), Centre for Heart Rhythm Disorders (J Noubiap MD), University of Adelaide, Adelaide, SA, Australia; Postgraduate Program in Epidemiology, Federal University of Rio Grande do Sul, Porto Alegre, Brazil (Prof B N G Goulart DSc); Department of Dermatology (A Grada MD), Health Informatic Lab (T Javaheri PhD), Department of Computer Science (R Rawassizadeh PhD), Boston University, Boston, MA, USA; Department of Family and Community Medicine, University Of Sulaimani, Sulaimani, Iraq (M I M Gubari PhD); UO Neurologia, Salute Pubblica e Disabilità, Fondazione IRCCS Istituto Neurologico Carlo Besta (Neurology, Public Health and Disability Unit, Carlo Besta Neurological Institute), Milan, Italy (D Guido PhD); Department of Epidemiology and Preventive Medicine (Prof Y Guo PhD), School of Public Health and Preventive Medicine (S Li PhD), School of Clinical Sciences at Monash Health (S Zaman MPH), Monash University, Melbourne, VIC, Australia; Department of Epidemiology, Binzhou Medical University, Yantai City, China (Prof Y Guo PhD); Department of Epidemiology and Biostatistics, University of South Carolina, Columbia, SC, USA (R Gupta MPH); Centre for Non-communicable Diseases and Nutrition, BRAC University, Dhaka, Bangladesh (R Gupta MPH); Department of Preventive Cardiology, Eternal Heart Care Centre & Research Institute, Jaipur, India (Prof R Gupta MD); Department of Medicine, Mahatma Gandhi University Medical Sciences, Jaipur, India (Prof R Gupta); Department of Epidemiology and Psychosocial Research, Ramón de la Fuente Muñiz National Institute of Psychiatry, Mexico City, Mexico (R A Gutiérrez PhD); Department of Family and Community Medicine, Arabian Gulf University, Manama, Bahrain (Prof R R Hamadeh PhD); Department of Zoology and Entomology, Al Azhar University, Cairo, Egypt (A I Hasaballah PhD); Gastrointestinal and Liver Diseases Research Center (S Hassanipour PhD), Caspian Digestive Disease Research Center (S Hassanipour), Department of Environmental Health Engineering (J Jaafari PhD), Guilan University of Medical Sciences, Rasht, Iran; Institute of Pharmaceutical Sciences, University of Veterinary and Animal Sciences, Lahore, Pakistan (K Hayat MS); Department of Pharmacy Administration and Clinical Pharmacy, Xian Jiaotong University, Xian, China (K Hayat MS); Center for Environmental and Respiratory Health Research, University of Oulu, Oulu, Finland (B Heibati PhD); Department of Nursing (R Heidari-Soureshjani MSc), Department of Clinical Biochemistry (A Mosapour PhD), Tarbiat Modares University, Tehran, Iran; School of Business, London South Bank University, London, UK (Prof C Herteliu PhD); Institute of Research and Development, Duy Tan University, Da Nang, Viet Nam (M Hosseinzadeh PhD); Faculty of Medicine of Tunis, University Tunis El Manar, Tunis, Tunisia (Prof M Hsairi MPH); Department of Epidemiology and Health Statistics, Central South University, Changsha, China (Prof G Hu PhD); Department of Community Medicine (O S Ilesanmi PhD), Department of Medicine (Prof M O Owolabi DrM), University College Hospital, Ibadan, Ibadan, Nigeria; Faculty of Medicine (I M Ilic PhD, Prof M M Santric-Milicevic PhD), School of Public Health and Health Management (Prof M M Santric-Milicevic), University of Belgrade, Belgrade, Serbia; Department of Epidemiology, University of Kragujevac, Kragujevac, Serbia (Prof M D Ilic PhD); Independent Consultant, Tabriz, Iran (S N Irvani MD); Institute for Physical Activity and Nutrition, Deakin University, Burwood, VIC, Australia (S Islam PhD); Sydney Medical School (S Islam PhD), School of Public Health (L D Knibbs PhD), School of Veterinary Science (B B Singh PhD), University of Sydney, Sydney, NSW, Australia; School of Health Systems and Public Health, University of Pretoria, Pretoria, South Africa (C C D Iwu MPH); Institute of Advanced Manufacturing Technologies, Peter the Great St Petersburg Polytechnic University, St Petersburg, Russia (Prof M Jakovljevic PhD); Institute of Comparative Economic Studies, Hosei University, Tokyo, Japan (Prof M Jakovljevic); Department of Community Medicine, Dr Baba Saheb Ambedkar Medical College & Hospital, Delhi, India (R P Jha MSc); Department of Community Medicine, Banaras Hindu University, Varanasi, India (R P Jha); Vanke School of Public Health, Tsinghua University, Beijing, China (J S Ji DSc); Institute of Molecular and Clinical Ophthalmology Basel, Basel, Switzerland (Prof J B Jonas MD); Department of Ophthalmology, Heidelberg University, Mannheim, Germany (Prof J B Jonas MD); School of Public Health, University College Cork, Cork, Ireland (Z Kabir PhD); Department of Forensic Medicine and Toxicology, All India Institute of Medical Sciences, Jodhpur, India (T Kanchan MD); Department of Epidemiology, Biostatistics and Clinical Research (Prof U Kapil MD), Department of Radiation Oncology (Prof G K Rath MD), Department of Psychiatry (Prof R Sagar MD), All India Institute of Medical Sciences, New Delhi, India; International Research Center of Excellence, Institute of Human Virology Nigeria, Abuja, Nigeria (G A Kayode PhD); Julius Centre for Health Sciences and Primary Care (G A Kayode PhD), Institute for Risk Assessment Sciences (E Traini MSc), Utrecht University, Utrecht, Netherlands; Open, Distance and eLearning Campus (Prof P N Keiyoro PhD), Department of Psychiatry (M Kumar PhD), University of Nairobi, Nairobi, Kenya; Department of Public Health, Jordan University of Science and Technology, Irbid, Jordan (Prof Y S Khader PhD); School of Food and Agricultural Sciences, University of Management and Technology, Lahore, Pakistan (N Khalid PhD); Department of Epidemiology and Biostatistics, Health Services Academy, Islamabad, Pakistan (E A Khan MPH); Department of Population Science, Jatiya Kabi Kazi Nazrul Islam University, Mymensingh, Bangladesh (M Khan PhD); Faculty of Health and Wellbeing, Sheffield Hallam University, Sheffield, UK (K Khatab PhD); College of Arts and Sciences, Ohio University, Zanesville, OH, USA (K Khatab); Department of Medical Parasitology (M M Khater MD), Public Health and Community Medicine Department (M R Salem MD), Cairo University, Cairo, Egypt; The Iranian Academy of Medical Sciences, Tehran, Iran (M Khayamzadeh MD); Department of Public Health, New Mexico State University, Las Cruces, NM, USA (Prof J Khubchandani PhD); Institute of Health Services Research (G Kim PhD, Prof E Park PhD), Department of Preventive Medicine (Prof E Park PhD), College of Medicine (Prof J Shin MD), Yonsei University, Seoul, South Korea; School of Traditional Chinese Medicine, Xiamen University Malaysia, Sepang, Malaysia (Y Kim PhD); Department of Nutrition, Simmons University, Boston, MA, USA (R W Kimokoti MD); School of Health Sciences, Kristiania University College, Oslo, Norway (Prof A Kisa PhD); Department of Global Community Health and Behavioral Sciences, Tulane University, New Orleans, LA, USA (Prof A Kisa); Department of Nursing and Health Promotion, Oslo Metropolitan University, Oslo, Norway (S Kisa PhD); Department of Internal and Pulmonary Medicine, Sheri Kashmir Institute of Medical Sciences, Srinagar, India (Prof P A Koul MD); Biomedical Research Networking Center for Mental Health Network, San Juan de Dios Sanitary Park, Sant Boi de Llobregat, Spain (A Koyanagi MD); Catalan Institution for Research and Advanced Studies, Barcelona, Spain (A Koyanagi MD); Department of Anthropology, Panjab University, Chandigarh, India (Prof K Krishan PhD); Faculty of Public Health, University of Indonesia, Depok, Indonesia (D Kusuma DSc); National Institute for Health Research Oxford Biomedical Research Centre, Oxford, UK (B Lacey PhD); Department of Community and Family Medicine, University of Baghdad, Baghdad, Iraq (F H Lami PhD); Division of Cancer Epidemiology and Genetics, National Cancer Institute, Rockville, MD, USA (Q Lan PhD); Department of Otorhinolaryngology, Father Muller Medical College, Mangalore, India (S Lasrado MS); International Society Doctors for the Environment, Arezzo, Italy (P Lauriola MD); Oxford University Clinical Research Unit, Wellcome Trust Asia Programme, Hanoi, Viet Nam (S Lewycka PhD); Center for Integration of Data and Health Knowledge (D B Machado PhD), Oswald Cruz Foundation (FIOCRUZ), Salvador, Brazil; Centre for Global Mental Health, London School of Hygiene & Tropical Medicine, London, UK (D B Machado); Grants, Innovation and Product Development Unit, South African Medical Research Council, Cape Town, South Africa (P W Mahasha PhD); Department of Public Health, Urmia University of Medical Science, Urmia, Iran (M Maheri PhD); Environmental Health Research Center, Kurdistan University of Medical Sciences, Sanandaj, Iran (Prof A Maleki PhD); Non-communicable Disease Research Center (Prof R Malekzadeh MD, S G Sepanlou MD), Health Policy Research Center (S Shahabi PhD), Shiraz University of Medical Sciences, Shiraz, Iran; Indonesian Public Health Association, Surabaya, Indonesia (S Martini PhD); Campus Caucaia, Federal Institute of Education, Science and Technology of Ceará, Caucaia, Brazil (F R Martins-Melo PhD); ICF International, Demographic and Health Surveys Program, Rockville, MD, USA (B K Mayala PhD); Department of Neurology, Janakpuri Super Specialty Hospital Society, New Delhi, India (Prof M Mehndiratta MD); Department of Neurology, Govind Ballabh Institute of Medical Education and Research, New Delhi, India (Prof M Mehndiratta); Peru Country Office, United Nations Population Fund, Lima, Peru (W Mendoza MD); Department of Reproductive Health and Population Studies, Bahir Dar University, Bahir Dar, Ethiopia (E W Mengesha MPH); Breast Surgery Unit, Helsinki University Hospital, Helsinki, Finland (T J Meretoja MD); University of Helsinki, Helsinki, Finland (T J Meretoja); University Centre Varazdin, University North, Varazdin, Croatia (T Mestrovic PhD); Woman-Mother-Child Department, Lausanne University Hospital, Lausanne, Switzerland (I Michalek PhD); Internal Medicine Programme, Kyrgyz State Medical Academy, Bishkek, Kyrgyzstan (Prof E M Mirrakhimov PhD); Department of Atherosclerosis and Coronary Heart Disease, National Center of Cardiology and Internal Disease, Bishkek, Kyrgyzstan (Prof E M Mirrakhimov); Institute of Addiction Research, Frankfurt University of Applied Sciences, Frankfurt, Germany (B Moazen MSc); Department of Epidemiology and Biostatistics, Shahrekord University of Medical Sciences, Shahrekord, Iran (A Mohammadian-Hafshejani PhD); Health Systems and Policy Research Unit (S Mohammed PhD), Department of Community Medicine (M B Sufiyan MD), Ahmadu Bello University, Zaria, Nigeria; Department of Health Care Management, Technical University of Berlin, Berlin, Germany (S Mohammed PhD); Clinical Epidemiology and Public Health Research Unit, Burlo Garofolo Institute for Maternal and Child Health, Trieste, Italy (L Monasta DSc, L Ronfani PhD, E Traini MSc); Computer, Electrical, and Mathematical Sciences and Engineering Division, King Abdullah University of Science and Technology, Thuwal, Saudi Arabia (P Moraga PhD); International Laboratory for Air Quality and Health, Queensland University of Technology, Brisbane, QLD, Australia (Prof L Morawska PhD); Department of Clinical Biochemistry (A Mosapour PhD), Social Determinants of Health Research Center (S Mouodi PhD), Babol University of Medical Sciences, Babol, Iran; Department of Fruit and Vegetable Product Technology, Prof Wacław Dąbrowski Institute of Agricultural and Food Biotechnology State Research Institute, Warsaw, Poland (Prof A Mousavi Khaneghah PhD); Department of Endocrinology & Metabolism, Institute of Post-Graduate Medical Education and Research and Seth Sukhlal Karnani Memorial Hospital, Kolkata, India (Prof S Mukhopadhyay MD); Scientific Communications Department, Invitae, Boulder, CO, USA (S B Munro PhD); Research and Analytics Department, Initiative for Financing Health and Human Development, Chennai, India (A J Nagarajan MTech); Department of Research and Analytics, Bioinsilico Technologies, Chennai, India (A J Nagarajan); Department of Pulmonary Medicine, Government Medical College Trivandrum, Trivandrum, India (S Nair MD); Health Action by People, Trivandrum, India (S Nair); Suraj Eye Institute, Nagpur, India (V Nangia MD); Department of Pediatrics, Arak University of Medical Sciences, Arak, Iran (J Nazari MD); Department of General Surgery (I Negoi PhD), Emergency Hospital of Bucharest, Bucharest, Romania; College of Medicine and Health Sciences, Bahir Dar University, Gondar, Ethiopia (H B Netsere MS); Department of Biological Sciences, University of Embu, Embu, Kenya (J W Ngunjiri DrPH); Institute for Global Health Innovations, Duy Tan University, Hanoi, Viet Nam (H L T Nguyen MPH); Administrative and Economic Sciences Department (Prof B Oancea PhD), University of Bucharest, Bucharest, Romania; Translational Health Research Institute, Western Sydney University, Sydney, NSW, Australia (F A Ogbo PhD); Department of Preventive Medicine, Kyung Hee University, Dongdaemun-gu, South Korea (I Oh PhD); Department of Psychiatry and Behavioural Neurosciences (A T Olagunju MD), Population Health Research Institute (T Sathish PhD), McMaster University, Hamilton, ON, Canada; Department of Psychiatry, University of Lagos, Lagos, Nigeria (A T Olagunju MD); Centre for Healthy Start Initiative, Lagos, Nigeria (B O Olusanya PhD, J O Olusanya MBA); Department of Pharmacology and Therapeutics, University of Nigeria Nsukka, Enugu, Nigeria (Prof O E Onwujekwe PhD); Laboratory of Public Health Indicators Analysis and Health Digitalization, Moscow Institute of Physics and Technology, Dolgoprudny, Russia (N Otstavnov BA, S S Otstavnov PhD); Department of Project Management, National Research University Higher School of Economics, Moscow, Russia (S S Otstavnov PhD); Department of Respiratory Medicine, Jagadguru Sri Shivarathreeswara Academy of Health Education and Research, Mysore, India (Prof M P A DNB); Department of Medical Humanities and Social Medicine, Kosin University, Busan, South Korea (Prof E Park PhD); Department of Poverty, Gender and Youth, Population Council, New Delhi, India (S K Patel PhD); Center of Excellence in Behavioral Medicine, Nguyen Tat Thanh University, Ho Chi Minh City, Viet Nam (H Q Pham MD, G T Vu BA); Department of Cardiology, University of Bern, Bern, Switzerland (T Pilgrim MD); HIV and Mental Health Department, Integrated Development Foundation Nepal, Kathmandu, Nepal (K N Pokhrel PhD); University Medical Center Groningen (Prof M J Postma PhD), School of Economics and Business (Prof M J Postma PhD), University of Groningen, Groningen, Netherlands; School of Engineering, Macquarie University, Sydney, NSW, Australia (N Rabiee PhD); Pohang University of Science and Technology, Pohang, South Korea (N Rabiee); College of Medicine, University of Central Florida, Orlando, FL, USA (A Radfar MD); Department of Community Medicine, Maharishi Markandeshwar Medical College & Hospital, Solan, India (M Rahman PhD); School of Nursing and Healthcare Professions, Federation University Australia, Berwick, VIC, Australia (M Rahman); Future Technology Research Center, National Yunlin University of Science and Technology, Yunlin, Taiwan (A Rahmani PhD); Research Department, Policy Research Institute, Kathmandu, Nepal (C L Ranabhat PhD); Health and Public Policy Department, Global Center for Research and Development, Kathmandu, Nepal (C L Ranabhat PhD); Department of Oral Pathology, Sharavathi Dental College and Hospital, Shimogga, India (S Rao MDS); Department of Forensic Medicine and Toxicology (Prof P Rastogi MD), Kasturba Medical College (Prof B Unnikrishnan MD), Manipal Academy of Higher Education, Mangalore, India; University College London Hospitals, London, UK (D L Rawaf MD); Academic Public Health England, Public Health England, London, UK (Prof S Rawaf MD); School of Health, Medical and Applied Sciences, CQ University, Sydney, NSW, Australia (L Rawal PhD); School of Medicine and Translational Health Research Institute, Western Sydney University, Campbelltown, NSW, Australia (Prof A M N Renzaho PhD); Department of Health Information Management (B Reshmi PhD), Department of Community Medicine (R S Shetty MD), Manipal Academy of Higher Education, Manipal, India; Network of Immunity in Infection, Malignancy and Autoimmunity, Universal Scientific Education and Research Network, Tehran, Iran (Prof N Rezaei PhD); Department of Surgery, University of Minnesota, Minneapolis, MN, USA (J Rickard MD); Department of Surgery, University Teaching Hospital of Kigali, Kigali, Rwanda (J Rickard); Department of Clinical Research, Federal University of Uberlândia, Uberlândia, Brazil (L Roever PhD); School of Medicine, Gonabad University of Medical Sciences, Gonabad, Iran (M Rostamian PhD); African Genome Center, Mohammed VI Polytechnic University (UM6P), Ben Guerir, Morocco (E Rubagotti PhD); Center for Research in Congenital Anomalies and Rare Diseases, ICESI University (Centro de Investigaciones en Anomalías Congénitas y Enfermedades Raras, Universidad Icesi), Cali, Colombia (E Rubagotti); Department of Internal Medicine, University of Botswana, Gaborone, Botswana (G M Rwegerera MD); Sharjah Institute for Medical Research (B Saddik PhD), University of Sharjah, Sharjah, United Arab Emirates; Applied Biomedical Research Center and Biotechnology Research Center, Mashhad University of Medical Sciences, Mashhad, Iran (A Sahebkar PhD); Department of Public Health, Madda Walabu University, Bale Robe, Ethiopia (B Sahiledengle MPH); Department of Entomology (A M Samy PhD), Department of Neurology (Prof A S Shalash PhD), Ain Shams University, Cairo, Egypt; Independent Consultant, Thiruvananthapuram, India (S Y Saraswathy PhD); Geriatric and Long Term Care Department, Hamad Medical Corporation, Doha, Qatar (B Sathian PhD); Faculty of Health & Social Sciences, Bournemouth University, Bournemouth, UK (B Sathian PhD); Department of Psychology (D C Schwebel PhD), School of Medicine (Prof J A Singh MD), University of Alabama at Birmingham, Birmingham, AL, USA; Public Health Division, An-Najah National University, Nablus, Palestine (A A Shaheen PhD); Department of Internal Medicine, Ziauddin University, Karachi, Pakistan (I Shahid MBBS); Independent Consultant, Karachi, Pakistan (M A Shaikh MD); School of Medicine, Alborz University of Medical Sciences, Karaj, Iran (M Shams-Beyranvand MSc); Symbiosis Medical College for Women, Symbiosis International University, Pune, India (M Shannawaz PhD); Centre for Medical Informatics, University of Edinburgh, Edinburgh, UK (Prof A Sheikh MD); National Institute of Infectious Diseases, Tokyo, Japan (M Shigematsu PhD); Department of Public Health Dentistry, Krishna Institute of Medical Sciences Deemed to be University, Karad, India (Prof K M Shivakumar PhD); School of Health, University of Technology Sydney, Sydney, NSW, Australia (S Siabani PhD); Department of Medicine, Dow University of Health Sciences, Karachi, Pakistan (T J Siddiqi MB); School of Public Health & Zoonoses, Guru Angad Dev Veterinary & Animal Sciences University, Ludhiana, India (B B Singh PhD); Medicine Service, US Department of Veterans Affairs, Birmingham, AL, USA (Prof J A Singh MD); Department of Midwifery, Dire Dawa University, Dire Dawa, Ethiopia (Y Sintayehu MSc); Department of Public Health, Arba Minch University, Arba Minch, Ethiopia (M B Sorrie MPH); Hull York Medical School, University of Hull, Hull, UK (I N Soyiri PhD); Division of Community Medicine, International Medical University, Kuala Lumpur, Malaysia (C T Sreeramareddy MD); Occupational and Environmental Medicine Department, University of Gothenburg, Gothenburg, Sweden (L Stockfelt PhD); National Institute of Epidemiology, Indian Council of Medical Research, Chennai, India (R Suliankatchi Abdulkader MD); Department of Medicine, University of Valencia, Valencia, Spain (Prof R Tabarés-Seisdedos PhD); Carlos III Health Institute, Biomedical Research Networking Center for Mental Health Network, Madrid, Spain (Prof R Tabarés-Seisdedos PhD); Cancer Control Center, Osaka International Cancer Institute, Osaka, Japan (T Tabuchi MD); Research Center for Molecular Medicine, Hamadan University of Medical Sciences, Hamadan, Iran (A Taherkhani PhD); Department of Public Health and Community Medicine, Central University of Kerala, Kasaragod, India (Prof K R Thankappan MD); Modestum, London, UK (M R Tovani-Palone PhD); Amity Institute of Biotechnology, Amity University Rajasthan, Jaipur, India (E Upadhyay PhD); Clinical Cancer Research Center, Milad General Hospital, Tehran, Iran (S Valadan Tahbaz PhD, S Yahyazadeh Jabbari MD); Department of Microbiology, Islamic Azad University, Tehran, Tehran, Iran (S Valadan Tahbaz PhD); Department of Nephrology, Christian Medical College and Hospital, Vellore, India (Prof S Varughese FRCP); Department of Medical and Surgical Sciences, University of Bologna, Bologna, Italy (Prof F S Violante MD); Occupational Health Unit, Sant'Orsola Malpighi Hospital, Bologna, Italy (Prof F S Violante); Faculty of Information Technology, HUTECH University, Ho Chi Minh City, Viet Nam (B Vo PhD); Foundation University Medical College, Foundation University Islamabad, Islamabad, Pakistan (Prof Y Waheed PhD); Demographic Change and Aging Research Area, Federal Institute for Population Research, Wiesbaden, Germany (A Werdecker PhD); Health Services Management Research Center, Kerman University of Medical Sciences, Kerman, Iran (V Yazdi-Feyzabadi PhD); Department of Health Management, Policy, and Economics, Kerman University of Medical Sciences, Kerman, Iran (V Yazdi-Feyzabadi); Department of Public Health, Wollega University, Nekemte, Ethiopia (M T Yilma MPH); Department of Neuropsychopharmacology, National Center of Neurology and Psychiatry, Kodaira, Japan (N Yonemoto PhD); Department of Public Health, Juntendo University, Tokyo, Japan (N Yonemoto PhD); Department of Health Policy and Management, Jackson State University, Jackson, MS, USA (Prof M Z Younis PhD); School of Business & Economics, University Putra Maylisia, Kuala Lumpur, Malaysia (Prof M Z Younis PhD); Department of Epidemiology and Biostatistics (Prof C Yu PhD), School of Medicine (Z Zhang PhD), Wuhan University, Wuhan, China; School of Public Health and Management, Hubei University of Medicine, Shiyan, China (Y Yu MS); Maternal and Child Health Division, International Centre for Diarrhoeal Disease Research, Bangladesh, Dhaka, Bangladesh (S Zaman MPH); School of Public Health, Hubei Province Key Laboratory of Occupational Hazard Identification and Control, Wuhan University of Science and Technology, Wuhan, China (Y Zhang PhD); School of Population and Public Health, University of British Columbia, Vancouver, BC, Canada (Prof M Brauer DSc).

**Declaration of interests**

R Ancuceanu reports consultancy or speakers’ fees from UCB, Sandoz, Abbvie, Zentiva, Teva, Laropharm, CEGEDIM, Angelini, B Braun, Biessen Pharma, Hofigal, AstraZeneca, and Stadam. M L Bell reports grants or contracts from the US Environmental Protection Agency, National Institutes of Health, High Tide Foundation, Yale Climate Change and Health Center, Robert Wood Johnson Foundation, Environmental Defense Fund, Health Effects Institute and the Wellcome Trust, all as payments to their institution; consulting fees from the Environmental Protection Agency as personal payments for membership on the Clean Air Scientific Advisory Committee; payment or honoraria for lectures, presentations, speakers bureaus, grant reviews, manuscript writing, external advisory committees, or educational events from Boston University, Korea University, the Organization of Teratology Information Specialists, the NIH, Health Canada, PAC-10, UK Research Institute, Harvard University, and the University of Montana; support for attending meetings or travel from Boston University, Harvard University, University of Illinois at Champaign, and the University of Texas; participation on a data safety monitoring board or advisory board with National Academies Panels and Committees; membership of Lancet Countdown, Fifth National Climate Assessment, Johns Hopkins University Deptartment of Environmental Health and Engineering Advisory Board, WHO Global Air Pollution and Health Technical Advisory group, and the US Environmental Protection Agency Clean Air Scientific Advisory Board. J M Castaldelli-Maia reports grants from the French National Institute for Cancer and Pfizer and consulting fees from L’Oreal for participation on international multidisciplinary scientific boards around skin conditions and mental wellness. A Deshpande reports consulting fees from Epidemiology Research & Methods. SMS Islam reports grants from the National Heart Foundation of Australia and from the Australian National Health and Medical Research Council. K Krishan reports non-financial support from UGC Centre of Advanced Study, CAS II, Department of Anthropology, Panjab University, Chandigarh, India. P W Mahasha reports leadership or fiduciary roles or membership in the Federation of Infectious Diseases Societies of Southern Africa, the EU-Africa PerMed Consortium, the South African Society for Biochemistry and Molecular, the International Society for Infectious Diseases, the Global Burden of Disease Collaborator Network, the South African Society of Microbiology, the COVID-19 Clinical Research Coalition, the Scholars Academic and Scientific Society, and the South African Council for Natural Scientific Professions. S Mohammed reports support from the Bill & Melinda Gates Foundation and a fellowship grant from the Alexander von Humboldt Foundation. S B Munro reports stock in Invitae and other financial or non-financial interests as an employee of Invitae. T Pilgrim reports grants or contracts from Biotronik, Boston Scientific, and Edwards Lifesciences as personal payments; participation on a data safety monitoring board or advisory board with Highlife SAS on the Clinical Event Adjudication Committee; and other financial and non-financial interests with Boston Scientific and Medtronic for proctoring. M J Postma reports stock or stock options in Health-Ecore (25%) and Pharmacoeconomics Advice Groningen (100%). A Radfar reports financial or non-financial support from Avicenna Medical and Clinical Research Institute. E Upadhyay reports published patents for “a system and method of reusable filters for anti-pollution mask” and “a system and method for electricity generation through crop stubble by using microbial fuel cells”, and filed patents for “a system for disposed personal protection equipment (PPE) into biofuel through pyrolysis and method” and “a novel herbal pharmaceutical aid for formulation of gel and method thereof” and is Joint Secretary of the Indian Meteorological Society, (Jaipur Chapter).
