# Supplementary material for: Mapping development and health effects of cooking with solid fuels in low-income and middle-income countries, 2000–18: a geospatial modelling study
Source: Lancet Glob Health. 2022 Sep 13;10(10):e1395–411. doi: 10.1016/S2214-109X(22)00332-1 (PMC9638039; doi:10.1016/S2214-109X(22)00332-1)
Supplement: Supplementary appendix [file mmc1.pdf]

THE LANCET
Global Health

Supplementary appendix

This appendix formed part of the original submission and has been peer reviewed. We post it as supplied by the authors.

Supplement to: Local Burden of Disease Household Air Pollution Collaborators. Mapping development and health effects of cooking with solid fuels in low-income and middle-income countries, 2000–18: a geospatial modelling study. *Lancet Glob Health* 2022; **10:** e1395–411.

Supplementary Information: HAP Stage II (2000–2018)

Contents

0.0 Guidelines for Accurate and Transparent Health Estimates Reporting (GATHER) Compliance 2
1.0 Data 2
1.1 Known issues 2
1.1.1 Countries with no data 2
1.2 Model geographies 2
1.3 Data inclusion criteria 3
1.4 Summary of included data sources 3
1.5 Processing data for modelling 3
1.6 Processing areal data for geostatistical modelling 3
1.7 Data validity 4
2.0 Covariates 4
2.1 Covariate selection 4
2.2 Modelling covariates via stacked generalisation 4
3.0 Geostatistical model 5
3.1 Model parameters 5
3.2 Metrics of predictive validity 6
3.3 Propagating uncertainty 6
3.4 $PM_{2.5}$ exposure and proportional PAFs 6
3.5 $PM_{2.5}$ dose–response 7
4.0 Supplementary figures 11

0.0 Guidelines for Accurate and Transparent Health Estimates Reporting (GATHER) Compliance

Supplementary Table 1 provides the Guidelines for Accurate and Transparent Health Estimates Reporting (GATHER) compliance checklist.

1.0 Data

1.1 Known issues

1.1.1 Countries with no data

This analysis does not produce estimates in countries where data did not meet our inclusion criteria due to data availability or quality of cooking fuel use data. We have masked all estimates in countries for which we had no data that we could geolocate below the national level. For cooking fuel use, this included Cabo Verde, Cuba, French Guyana, Equatorial Guinea, Malaysia, Venezuela, and Western Sahara. If data become available from any of these countries, we will include them in the models and share the results.

1.2 Model geographies

This analysis selected 98 countries based on their Socio-demographic Index (SDI).[1] The SDI is a measure of education, fertility, and income. Countries included were in the middle, lower-middle, or low SDI quintiles, with certain exceptions. China and Libya were included despite higher-middle SDI status to create better geographical continuity. Despite the requisite SDI quintiles for inclusion, we excluded the countries of Cabo Verde, Cuba, French Guyana, Equatorial Guinea, Malaysia, Venezuela, and Western Sahara as they had no relevant data available. We also excluded island nations with fewer than 1 million inhabitants, which were the nations of Fiji, Solomon Islands, Maldives, Vanuatu, Samoa, Saint Lucia, Kiribati, St. Vincent and the Grenadines, Grenada, Micronesia, Tonga, Seychelles, Dominica, Marshall Islands, and American Samoa. These countries were typically data-scarce and did not have sufficient geographical continuity for a geospatial analytical approach to be advantageous over a national one.

We subdivided the spatial modelling domain into 18 regions, consisting of groupings of countries. The regions were created in consideration of geographical contiguity and epidemiological similarity between the constituent countries. In the case of China, Mongolia, Nigeria, South Africa, and the combined area of Eritrea, Djibouti, and Yemen, the data exhibited distinct spatial and temporal trends in comparison of their neighbouring countries. Therefore, the aforementioned countries were modelled individually. By creating modelling regions, we were able to account for potential non-stationarity in the relationships between covariates and the outcome across the modelling domain as each region would be modelled independently. Furthermore, subdividing the 98 countries into 18 modelling regions allowed for computational feasibility.

Computational limitations meant that we were unable to account for potential non-stationarity within countries/regions, as we assumed an isotropic and stationary Matérn function (see Section 3.1 for more detail) to model spatial covariance at that level. Assuming stationarity likely introduces bias to the results but would be computationally infeasible to model at the spatial scale required for the scope of

this analysis. Community-level drivers have been observed to account for within-country heterogeneity in solid fuel use, and the spatial correlation introduced by these socioeconomic effects is unlikely to be fully captured by an isotropic process.[1] Comparison of stationary and non-stationary models for other health risk data has previously demonstrated that non-stationary models can increase prediction accuracy, while estimating roughly similar spatial trends.[2] An inherent irony is that modelling non-stationarity becomes more important as the size of the modelling area increases, while being increasingly computationally intensive on the same axis. The environmental indicators we used as geospatial covariates, such as land use and travel time to the nearest settlement, should explain some of this variation,[3,4] but additional anisotropic effects may exist as cooking behaviours are understood to correlate with nuanced social patterns.[5,6] We expect that relaxing the assumption of isotropy for modelling cooking fuel use would reveal additional within-country heterogeneity and demonstrate even higher subnational inequalities, and we think this is an important area for future research as additional data become accessible and as the computational power available to researchers continues to increase. Given that most of our results are derived from the aggregation of the geospatial model to administrative units for comparison, an additional area of further investigation could be the use of areal modelling approaches such as locally adaptive conditional auto-regressive (CAR) models, which could relax the assumption of stationarity and potentially reveal discontinuities in the spatial surface that may be smoothed over by our geostatistical model.

1.3 Data inclusion criteria

Sources were only included for modelling if they were representative of the entire population during the time period and across the geographical area of measurement. Furthermore, certain sources were excluded if the associated estimates seemed implausible based on expert review of estimates and comparison with other sources in the same country and time period.

1.4 Summary of included data sources

The household surveys used in modelling solid fuel use are listed in Table 4. Each source is listed with the number of households represented, country, and year. Additionally, the tables provide the number of georeferenced points or spatial areas represented by each source as well as a unique identifier (GHDx ID) which can be used to reference the source via the Global Health Data Exchange (GHDx).[2] 663 household surveys from 98 countries were included in the solid fuel use model.

1.5 Processing data for modelling

Data corresponding to each georeferenced point were summarised as survey weighted means. For data without corresponding GPS coordinates, data were summarised across the smallest spatial area the data were representative over to produce areal estimates. Household sizes and survey weights were used to produce weighted means of access representative at the individual level. The household sizes and survey weights were further used in a Kish approximation[4] of an effective sample size for each mean to account for the complex survey design of most of the data used.

1.6 Processing areal data for geostatistical modelling

For areal data, 10,000 locations were randomly sampled from the area using population values from WorldPop[5] raster to weight the sampled points. K-means clustering was performed over these points to generate integration points (1 point per 1000 WorldPop grid cells encompassing the area) to be used in the modelling. Weights were assigned to integration points as the proportion of the original 10,000 points that entered the k-means cluster. Each of these integration points was assigned the areal mean. The integration points were included in the input dataset for further modelling. In this manner, the

spatial variation of covariates could be leveraged and areal data could be incorporated into the geostatistical model. The above resampling method used is consistent with the resampling conducted in the previously published geospatial modelling of child growth failure.

1.7 Data validity

Following the vetting of indicator string matching, all surveys were systematically reviewed for data quality. Country-specific diagnostic plots were produced for cooking fuel. The estimate for each indicator from each data source was compared to the other available data sources for that country for similar years. If the data source's estimate was considered implausibly different from other sources, then the source was excluded. Each country-specific diagnostic plot was independently reviewed, considering country and time trends, as well as whether a survey was nationally and/or subnationally representative.

2.0 Covariates

2.1 Covariate selection

The first stage of modelling used 27 covariates, composed of various environmental and social indicators. Each covariate was formatted at a 5 x 5 km resolution from 2000 to 2018. They included travel time to nearest settlement, aridity, diurnal temperature range, frost day frequency, potential evapotranspiration, average daily mean temperature, dependency ratio of dependents to working age adults, distance from rivers or lakes ≥ 50 km^2, nighttime lights, elevation, agricultural land, enhanced vegetation index, fertility, urban or rural, nutrient yield, irrigation, urban proportion of location, average land surface temperature, precipitation, normalised difference vegetation index, tassled cap brightness, tassled cap wetness, and population. For covariates which were continuous variables, we calculated rolling means using a 5-year window and used the results for modelling. This was conducted to stabilise any shocks in the time-trends of the covariates, aiding in stabilising the time-series predicted from modelling.

In the first stage, for each region, three types of models were fit to the data: generalised additive models, boosted regression trees, and lasso regression. In doing so, we attempted to account for the varying and potentially non-linear relationship between each of the covariates and the outcome indicator. Each of the 23 covariates was used in the boosted regression tree and lasso regression model. To prevent issues with collinearity and optimise performance, covariate selection was performed for the generalised additive model as follows:

Each covariate was regressed against all other covariates in order to identify if a covariate's variance can be accounted for via a linear combination of the other covariates. A variance inflation factor (VIF) was calculated for each such regression. The covariate corresponding with the independent variable in the regression with highest VIF was eliminated. This procedure was iteratively repeated until all associated VIFs were under 5.

2.2 Modelling covariates via stacked generalisation

As noted above, for each region, three models were fit to the data: generalised additive models, boosted regression trees, and lasso regression. Each model was fit using five-fold cross validation (out-of-sample) as well as on the entire dataset at once (in-sample). Out of sample predictions were generated from each model and logit transformed for subsequent use as covariates in the geostatistical model. The logit transformation was conducted to ensure the covariate and the outcome solid fuel use

sanitation indicator data would be in the same functional space. The in-sample predictions from these "sub-models" were used as covariates when generating predictions using the fitted relationships from the geostatistical model.

3.0 Geostatistical model

3.1 Model parameters

$$C_d | p_{i(d),} N_d \sim Binomial(p_{i(d)}, N_d) \; \forall \; obs. clusters \; d$$

$$logit(p_{i,t}) = \beta_0 + X_{i,\beta} + \varepsilon_{c(i)} + \varepsilon_{n(i)} + \varepsilon_i + Z_i$$

$$\sum\nolimits_{h=1}^{3} \beta_h = 1$$

$$\varepsilon_c \overset{iid}{\sim} N(0, {\gamma_c}^2)$$

$$\varepsilon_n \overset{iid}{\sim} N(0, {\gamma_n}^2)$$

$$\varepsilon_i \overset{iid}{\sim} N(0, \sigma^2)$$

$$Z \sim GP\left(0, \sum\nolimits^{space}\right)$$

$$\sum\nolimits^{space} = \frac{\omega^2}{\Gamma(v)2^{v-1}} * (kD)^v * K_v(kD)$$

The coefficients $X_{i,\beta}$ for the three stacked sub-models represent their respective predictive weighting in the mean logit link, while the joint error term, Z_i, accounts for any residual spatiotemporal autocorrelation. These residuals, Z_i, are modelled as a three-dimensional space-time Gaussian process (GP) which was centred at zero. The covariance matrix for this GP was constructed from a Kronecker product of spatial and temporal covariance. The spatial covariance, Σspace, is modelled based on a stationary and isotropic Matérn function. For the Matérn function, Γ is the gamma function, Kv is the modified Bessel function of order v > 0, κ > 0 is a scaling parameter, D represents the distance (Euclidean), and ω2 is the marginal variance. We defined the scaling parameter, κ, as $k = \sqrt{8v}/_{\delta}$ where δ is a range parameter (reflective of the distance where the covariance function is approaching 0.1) and v is a scaling constant, which is set to 2. This scaling constant, v, has been documented in other analyses to be difficult to fit reliably from data and is generally recommended to be fixed at the aforementioned value of 2.[7,8]

Our implementation of INLA using the R-INLA software relies on a Gaussian approximation of the full conditional distribution of latent variables, and uses the empirical Bayes approximation for the

hyperparameters. We have tried the full hyperparameter grid integration and CCD integration in various settings and have found our models to be nearly indistinguishable. For the sake of computing resource efficiency (with which we always operate at the margins), we have proceeded with using the empirical Bayes procedure. In a very similar setting with malaria household survey data, other authors compared the INLA results directly with results from Hamiltonian Markov Chain Monte Carlo and found nearly identical results between the two fits.[9,10]

3.2 Metrics of predictive validity

We assessed model performance based on a five-fold out-of-sample cross-validation framework with spatial stratification. Each fold was derived using a modified bi-tree algorithm[11] in order to aggregate datapoints spatially. The algorithm generates recursive partitions in two-dimensional space, alternating horizontal and vertical divisions on the median values of weighted sample sizes of the data until each spatial partition contains data of similar sample size. Constraints on the depth of the recursive partition are derived from the target sample size in each partition, as well as the minimum number of clusters or pseudo-clusters allowed for a given partition. For this analysis, a minimum sample size of 500 was used. We allocated these spatial partitions to five different folds for the cross-validation analysis. Each of these five folds was then excluded in turn while fitting the entire model five times. The withheld data were then compared with model predictions in order to generate the OOS metrics, including the mean error (bias), root-mean-squared-error (RMSE), correlation, and the 95% coverage rate of the predicted intervals (proportion of observed data that fall within the predicted 95% credible intervals). Each metric was produced by aggregating the data and predictions up to different administrative levels (levels 0, 1, 2 as defined by the Database of Global Administrative Areas [GADM]), in order to assess performance at different levels of spatial granularity. These metrics are calculated across all modelling regions and summarised in Supplementary Tables 8-10 and Figures 21a-22.

3.3 Propagating uncertainty

All estimates were generated by taking 1,000 draws of all model parameters from the approximated joint posterior distribution using the inla.posterior.sample() function in R-INLA[12,13], yielding 1000 candidate maps from which to summarise the grid-cell- and aggregated-level statistics. For estimates at the grid-cell level, these draws were used directly to generate estimates and uncertainty. Aggregated estimates, in which grid-cell-level estimates were summarised to administrative boundaries, were generated by creating population-weighted averages for each administrative boundary, for each draw. 95% uncertainty intervals around the mean of our estimates were generated.

3.4 $PM_{2.5}$ exposure and proportional PAFs

To estimate the increased health risk from cooking with solid fuels using a dose-specific risk function, we estimated the level of exposure to $PM_{2.5}$ expected for households using solid fuels to cook. WHO's Global Household Air Pollution (HAP) Measurements database contains 196 studies with measurements

from 43 countries of various pollution metrics in households using solid fuel for cooking.[14] From this database, a model was constructed based on all measurements of $PM_{2.5}$ using indoor or personal monitors. The final dataset included 336 estimates from 75 studies in 43 unique locations. 274 estimates were in households using solid fuels, 47 in households only using clean (gas or electricity) fuels, and 15 in households using a mixture of solid and clean fuels. Before modelling, each datapoint was converted from total particulates to the excess particulate matter in households using solid fuel by subtracting off the predicted ambient $PM_{2.5}$ value in the study location and year based on the GBD 2017 $PM_{2.5}$ exposure model.[15] A model constructed from this database was used as part of the GBD 2019 study to predict the expected incremental $PM_{2.5}$ for every modelled location.[16]

The excess $PM_{2.5}$ exposure that was generated by households primarily using solid fuels for cooking was combined for each location with an updated model from GBD 2019 of geospatially resolved ambient $PM_{2.5,}$ calculating the total $PM_{2.5}$ exposure for every modelled location. The ratio of excess household particulates to ambient particulates was estimated as part of this process. To account for overlap in ambient and household particulate exposures in countries with a high dual burden and prevent double-counting, we utilised a proportional PAF approach that was developed for GBD 2017.[15] This method relies on using published $PM_{2.5}$ dose–response curves (see appendix, section 3.5) applied to estimates of the total $PM_{2.5}$ exposure in a given location to estimate the PAF for total $PM_{2.5,}$ and then splitting it proportionally based on the previously estimated ambient:household $PM_{2.5}$ ratio (see appendix, figure 2b). This method assumes that it is unknown which area of the dose–response curve each source is contributing to, and therefore they must be considered equivalently as opposed to being subtracted from a particular slope of risk increase. Our approach is also dependent on an assumption of particle equitoxicity, and there is evidence that bias may be introduced by this method if there are fundamental differences in the chemical composition of particles generated by household and ambient sources of pollution.[17] Upon the availability of adequate data from which to develop more granular risk–response curves that provide source-specific distinctions, this assumption should be revisited.

3.5 $PM_{2.5}$ dose–response

To estimate the relative risk of exposure to ambient and household particulate matter ($PM_{2.5}$) air pollution we leveraged a cause-specific risk curve for acute lower respiratory infection, which had been produced as part of the GBD 2019 study.[16] To generate dose–response curves as a function of $PM_{2.5}$, splines were fit to relative risk and $PM_{2.5}$ exposure estimates from studies of ambient $PM_{2.5}$, household solid fuel use, and secondhand smoking using an open-source Bayesian, regularised, trimmed, meta-regression software (MR-BRT). With the flexibility of splines in MR-BRT and the addition of recent studies from high exposure settings,[18–22] the approach utilised for GBD 2019 no longer included studies of active smoking data in the risk curves. Specifically, for each of the risk–outcome pairs, various model settings and priors were tested in order to fit splines specified for the relationship as:

log((MRBRT(X))/MRBRT(X_CF))~log(Published Effect Size)

where X and XCF represent the range of exposure characterised by the effect size. The final models included third-order splines with two interior knots and a constraint on the right-most segment forcing a linear fit. GBD 2019 used an ensemble approach to knot placement, where 100 different models were run with randomly placed knots and then combined by weighting based on a measure of fit that penalises excessive changes in the third derivative of the curve. Knots were free to be placed anywhere within the 5th and 95th percentile of the data, as long as a minimum width of 10% of that domain exists between them. The functions were shaped constrained, such that the curves were monotonically increasing and concave downwards, the most biologically plausible shape for $PM_{2.5}$ risks. On the nonlinear segments, a Gaussian prior was specified on the third derivative of mean 0 and variance 0.01 to prevent over-fitting; on the linear segment, a stronger prior of mean 0 and variance 1e-6 was used to ensure that the risk curves did not continue to increase beyond the range of the data. Additional information about how this approach was specified is available in the GBD 2019 risk factors summary manuscript.[16]

References

1. Shupler, M. *et al.* Household, community, sub-national and country-level predictors of primary cooking fuel switching in nine countries from the PURE study. *Environ. Res. Lett.* **14**, 085006 (2019).
2. Gosoniu, L., Vounatsou, P., Sogoba, N. & Smith, T. Bayesian modelling of geostatistical malaria risk data. *Geospatial Health* **1**, 127–139 (2006).
3. Beauchamp, M., de Fouquet, C. & Malherbe, L. Dealing with non-stationarity through explanatory variables in kriging-based air quality maps. *Spat. Stat.* **22**, 18–46 (2017).
4. Bertazzon, S., Johnson, M., Eccles, K. & Kaplan, G. G. Accounting for spatial effects in land use regression for urban air pollution modeling. *Spat. Spatio-Temporal Epidemiol.* **14–15**, 9–21 (2015).
5. Mani, S., Jain, A., Tripathi, S. & Gould, C. F. The drivers of sustained use of liquified petroleum gas in India. *Nat. Energy* **5**, 450–457 (2020).

6. Pye, A. *et al.* Drivers of the Adoption and Exclusive Use of Clean Fuel for Cooking in Sub-Saharan Africa: Learnings and Policy Considerations from Cameroon. *Int. J. Environ. Res. Public. Health* **17**, 5874 (2020).
7. Kinyoki, D. K. *et al.* Mapping child growth failure across low- and middle-income countries. *Nature* **577**, 231–234 (2020).
8. Lindgren, F. & Rue, H. Bayesian Spatial Modelling with R-INLA. *J. Stat. Softw.* **63**, 1–25 (2015).
9. Patil, A. P., Gething, P. W., Piel, F. B. & Hay, S. I. Bayesian geostatistics in health cartography: the perspective of malaria. *Trends Parasitol.* **27**, 246–253 (2011).
10. Lindgren, F., Rue, H. & Lindström, J. An explicit link between Gaussian fields and Gaussian Markov random fields: the stochastic partial differential equation approach. *J. R. Stat. Soc. Ser. B Stat. Methodol.* **73**, 423–498 (2011).
11. Roberts, D. R. *et al.* Cross-validation strategies for data with temporal, spatial, hierarchical, or phylogenetic structure. *Ecography* **40**, 913–929 (2017).
12. Gómez-Rubio, V. *Chapter 2 The Integrated Nested Laplace Approximation | Bayesian inference with INLA*.
13. Fuglstad, G.-A. & Beguin, J. Environmental mapping using Bayesian spatial modelling (INLA/SPDE): A reply to Huang et al. (2017). *Sci. Total Environ.* **624**, 596–598 (2018).
14. Shupler, M. *et al.* Global household air pollution database: Kitchen concentrations and personal exposures of particulate matter and carbon monoxide. *Data Brief* **21**, 1292–1295 (2018).
15. Stanaway, J. D. *et al.* Global, regional, and national comparative risk assessment of 84 behavioural, environmental and occupational, and metabolic risks or clusters of risks for 195 countries and territories, 1990–2017: a systematic analysis for the Global Burden of Disease Study 2017. *The Lancet* **392**, 1923–1994 (2018).

16. Murray, C. J. L. *et al.* Global burden of 87 risk factors in 204 countries and territories, 1990–2019: a systematic analysis for the Global Burden of Disease Study 2019. *The Lancet* **396**, 1223–1249 (2020).

17. Goldstein, A. H., Nazaroff, W. W., Weschler, C. J. & Williams, J. How Do Indoor Environments Affect Air Pollution Exposure? *Environ. Sci. Technol.* **55**, 100–108 (2021).

18. Yin, P. *et al.* Long-term Fine Particulate Matter Exposure and Nonaccidental and Cause-specific Mortality in a Large National Cohort of Chinese Men. *Environ. Health Perspect.* **125**, 117002 (2017).

19. Li, T. *et al.* All-cause mortality risk associated with long-term exposure to ambient PM2·5 in China: a cohort study. *Lancet Public Health* **3**, e470–e477 (2018).

20. Yang, Y. *et al.* Long term exposure to air pollution and mortality in an elderly cohort in Hong Kong. *Environ. Int.* **117**, 99–106 (2018).

21. Yusuf, S. *et al.* Modifiable risk factors, cardiovascular disease, and mortality in 155 722 individuals from 21 high-income, middle-income, and low-income countries (PURE): a prospective cohort study. *Lancet Lond. Engl.* **395**, 795–808 (2020).

22. Hystad, P. *et al.* Health Effects of Household Solid Fuel Use: Findings from 11 Countries within the Prospective Urban and Rural Epidemiology Study. *Environ. Health Perspect.* **127**, 57003 (2019).

23. Murray, C. J. L., GBD 2019 Risk Factors Collaborators & S.S., Lim. Global burden of 87 risk factors in 204 countries and territories, 1990–2019: a systematic analysis for the Global Burden of Disease Study 2019. *The Lancet* **396**, (2020).

24. Shupler, M. *et al.* Global estimation of exposure to fine particulate matter (PM2.5) from household air pollution. *Environ. Int.* **120**, 354–363 (2018).

25. Reiner, R. C. *et al.* Identifying residual hotspots and mapping lower respiratory infection morbidity and mortality in African children from 2000 to 2017. *Nat. Microbiol.* **4**, 2310–2318 (2019).

4.0 Supplementary figures

Supplementary Figure 1: Data processing flowchart 12
Supplementary Figure 2a/b: Geospatial modelling/Post-estimation flowcharts 14
Supplementary Figure 3: Map of modelling regions 18
Supplementary Figure 4a-e: Solid fuel use data availability by type and country, 2000-2018 19
Supplementary Figure 5: Geospatial covariates 24
Supplementary Figure 6: Finite elements mesh 26
Supplementary Figure 7: Posterior means and 95% uncertainty intervals for solid fuel use prevalence at the first administrative level, 2018 27
Supplementary Figure 8: Posterior means and 95% uncertainty intervals for TAP dose at the second administrative level, 2018 29
Supplementary Figure 9: Posterior means and 95% uncertainty intervals for TAP dose at the first administrative level, 2018 31
Supplementary Figure 10: Posterior means and 95% uncertainty intervals for HAP share at the second administrative level, 2018 32
Supplementary Figure 11: Posterior means and 95% uncertainty intervals for HAP share at the first administrative level, 2018 34
Supplementary Figure 12: Posterior means and 95% uncertainty intervals for LRI mortality counts due to TAP at the second administrative level, 2018 35
Supplementary Figure 13: Posterior means and 95% uncertainty intervals for LRI mortality counts due to TAP at the first administrative level, 2018 37
Supplementary Figure 14: Posterior means and 95% uncertainty intervals for LRI mortality rates due to TAP at the second administrative level, 2018 38
Supplementary Figure 15: Posterior means and 95% uncertainty intervals for LRI mortality rates due to TAP at the first administrative level, 2018 40
Supplementary Figure 16: HAP annualized rate of change 2000-2018 at the second administrative level 41
Supplementary Figure 17a-u: Population and attributable under-5 LRI mortalities, distributed as a function of PM2.5 by region. .... **Error! Bookmark not defined.**
Supplementary Figure 18a-v: Air pollution risk transition (2000–2018) by region. ..... **Error! Bookmark not defined.**
Supplementary Figure 19a-c: SFU in-sample by aggregation level.......... **Error! Bookmark not defined.**
Supplementary Figure 20a-c: SFU out-of-sample by aggregation level.......... **Error! Bookmark not defined.**

Supplementary Figure 1: Data processing flowchart

Source identification and inclusion and exclusion criteria are outlined above. This illustrates the data pipeline starting with data intake to finalised data for modelling. Sources that met initial cooking fuel, household sampling, country, and time frame criteria were extracted and standardised. Each survey's set of clusters were matched to the finest geography possible. Surveys were dropped due to implausible or insufficient cooking fuel and household sampling data. All remaining data were then collapsed by survey, year, country, and geography. The collapse process filters out high-income countries, data before 1998–2000, and the absence of survey weights. Observations and survey clusters were also dropped if there was excessive (>20%) missing data in cooking fuel or survey weights as well as if geography information was not able to be matched. Usable survey reports were manually extracted at the finest aggregated level available and appended onto collapsed data, where all polygon data were then resampled to points. All surveys underwent final vetting reviews with diagnostic plots, where final exclusions took place due to implausible trends. The resulting cleaned data were used as input data ready for modelling.

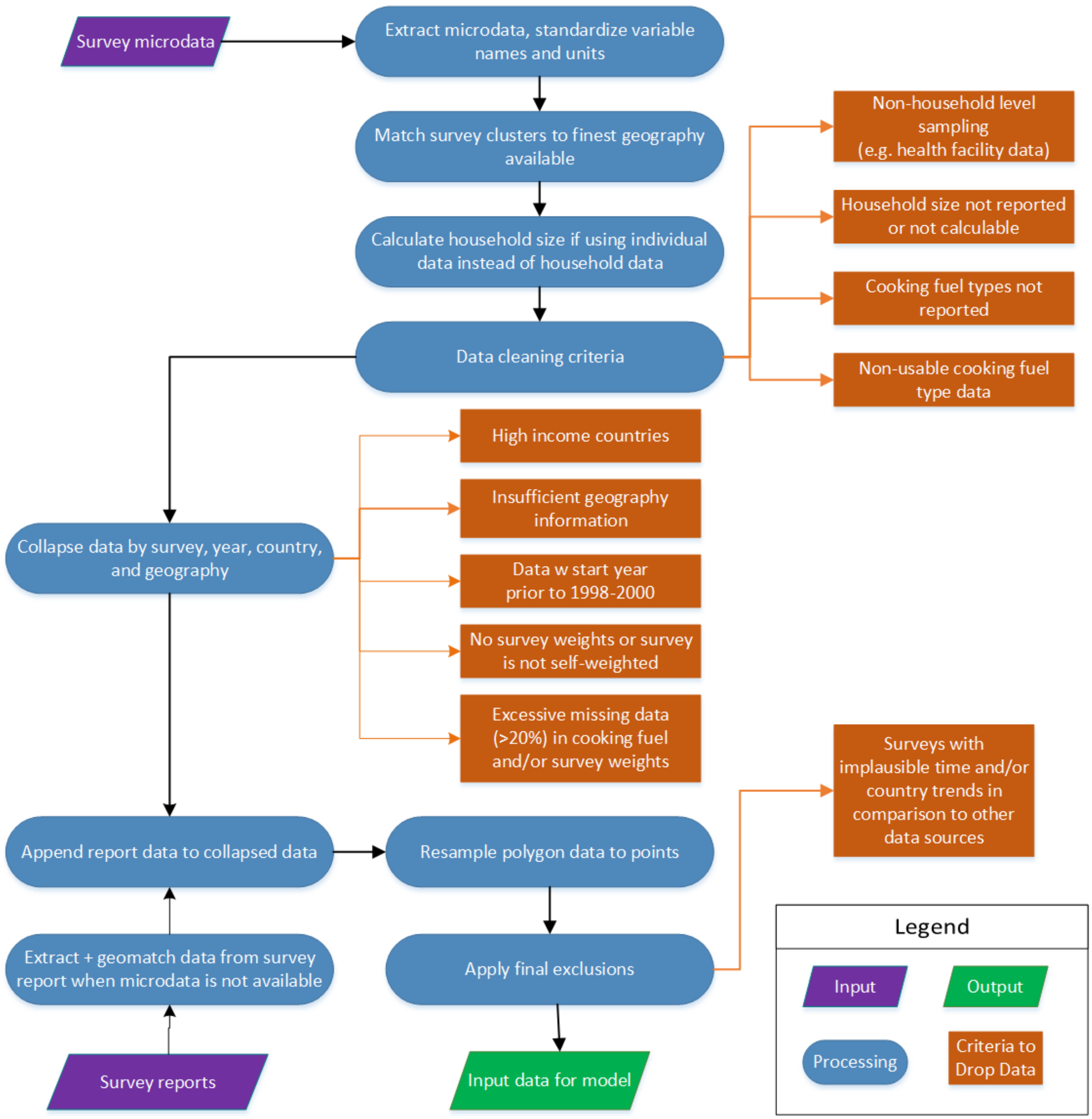
Survey microdata
Extract microdata, standardize variable names and units
Match survey clusters to finest geography available
Calculate household size if using individual data instead of household data
Data cleaning criteria
Non-household level sampling (e.g. health facility data)
Household size not reported or not calculable
Cooking fuel types not reported
Non-usable cooking fuel type data
High income countries
Insufficient geography information
Data w start year prior to 1998-2000
No survey weights or survey is not self-weighted
Excessive missing data (>20%) in cooking fuel and/or survey weights
Collapse data by survey, year, country, and geography
Surveys with implausible time and/or country trends in comparison to other data sources
Append report data to collapsed data
Resample polygon data to points
Extract + geomatch data from survey report when microdata is not available
Apply final exclusions
Survey reports
Input data for model
Legend
Input
Output
Processing
Criteria to Drop Data

Supplementary Figure 2a: Geospatial modelling flowchart

The geospatial modelling process consists of three sections. First (in blue), all available survey data are compiled if they could be referenced to coordinates/points or polygon units from survey clusters as well as calculate the use of cooking fuel types at their respective levels. Data matched to polygons are resampled into pseudo points using a k-means clustering algorithm. Covariates are subsequently merged to the points and pseudo points via a spatial join. Second (in green), point data and their associated covariates along with a stacked generalisation ensemble model is used. The children models, boosted regression trees, generalised additive models, and elastic net regression were fit using a 5-fold cross-validation process. The cross-validated predictions from each model then serve as the covariate values for the main/parent model (spatiotemporal GPR) model. The predictions from when the child models were fit on all the data (rather than 4/5ths implied by the cross-validation) are then used to create posterior predictions of solid fuel use prevalence in a 5x5 km grid for the years 2000–2018. Finally (in orange), our estimates are aggregated to the first administrative-level units.

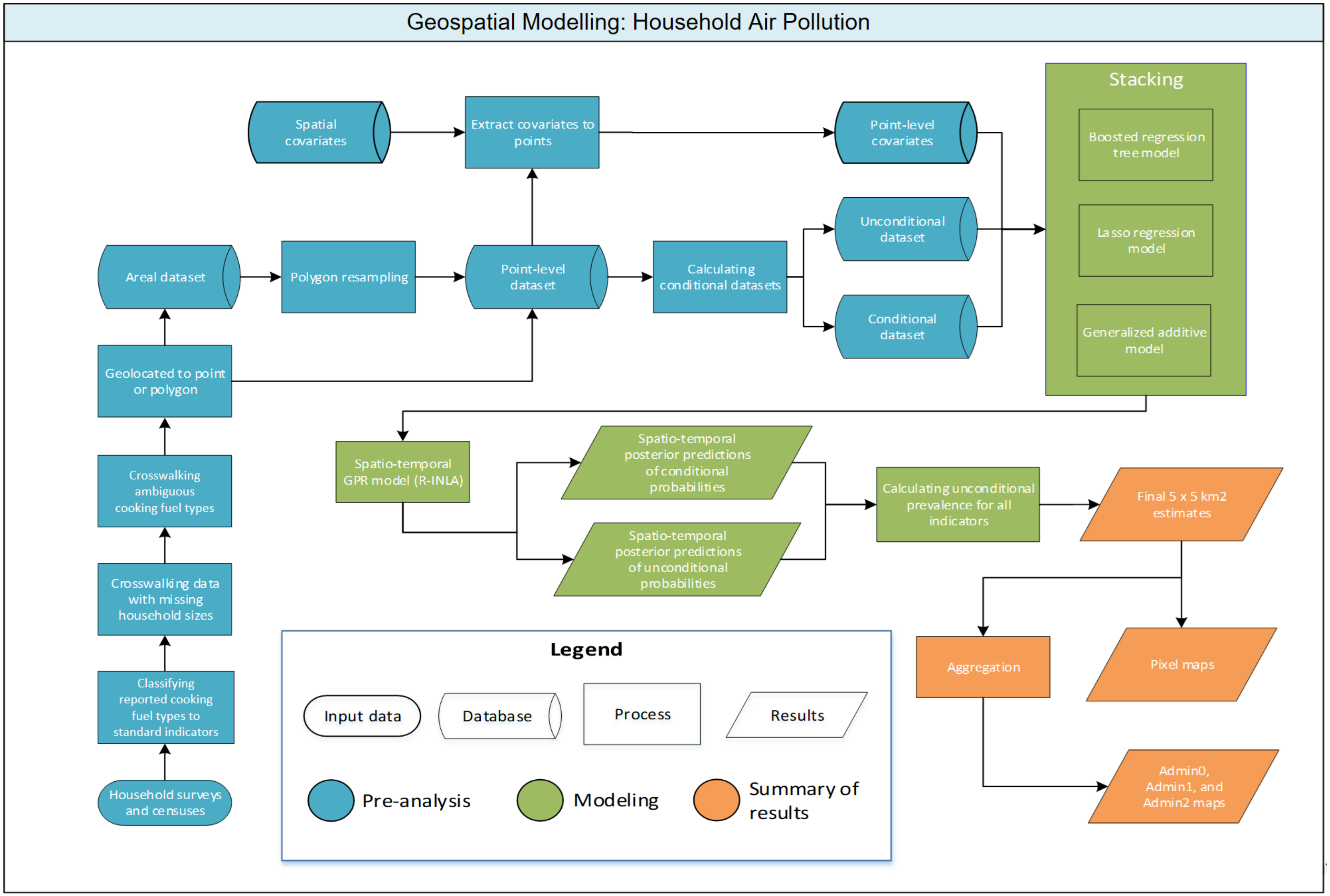
Geospatial Modelling: Household Air Pollution
Stacking
Boosted regression tree model
Lasso regression model
Generalized additive model
Spatial covariates
Extract covariates to points
Point-level covariates
Unconditional dataset
Areal dataset
Polygon resampling
Point-level dataset
Calculating conditional datasets
Conditional dataset
Geolocated to point or polygon
Crosswalking ambiguous cooking fuel types
Crosswalking data with missing household sizes
Classifying reported cooking fuel types to standard indicators
Household surveys and censuses
Spatio-temporal GPR model (R-INLA)
Spatio-temporal posterior predictions of conditional probabilities
Spatio-temporal posterior predictions of unconditional probabilities
Calculating unconditional prevalence for all indicators
Final 5 x 5 km2 estimates
Aggregation
Pixel maps
Admin0, Admin1, and Admin2 maps
Legend
Input data
Database
Process
Results
Pre-analysis
Modeling
Summary of results

Supplementary Figure 2b: Post-estimation flowchart

We combined the model output with geospatial estimates of ambient $PM_{2.5}$ exposure from GBD 2019[23] in order to calculate personal exposure to total $PM_{2.5}$ pollution (TAP) concentration of $PM_{2.5}$ as the sum of the AAP and HAP concentration in each 5 x 5-km grid cell i (pixel). Estimates of the expected incremental $PM_{2.5}$ concentration (see *Definitions)* generated in a household using solid fuels ($HPM_{2.5}$) for a given country (c) and year (t) from GBD 2019 were used to calculate the HAP concentration for the exposed population.[24] The per capita annual average ambient $PM_{2.5}$ estimate from GBD 2019 ($APM_{2.5}$) was summed with the HAP concentration to provide the TAP concentration. The fraction of TAP contributed by HAP in each pixel was estimated to provide the HAP share ($AAP{:}\,HAP\ ratio$). Finally, the per capita TAP concentration in each pixel was used as an input to the GBD 2019 risk (IER)[23] curve for LRI in order to estimate a relative risk (RR) and PAF for every $PM_{2.5}$-associated outcome (o) in each pixel. The PAF for LRI was combined with pixel-level estimates[25] of under-5 LRI mortality counts $\left(N_{i,c,t,o}\right)$ in order to estimate the count $(TAP\ N)$ and rate of LRI deaths that were attributable to TAP and – using the estimated HAP share – HAP $\left(HAP\ Deaths\ N_{i,c,t,o}\right)$ versus AAP in each district.

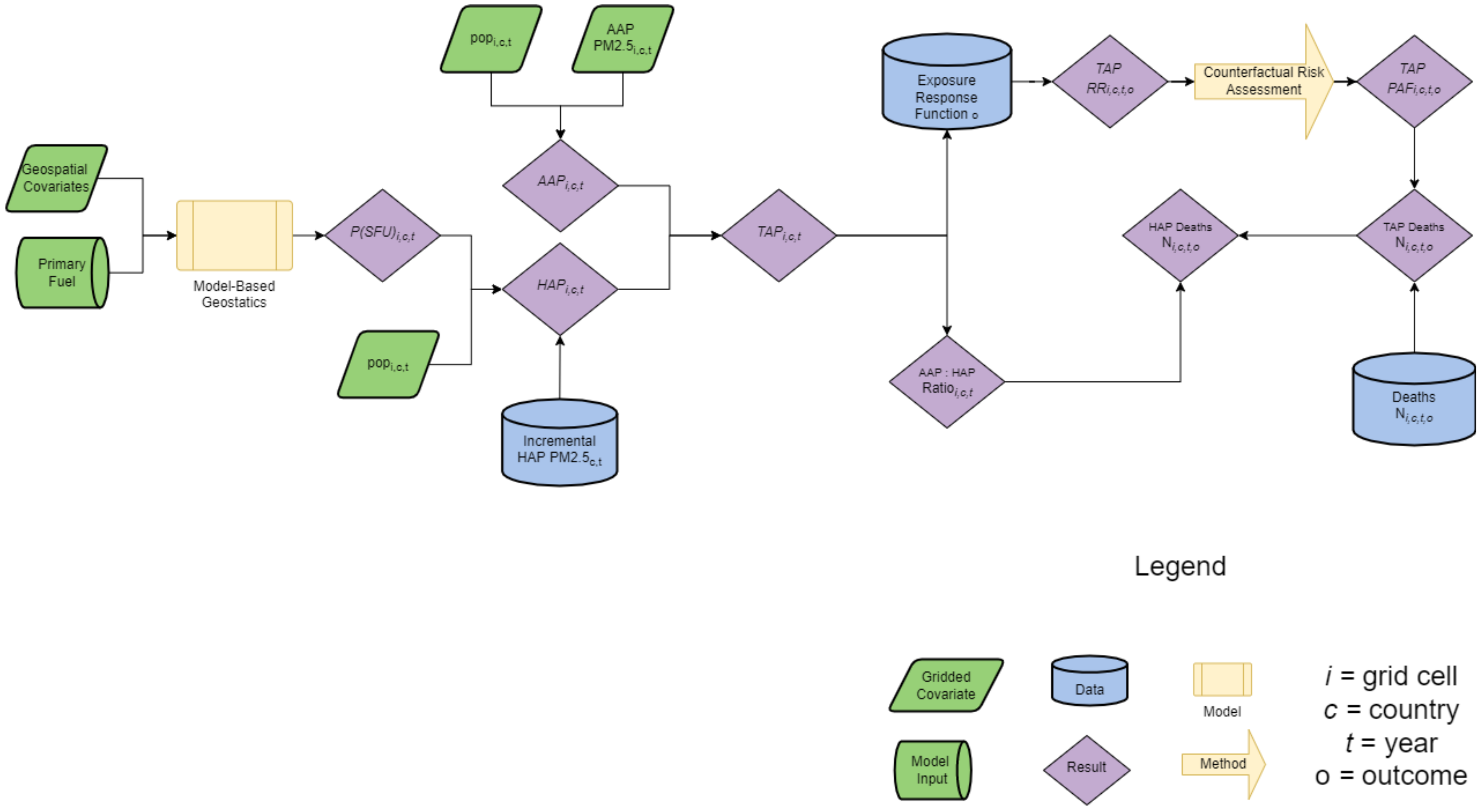
pop$_{i,c,t}$
AAP PM2.5$_{i,c,t}$
Geospatial Covariates
Primary Fuel
Model-Based Geostatics
$P(SFU)_{i,c,t}$
$AAP_{i,c,t}$
$HAP_{i,c,t}$
pop$_{i,c,t}$
Incremental HAP PM2.5$_{c,t}$
$TAP_{i,c,t}$
Exposure Response Function $_o$
TAP $RR_{i,c,t,o}$
Counterfactual Risk Assessment
TAP $PAF_{i,c,t,o}$
AAP : HAP Ratio$_{i,c,t}$
HAP Deaths $N_{i,c,t,o}$
TAP Deaths $N_{i,c,t,o}$
Deaths $N_{i,c,t,o}$
Legend
Gridded Covariate
Data
Model
Model Input
Result
Method
i = grid cell
c = country
t = year
o = outcome

Supplementary Figure 3: Map of modelling regions

We stratified our data and analyses into 21 contiguous regions selected. Each color represents a different modelling region, where grey represents countries that we did not include in our analysis. In order of appearance in the legend, the regions are: Andean South America (yellow), Central America and the Caribbean (red), China (lavender), central sub-Saharan Africa (purple), Eritrea, Djibouti and Yemen (burgundy), eastern sub-Saharan Africa (light pink), Middle East (mint green), Mongolia (pastel yellow), north Africa (blue), Oceania (lime green), southeast Asia (turquoise), south Asia (peach), southern sub-Saharan Africa (dark teal), central Asia (brown), Tropical South America (green), western sub-Saharan Africa (orange), and South Africa (navy).

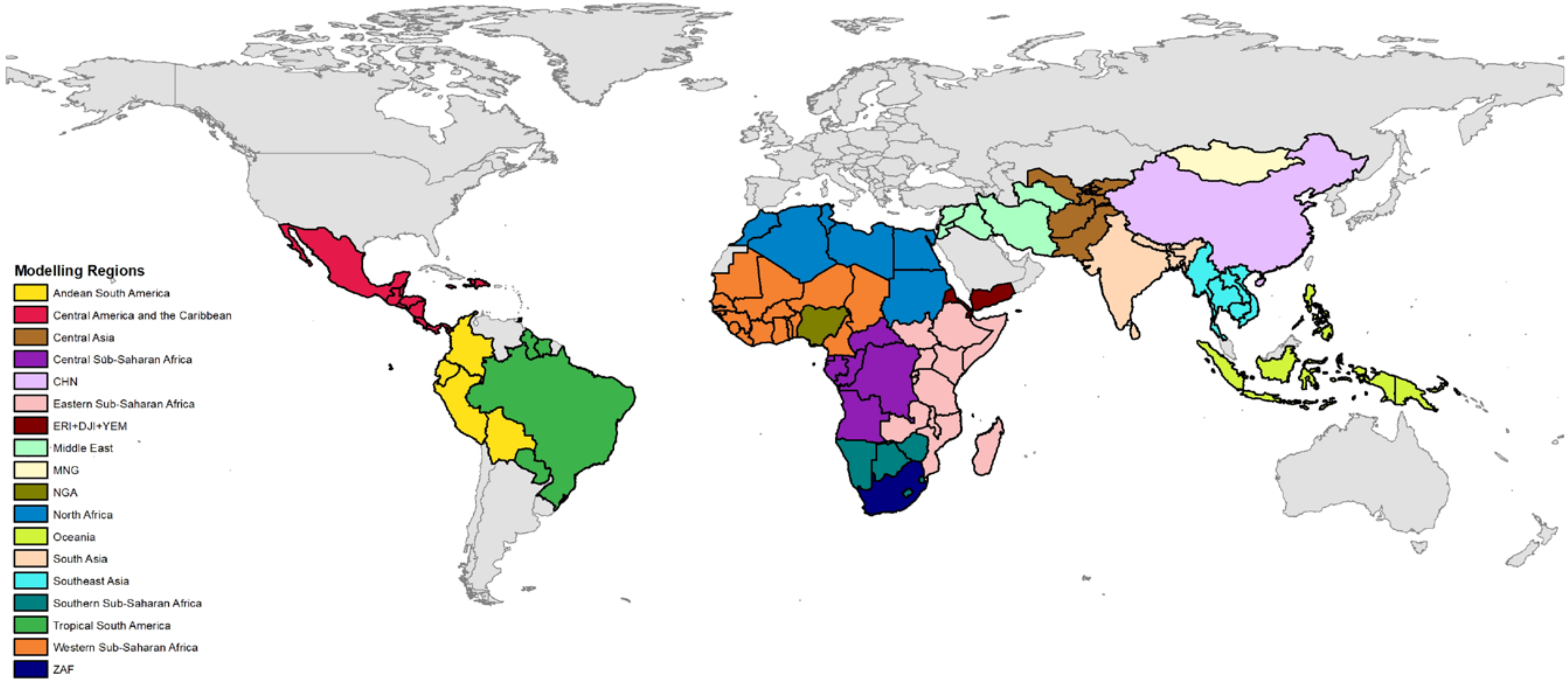

Supplementary Figure 3a-e: Solid fuel use data availability by type and country, 2000–2018

All data shown are by country and year of survey and mapped at its corresponding geopositioned coordinate or cluster area. In the left panel, the total number of points and polygons (areal) for each country are plotted by data source, data type (point coordinate or polygon area) and sample size. The sample size represents the total number of individual microdata records for each survey. In the right panel, the mean solid fuel use for the input coordinate or area is mapped. Figure **a)** displays solid fuel use data availability in Africa by type and country from 2000 to 2018. Figure **b)** displays solid fuel use data availability in Latin America and the Caribbean by type and country from 2000 to 2018. Figure **c)** displays solid fuel use data availability in Middle East and central Asia by type and country from 2000 to 2018. Figure **d)** displays solid fuel use data availability in southeast Asia by type and country from 2000 to 2018. Figure **e)** displays solid fuel use data availability in south Asia by type and country from 2000 to 2018.

a)

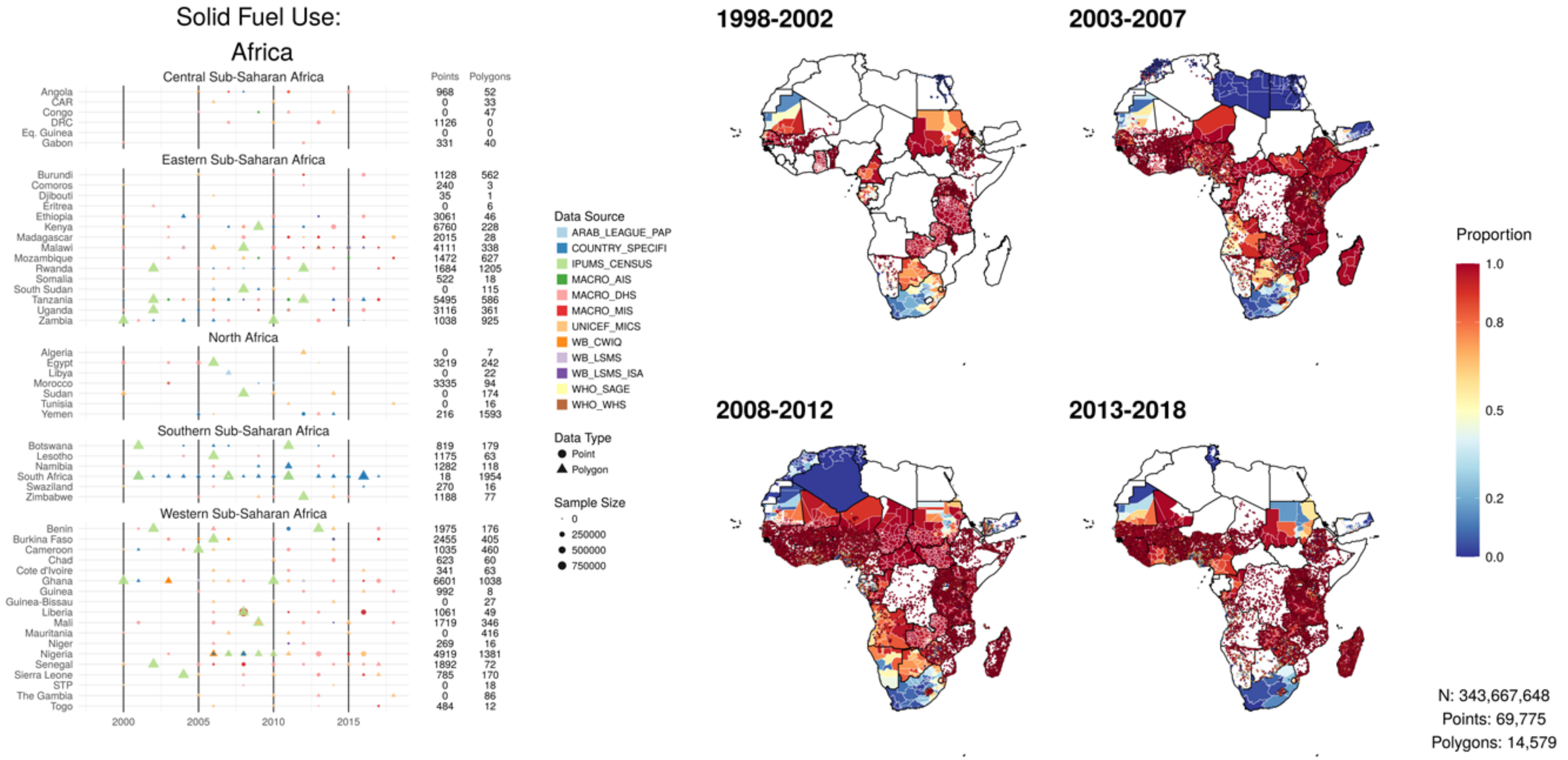

b)

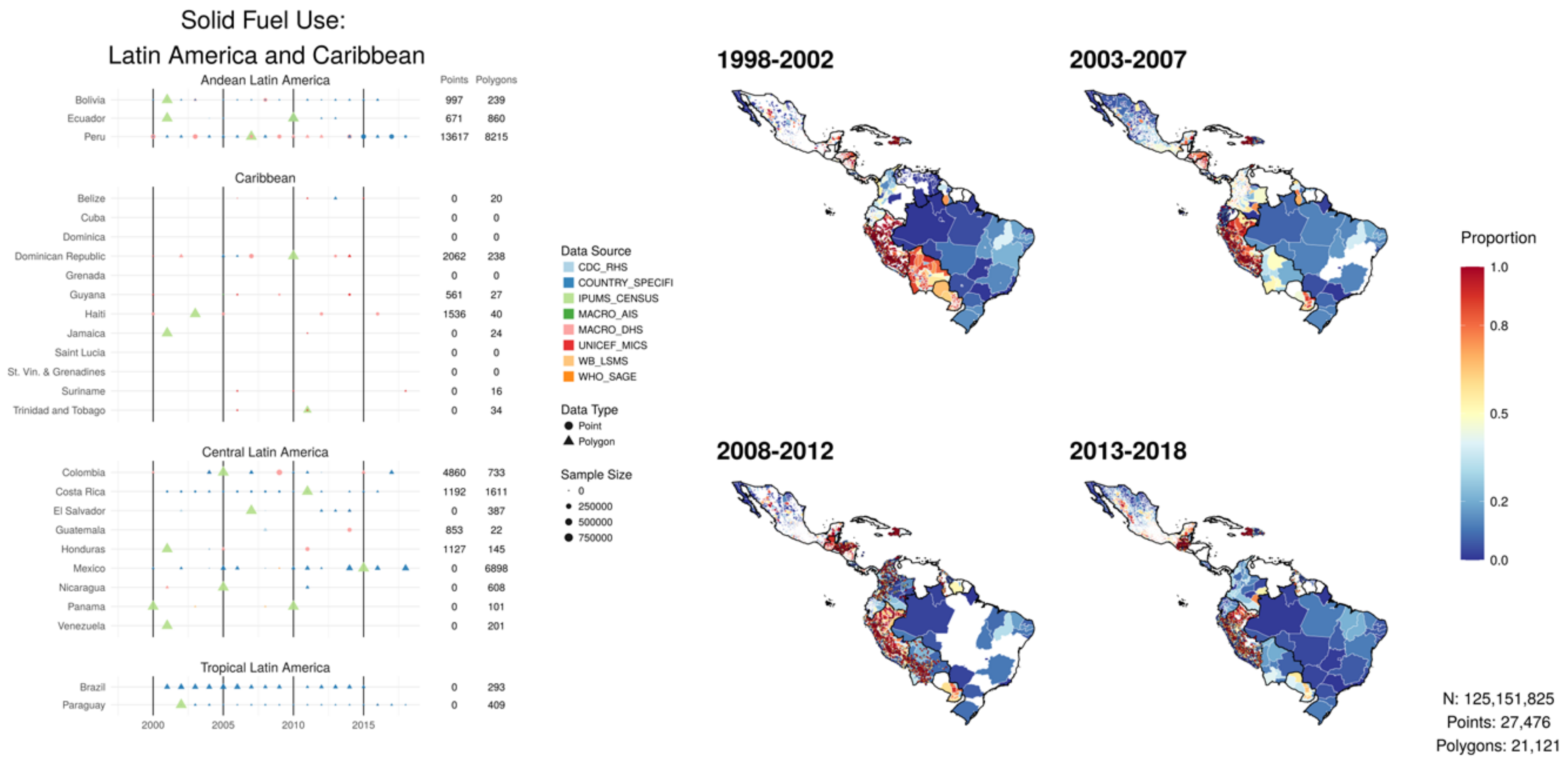
Solid Fuel Use:
Latin America and Caribbean
Andean Latin America
Points
Polygons
Bolivia 997 239
Ecuador 671 860
Peru 13617 8215
Caribbean
Belize 0 20
Cuba 0 0
Dominica 0 0
Dominican Republic 2062 238
Grenada 0 0
Guyana 561 27
Haiti 1536 40
Jamaica 0 24
Saint Lucia 0 0
St. Vin. & Grenadines 0 0
Suriname 0 16
Trinidad and Tobago 0 34
Central Latin America
Colombia 4860 733
Costa Rica 1192 1611
El Salvador 0 387
Guatemala 853 22
Honduras 1127 145
Mexico 0 6898
Nicaragua 0 608
Panama 0 101
Venezuela 0 201
Tropical Latin America
Brazil 0 293
Paraguay 0 409
2000
2005
2010
2015
Data Source
CDC_RHS
COUNTRY_SPECIFI
IPUMS_CENSUS
MACRO_AIS
MACRO_DHS
UNICEF_MICS
WB_LSMS
WHO_SAGE
Data Type
Point
Polygon
Sample Size
0
250000
500000
750000
1998-2002
2003-2007
2008-2012
2013-2018
Proportion
1.0
0.8
0.5
0.2
0.0
N: 125,151,825
Points: 27,476
Polygons: 21,121

c)

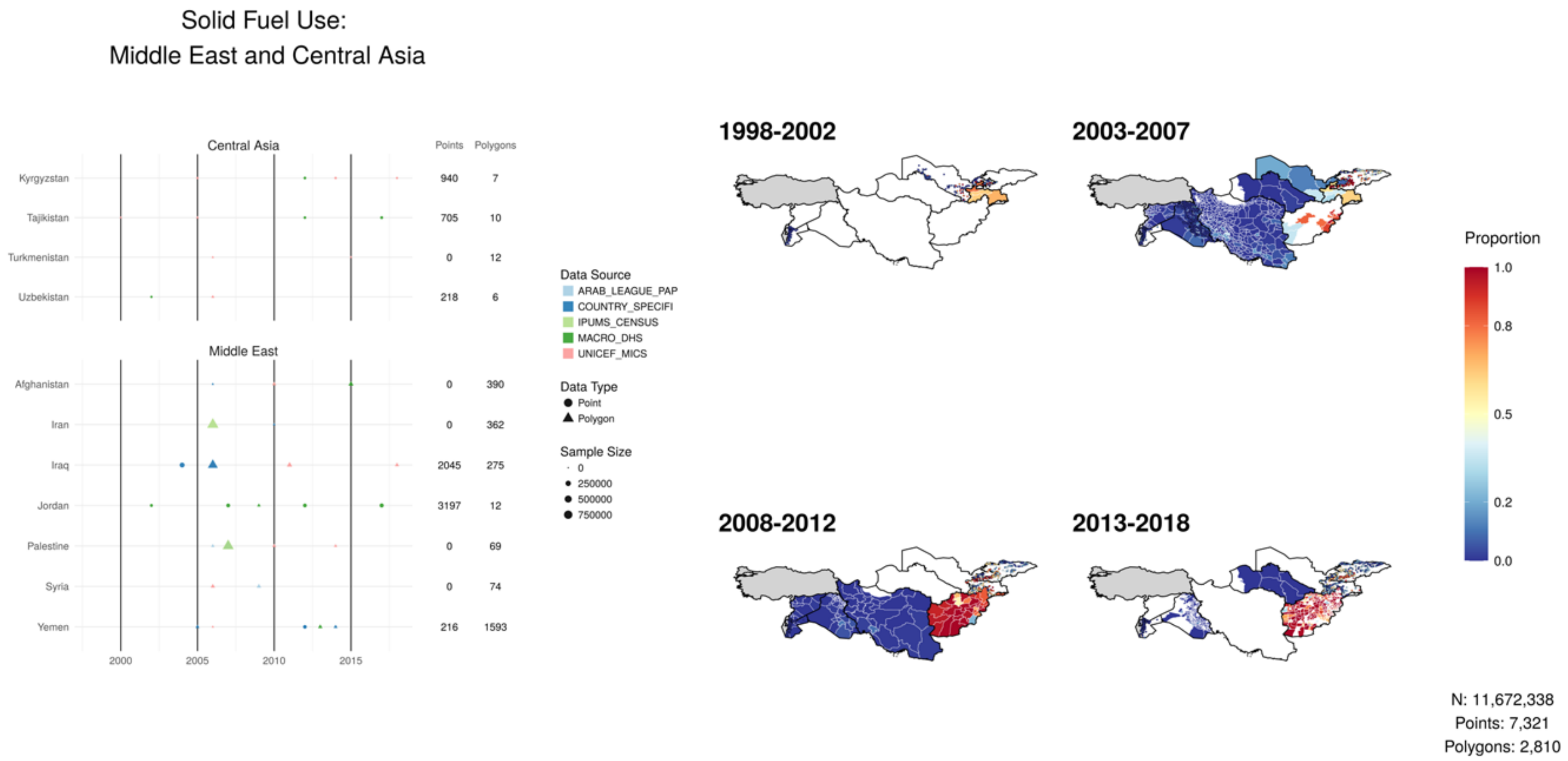
Solid Fuel Use:
Middle East and Central Asia
Central Asia
Points
Polygons
Kyrgyzstan 940 7
Tajikistan 705 10
Turkmenistan 0 12
Uzbekistan 218 6
Middle East
Afghanistan 0 390
Iran 0 362
Iraq 2045 275
Jordan 3197 12
Palestine 0 69
Syria 0 74
Yemen 216 1593
2000
2005
2010
2015
Data Source
ARAB_LEAGUE_PAP
COUNTRY_SPECIFI
IPUMS_CENSUS
MACRO_DHS
UNICEF_MICS
Data Type
Point
Polygon
Sample Size
0
250000
500000
750000
1998-2002
2003-2007
2008-2012
2013-2018
Proportion
1.0
0.8
0.5
0.2
0.0
N: 11,672,338
Points: 7,321
Polygons: 2,810

d)

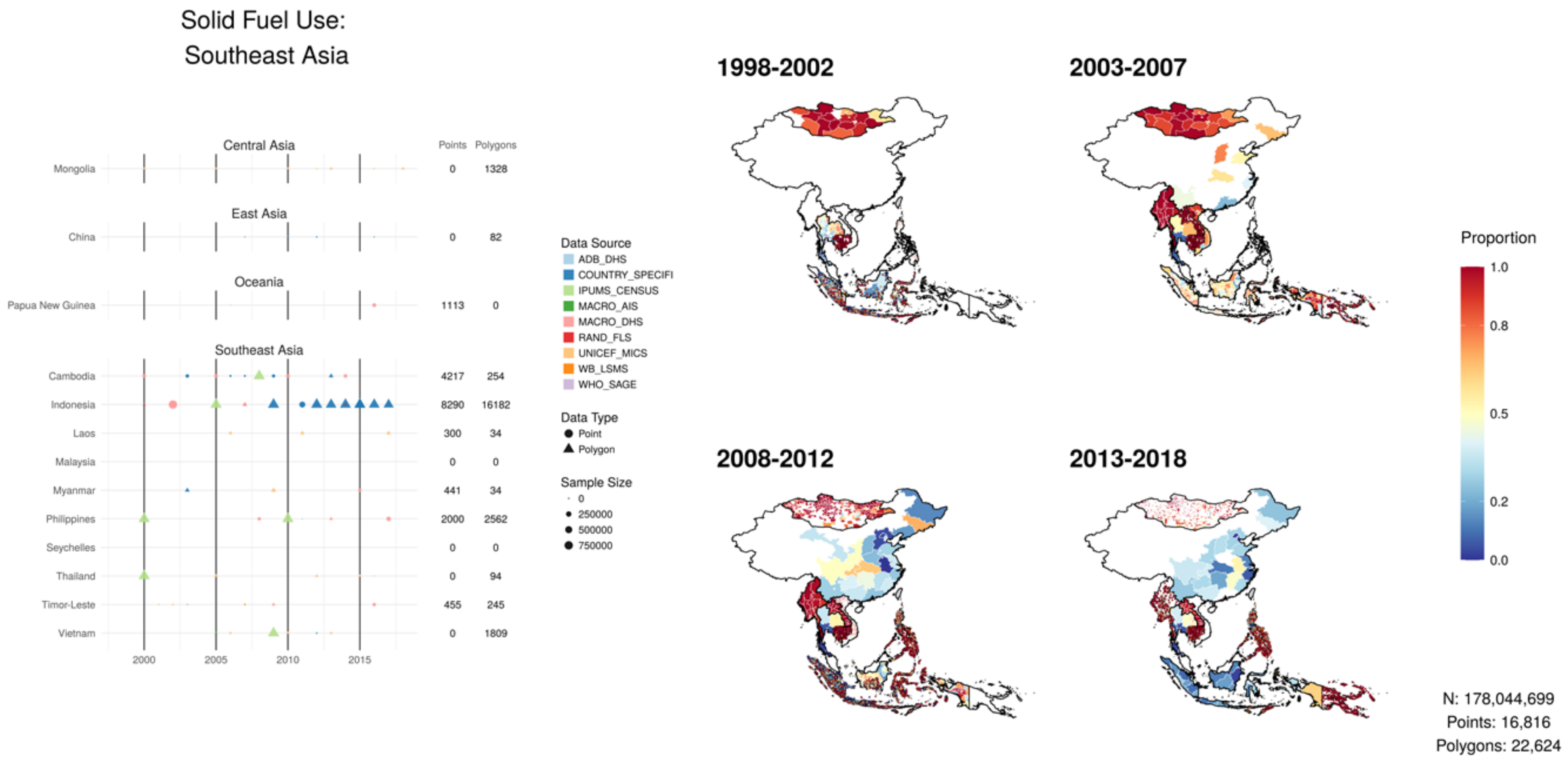
Solid Fuel Use:
Southeast Asia
Central Asia
Points
Polygons
Mongolia
0
1328
East Asia
China
0
82
Oceania
Papua New Guinea
1113
0
Southeast Asia
Cambodia
4217
254
Indonesia
8290
16182
Laos
300
34
Malaysia
0
0
Myanmar
441
34
Philippines
2000
2562
Seychelles
0
0
Thailand
0
94
Timor-Leste
455
245
Vietnam
0
1809
2000
2005
2010
2015
Data Source
ADB_DHS
COUNTRY_SPECIFI
IPUMS_CENSUS
MACRO_AIS
MACRO_DHS
RAND_FLS
UNICEF_MICS
WB_LSMS
WHO_SAGE
Data Type
Point
Polygon
Sample Size
0
250000
500000
750000
1998-2002
2003-2007
2008-2012
2013-2018
Proportion
1.0
0.8
0.5
0.2
0.0
N: 178,044,699
Points: 16,816
Polygons: 22,624

e)

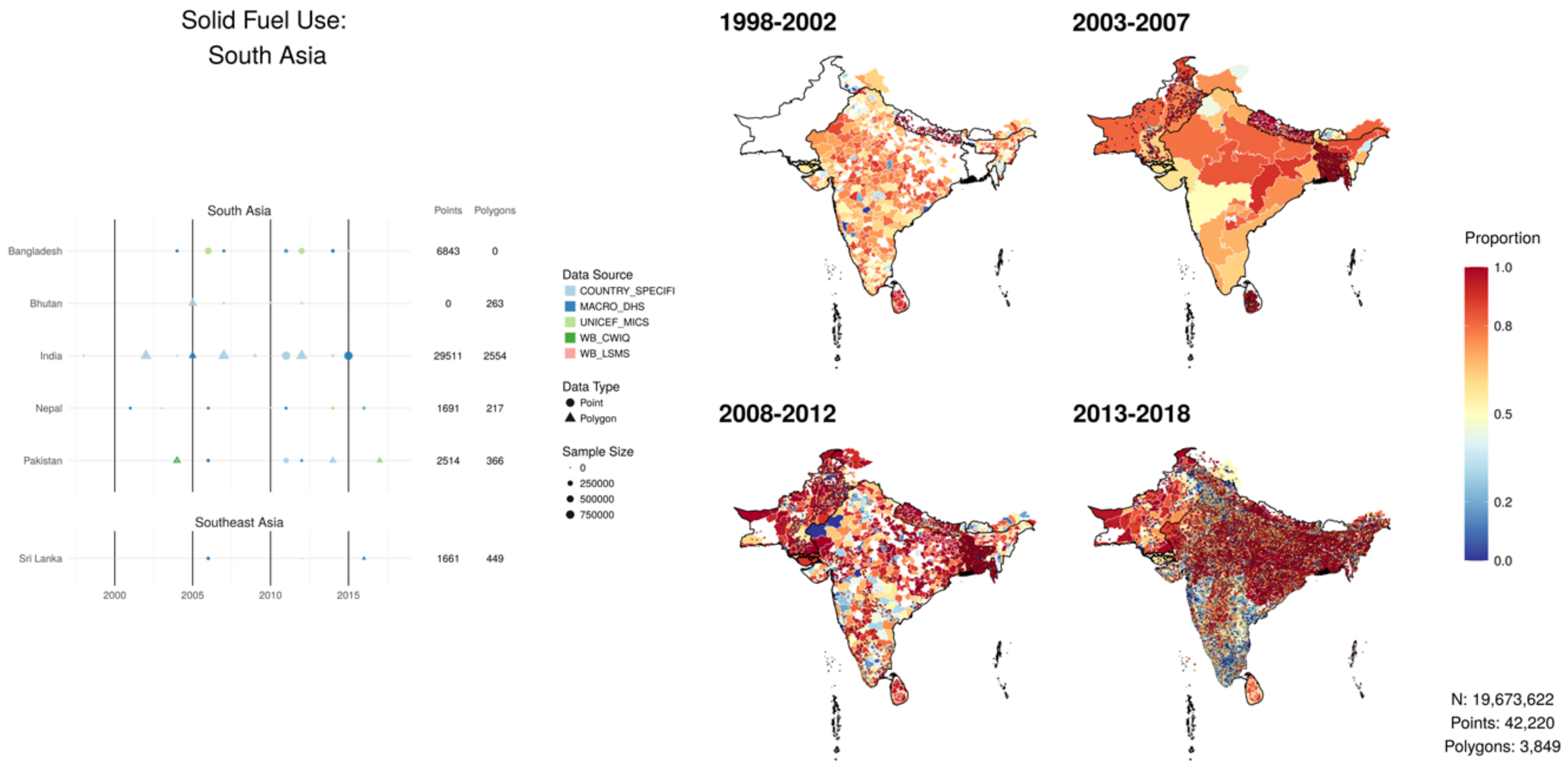
Solid Fuel Use:
South Asia
South Asia
Points
Polygons
Bangladesh
6843
0
Bhutan
0
263
India
29511
2554
Nepal
1691
217
Pakistan
2514
366
Southeast Asia
Sri Lanka
1661
449
2000
2005
2010
2015
Data Source
COUNTRY_SPECIFI
MACRO_DHS
UNICEF_MICS
WB_CWIQ
WB_LSMS
Data Type
Point
Polygon
Sample Size
0
250000
500000
750000
1998-2002
2003-2007
2008-2012
2013-2018
Proportion
1.0
0.8
0.5
0.2
0.0
N: 19,673,622
Points: 42,220
Polygons: 3,849

Supplementary Figure 5: Geospatial covariates

23 covariate raster layers of possible socioeconomic and environmental correlates of solid fuel use were used as inputs for the stacking modelling process. Time-varying covariates are presented for the year 2018. For the year of production of non-time-varying covariates and additional details, please refer to the individual covariate citation in Supplementary Table 4 for additional details. These maps reflect administrative boundaries, land cover, lakes, and population. Pixels with fewer than ten people per 1x1 km grid cell and classified as “barren” or “sparsely vegetated” are colored in grey. Covariates are labeled as follows: travel time to nearest settlement [*access2*], aridity [*aridity*], diurnal temperature range [*crutsdtr*], frost day frequency [*crutsfrs*], potential evapotranspiration [*crutspet*], average daily mean temperature [*crutstmp*], dependency ratio of dependents to working-age adults [*depratio*], distance from rivers or lakes ≥ 50 km^2 [*distriverslakes*], nighttime lights [*dmspntl*], elevation [*elevation*], agricultural land [*escroplandarea*], enhanced vegetation index [*evi*], fertility [*fertility*], urban or rural [*ghslurbanicity*], nutrient yield [*herreronyield*], irrigation [*irrigation*], urban proportion of location [*landcover*], average land surface temperature [*lst_avg_v6*], precipitation [*mswep*], normalised difference vegetation index [*nexndvi*], tassled cap brightness [*tcb_v6*], tassled cap wetness [*tcw_v6*], and population [*worldpop*].

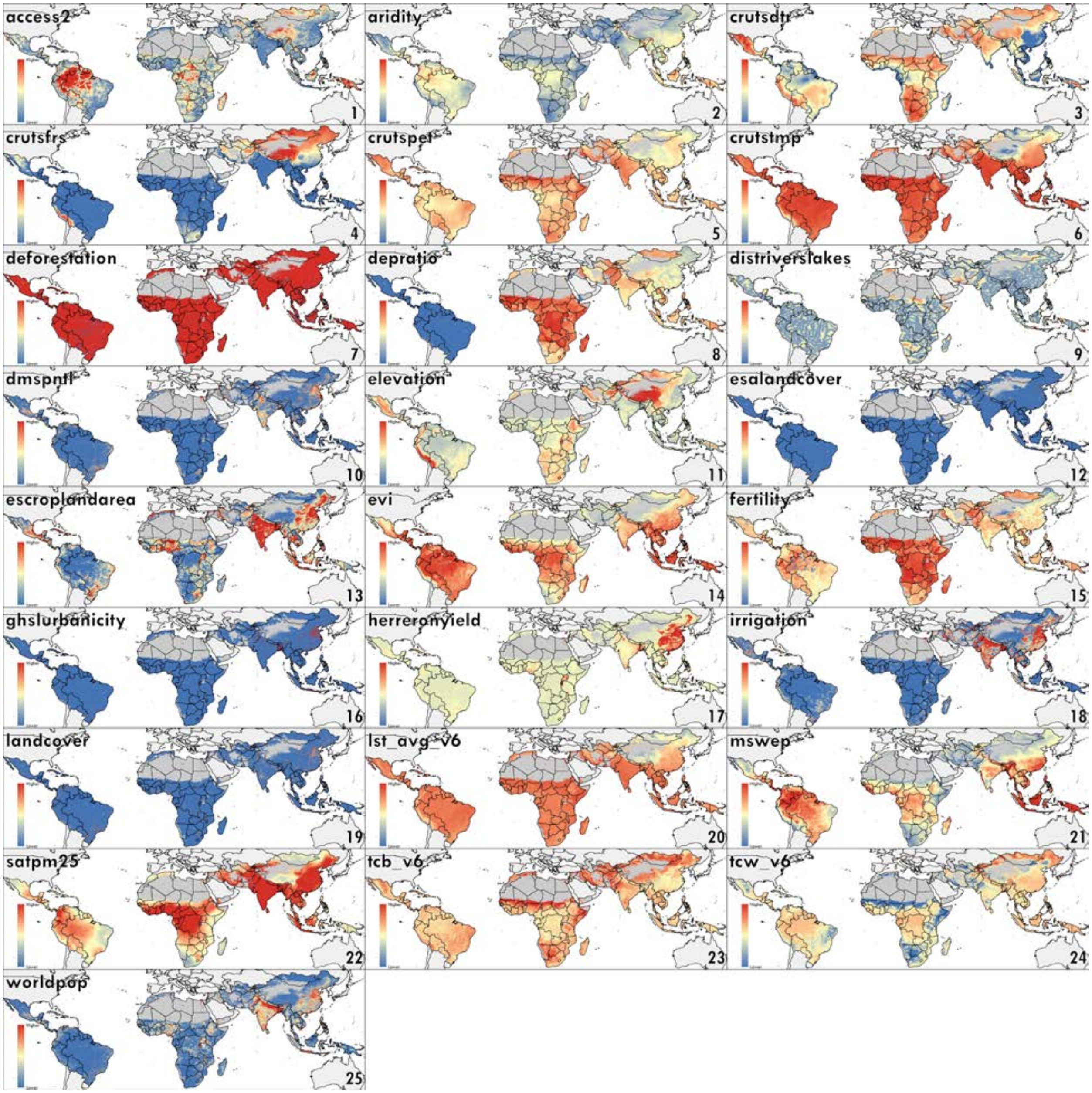
access2
1
aridity
2
crutsdtr
3
crutsfrs
4
crutspet
5
crutstmp
6
deforestation
7
depratio
8
distriverslakes
9
dmspntl
10
elevation
11
esalandcover
12
escroplandarea
13
evi
14
fertility
15
ghslurbanicity
16
herreronyield
17
irrigation
18
landcover
19
lst_avg_v6
20
mswep
21
satpm25
22
tcb_v6
23
tcw_v6
24
worldpop
25

Supplementary Figure 5: Finite elements mesh

The finite elements mesh used to fit the space-time correlated error for the north Africa (NOAF) region overlaid on the countries in NOAF. Both the fine-scale mesh over land in the modelling region as well as the coarser buffer region mesh are shown. The simplified region polygon used to determine the boundary for the modelling region is shown in blue.

Finite elements mesh over north Africa

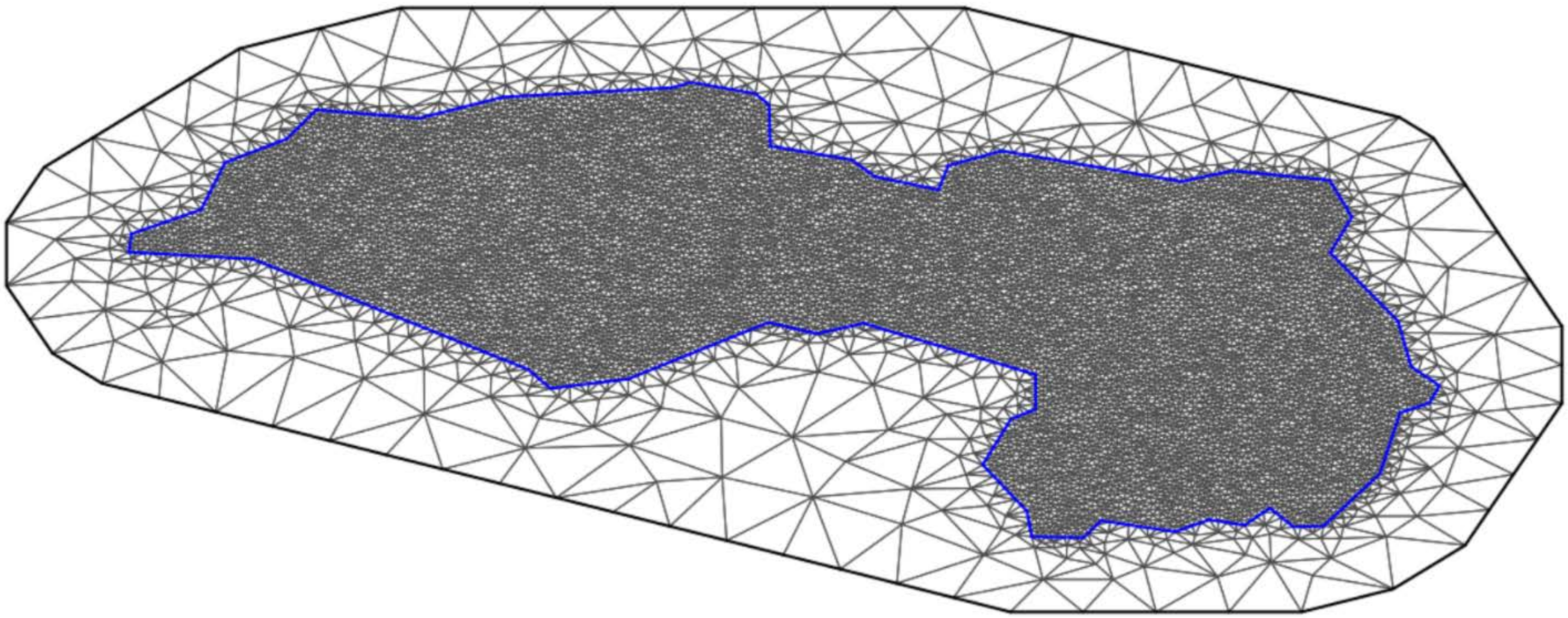

Supplementary Figure 6: Posterior means and 95% uncertainty intervals for solid fuel use prevalence at the second administrative level, 2018

95% uncertainty intervals were calculated as the difference between the 97.5th percentile and the 2.5th percentile of 1000 draws from the posterior distribution. This map presents uncertainty at the second administrative level for solid fuel use prevalence. Maps reflect administrative boundaries, land cover, lakes, and population; grey-coloured grid cells were classified as “barren or sparsely vegetated” and had fewer than ten people per 1x1km grid cell, or were not included in these analyses.

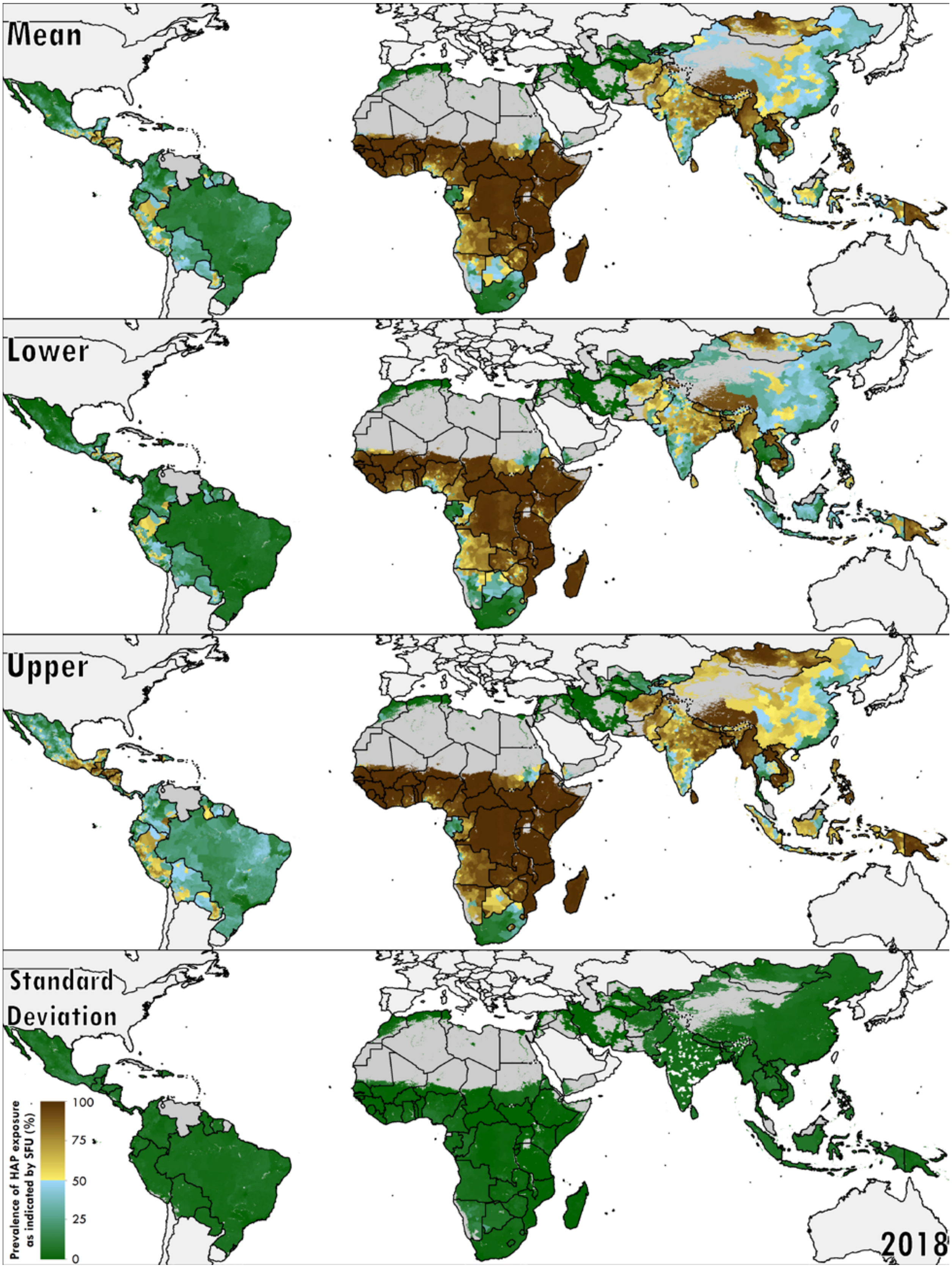

Mean
Lower
Upper
Standard Deviation
Prevalence of HAP exposure as indicated by SFU (%)
100
75
50
25
0
2018

Supplementary Figure 7: Posterior means and 95% uncertainty intervals for TAP dose at the second administrative level, 2018

95% uncertainty intervals were calculated as the difference between the 97.5th percentile and the 2.5th percentile of 1000 draws from the posterior distribution. This map presents uncertainty at the second administrative level for TAP dose. Maps reflect administrative boundaries, land cover, lakes, and population; grey-coloured grid cells were classified as “barren or sparsely vegetated” and had fewer than ten people per 1x1km grid cell, or were not included in these analyses.

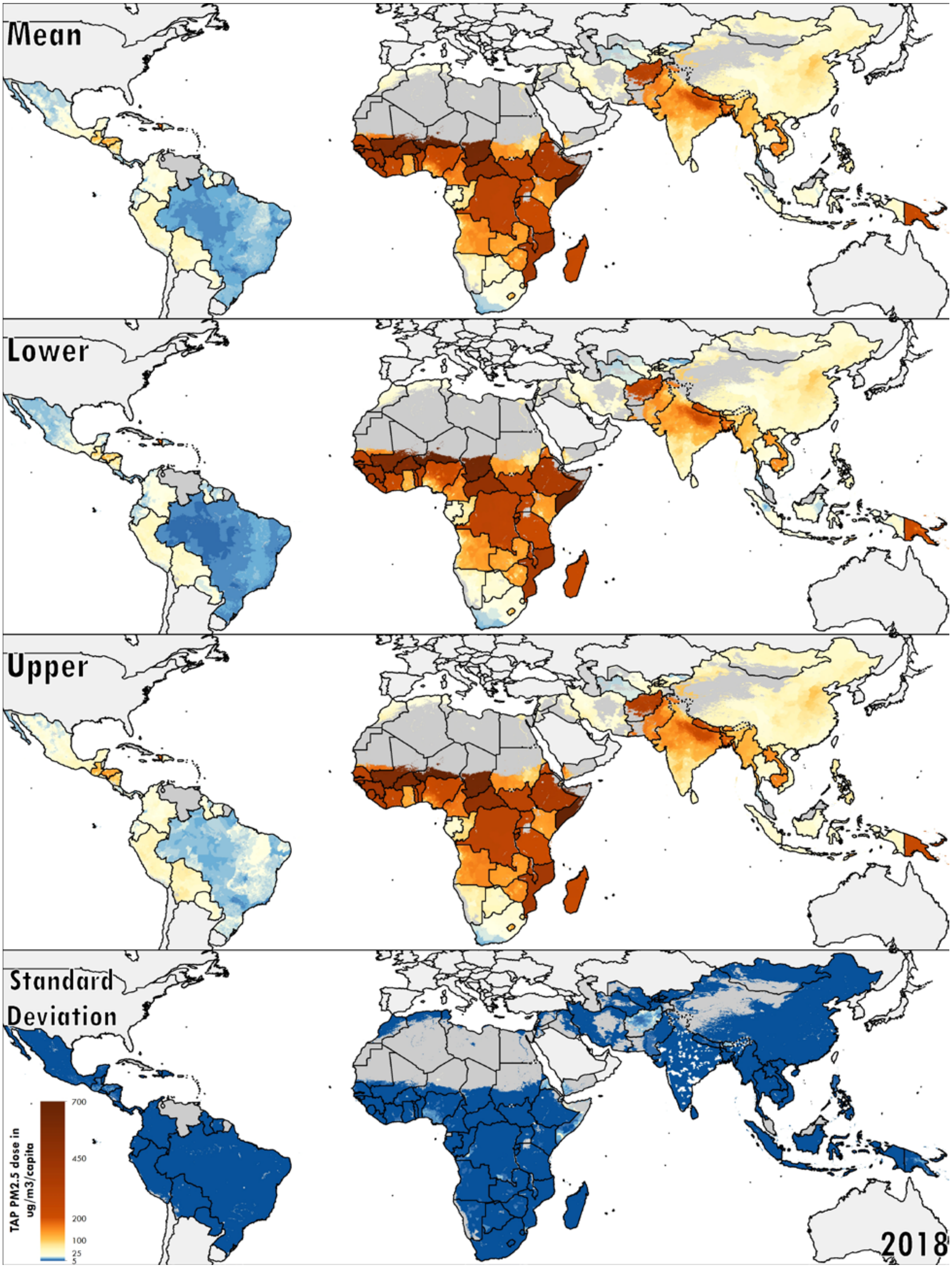
Mean
Lower
Upper
Standard Deviation
TAP PM2.5 dose in ug/m3/capita
700
450
200
100
25
5
2018

Supplementary Figure 8: Posterior means and 95% uncertainty intervals for TAP dose at the first administrative level, 2018

95% uncertainty intervals were calculated as the difference between the 97.5th percentile and the 2.5th percentile of 1000 draws from the posterior distribution. This map presents uncertainty at the first administrative level for TAP dose. Maps reflect administrative boundaries, land cover, lakes, and population; grey-coloured grid cells were classified as “barren or sparsely vegetated” and had fewer than ten people per 1x1km grid cell, or were not included in these analyses.

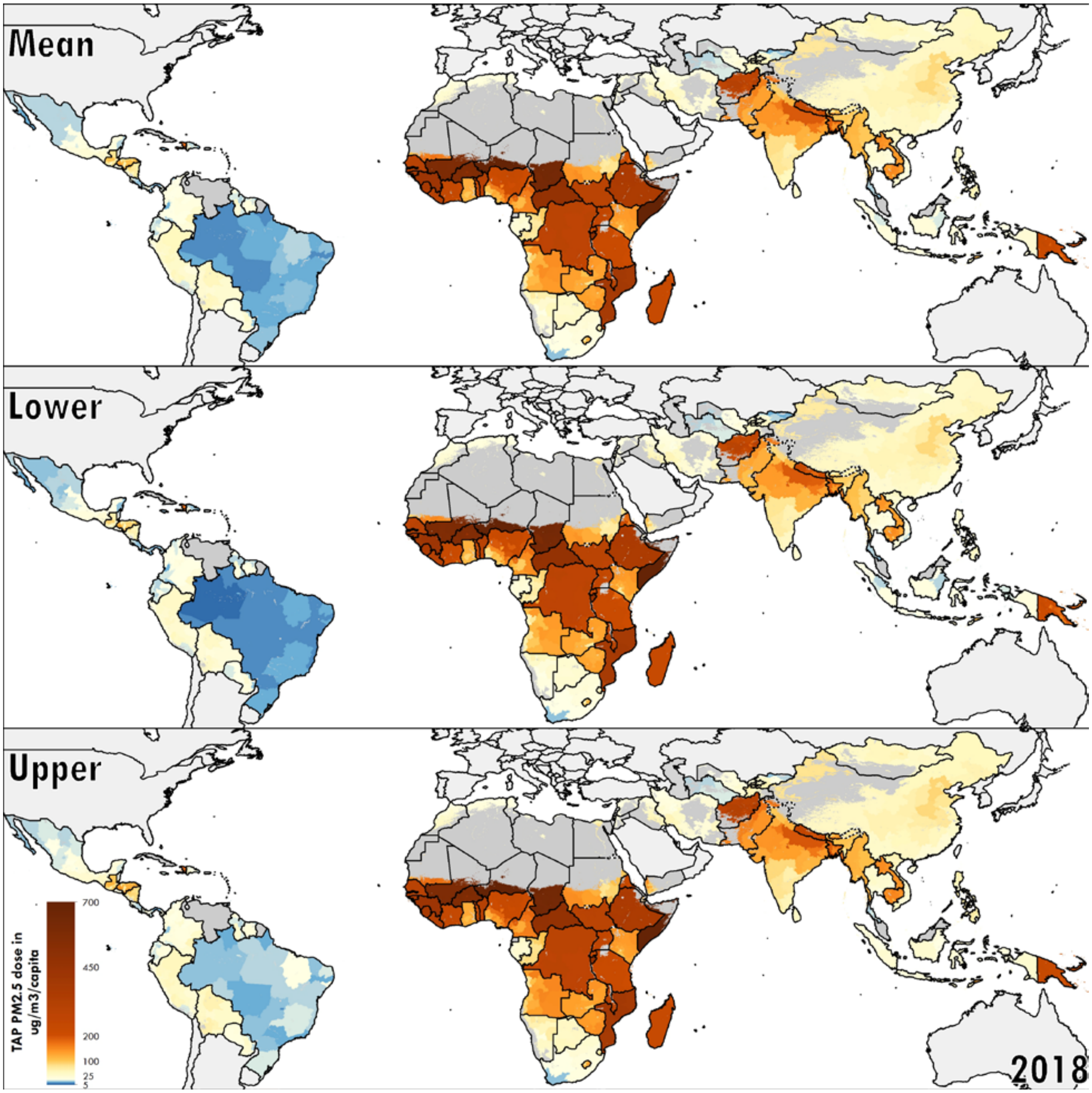

Supplementary Figure 9: Posterior means and 95% uncertainty intervals for HAP share at the second administrative level, 2018

95% uncertainty intervals were calculated as the difference between the 97.5th percentile and the 2.5th percentile of 1000 draws from the posterior distribution. This map presents uncertainty at the second administrative level for HAP ratio. Maps reflect administrative boundaries, land cover, lakes, and population; grey-coloured grid cells were classified as “barren or sparsely vegetated” and had fewer than ten people per 1x1km grid cell, or were not included in these analyses.

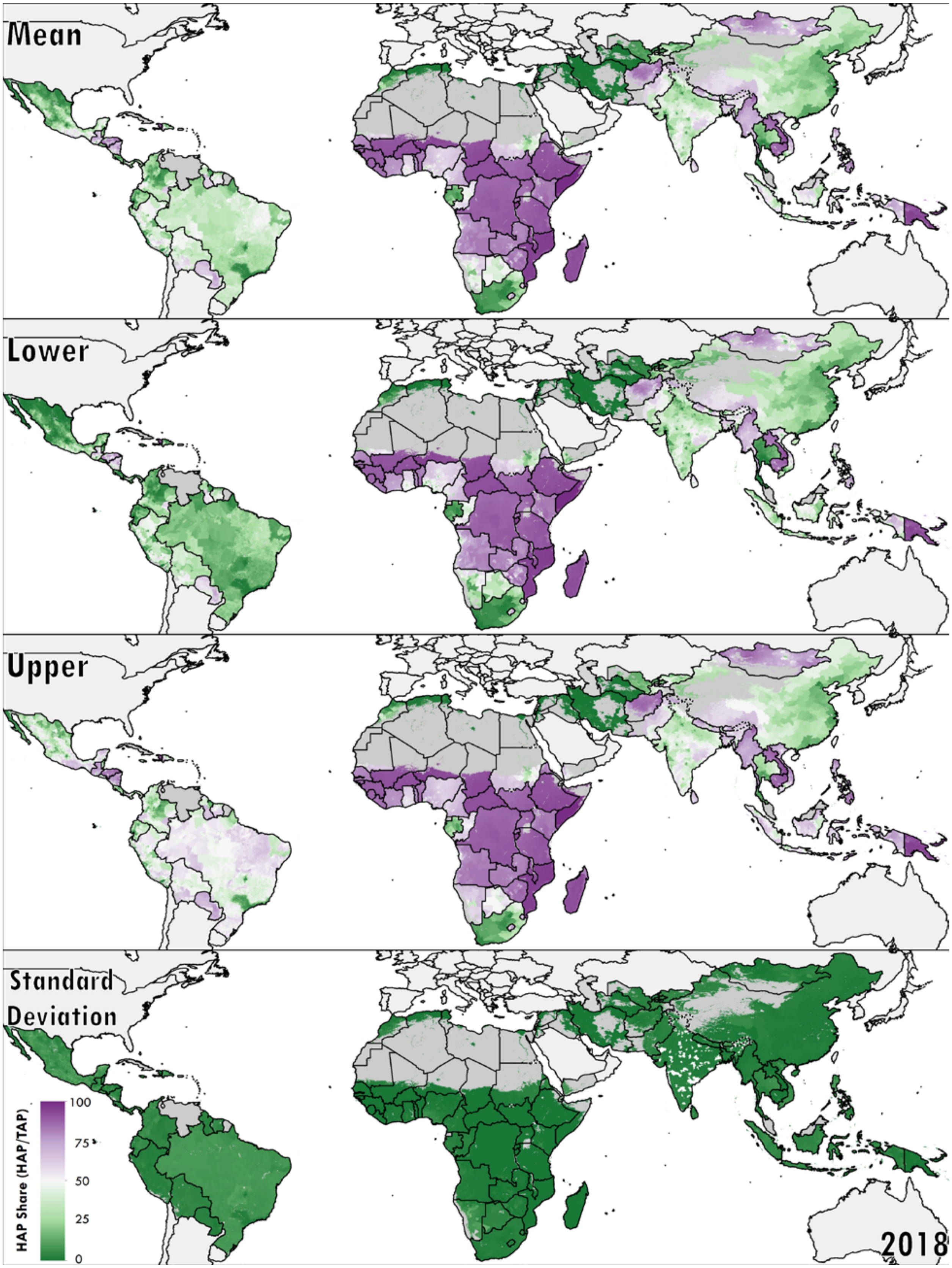
Mean
Lower
Upper
Standard Deviation
HAP Share (HAP/TAP)
100
75
50
25
0
2018

Supplementary Figure 10: Posterior means and 95% uncertainty intervals for HAP share at the first administrative level, 2018

95% uncertainty intervals were calculated as the difference between the 97.5th percentile and the 2.5th percentile of 1000 draws from the posterior distribution. This map presents uncertainty at the first administrative level for HAP ratio. Maps reflect administrative boundaries, land cover, lakes, and population; grey-coloured grid cells were classified as “barren or sparsely vegetated” and had fewer than ten people per 1x1km grid cell, or were not included in these analyses.

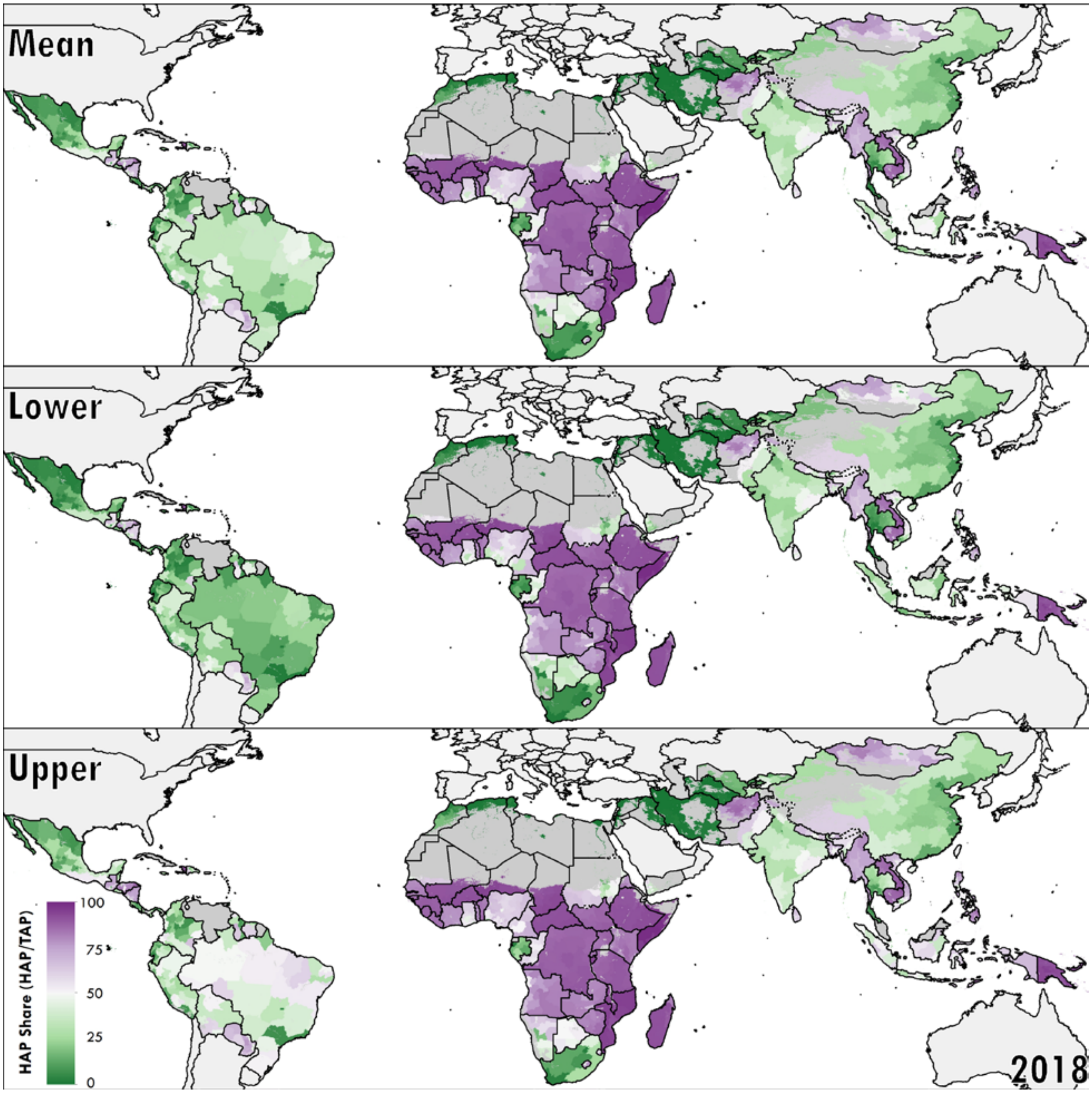

Supplementary Figure 11: Posterior means and 95% uncertainty intervals for LRI mortality counts due to TAP at the second administrative level, 2018

95% uncertainty intervals were calculated as the difference between the 97.5th percentile and the 2.5th percentile of 1000 draws from the posterior distribution. This map presents uncertainty at the second administrative level for LRI mortality counts due to TAP. Maps reflect administrative boundaries, land cover, lakes, and population; grey-coloured grid cells were classified as “barren or sparsely vegetated” and had fewer than ten people per 1x1km grid cell, or were not included in these analyses.

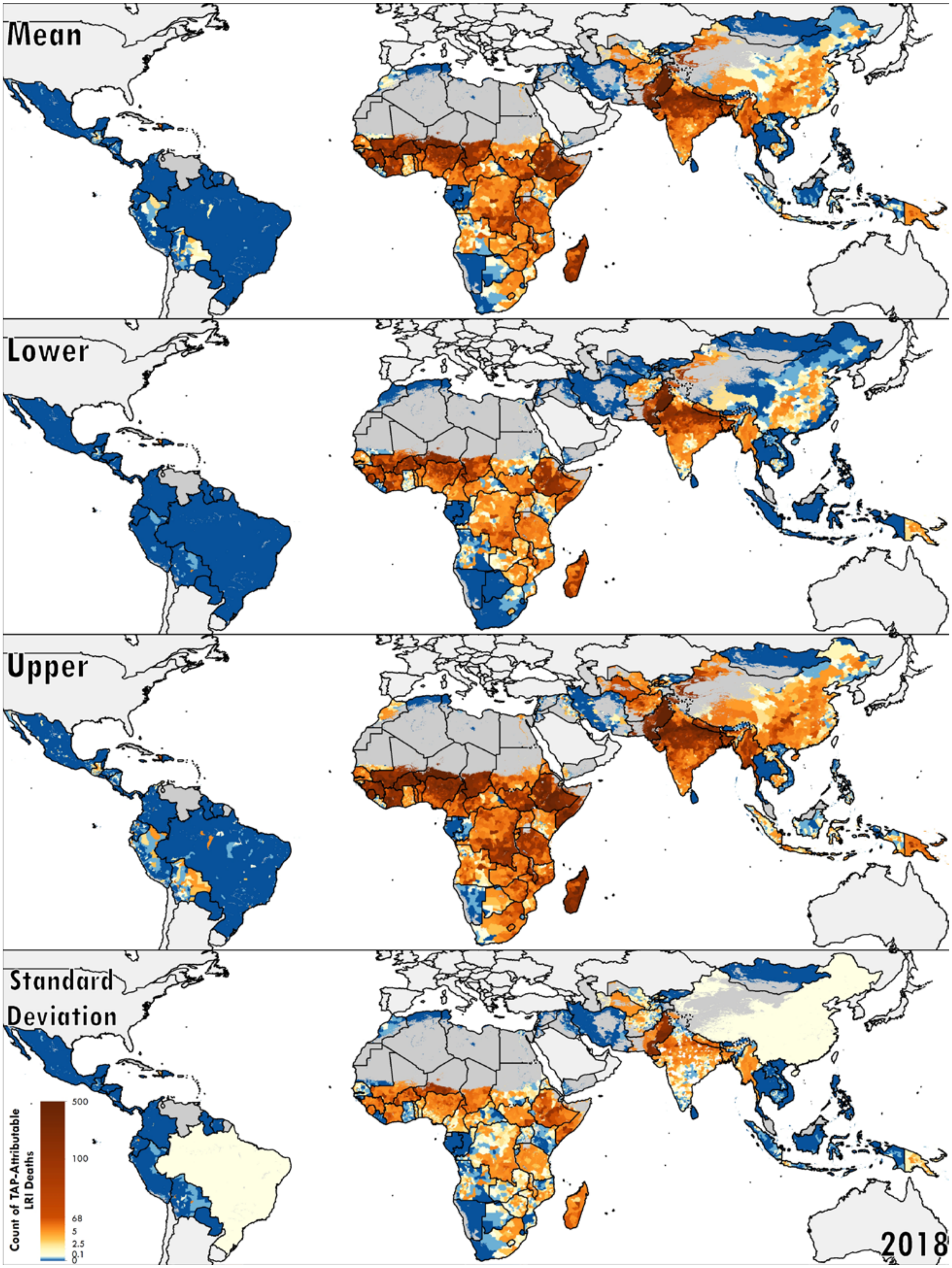

Mean
Lower
Upper
Standard Deviation
Count of TAP-Attributable LRI Deaths
500
100
68
5
2.5
0.1
0
2018

Supplementary Figure 12: Posterior means and 95% uncertainty intervals for LRI mortality counts due to TAP at the first administrative level, 2018

95% uncertainty intervals were calculated as the difference between the 97.5th percentile and the 2.5th percentile of 1000 draws from the posterior distribution. This map presents uncertainty at the first administrative level for LRI mortality counts due to TAP. Maps reflect administrative boundaries, land cover, lakes, and population; grey-coloured grid cells were classified as "barren or sparsely vegetated" and had fewer than ten people per 1x1km grid cell, or were not included in these analyses.

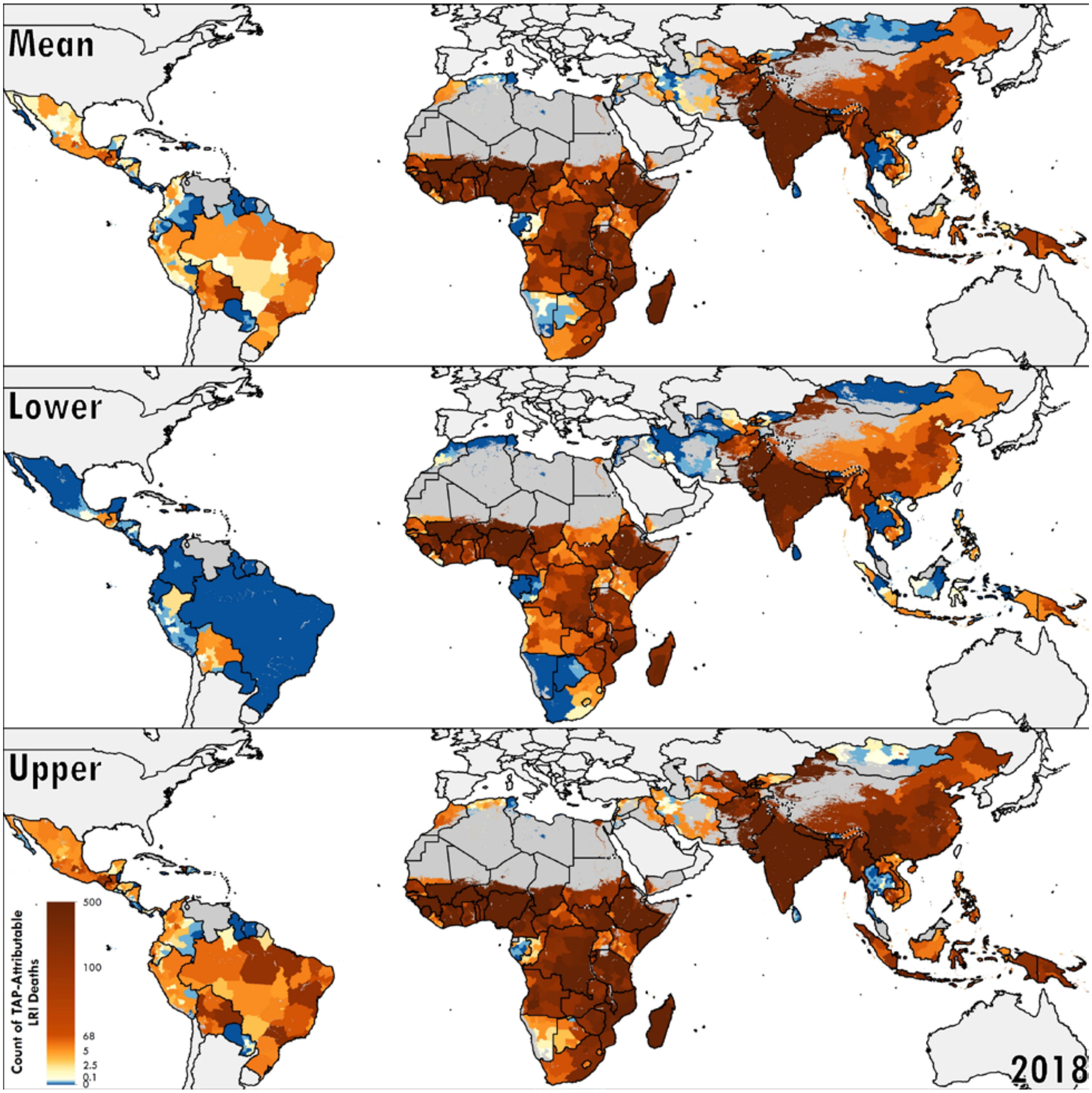

Supplementary Figure 13: Posterior means and 95% uncertainty intervals for LRI mortality rates due to TAP at the second administrative level, 2018

95% uncertainty intervals were calculated as the difference between the 97.5th percentile and the 2.5th percentile of 1000 draws from the posterior distribution. This map presents uncertainty at the second administrative level for LRI mortality rates due to TAP. Maps reflect administrative boundaries, land cover, lakes, and population; grey-coloured grid cells were classified as “barren or sparsely vegetated” and had fewer than ten people per 1x1km grid cell, or were not included in these analyses.

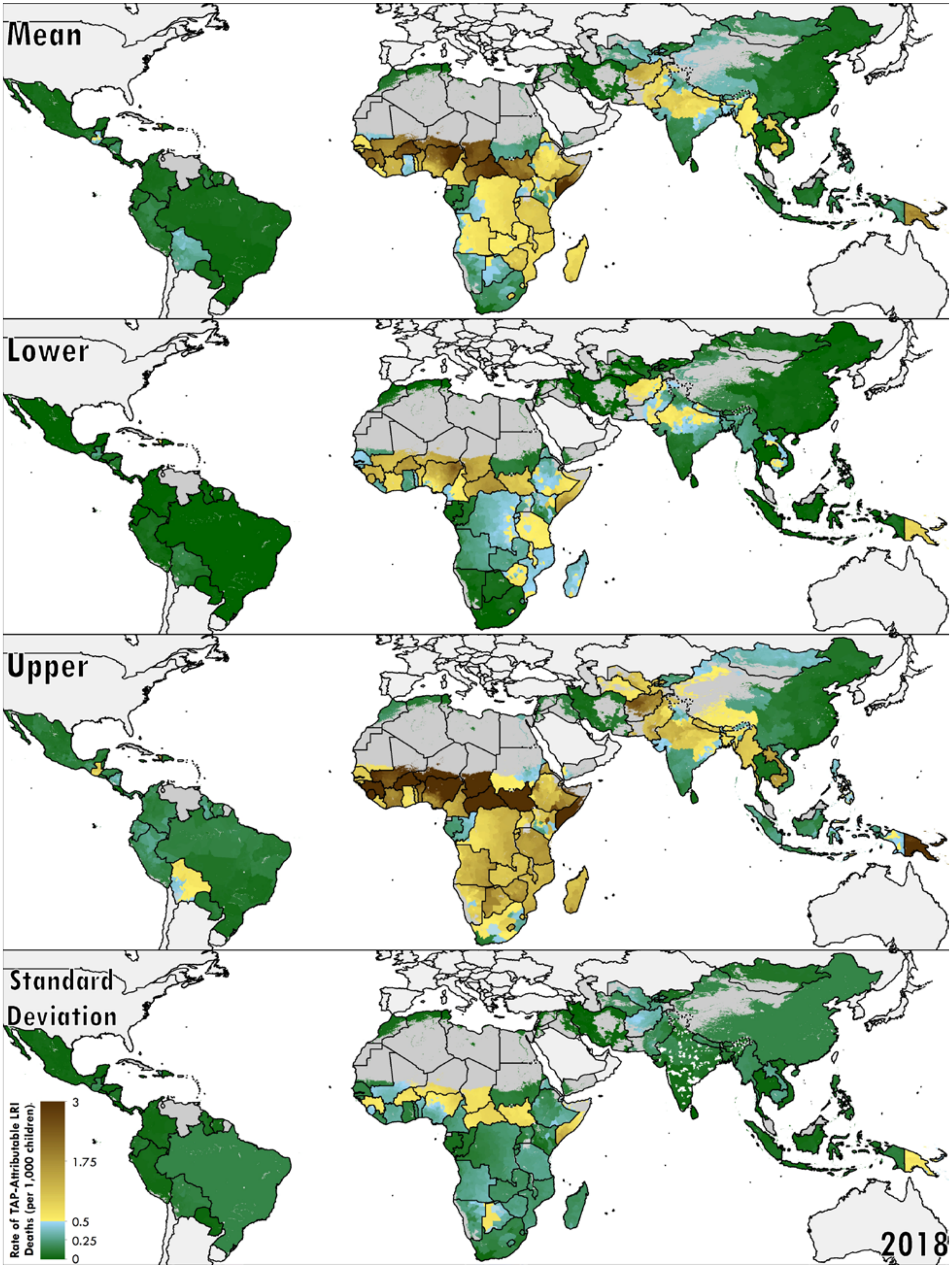
Mean
Lower
Upper
Standard Deviation
Rate of TAP-Attributable LRI Deaths (per 1,000 children)
3
1.75
0.5
0.25
0
2018

Supplementary Figure 14: Posterior means and 95% uncertainty intervals for LRI mortality rates due to TAP at the first administrative level, 2018

95% uncertainty intervals were calculated as the difference between the 97.5th percentile and the 2.5th percentile of 1000 draws from the posterior distribution. This map presents uncertainty at the first administrative level for LRI mortality rates due to TAP. Maps reflect administrative boundaries, land cover, lakes, and population; grey-coloured grid cells were classified as “barren or sparsely vegetated” and had fewer than ten people per 1x1km grid cell, or were not included in these analyses.

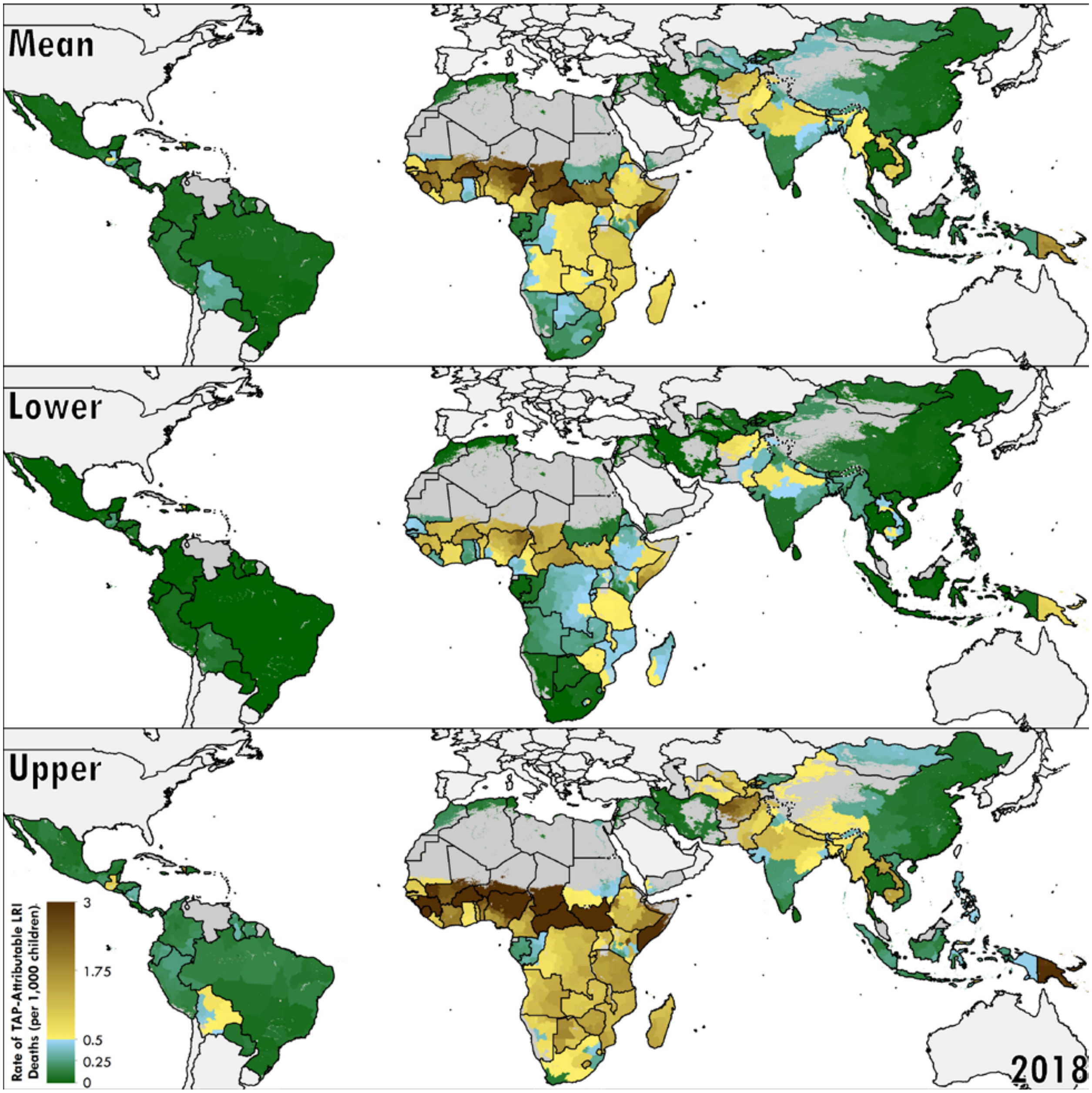

Supplementary Figure 15: HAP percentage change, 2000–2018, at the second administrative level

Estimated percentage decrease in household air pollution from 2000 to 2018. Maps display the rates aggregated up to the second administrative level using population weighting and reflects administrative boundaries, land cover, lakes, and population; grey-coloured grid cells were classified as “barren or sparsely vegetated” and had fewer than ten people per 1x1km grid cell, or were not included in these analyses.

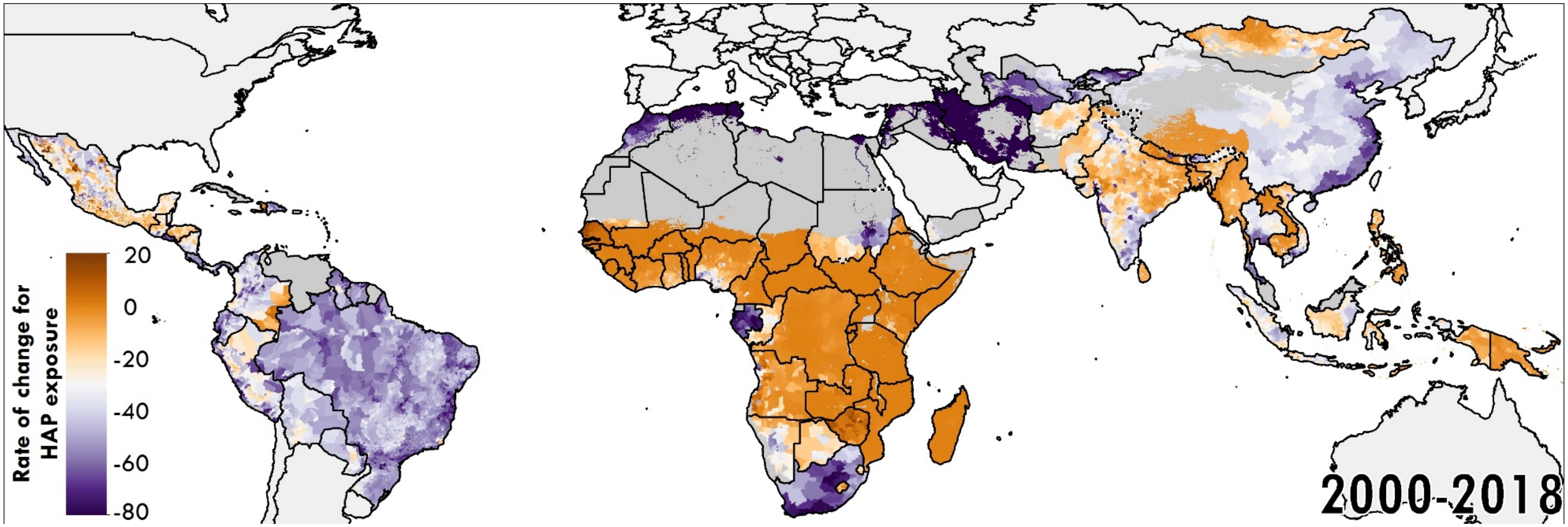

Supplementary Figure 17a-u: Population and attributable under-5 LRI mortalities, distributed as a function of PM2.5 by region.

Population (a) and under-5 LRI mortality attributable to total PM2.5 (b) for the year 2018, plotted a function of total PM2.5 concentrations. The mirror image reflects the distribution for the year 2000. The distributions are colored bycountry. Within each distribution, the dark shading represents the portion contributed by household sources, while the lighter shading indicates the portion contributed by ambient sources. The plotted data represent local smoothing of normalized distributions that were computed over 400 logarithmically spaced bins. The dashed vertical line indicates WHO's interim threshold of 35 ug/m3.

a) Southeast Asia

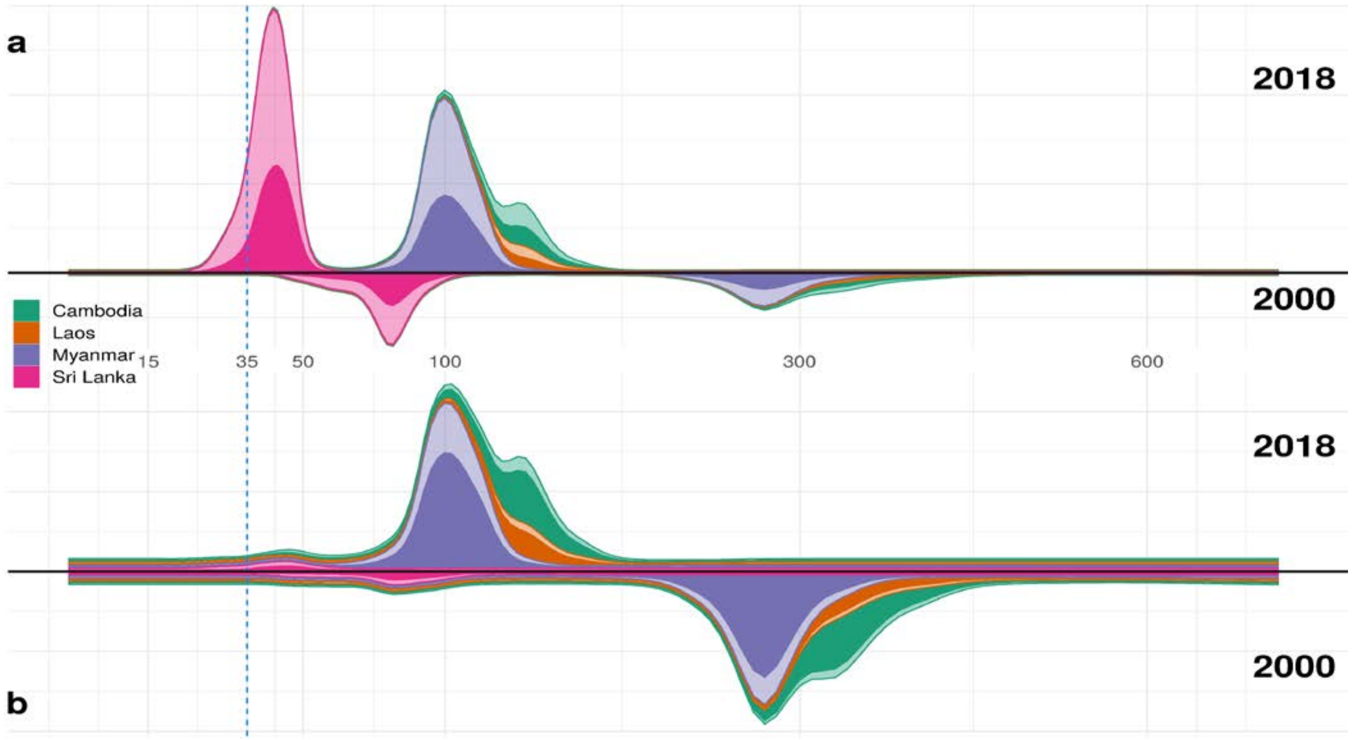

b) Southeast Asia, continued

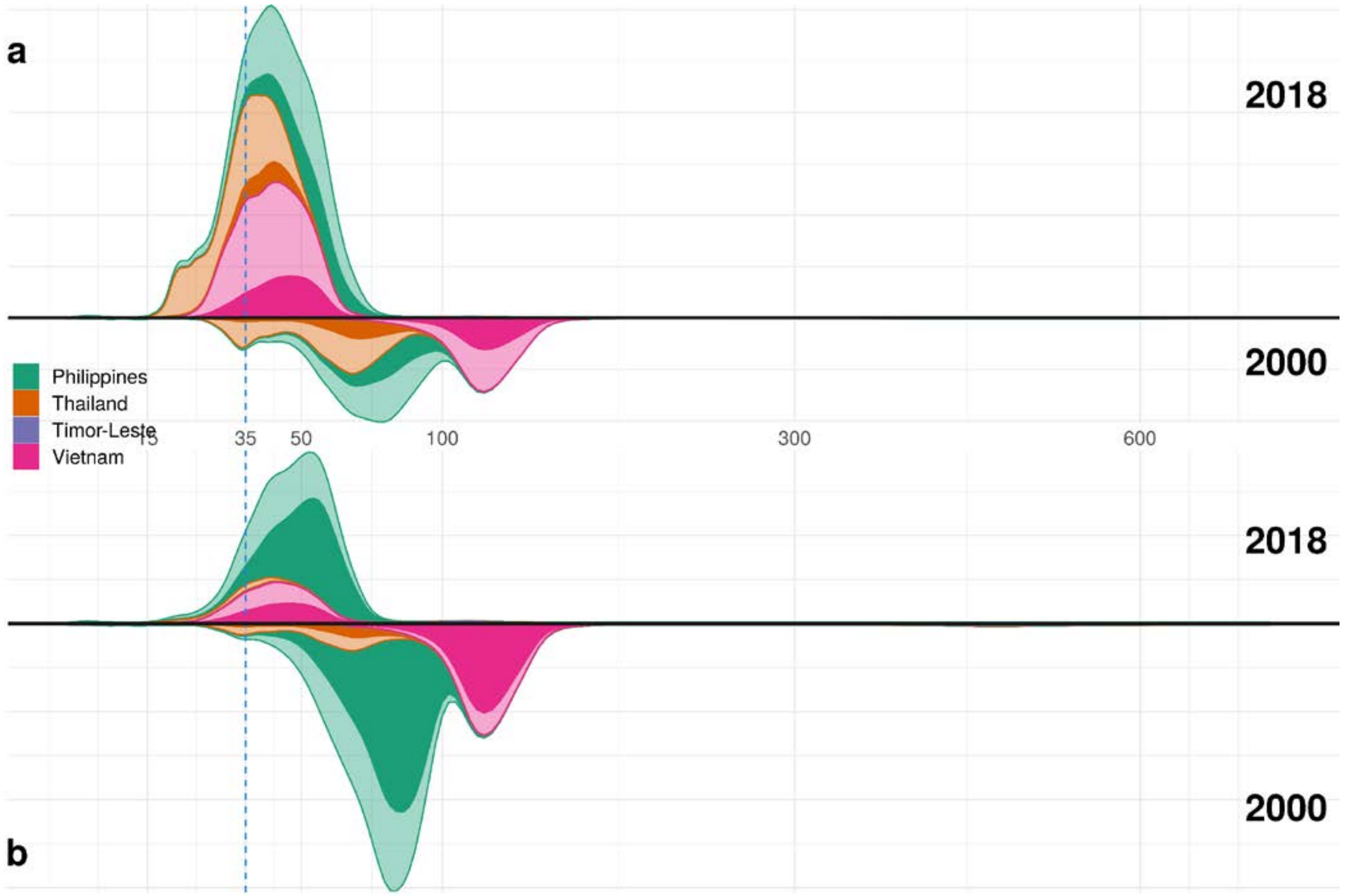

c) Oceania

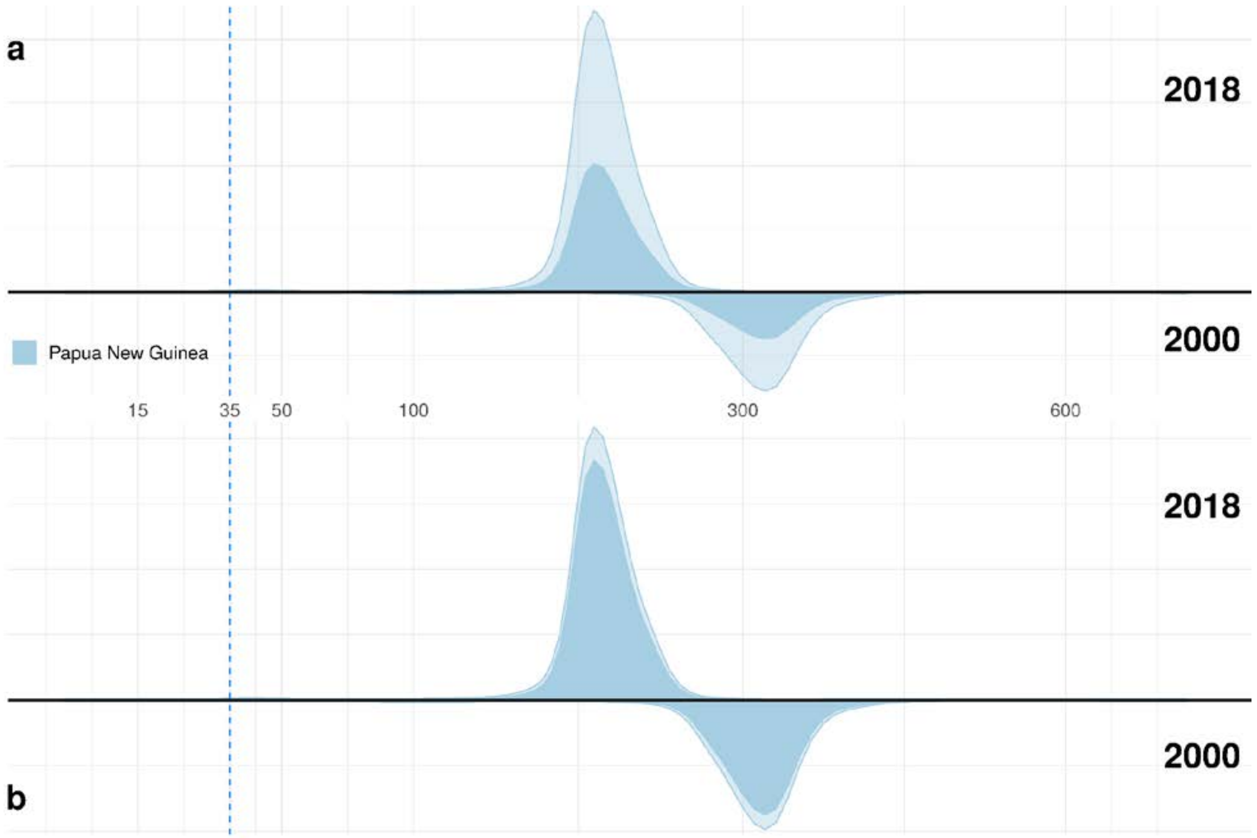

d) Central Asia

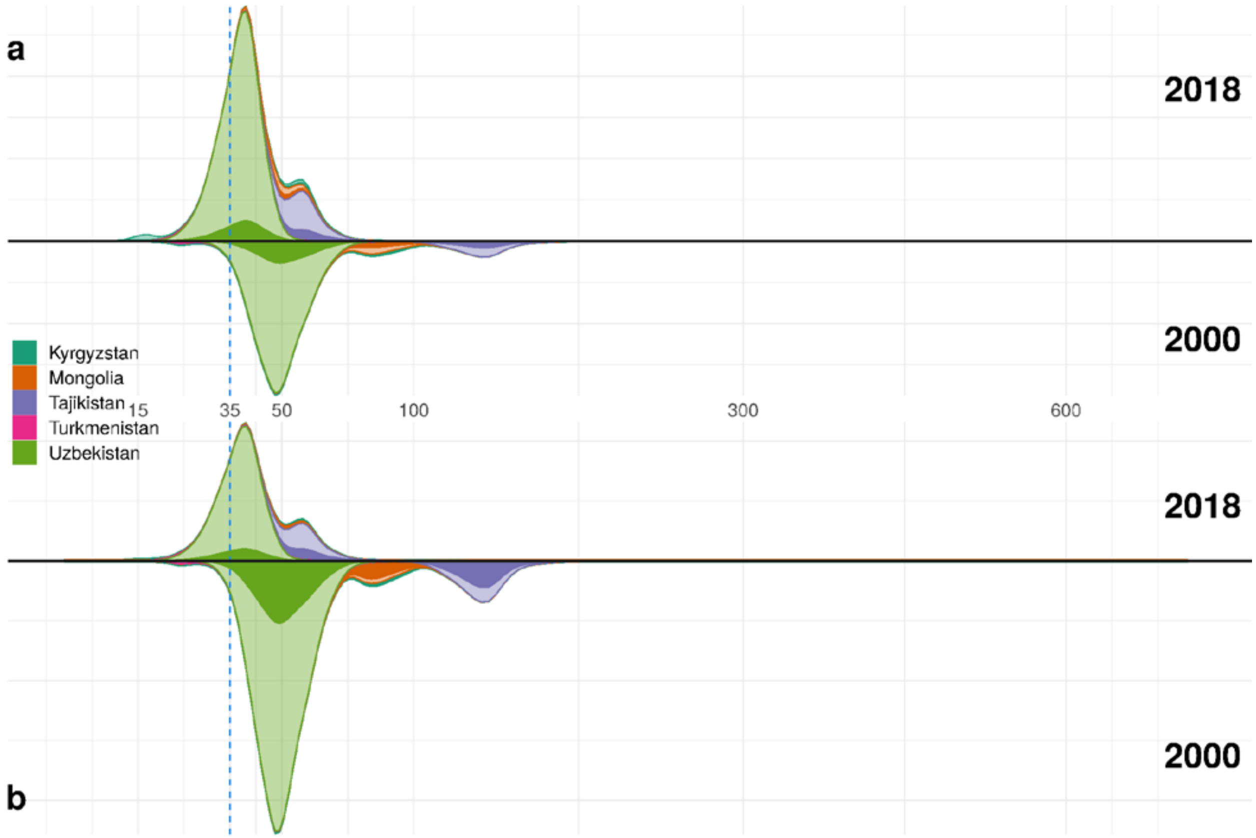

e) Caribbean

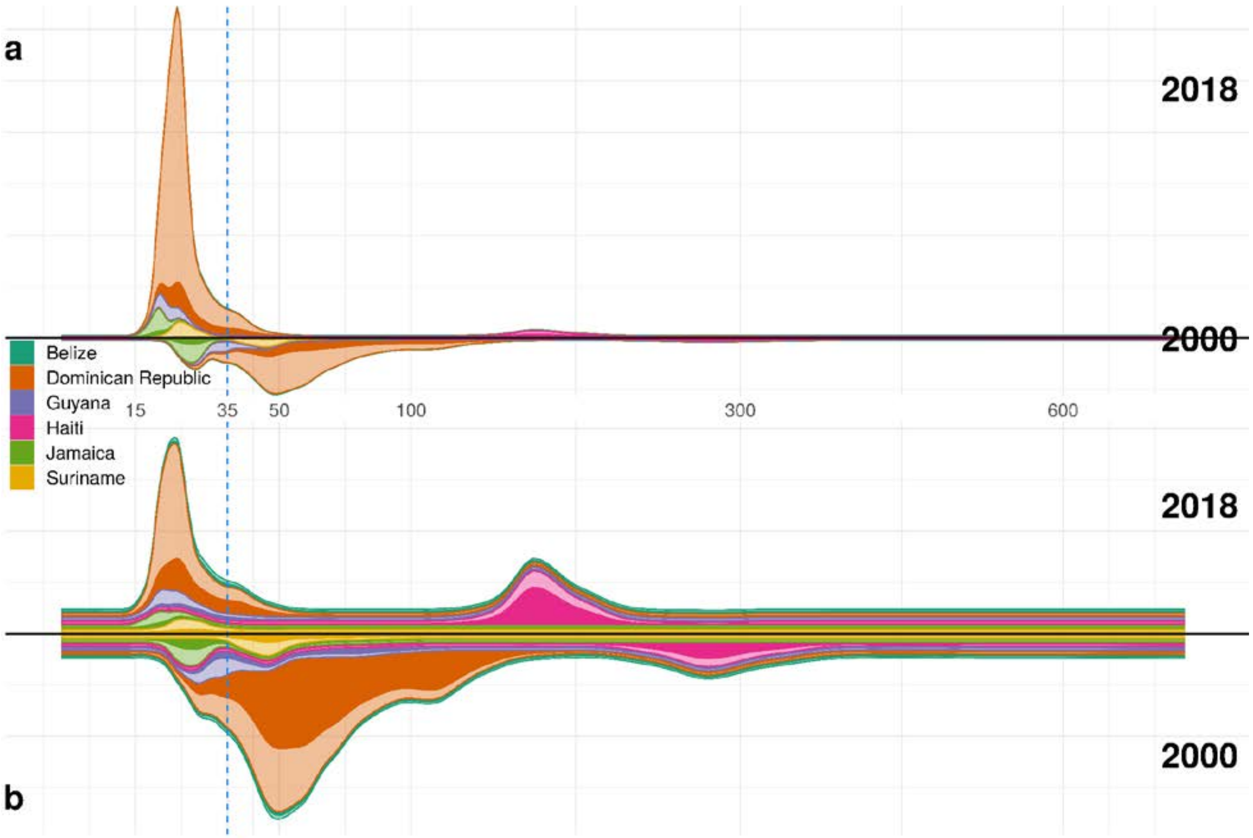
a
2018
2000
Belize
Dominican Republic
Guyana
Haiti
Jamaica
Suriname
15
35
50
100
300
600
2018
2000
b

f) Andean Latin America

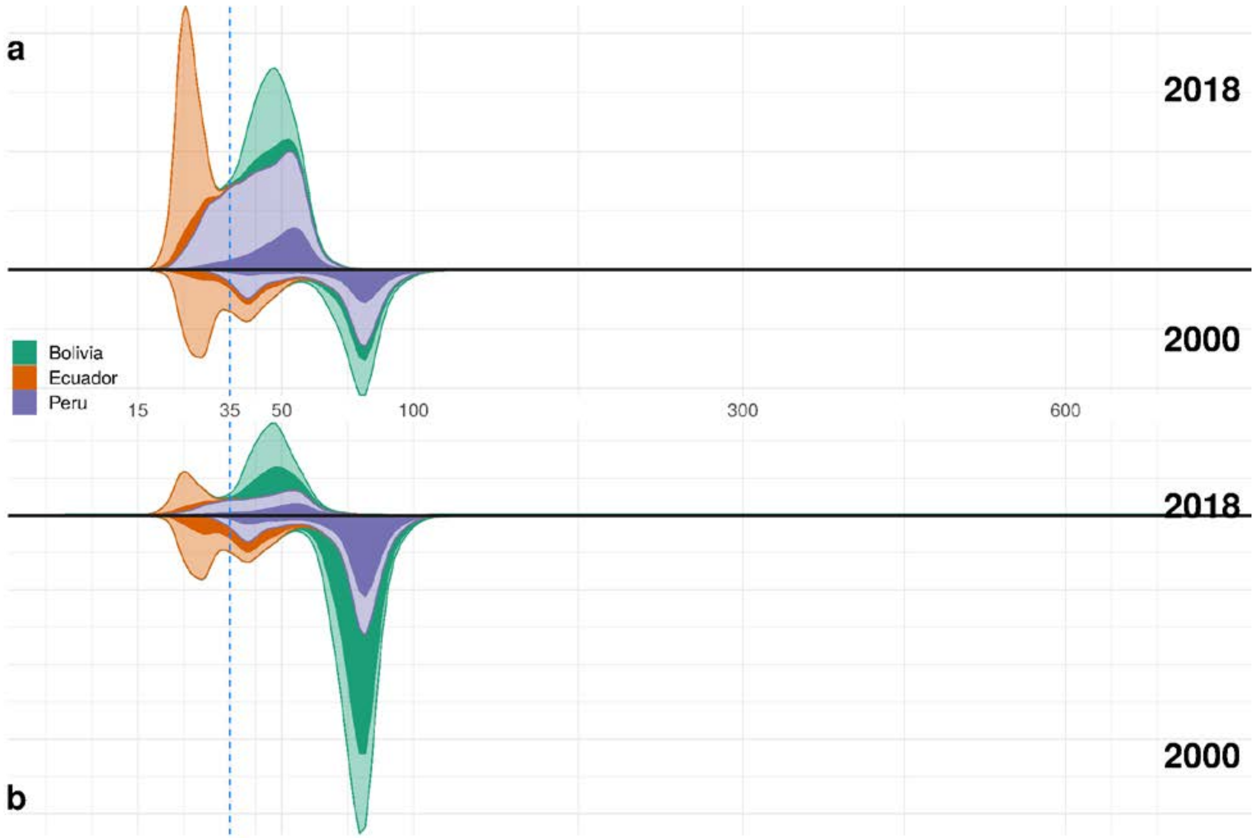

g) Central Latin America

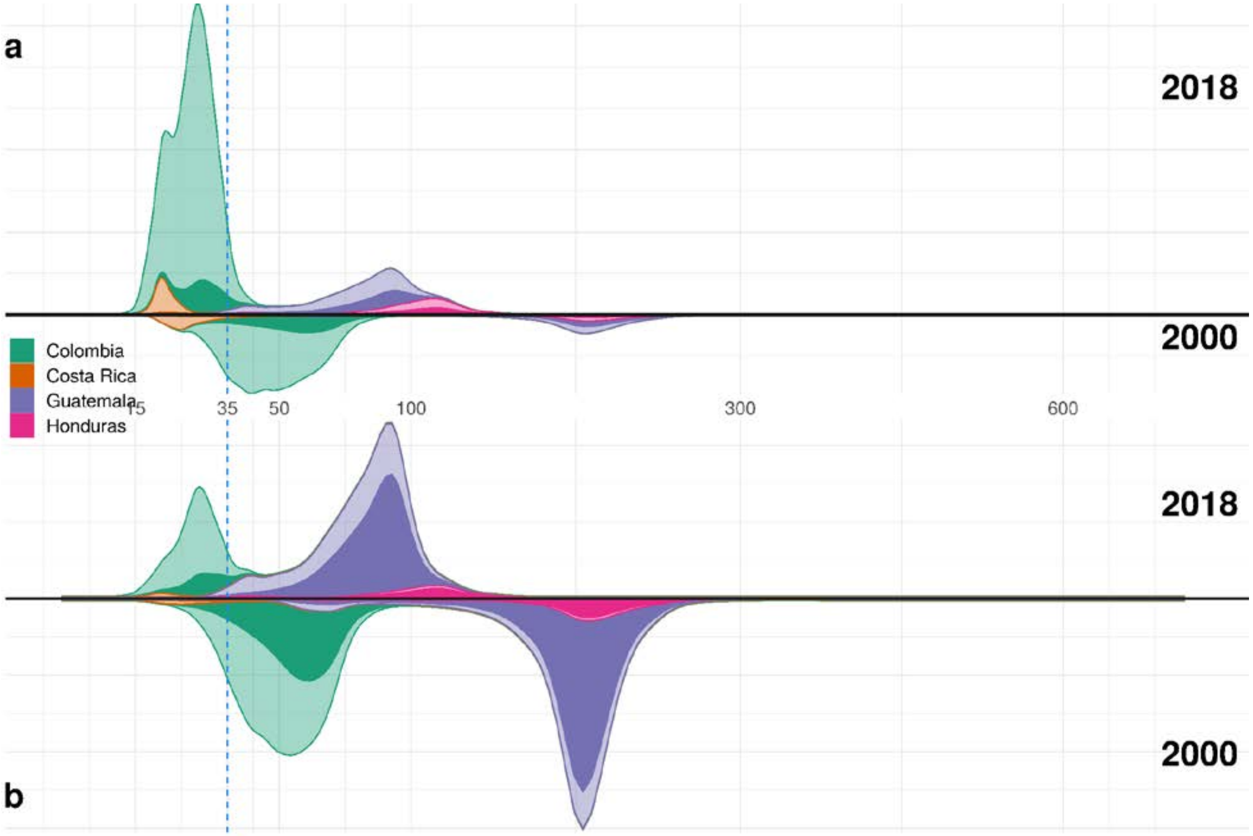

h) Central Latin America, continued

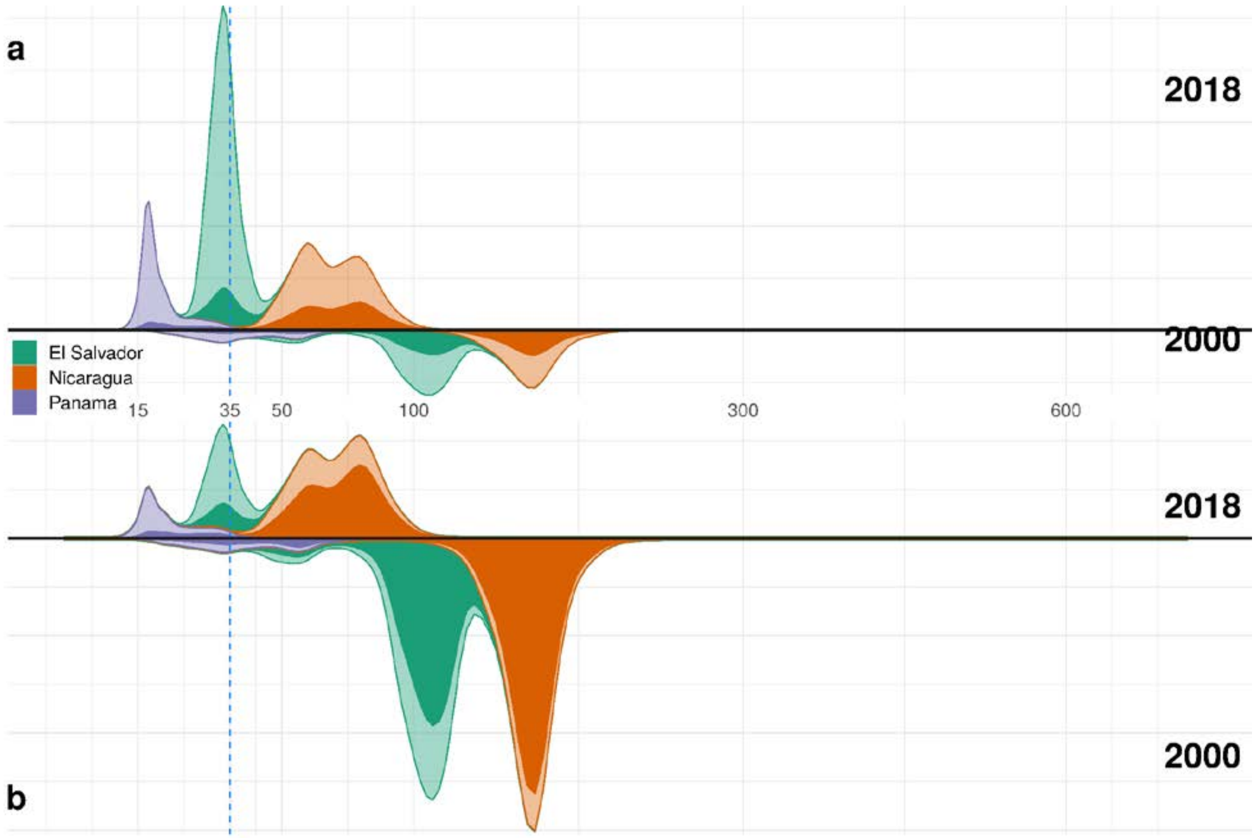

i) Tropical Latin America

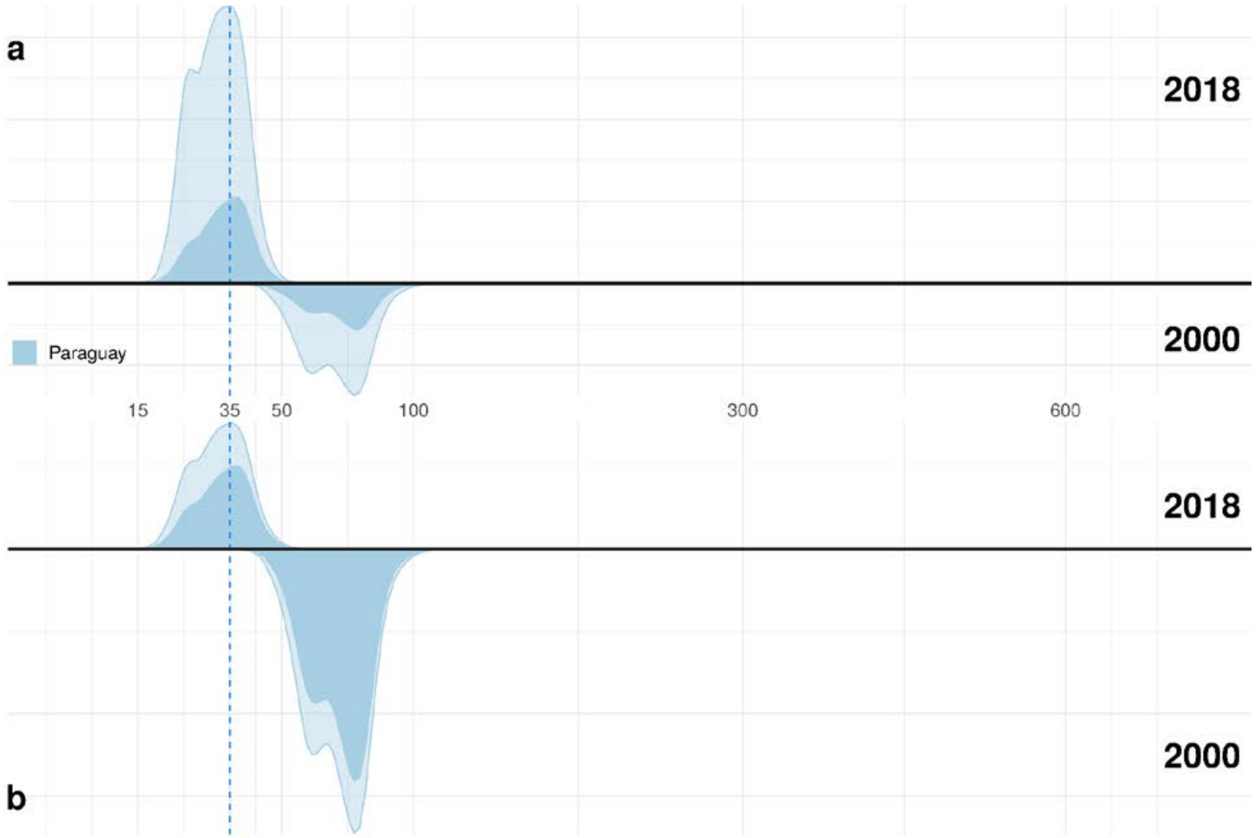

j) North Africa and Middle East

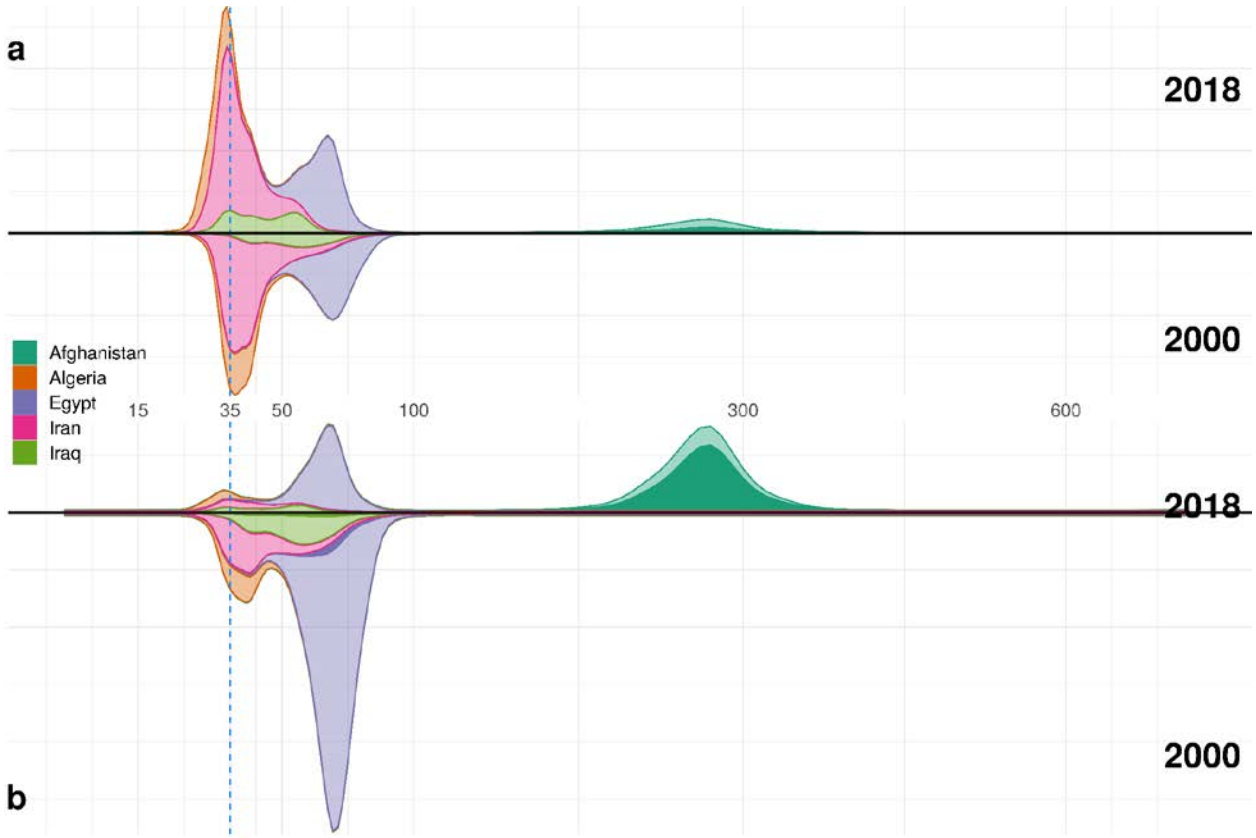

k) North Africa and Middle East

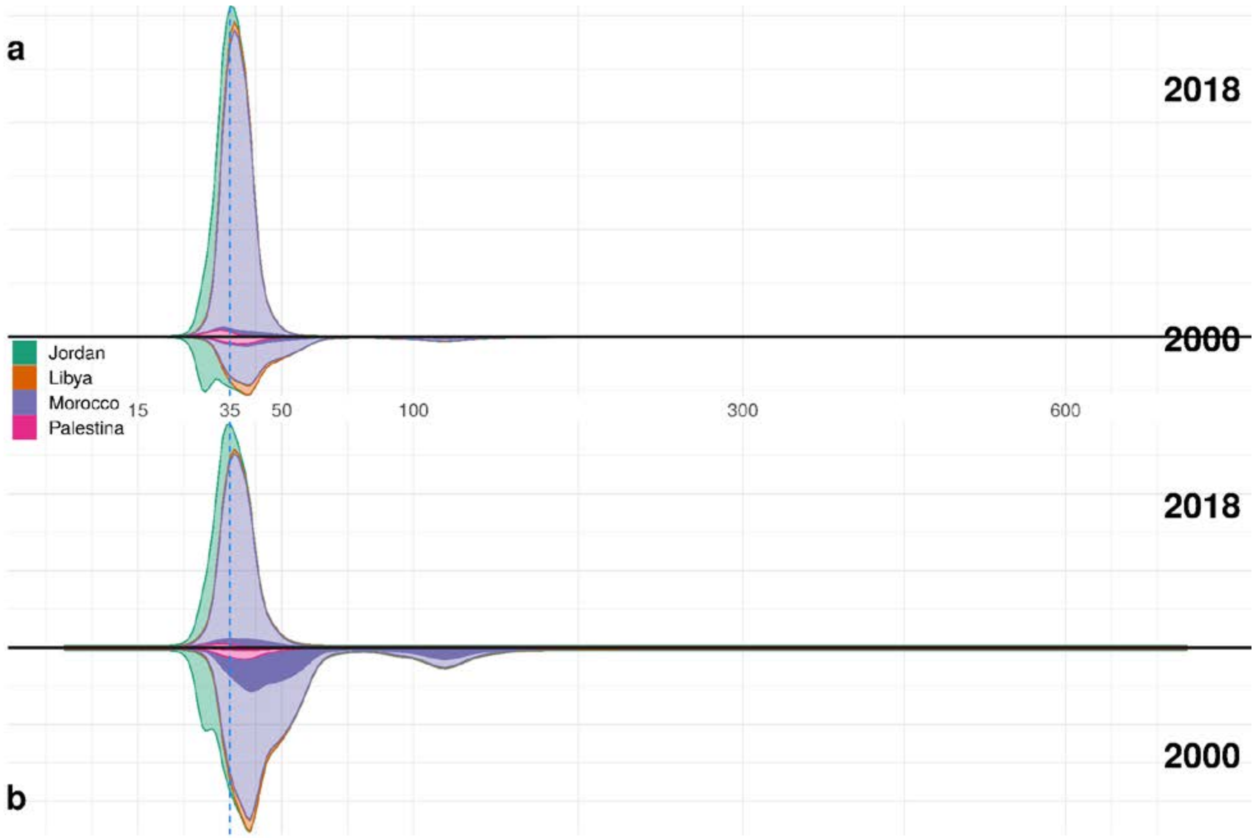

l) North Africa and Middle East, continued

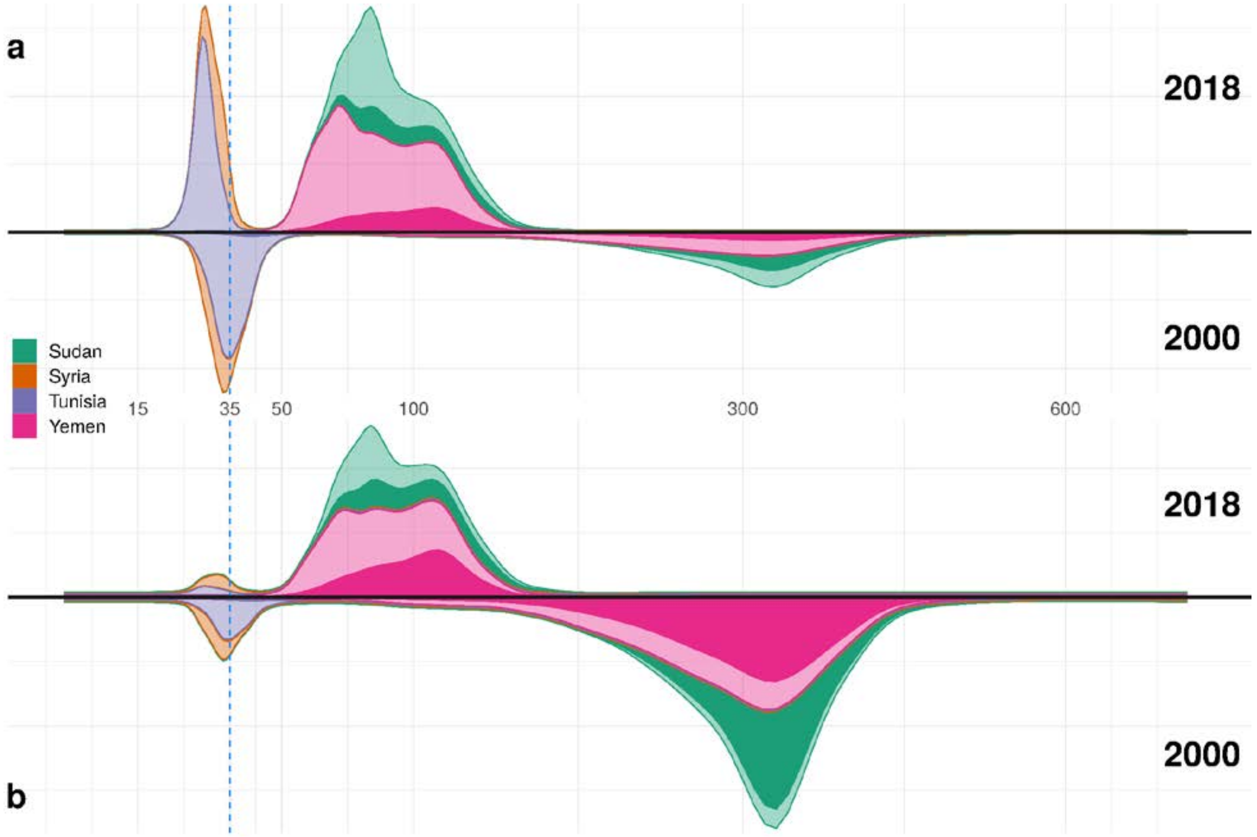

m) South Asia

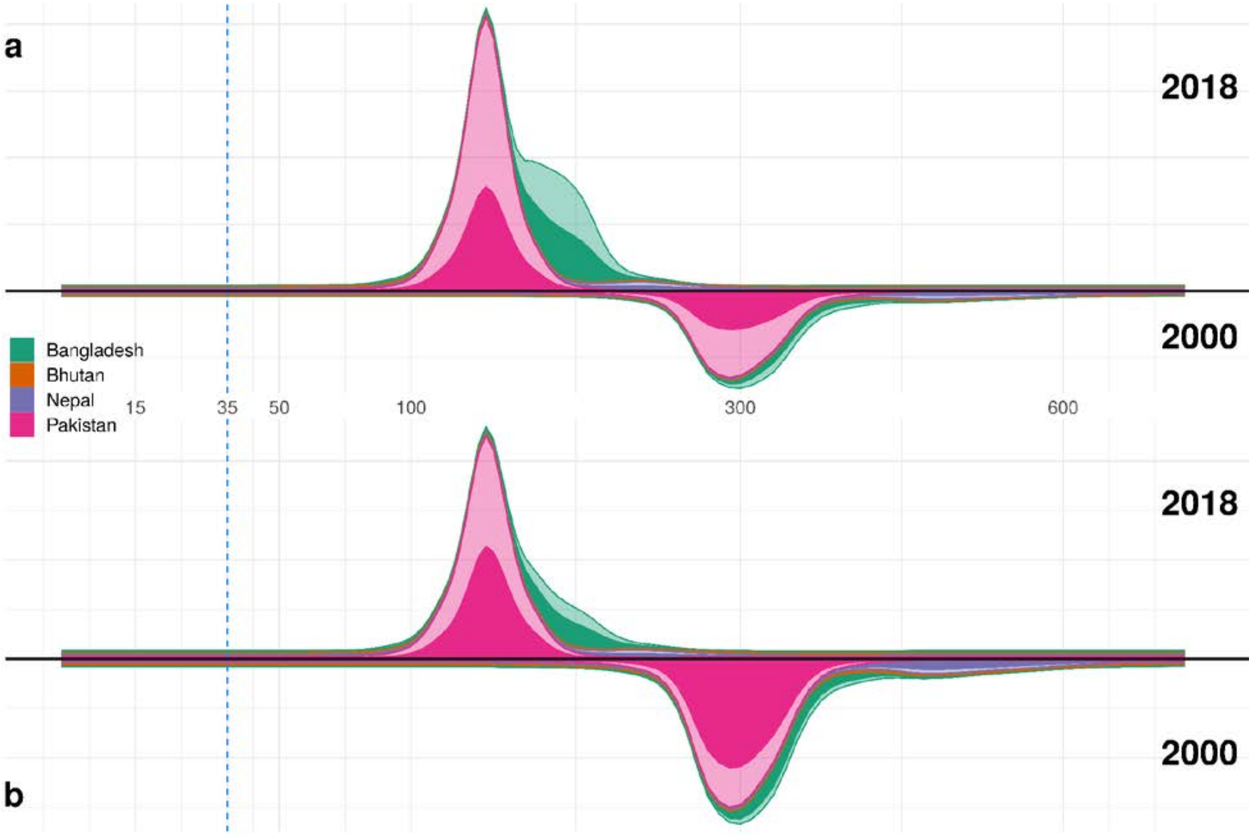

a
2018
2000
Bangladesh
Bhutan
Nepal
Pakistan
15
35
50
100
300
600
2018
2000
b

n) Central Sub-Saharan Africa

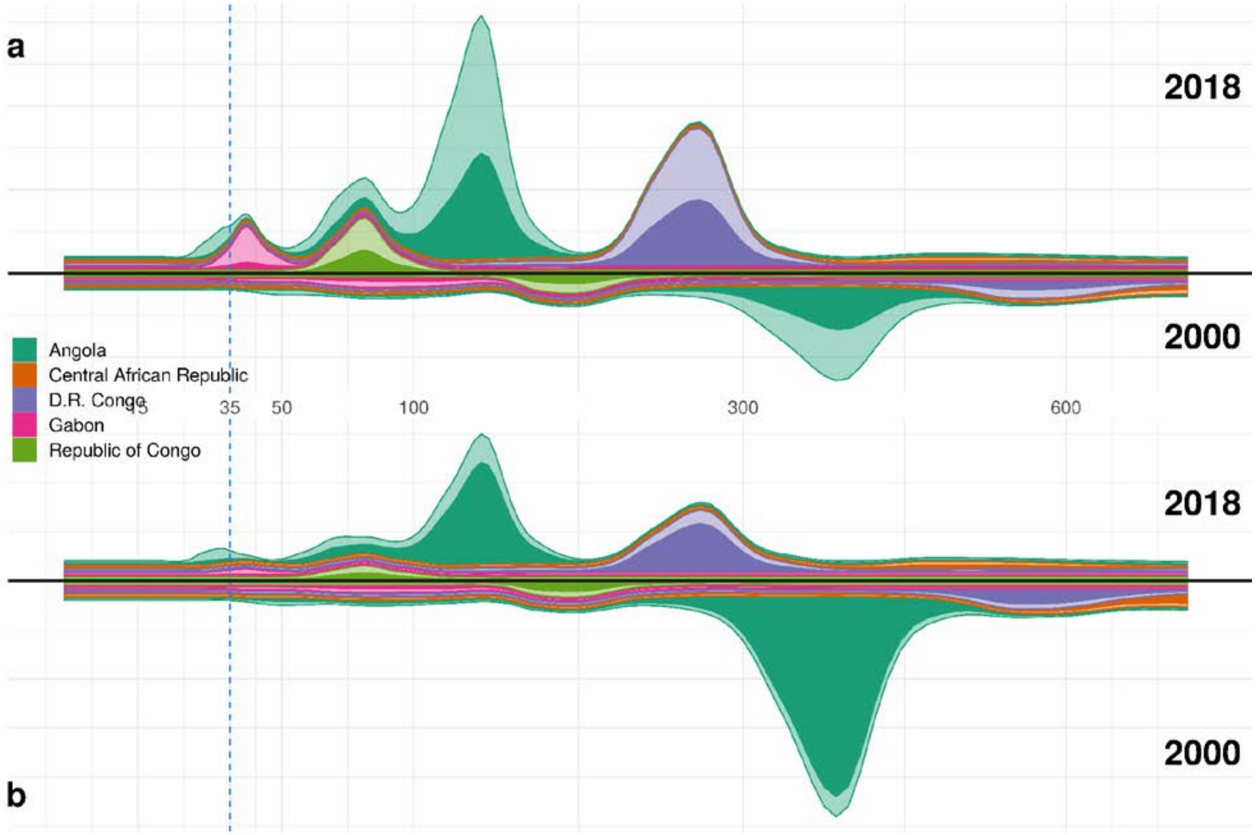

o) Eastern Sub-Saharan Africa

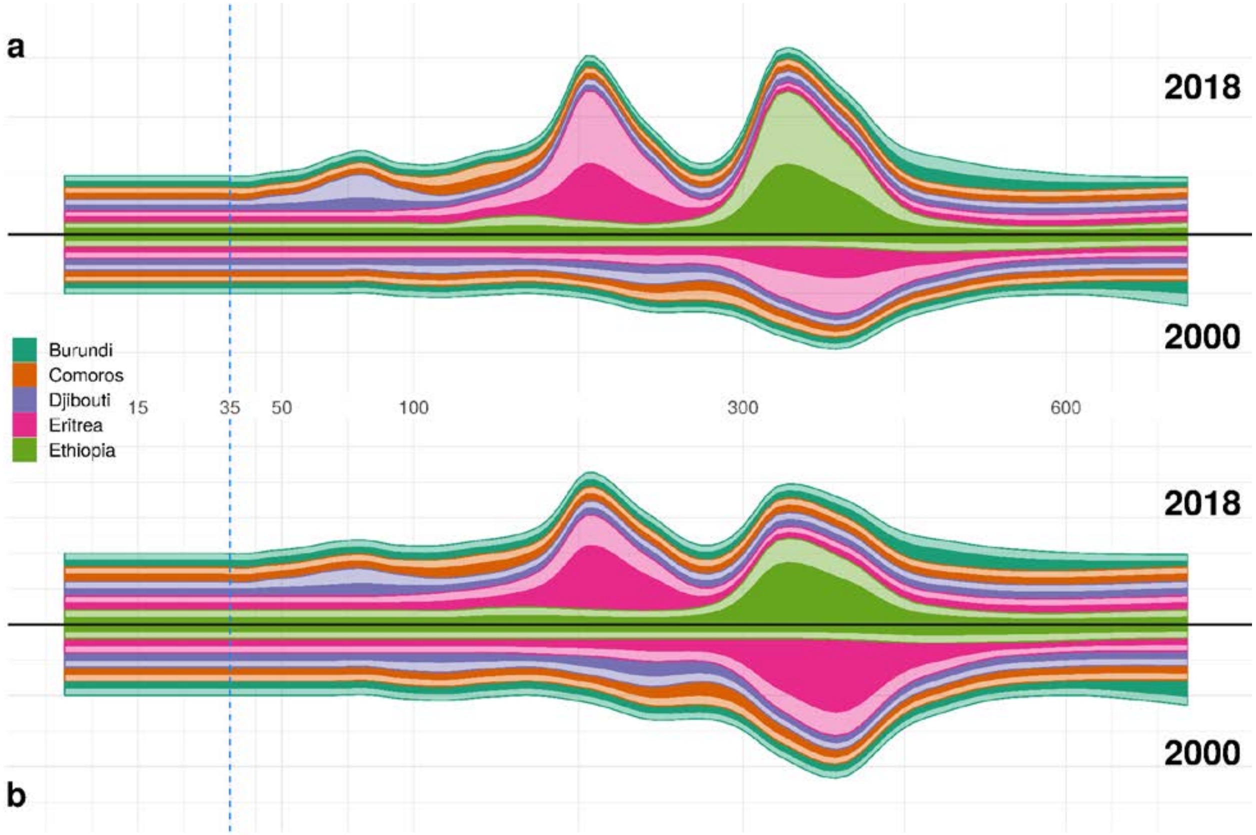

p) Eastern Sub-Saharan Africa, continued

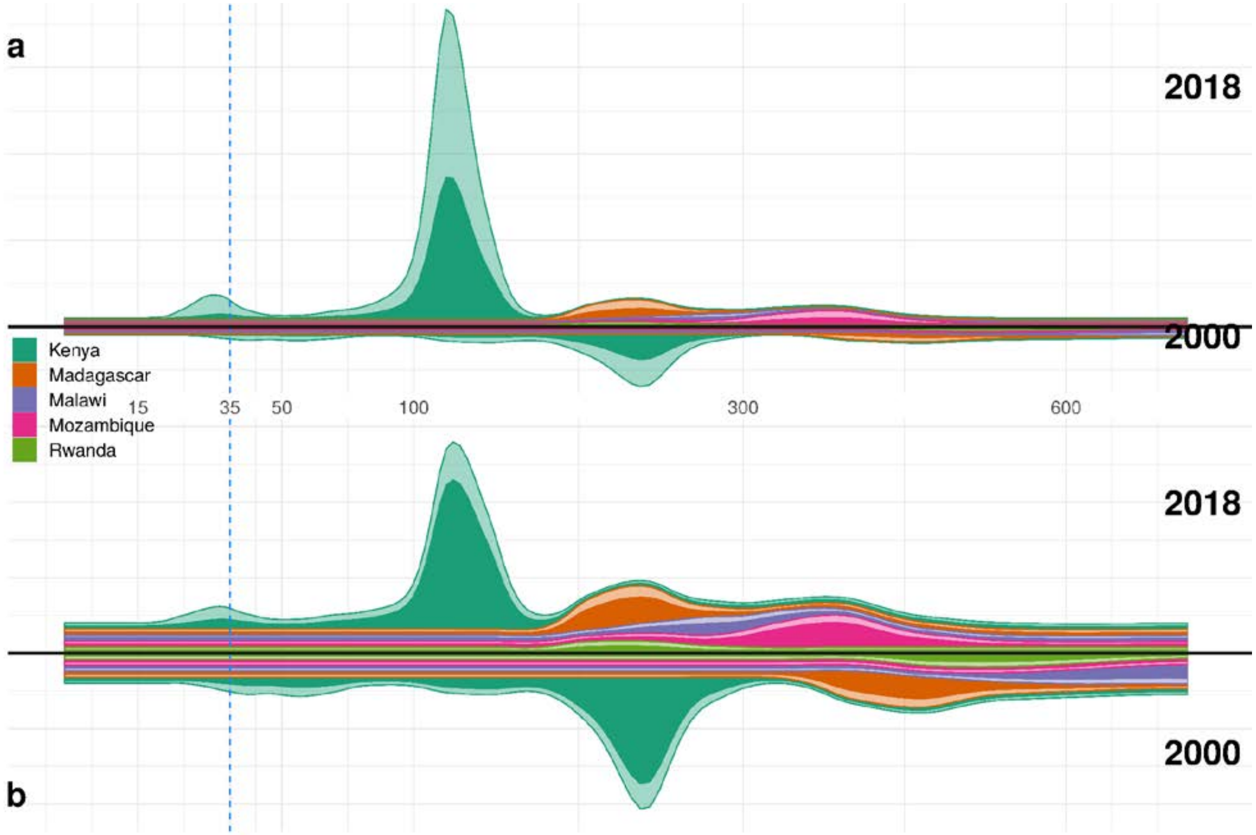

q) Eastern Sub-Saharan Africa, Continued

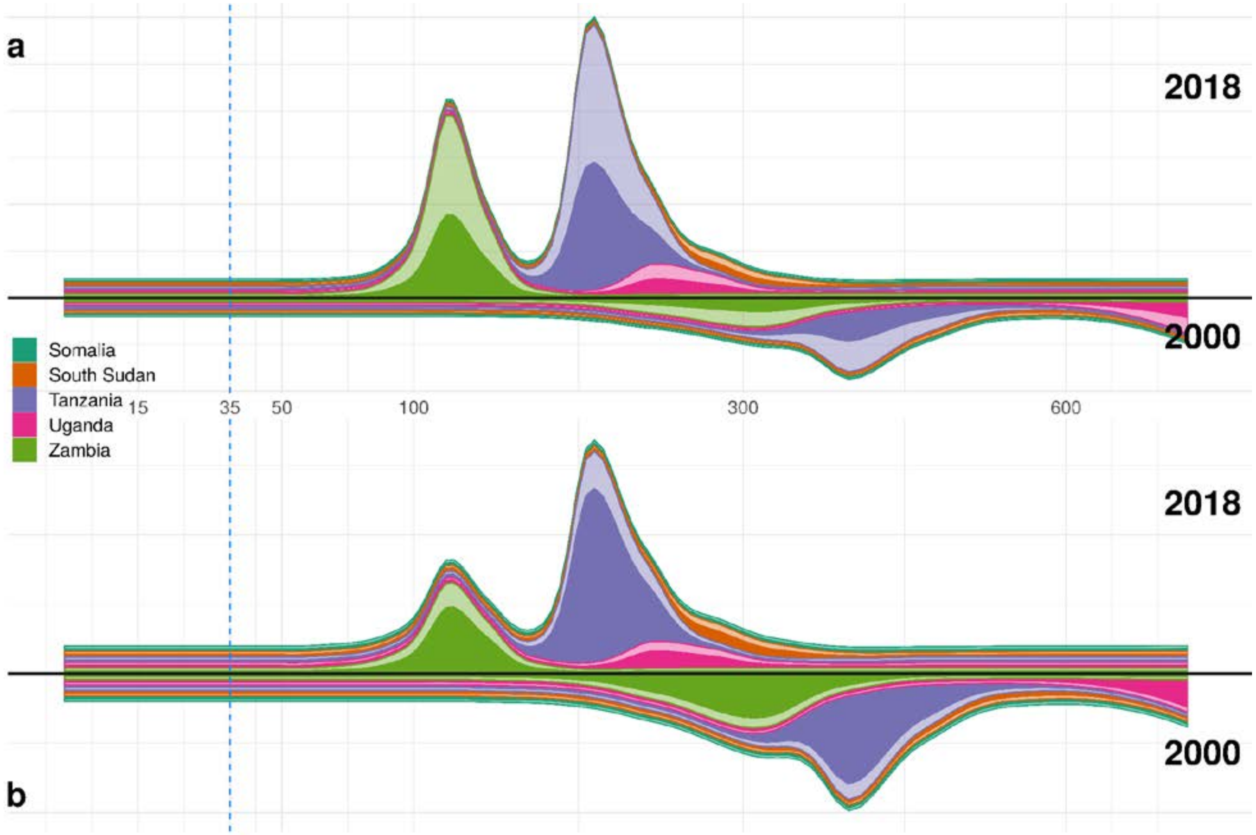

r) Southern Sub-Saharan Africa

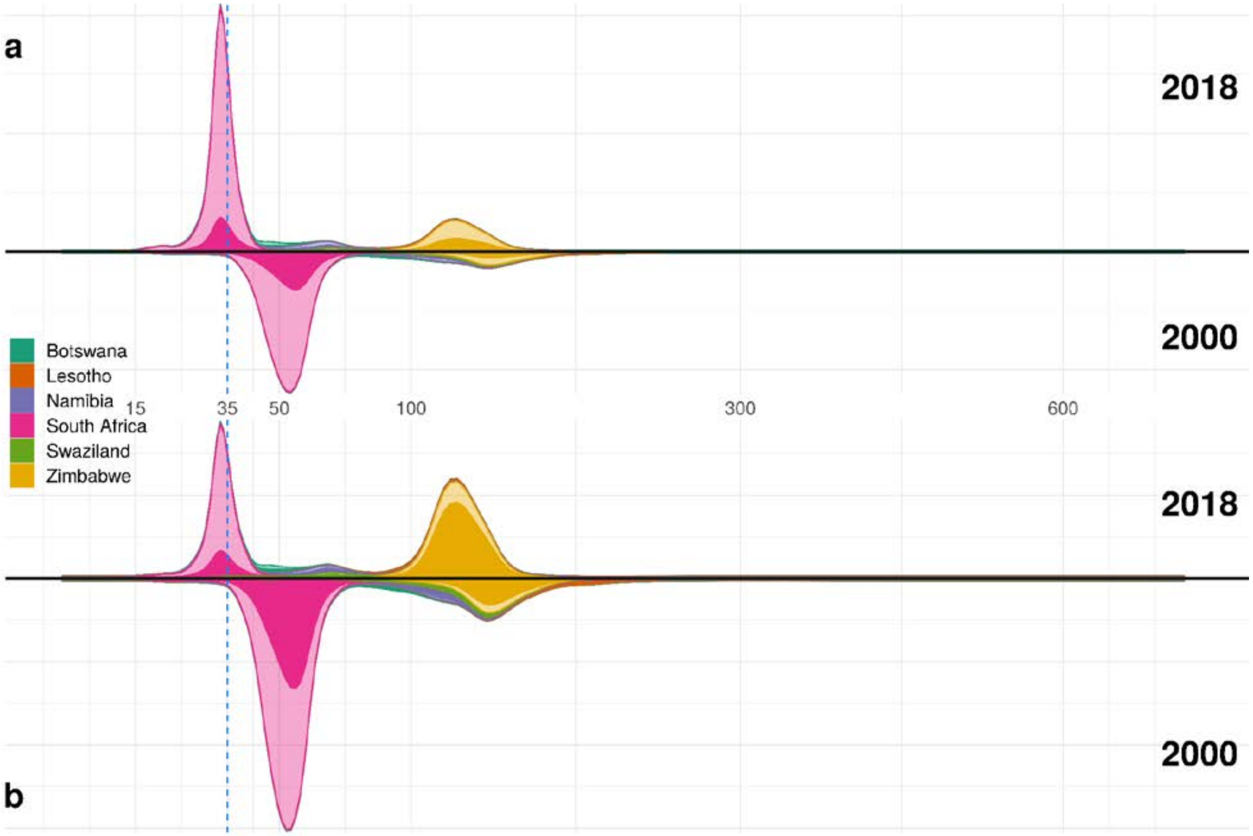

s) Western Sub-Saharan Africa

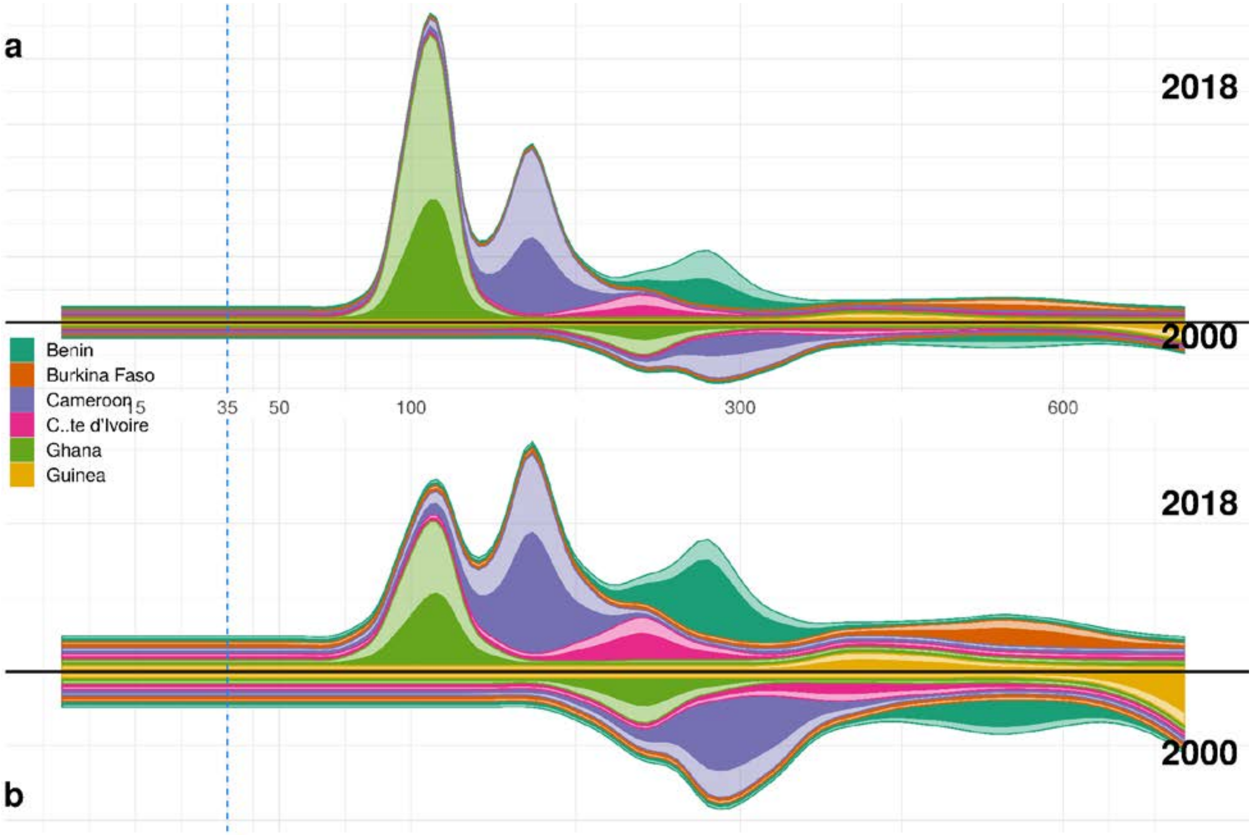

t) Western Sub-Saharan Africa, Continued

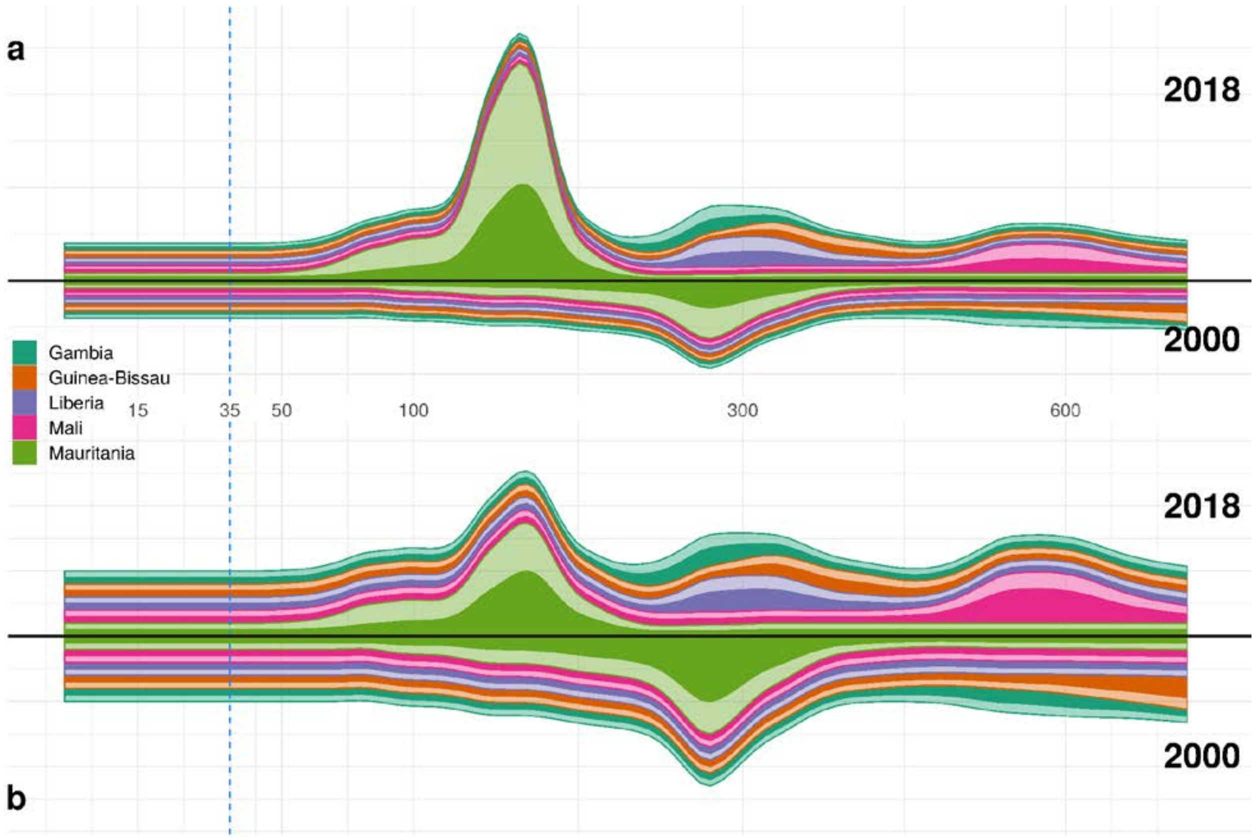

u) Western Sub-Saharan Africa, Continued

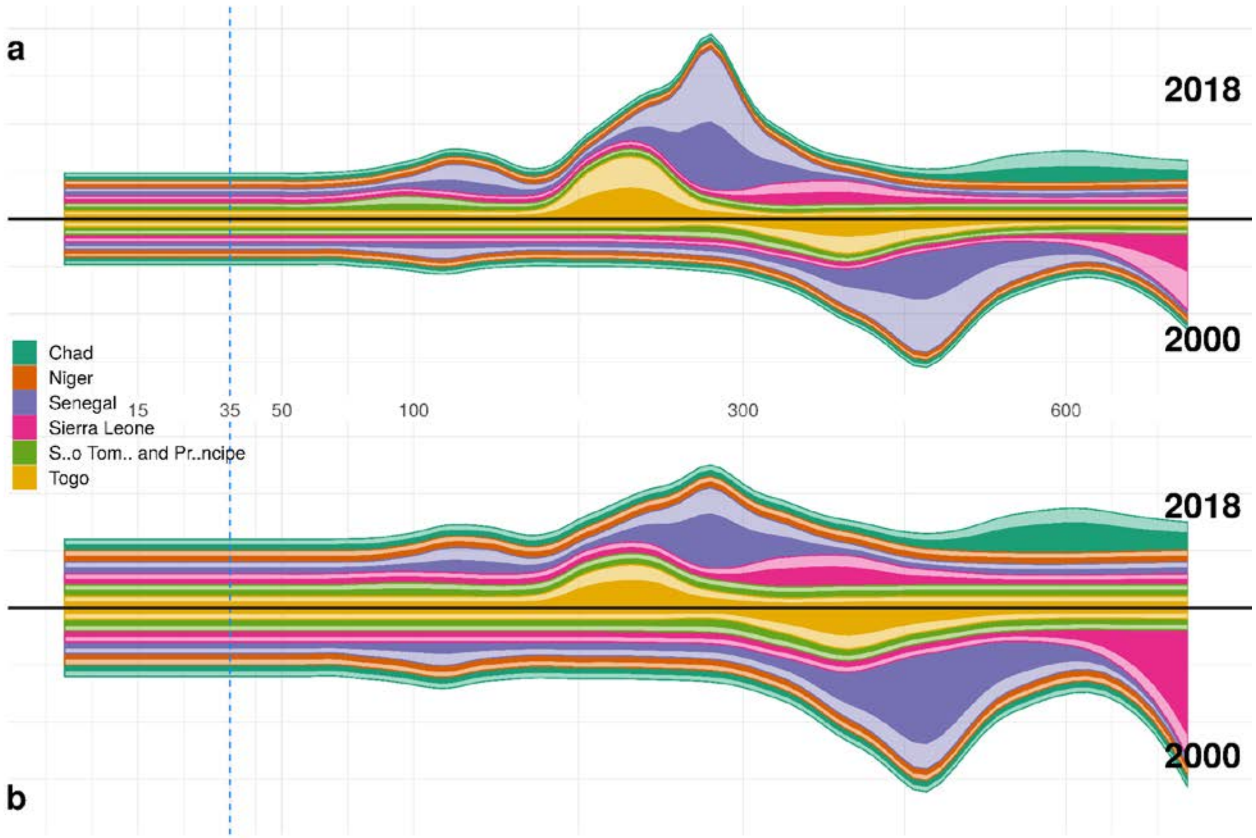

Supplementary Figure 18a-v: Air pollution risk transition (2000–2018) by region.

a. Trends in the percent of LRIs attributed to TAP at the second administrative unit (districts). The x-axis displays the percent of total air pollution exposure that is contributed by ambient air pollution. The blue and red background shading indicate the dominant source of air pollution is household or ambient sources. The y-axis displays the fraction of under-5 LRIs that are attributable to TAP, as estimated by the population attributable fraction (PAF). The y-axis rugs indicate the gradient of background LRI mortality rates for 2000 (left) and 2018 (right), illustrating the correlation between LRI rates and the fraction attributable to TAP. The lines connect a district to its preceding time point across the series.

a) East Asia

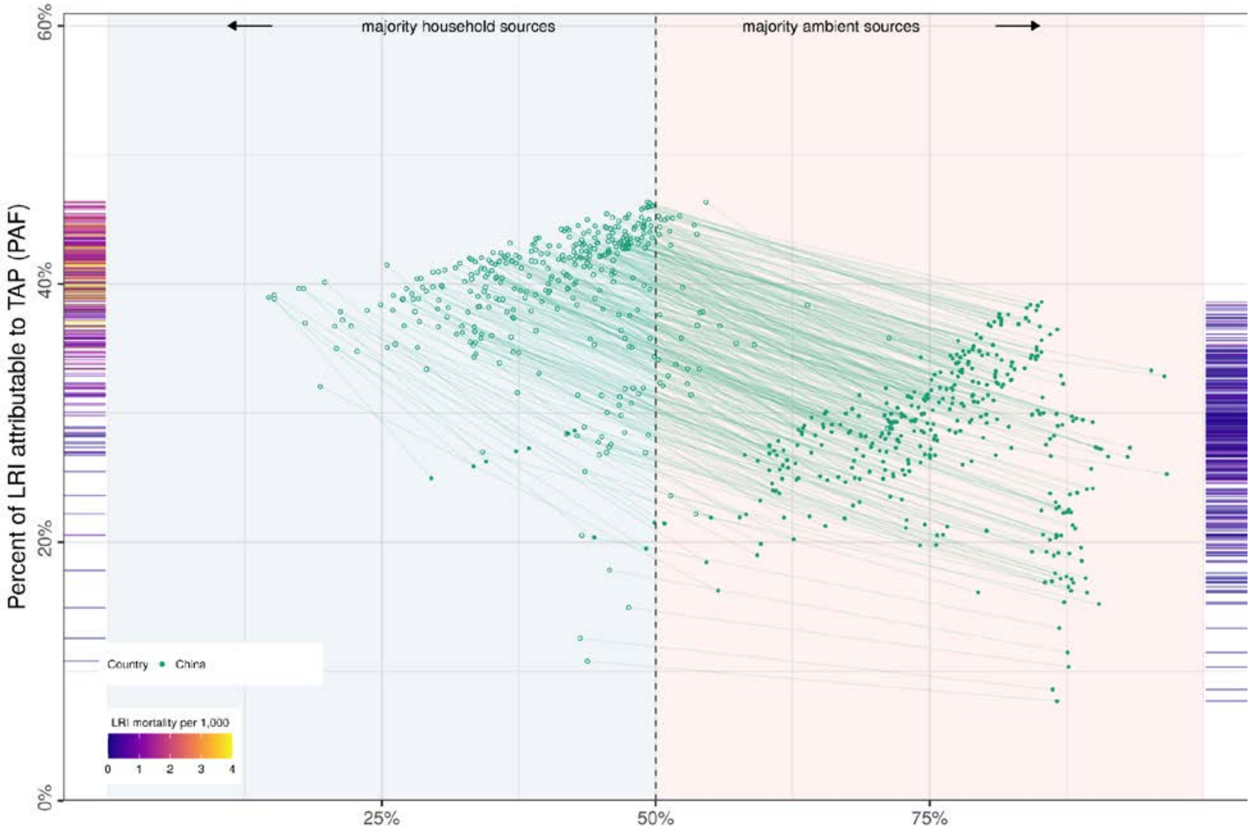

b) Southeast Asia

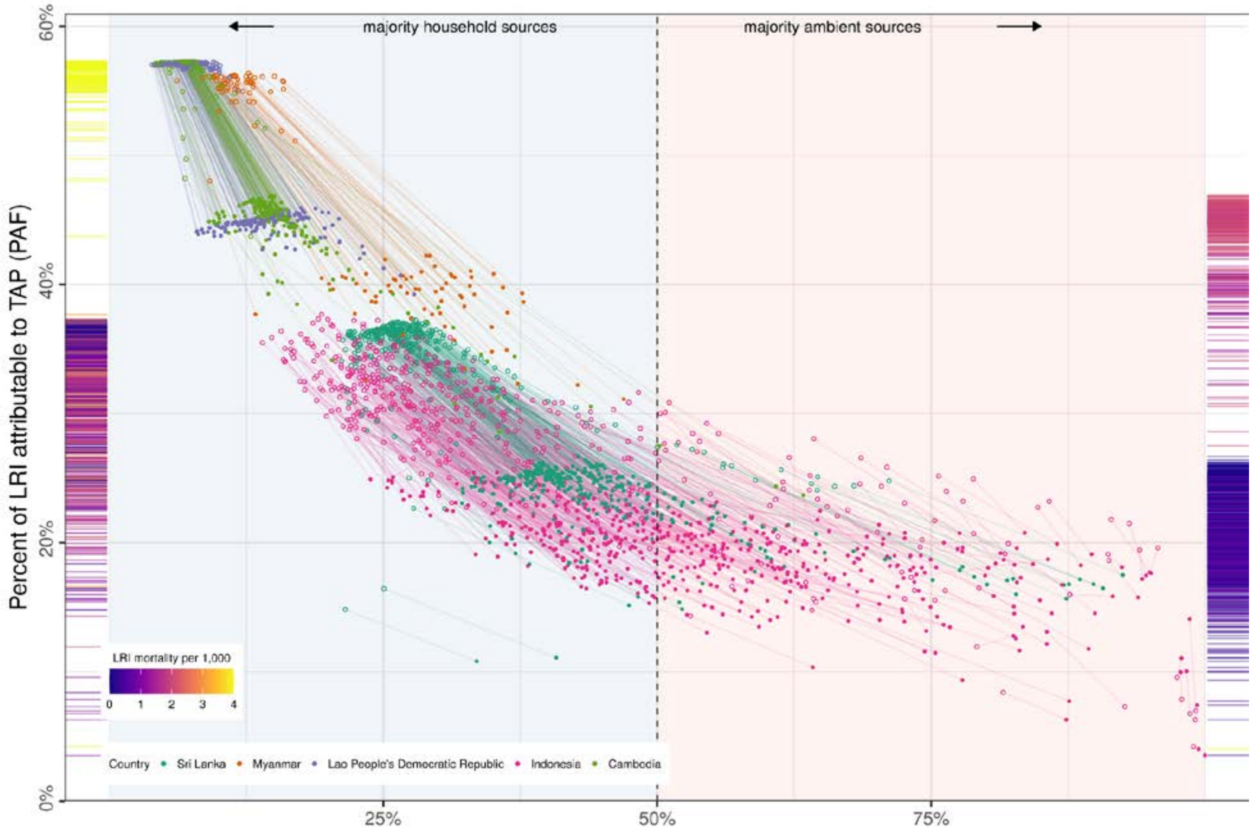

c) Southeast Asia, continued

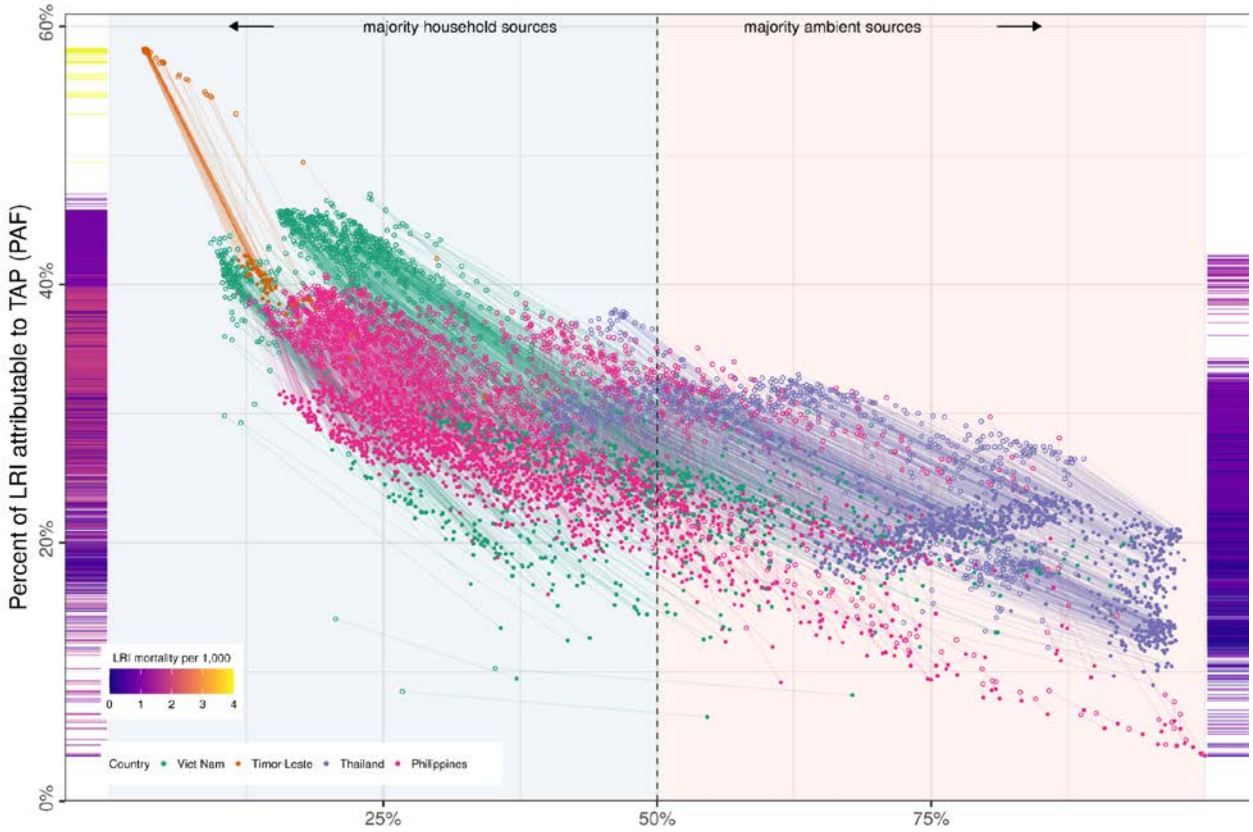

d) Oceania

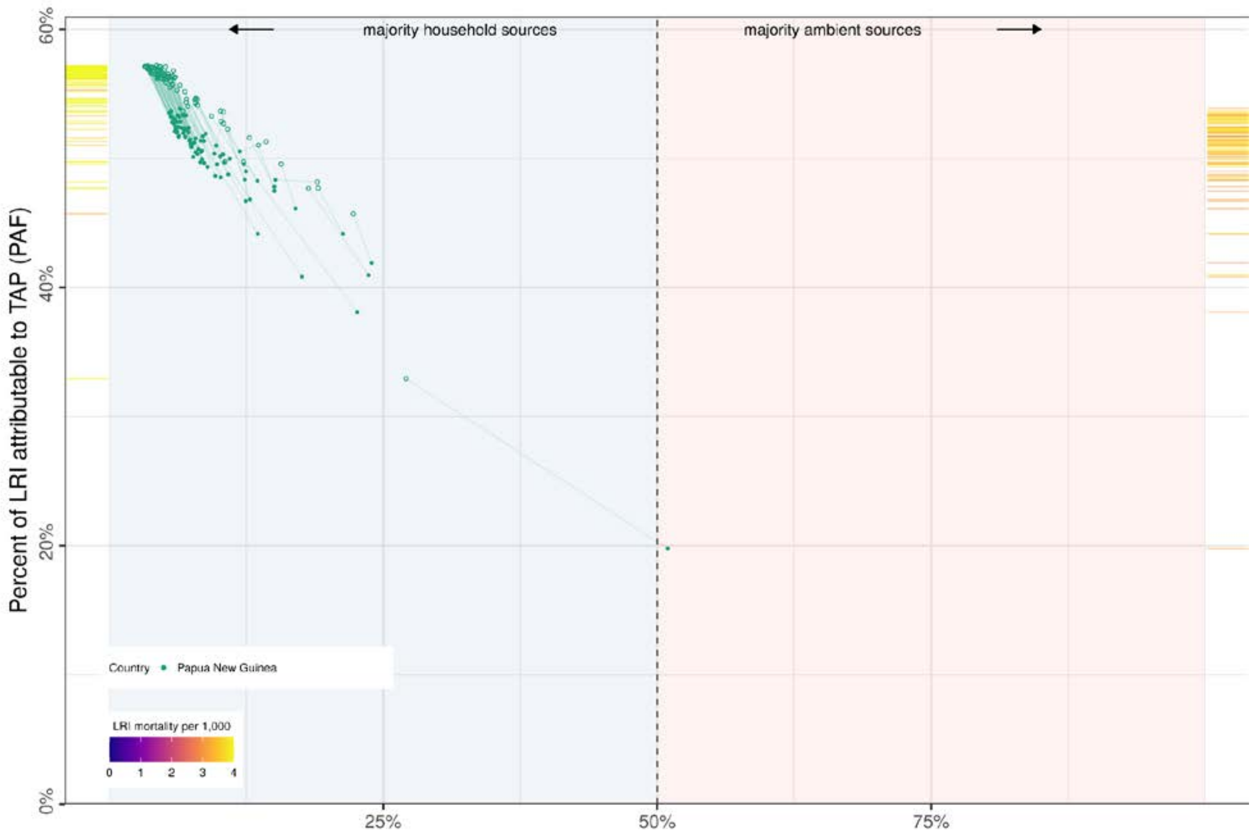
majority household sources
majority ambient sources
Percent of LRI attributable to TAP (PAF)
60%
40%
20%
0%
25%
50%
75%
Country
Papua New Guinea
LRI mortality per 1,000
0
1
2
3
4

e) Central Asia

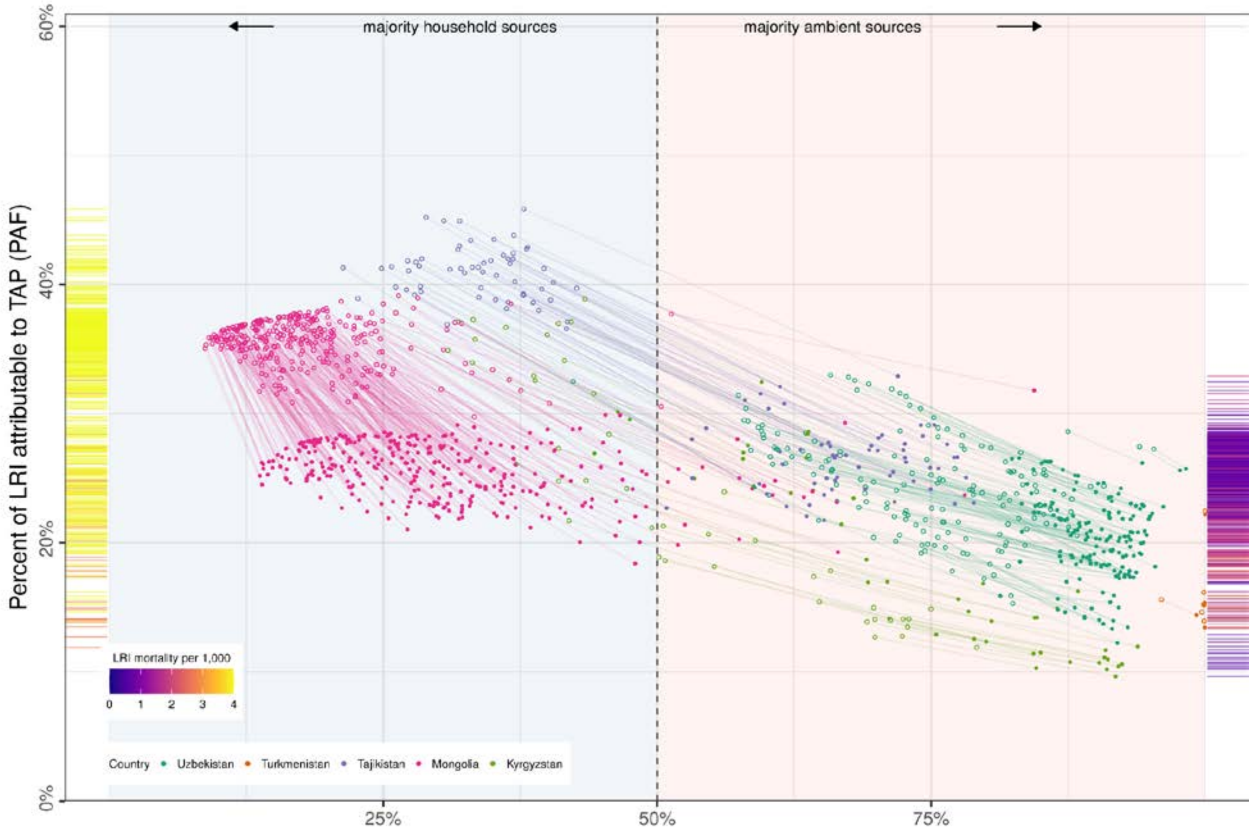

f) Caribbean

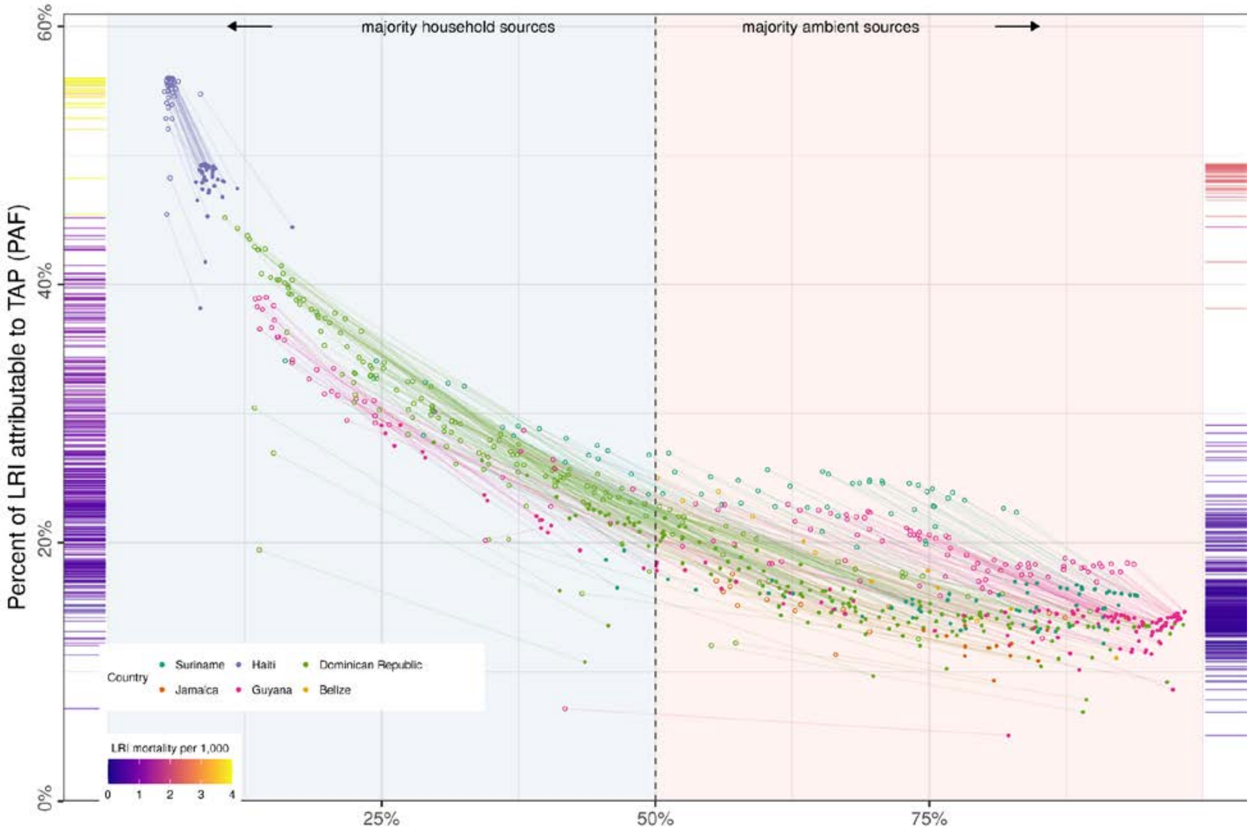

majority household sources
majority ambient sources
Percent of LRI attributable to TAP (PAF)
60%
40%
20%
0%
25%
50%
75%
Country
Suriname
Haiti
Dominican Republic
Jamaica
Guyana
Belize
LRI mortality per 1,000
0
1
2
3
4

g) Andean Latin America

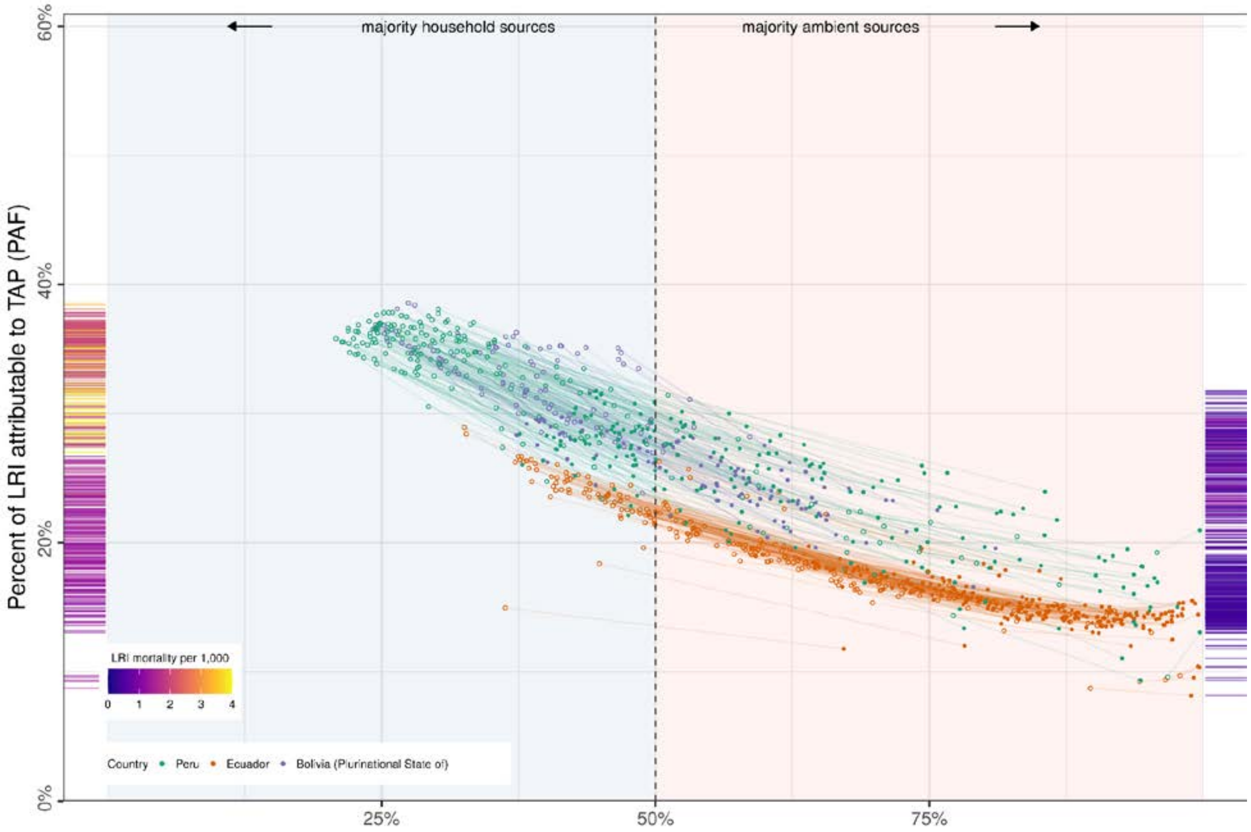

h) Central Latin America

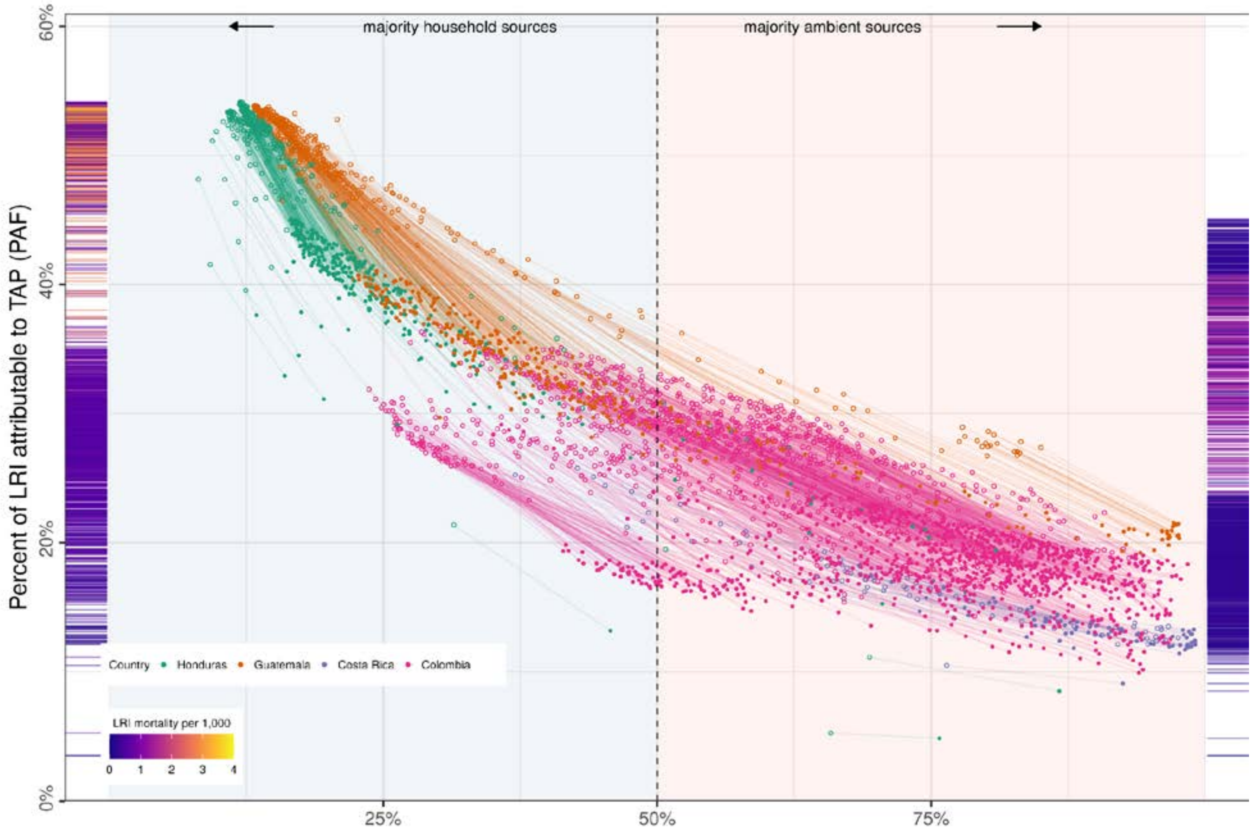

i) Central Latin America, continued

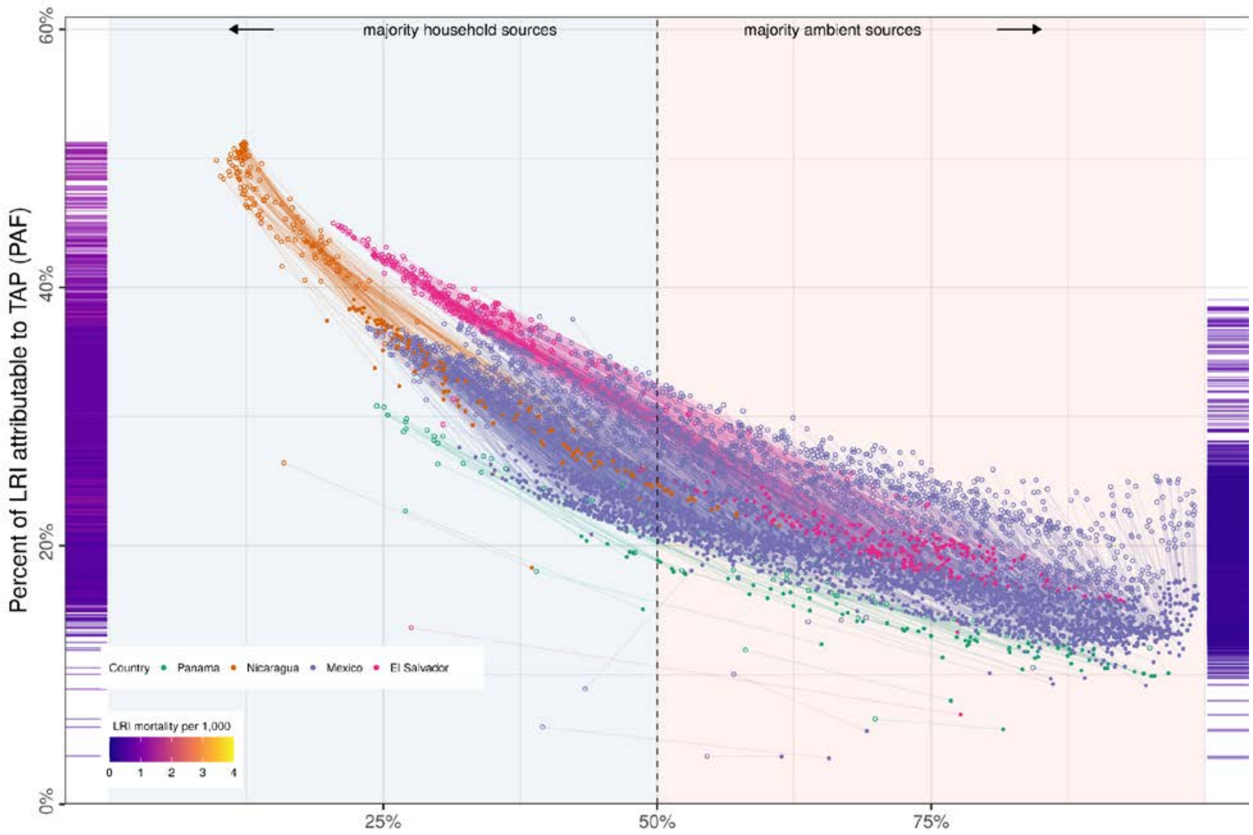

j) Tropical Latin America

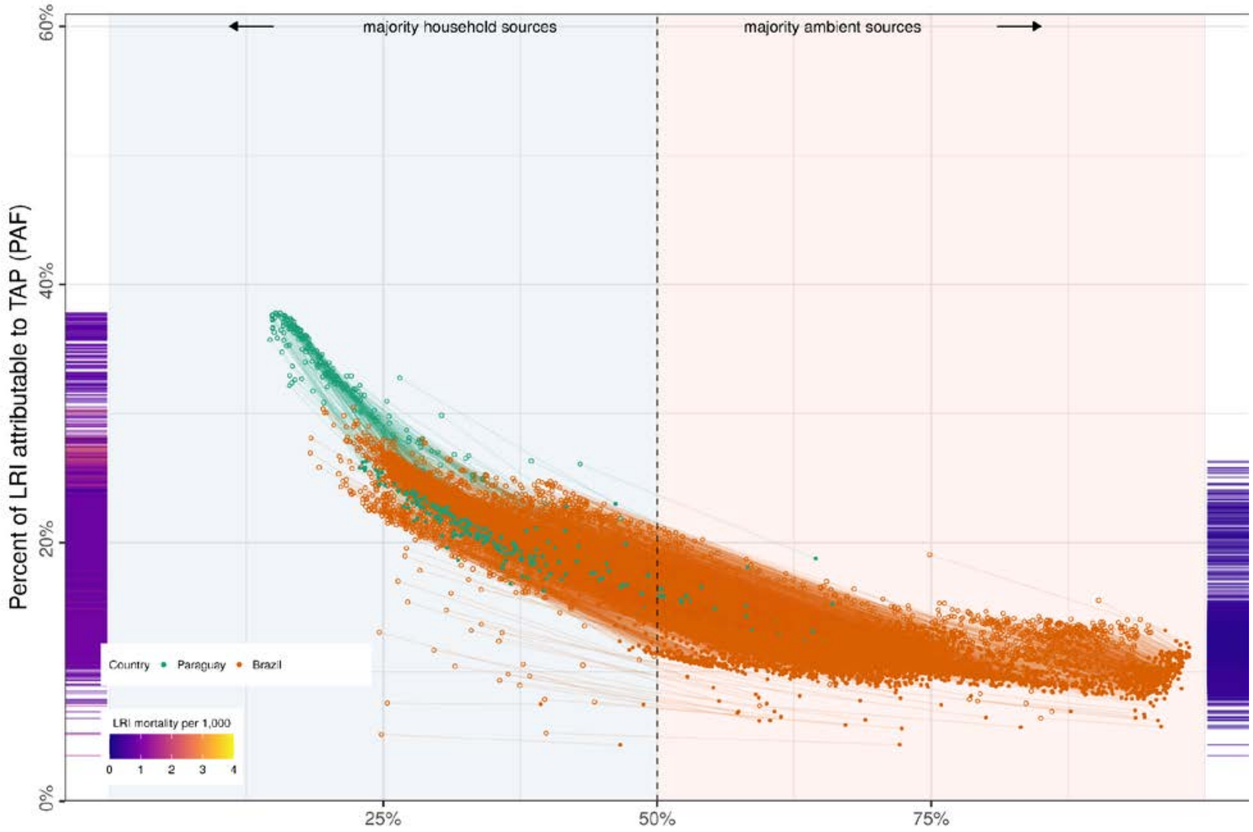

k) North Africa and Middle East

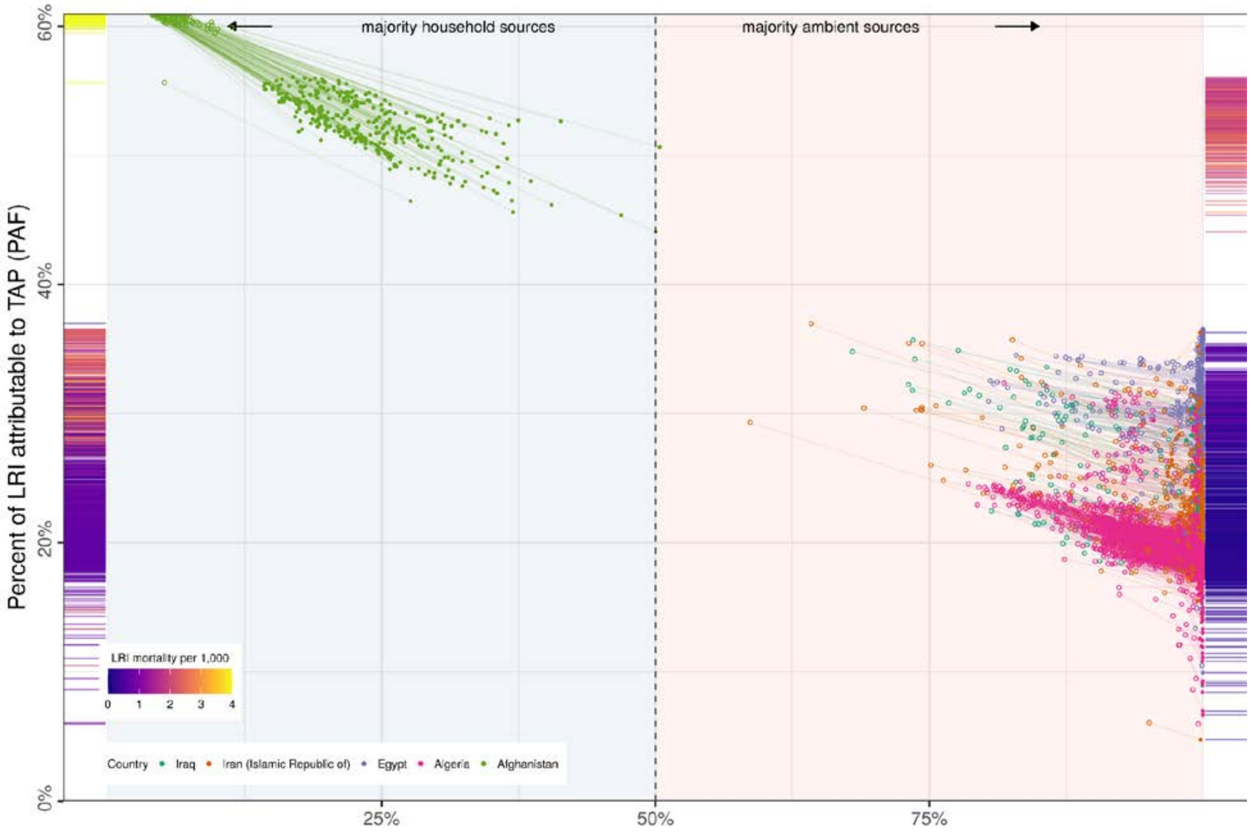

l) North Africa and Middle East

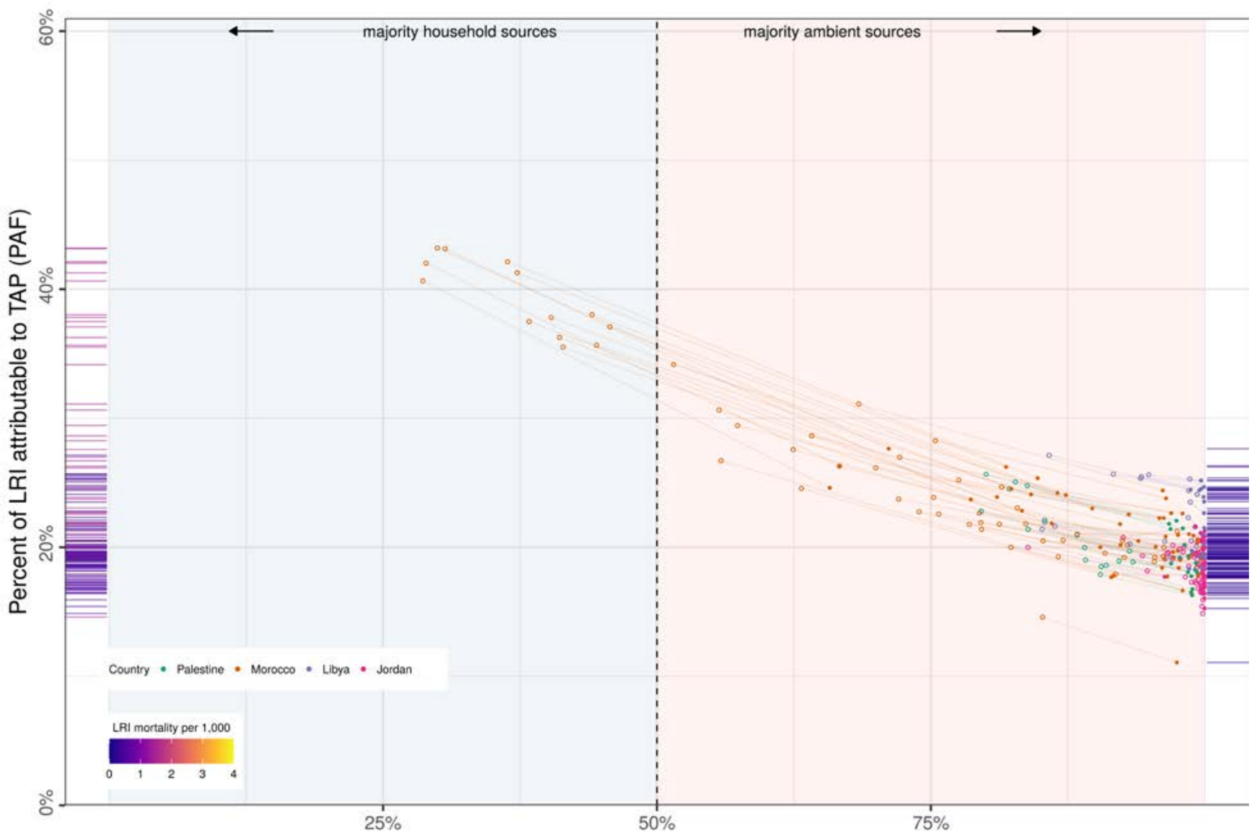

m) North Africa and Middle East, continued

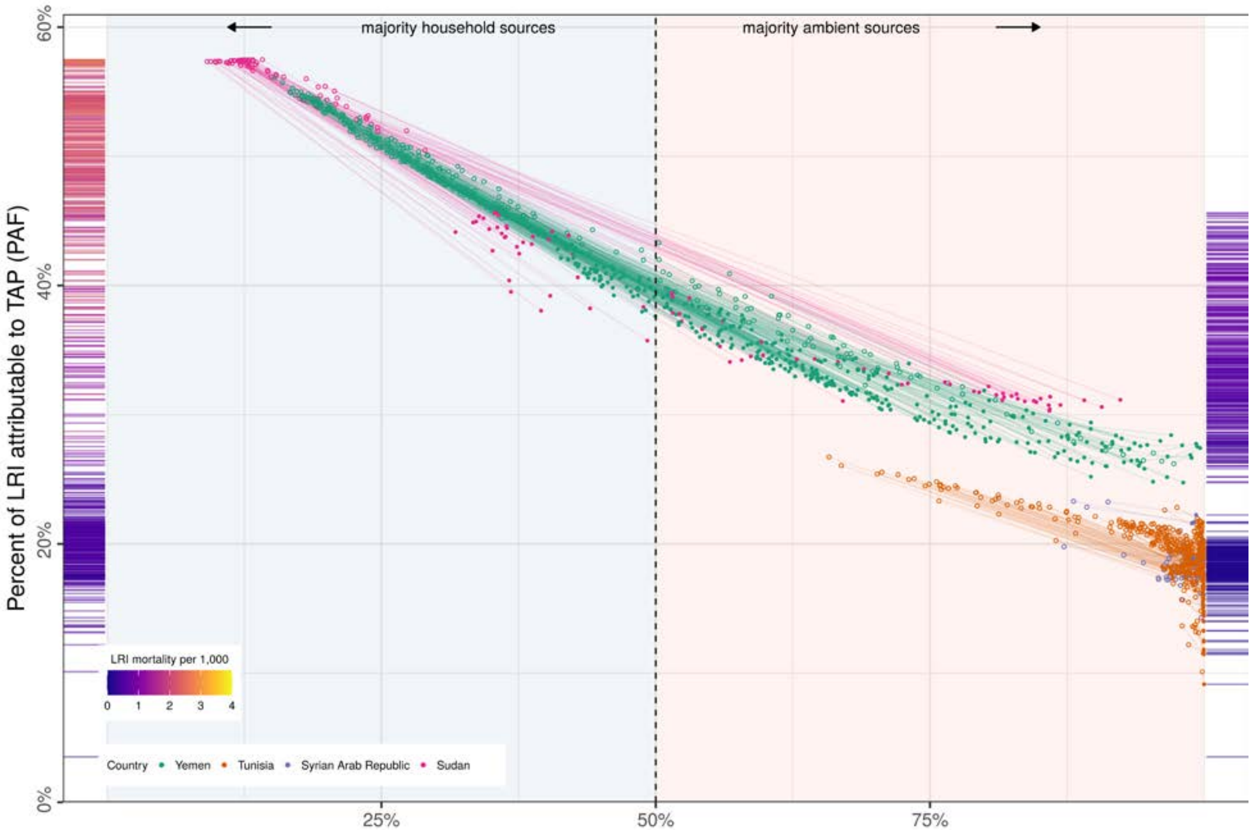

n) South Asia

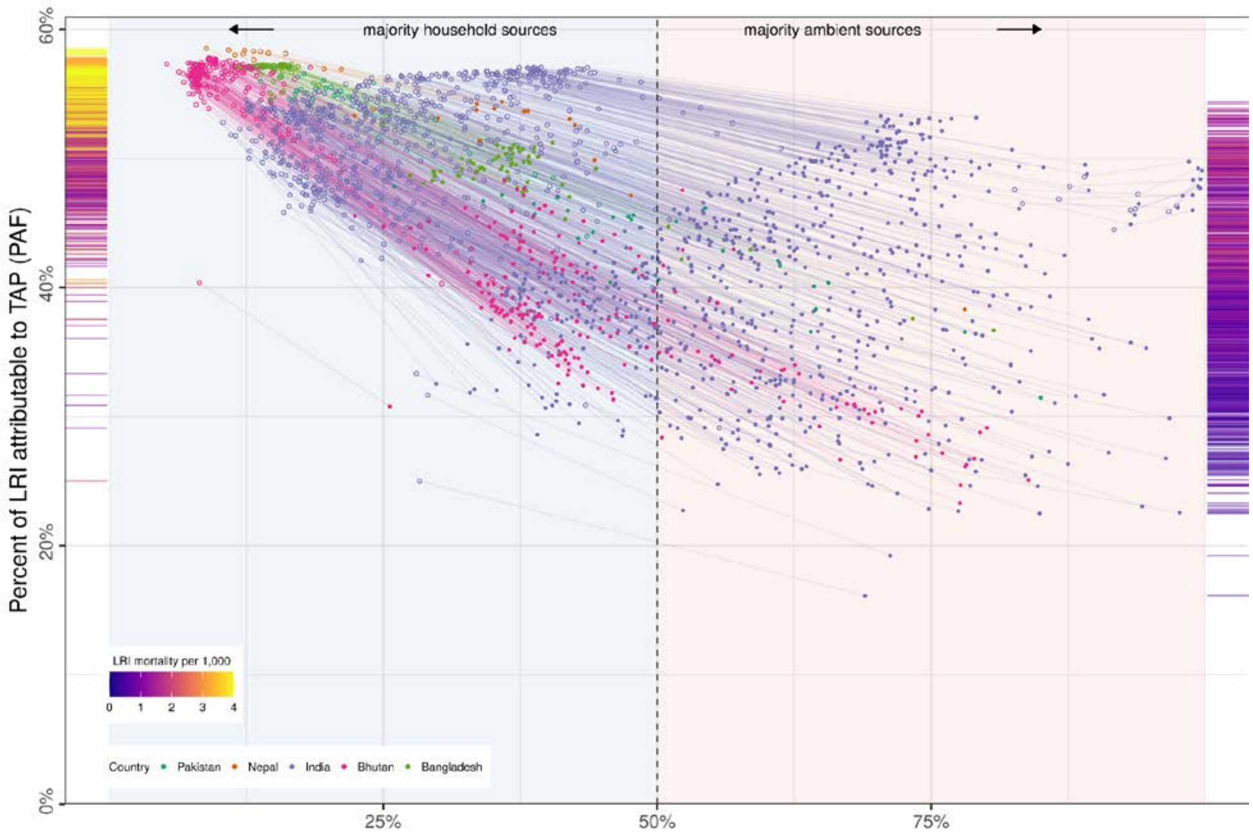

majority household sources
majority ambient sources
Percent of LRI attributable to TAP (PAF)
60%
40%
20%
0%
25%
50%
75%
LRI mortality per 1,000
0
1
2
3
4
Country
Pakistan
Nepal
India
Bhutan
Bangladesh

o) Central Sub-Saharan Africa

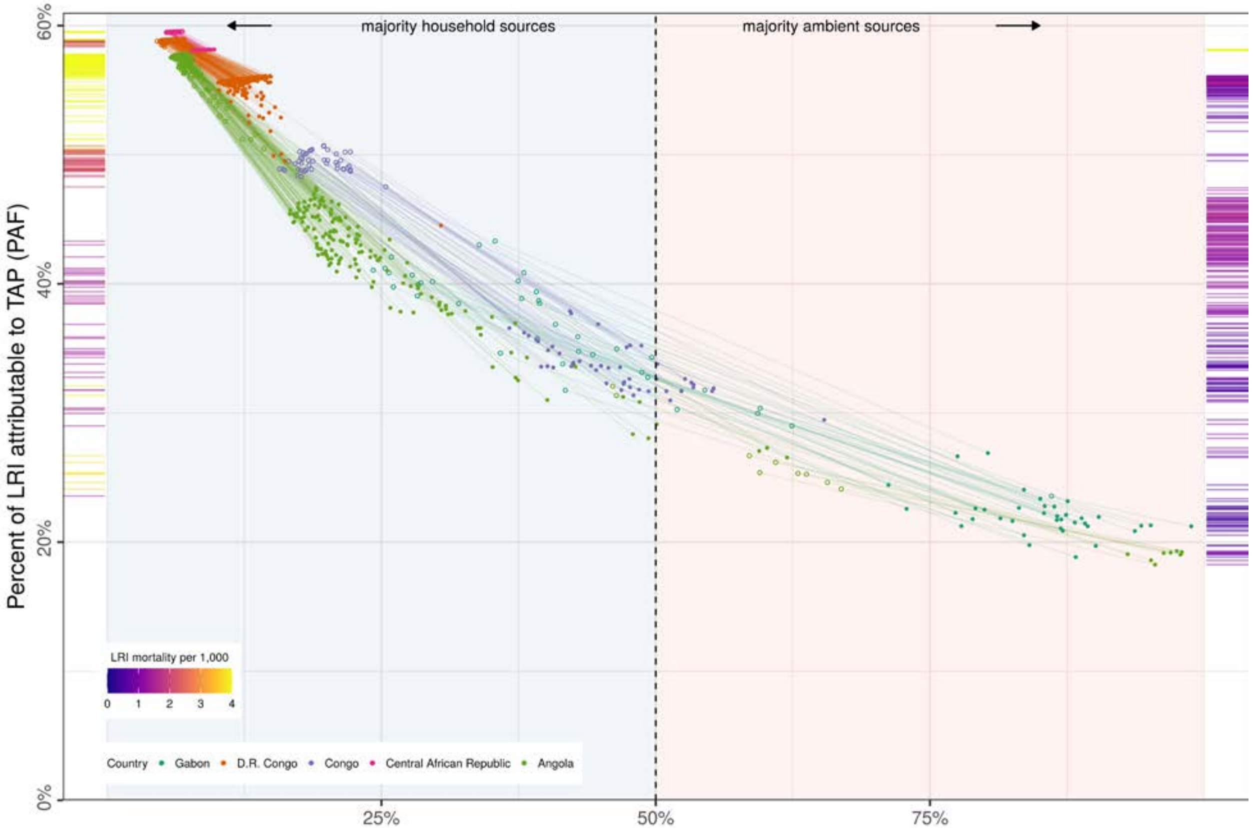

p) Eastern Sub-Saharan Africa

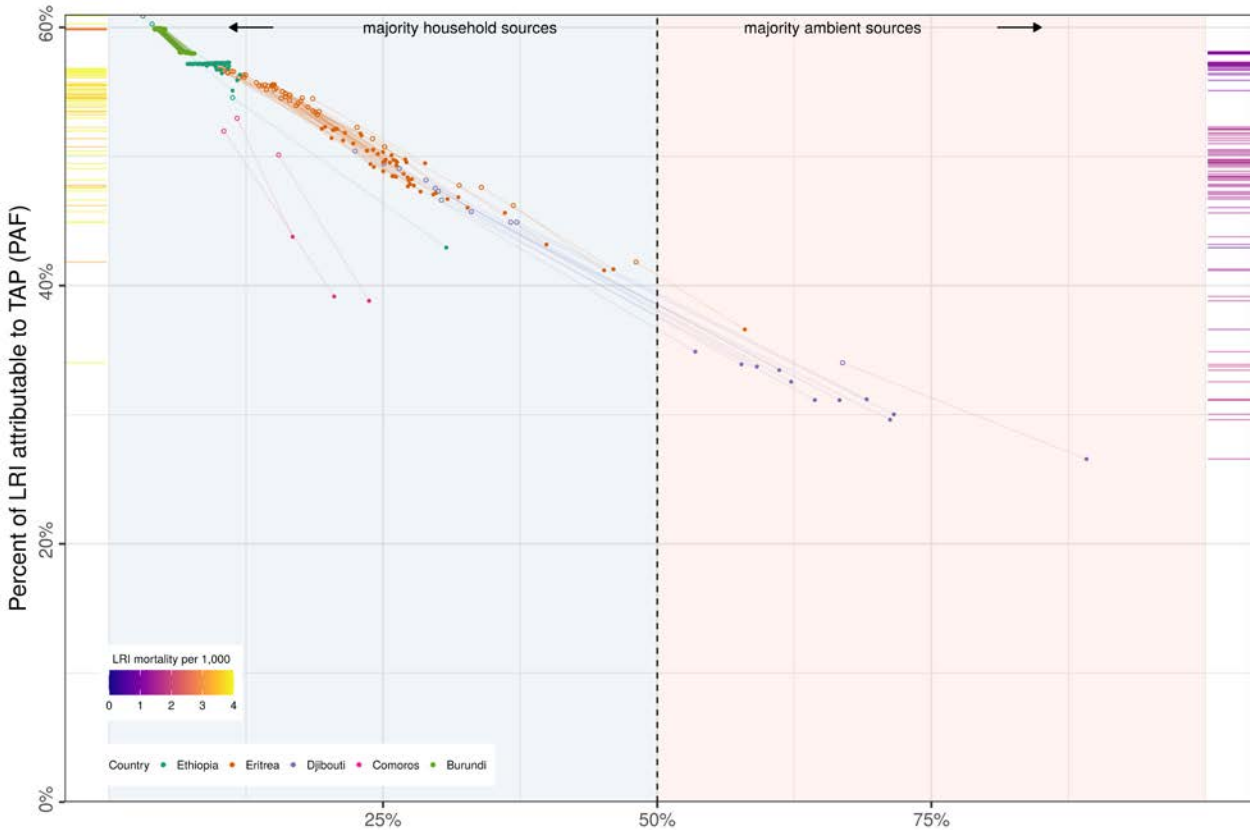

q) Eastern Sub-Saharan Africa, continued

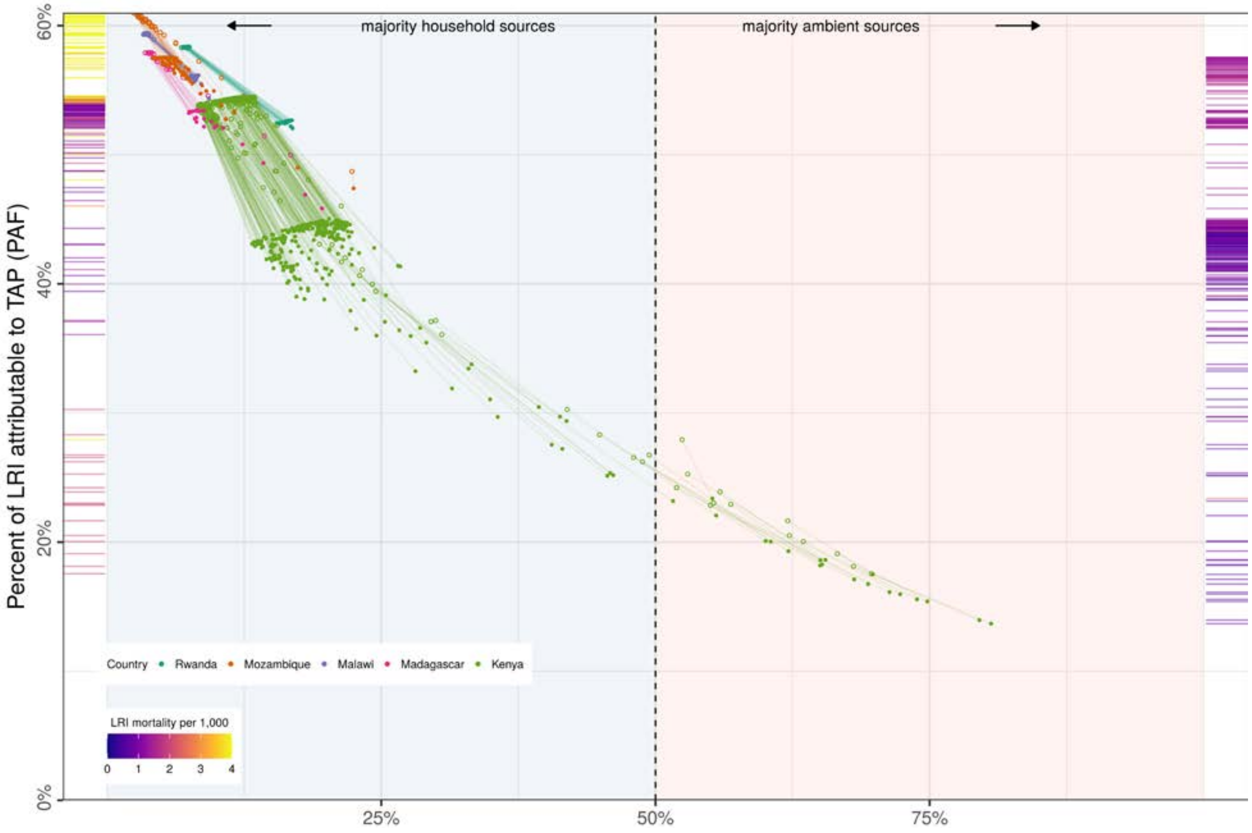

r) Eastern Sub-Saharan Africa, Continued

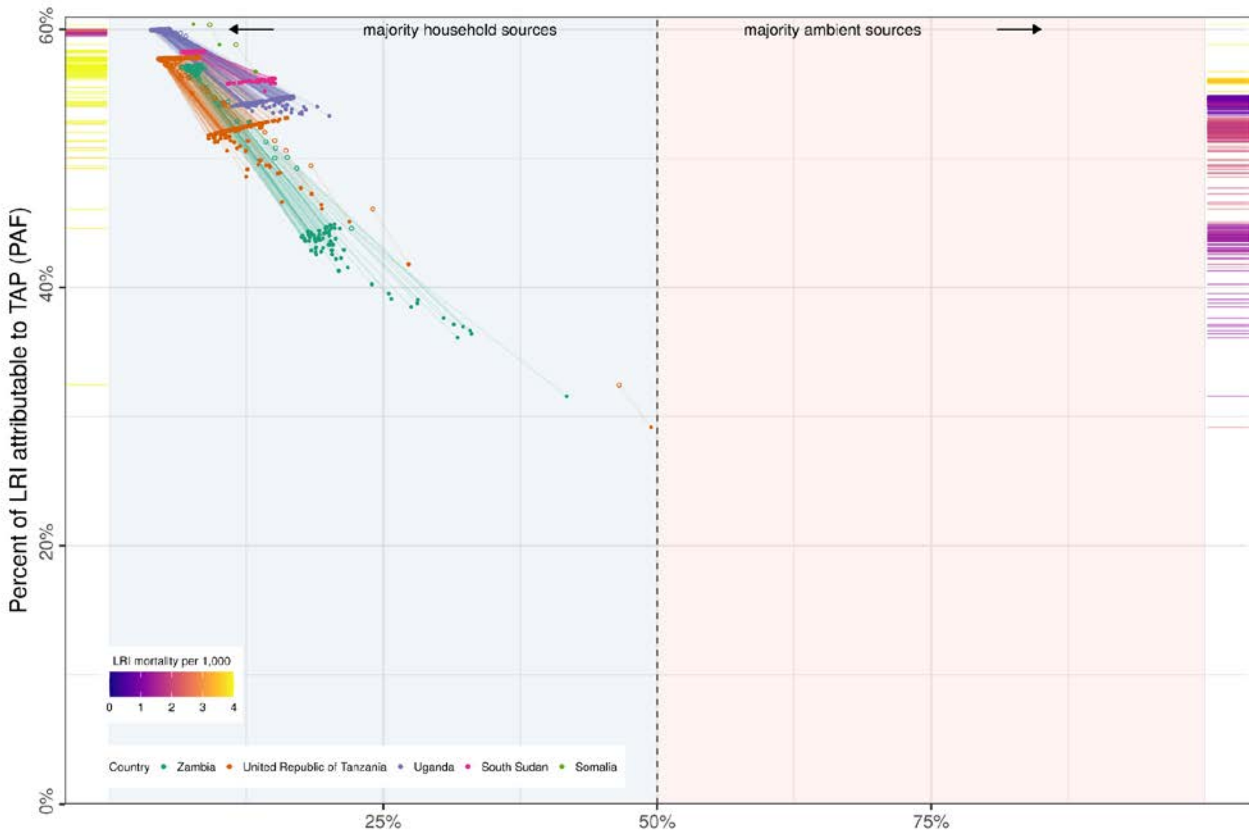

s) Southern Sub-Saharan Africa

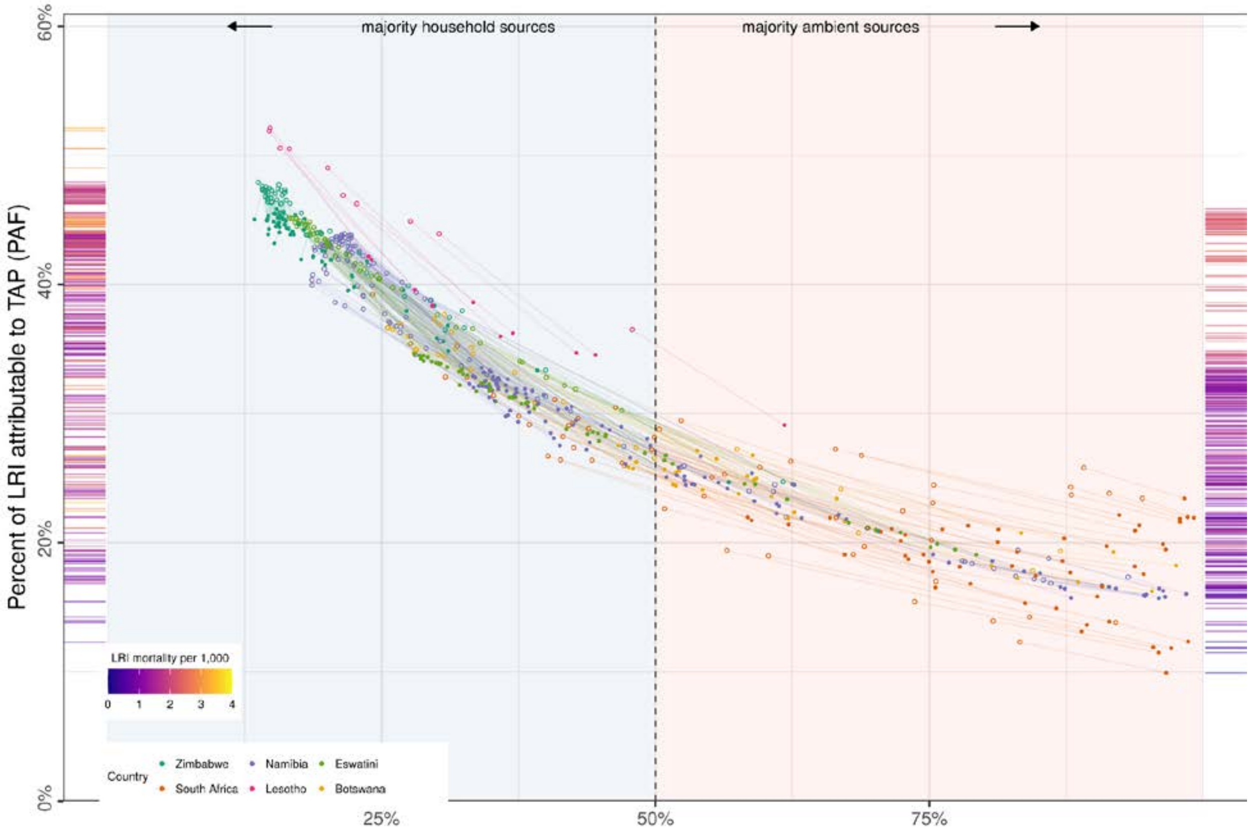

t) Western Sub-Saharan Africa

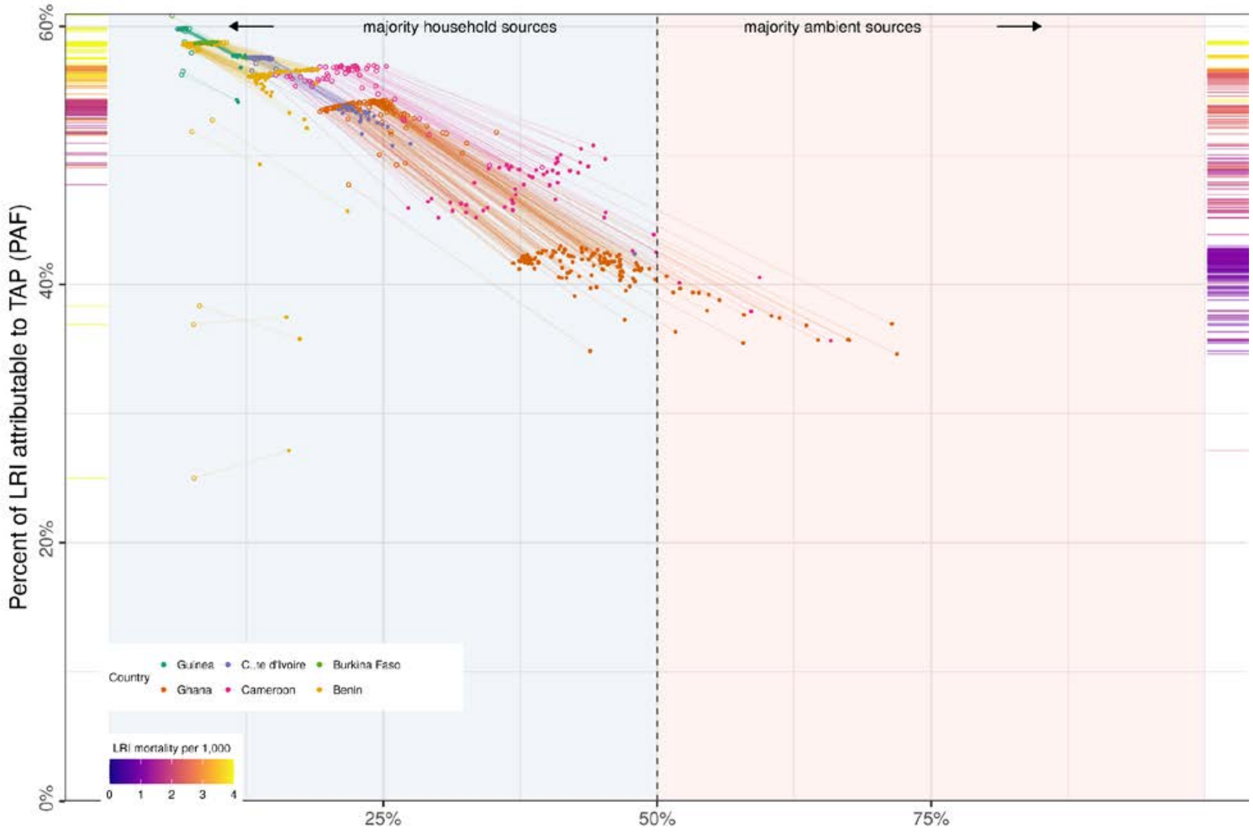

u) Western Sub-Saharan Africa, Continued

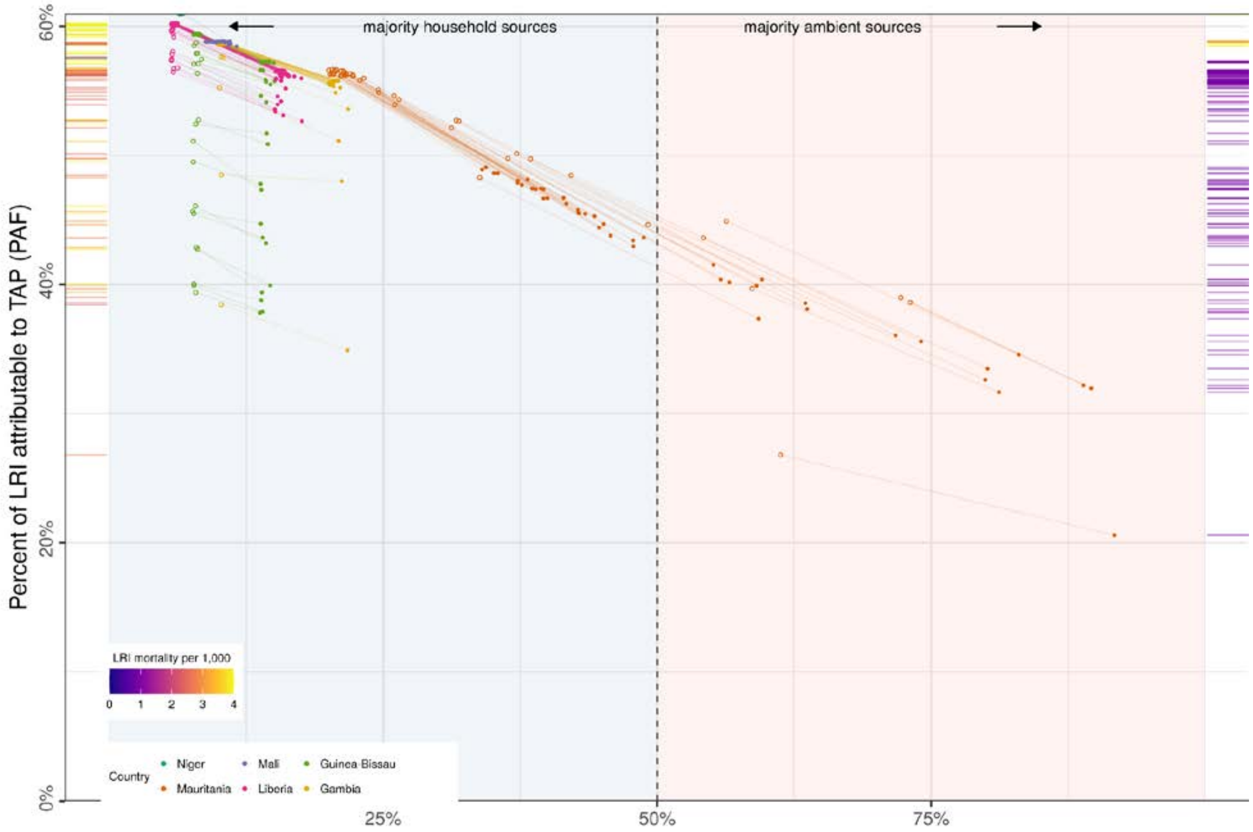

v) Western Sub-Saharan Africa, Continued

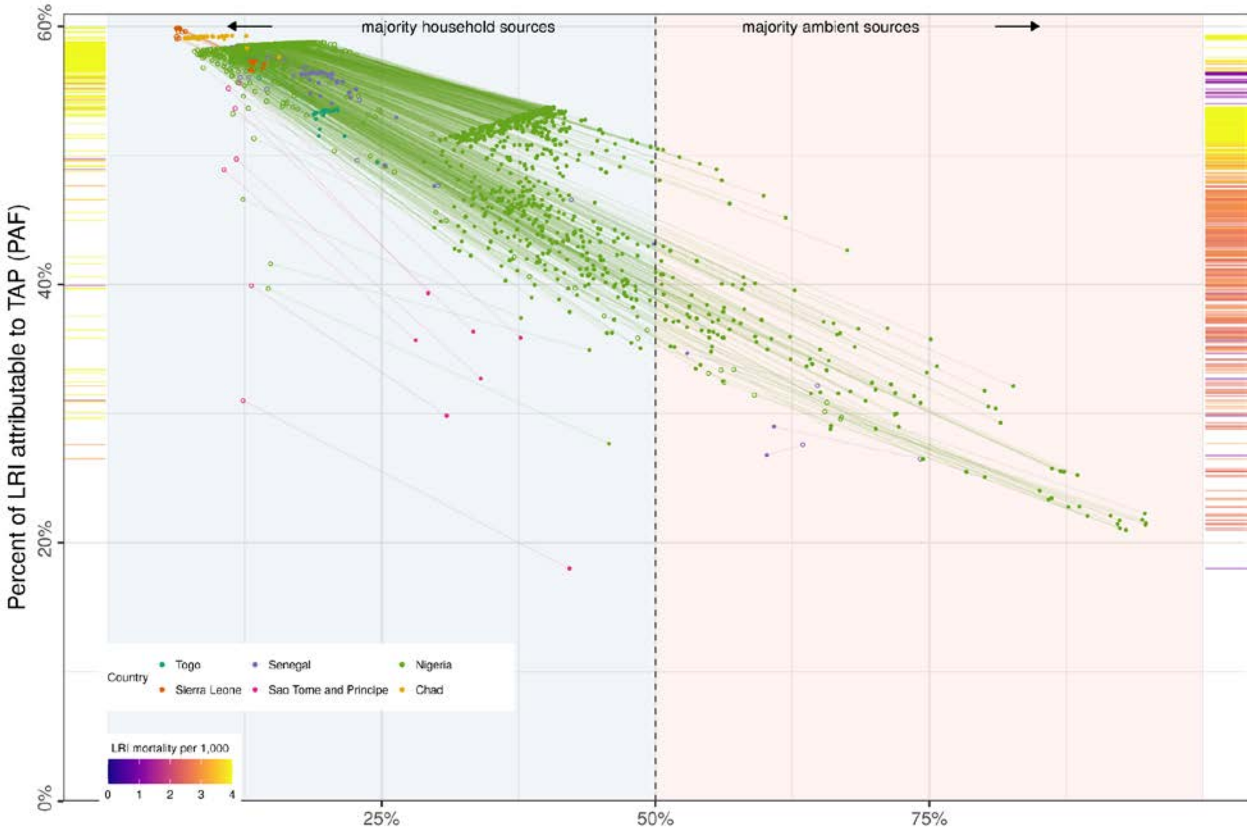

Supplementary Figure 19a-c: SFU in-sample by aggregation level

a) **Admin 0**

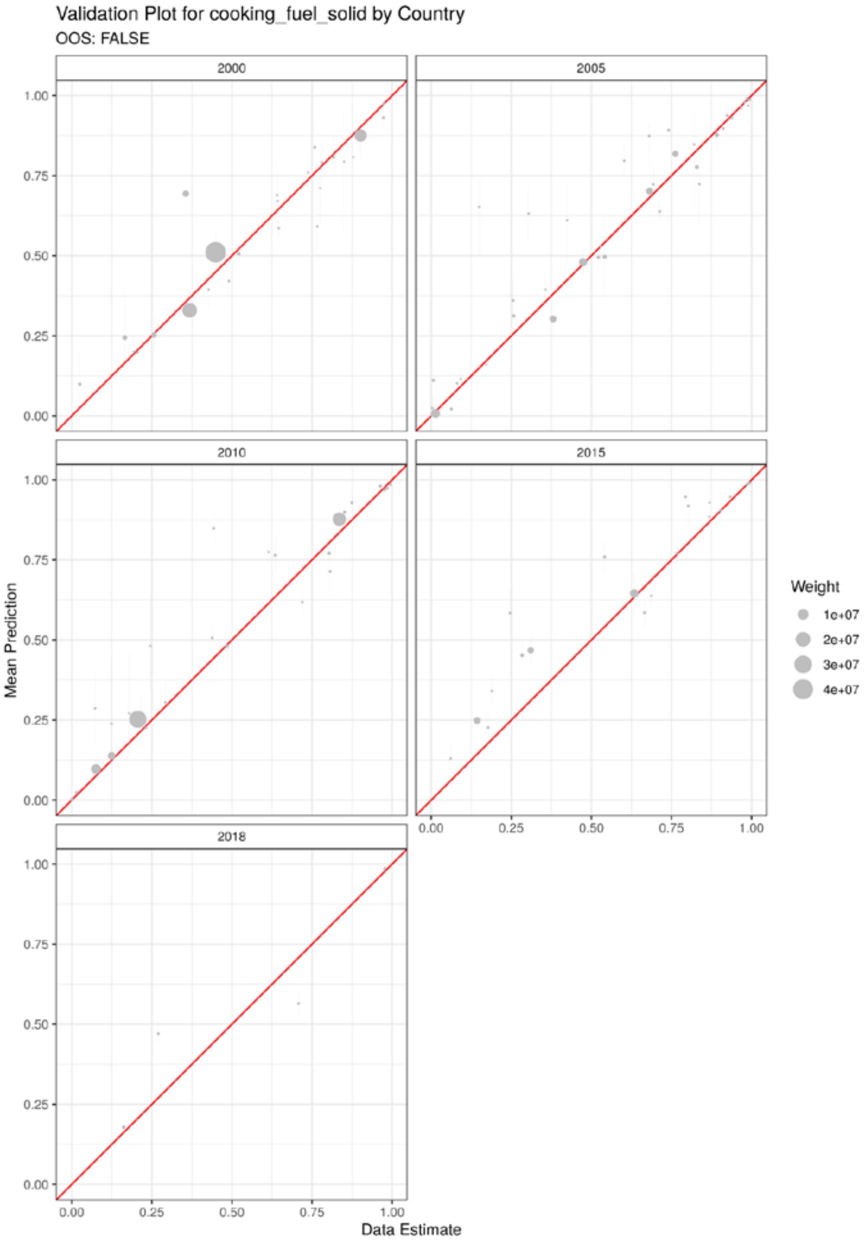

b) Admin 1

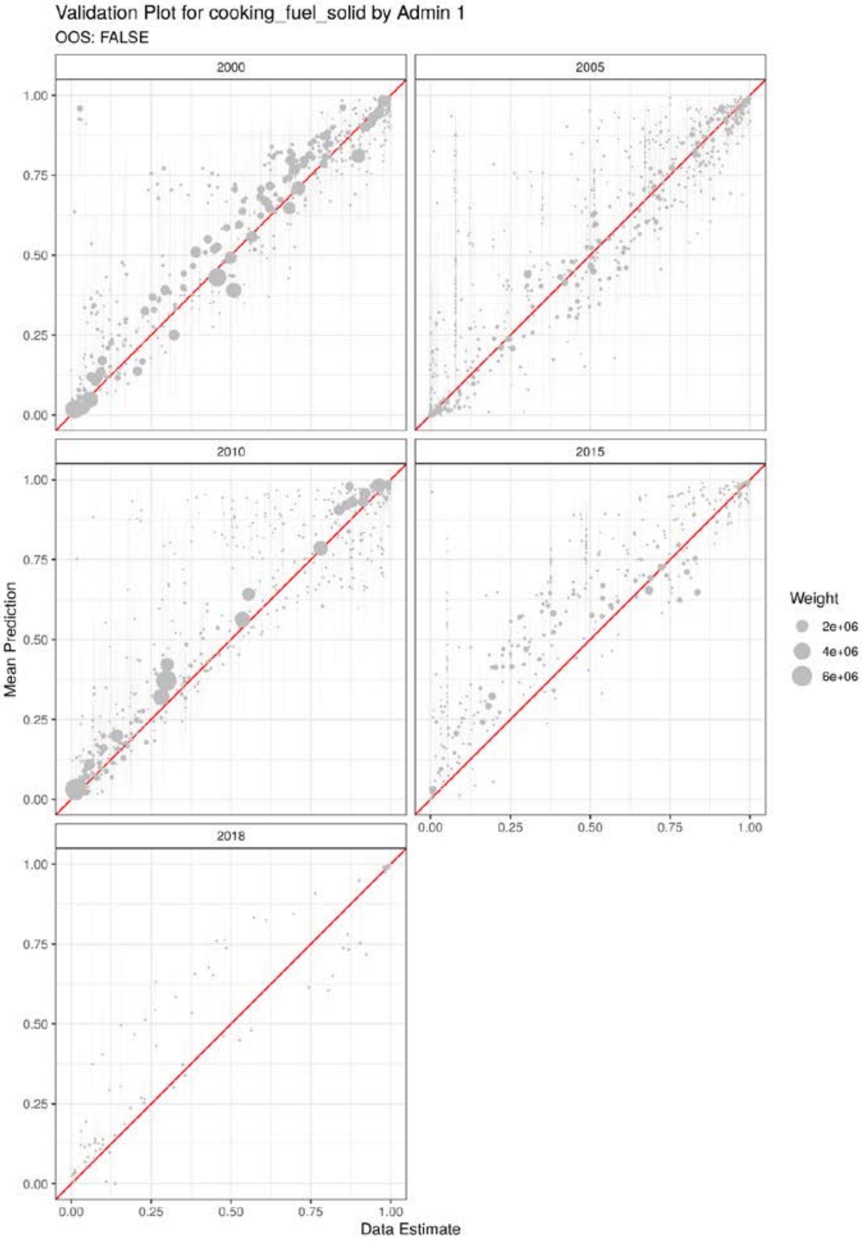
Validation Plot for cooking_fuel_solid by Admin 1
OOS: FALSE
2000
2005
2010
2015
2018
Mean Prediction
Data Estimate
0.00
0.25
0.50
0.75
1.00
Weight
2e+06
4e+06
6e+06

c) **Admin 2**

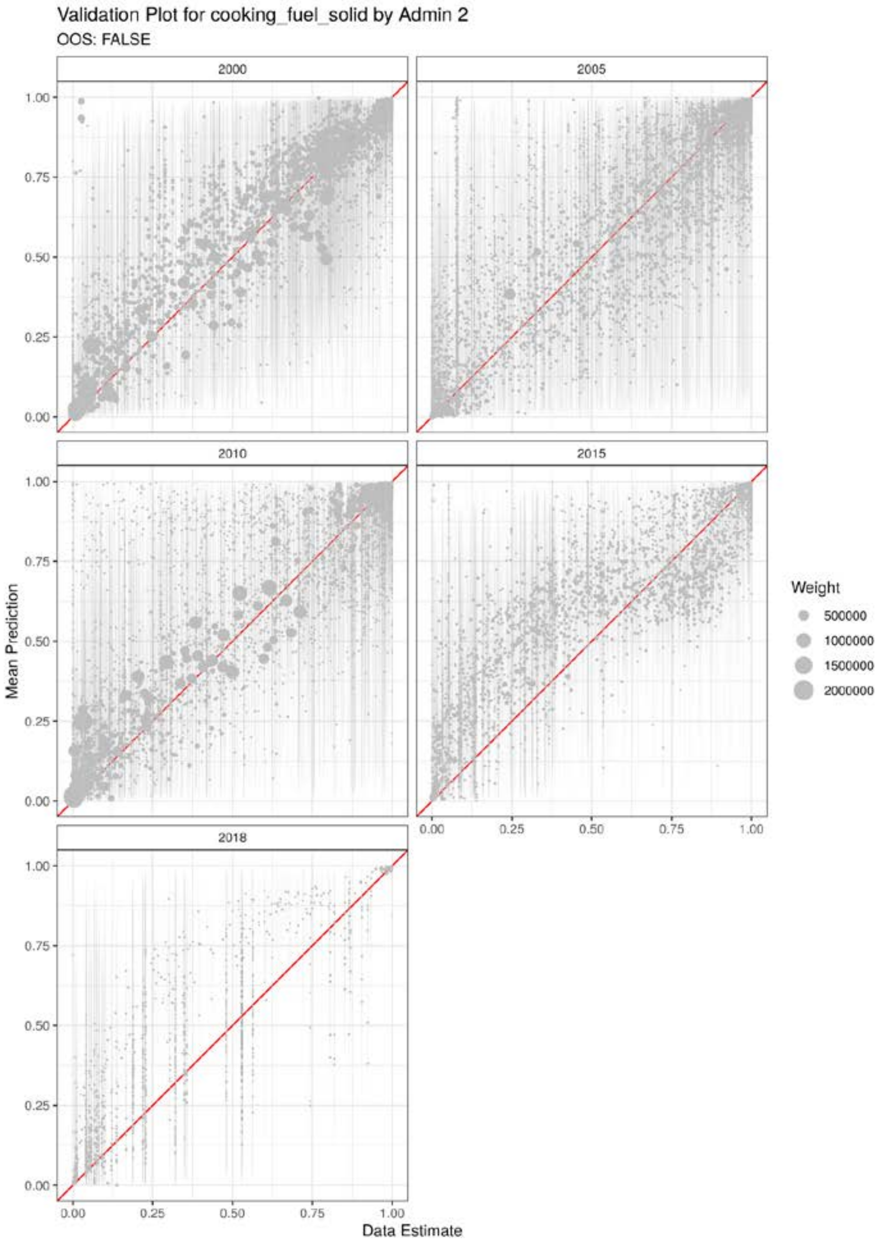

Validation Plot for cooking_fuel_solid by Admin 2
OOS: FALSE
2000
2005
2010
2015
2018
Mean Prediction
Data Estimate
0.00
0.25
0.50
0.75
1.00
Weight
500000
1000000
1500000
2000000

Supplementary Figure 20a-c: SFU out-of-sample by aggregation level

a) **Admin 0**

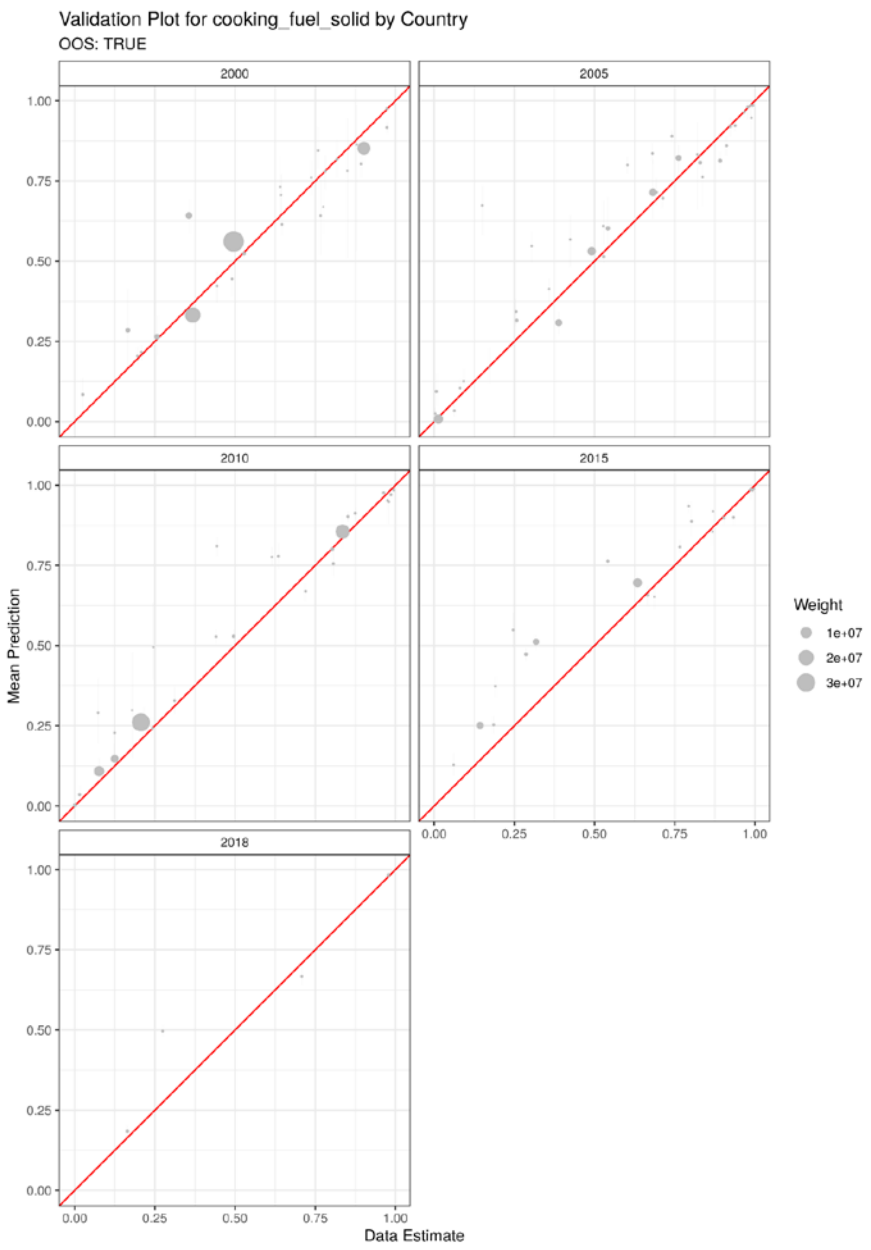

b) Admin 1

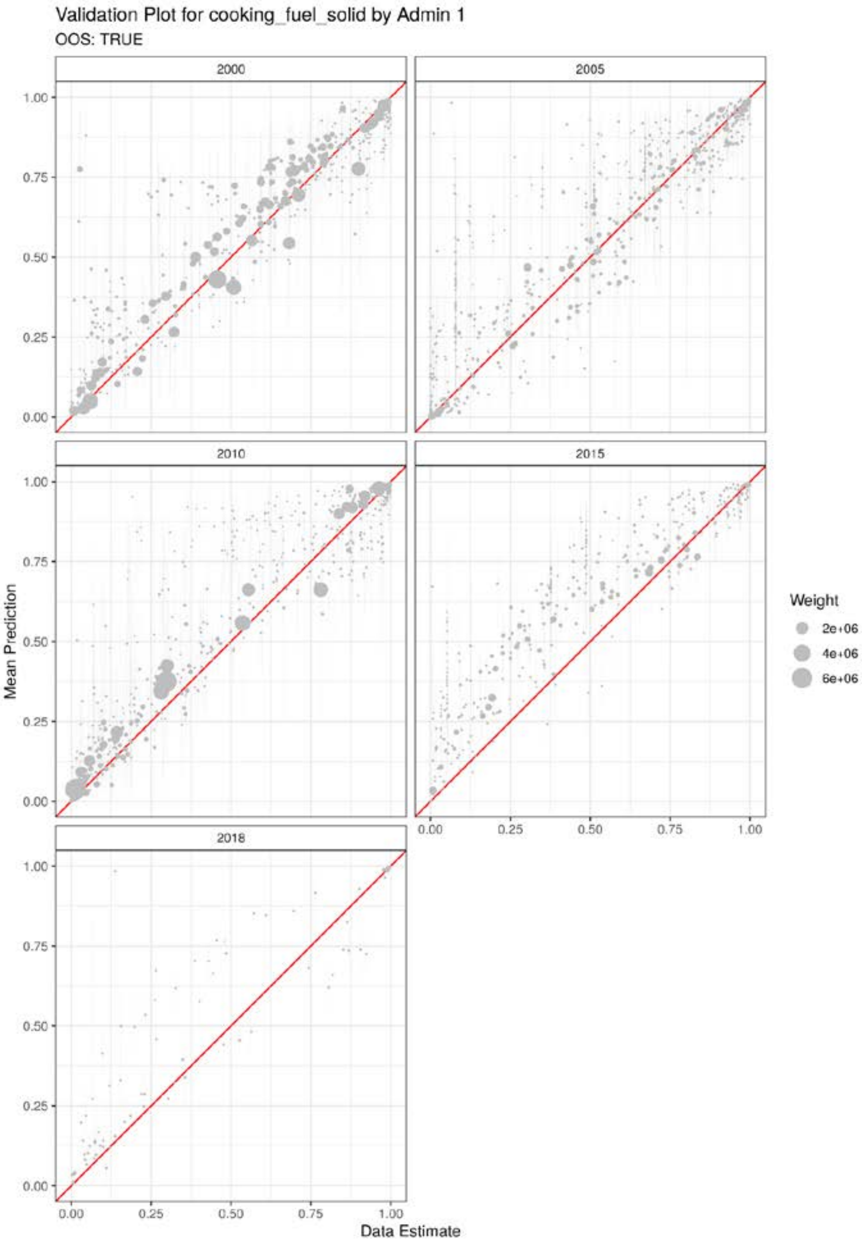

Validation Plot for cooking_fuel_solid by Admin 1
OOS: TRUE
2000
2005
2010
2015
2018
Mean Prediction
Data Estimate
0.00
0.25
0.50
0.75
1.00
Weight
2e+06
4e+06
6e+06

c) Admin 2

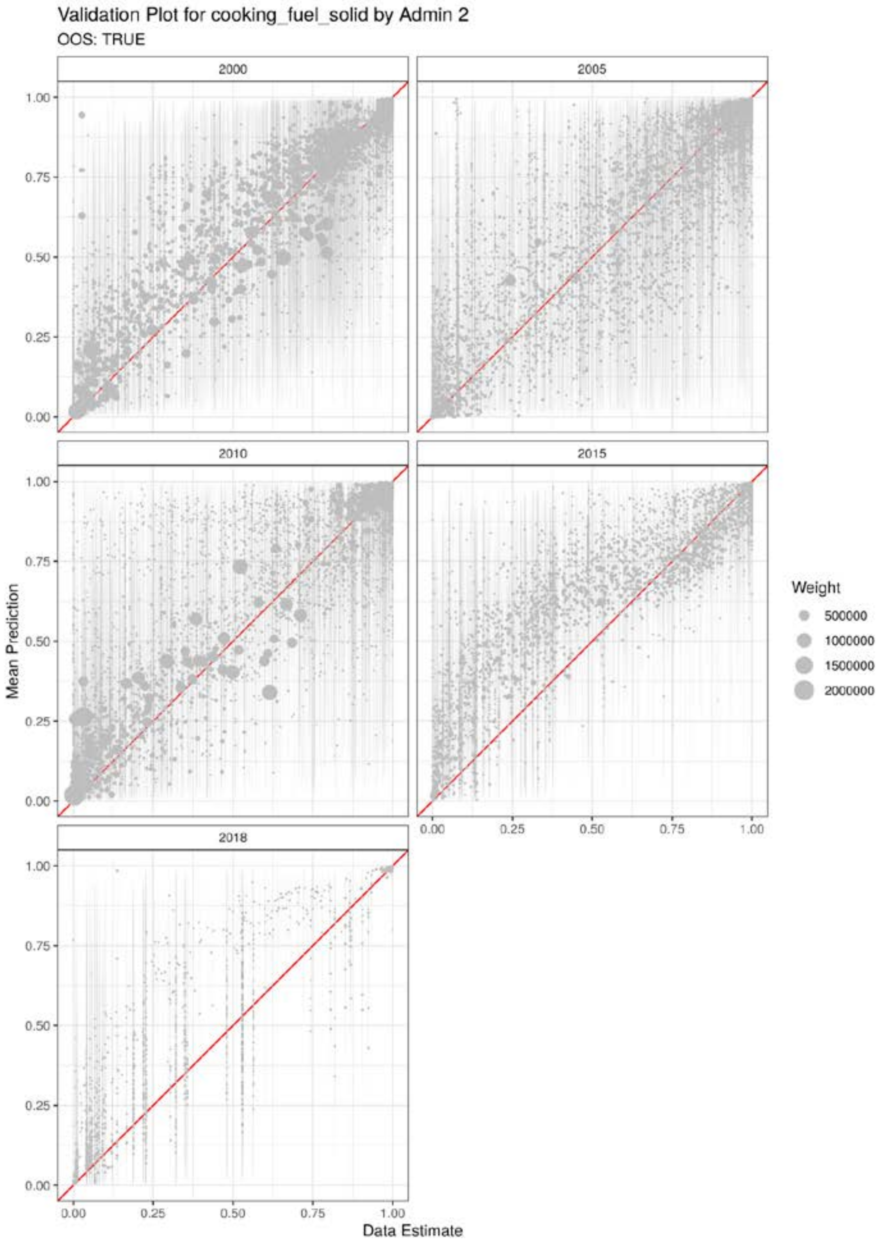
Validation Plot for cooking_fuel_solid by Admin 2
OOS: TRUE
2000
2005
2010
2015
2018
Mean Prediction
Data Estimate
0.00
0.25
0.50
0.75
1.00
Weight
500000
1000000
1500000
2000000

5.0 Supplementary Tables

Supplementary Table 1: Guidelines for Accurate and Transparent Health Estimates Reporting (GATHER) 78
Supplementary Table 2: Socio-demographic Index (SDI) of countries included in analysis 80
Supplementary Table 3: Socio-demographic Index (SDI) of countries excluded from analysis..... 83
Supplementary Table 4: Cooking Fuel input dataset 84
Supplementary Table 5: Covariates used in mapping 106
Supplementary Table 6a-b: Covariates used in ensemble modelling via stacked generalisation, stratified by modelling region 109
Supplementary Table 7: Fitted parameters by region for model of Solid Fuel Use 113
Supplementary Table 8: Predictive metrics for SFU, aggregated to the national level (Admin 0) 116
Supplementary Table 9: Predictive metrics for SFU, aggregated to the first administrative level (Admin 1) 117
Supplementary Table 10: Predictive metrics for SFU, aggregated to the second administrative level (Admin 2) 117

Supplementary Table 1: Guidelines for Accurate and Transparent Health Estimates Reporting (GATHER)

This table's 'Reported on' column is currently only tentative will be updated when all manuscript and Supplementary Information text are finalized.

Item #	Checklist item	Reported on
**Objectives and funding**		
**1**	Define the indicator(s), populations (including age, sex, and geographic entities), and time period(s) for which estimates were made.	Manuscript: Methods<br><br>Supplementary Information: Data Section
**2**	List the funding sources for the work.	Manuscript: Acknowledgements
**Data Inputs**		
For all data inputs from multiple sources that are synthesized as part of the study:		
**3**	Describe how the data were identified and how the data were accessed.	Manuscript: Methods<br><br>Supplementary Information: Data Section
**4**	Specify the inclusion and exclusion criteria. Identify all ad-hoc exclusions.	Manuscript: Methods<br><br>Supplementary Information: Data Section; Figure 1
**5**	Provide information on all included data sources and their main characteristics. For each data source used,	Supplementary Information: Data Section

	report reference information or contact name/institution, population represented, data collection method, year(s) of data collection, sex and age range, diagnostic criteria or measurement method, and sample size, as relevant.	
**6**	Identify and describe any categories of input data that have potentially important biases (e.g., based on characteristics listed in item 5).	Manuscript: Methods, Limitations
For data inputs that contribute to the analysis but were not synthesized as part of the study:		
**7**	Describe and give sources for any other data inputs.	Manuscript: Methods<br><br>Supplementary Information: Covariates Section
For all data inputs:		
**8**	Provide all data inputs in a file format from which data can be efficiently extracted (e.g., a spreadsheet rather than a PDF), including all relevant meta-data listed in item 5. For any data inputs that cannot be shared because of ethical or legal reasons, such as third-party ownership, provide a contact name or the name of the institution that retains the right to the data.	**GHDx link available upon publication**<br><br>Supplementary Information: Data Section
**Data analysis**		
**9**	Provide a conceptual overview of the data analysis method. A diagram may be helpful.	Manuscript: Methods<br><br>Supplementary Information: Figure 1-2
**10**	Provide a detailed description of all steps of the analysis, including mathematical formulae. This description should cover, as relevant, data cleaning, data pre-processing, data adjustments and weighting of data sources, and mathematical or statistical model(s).	Manuscript: Methods<br><br>Supplementary Information: Data section, Figure 1-2
**11**	Describe how candidate models were evaluated and how the final model(s) were selected.	Manuscript: Methods
**12**	Provide the results of an evaluation of model performance, if done, as well as the results of any relevant sensitivity analysis.	Manuscript: Methods
**13**	Describe methods for calculating uncertainty of the estimates. State which sources of uncertainty were, and were not, accounted for in the uncertainty analysis.	Manuscript: Methods
**14**	State how analytic or statistical source code used to generate estimates can be accessed.	https://github.com
**Results and Discussion**		
**15**	Provide published estimates in a file format from which data can be efficiently extracted.	**GHDx link available upon publication**

**16**	Report a quantitative measure of the uncertainty of the estimates (e.g., uncertainty intervals).	Manuscript: Methods
**17**	Interpret results in light of existing evidence. If updating a previous set of estimates, describe the reasons for changes in estimates.	Manuscript: Discussion, Limitations, Future Work
**18**	Discuss limitations of the estimates. Include a discussion of any modelling assumptions or data limitations that affect interpretation of the estimates.	Manuscript: Discussion, Limitations, Future Work

Supplementary Table 2: Socio-demographic Index (SDI) of countries included in analysis

Location Name	ISO3	Region	SDI Level (2019)	SDI (2019)
**Afghanistan**	AFG	North Africa and Middle East	Low SDI	0.343232181
**Benin**	BEN	Western Sub-Saharan Africa	Low SDI	0.352031382
**Burkina Faso**	BFA	Western Sub-Saharan Africa	Low SDI	0.25738523
**Burundi**	BDI	Eastern Sub-Saharan Africa	Low SDI	0.2839369
**Central African Republic**	CAF	Central Sub-Saharan Africa	Low SDI	0.273669311
**Chad**	TCD	Western Sub-Saharan Africa	Low SDI	0.238012087
**Comoros**	COM	Eastern Sub-Saharan Africa	Low SDI	0.45466725
**Cote d'Ivoire**	CIV	Western Sub-Saharan Africa	Low SDI	0.408196034
**Democratic Republic of the Congo**	COD	Central Sub-Saharan Africa	Low SDI	0.382172191
**Eritrea**	ERI	Eastern Sub-Saharan Africa	Low SDI	0.39598799

**Ethiopia**	ETH	Eastern Sub-Saharan Africa	Low SDI	0.342967449
**Guinea**	GIN	Western Sub-Saharan Africa	Low SDI	0.324580469
**Guinea-Bissau**	GNB	Western Sub-Saharan Africa	Low SDI	0.355423473
**Haiti**	HTI	Caribbean	Low SDI	0.431969896
**Liberia**	LBR	Western Sub-Saharan Africa	Low SDI	0.369615587
**Madagascar**	MDG	Eastern Sub-Saharan Africa	Low SDI	0.396293769
**Malawi**	MWI	Eastern Sub-Saharan Africa	Low SDI	0.383936706
**Mali**	MLI	Western Sub-Saharan Africa	Low SDI	0.263484935
**Mozambique**	MOZ	Eastern Sub-Saharan Africa	Low SDI	0.3073155
**Nepal**	NPL	South Asia	Low SDI	0.421996036
**Niger**	NER	Western Sub-Saharan Africa	Low SDI	0.161629227
**Papua New Guinea**	PNG	Oceania	Low SDI	0.394095992
**Rwanda**	RWA	Eastern Sub-Saharan Africa	Low SDI	0.429139732
**Senegal**	SEN	Western Sub-Saharan Africa	Low SDI	0.388935561
**Sierra Leone**	SLE	Western Sub-Saharan Africa	Low SDI	0.347333599
**Somalia**	SOM	Eastern Sub-Saharan Africa	Low SDI	0.080983671
**South Sudan**	SSD	Eastern Sub-Saharan Africa	Low SDI	0.363419895
**Tanzania**	TZA	Eastern Sub-Saharan Africa	Low SDI	0.423252126
**The Gambia**	GMB	Western Sub-Saharan Africa	Low SDI	0.398656595
**Togo**	TGO	Western Sub-Saharan Africa	Low SDI	0.417121069
**Uganda**	UGA	Eastern Sub-Saharan Africa	Low SDI	0.404470575
**Yemen**	YEM	North Africa and Middle East	Low SDI	0.412238638
**Algeria**	DZA	North Africa and Middle East	Middle SDI	0.651946134
**Botswana**	BWA	Southern Sub-Saharan Africa	Middle SDI	0.634369974
**Brazil**	BRA	Tropical Latin America	Middle SDI	0.639528523
**Colombia**	COL	Central Latin America	Middle SDI	0.632568305
**Costa Rica**	CRI	Central Latin America	Middle SDI	0.680111655
**Ecuador**	ECU	Andean Latin America	Middle SDI	0.639975089

**Egypt**	EGY	North Africa and Middle East	Middle SDI	0.657680121
**Equatorial Guinea**	GNQ	Central Sub-Saharan Africa	Middle SDI	0.685124105
**Gabon**	GAB	Central Sub-Saharan Africa	Middle SDI	0.655830167
**Guyana**	GUY	Caribbean	Middle SDI	0.618266528
**Indonesia**	IDN	Southeast Asia	Middle SDI	0.659878161
**Iraq**	IRQ	North Africa and Middle East	Middle SDI	0.670654
**Jamaica**	JAM	Caribbean	Middle SDI	0.684352436
**Mexico**	MEX	Central Latin America	Middle SDI	0.64908918
**Namibia**	NAM	Southern Sub-Saharan Africa	Middle SDI	0.612335601
**Panama**	PAN	Central Latin America	Middle SDI	0.685526052
**Paraguay**	PRY	Tropical Latin America	Middle SDI	0.638276934
**Peru**	PER	Andean Latin America	Middle SDI	0.648408781
**Philippines**	PHL	Southeast Asia	Middle SDI	0.622858401
**South Africa**	ZAF	Southern Sub-Saharan Africa	Middle SDI	0.67825053
**Suriname**	SUR	Caribbean	Middle SDI	0.635912299
**Syria**	SYR	North Africa and Middle East	Middle SDI	0.619444771
**Thailand**	THA	Southeast Asia	Middle SDI	0.686576643
**Tunisia**	TUN	North Africa and Middle East	Middle SDI	0.67176511
**Turkmenistan**	TKM	Central Asia	Middle SDI	0.670472955
**Uzbekistan**	UZB	Central Asia	Middle SDI	0.631435113
**Vietnam**	VNM	Southeast Asia	Middle SDI	0.61743942
**Angola**	AGO	Central Sub-Saharan Africa	Low-middle SDI	0.469857606
**Bangladesh**	BGD	South Asia	Low-middle SDI	0.482676885
**Belize**	BLZ	Caribbean	Low-middle SDI	0.602517206
**Bhutan**	BTN	South Asia	Low-middle SDI	0.455077121
**Bolivia**	BOL	Andean Latin America	Low-middle SDI	0.56641717
**Cambodia**	KHM	Southeast Asia	Low-middle SDI	0.468990684
**Cameroon**	CMR	Western Sub-Saharan Africa	Low-middle SDI	0.489610885
**Djibouti**	DJI	Eastern Sub-Saharan Africa	Low-middle SDI	0.458670351
**Dominican Republic**	DOM	Caribbean	Low-middle SDI	0.591906159
**El Salvador**	SLV	Central Latin America	Low-middle SDI	0.572623622
**Ghana**	GHA	Western Sub-Saharan Africa	Low-middle SDI	0.556887637
**Guatemala**	GTM	Central Latin America	Low-middle SDI	0.526345014
**Honduras**	HND	Central Latin America	Low-middle SDI	0.495975306

**India**	IND	South Asia	Low-middle SDI	0.565900043
**Kenya**	KEN	Eastern Sub-Saharan Africa	Low-middle SDI	0.50844187
**Kyrgyzstan**	KGZ	Central Asia	Low-middle SDI	0.595661124
**Laos**	LAO	Southeast Asia	Low-middle SDI	0.48997377
**Lesotho**	LSO	Southern Sub-Saharan Africa	Low-middle SDI	0.507011919
**Mauritania**	MRT	Western Sub-Saharan Africa	Low-middle SDI	0.49569688
**Mongolia**	MNG	Central Asia	Low-middle SDI	0.605566533
**Morocco**	MAR	North Africa and Middle East	Low-middle SDI	0.548494837
**Myanmar**	MMR	Southeast Asia	Low-middle SDI	0.520765117
**Nicaragua**	NIC	Central Latin America	Low-middle SDI	0.516771682
**Nigeria**	NGA	Western Sub-Saharan Africa	Low-middle SDI	0.51525579
**Pakistan**	PAK	South Asia	Low-middle SDI	0.448851517
**Palestine**	PSE	North Africa and Middle East	Low-middle SDI	0.587868157
**Republic of the Congo**	COG	Central Sub-Saharan Africa	Low-middle SDI	0.568429044
**Sao Tome and Principe**	STP	Western Sub-Saharan Africa	Low-middle SDI	0.502156323
**Sudan**	SDN	North Africa and Middle East	Low-middle SDI	0.515086174
**Swaziland**	SWZ	Southern Sub-Saharan Africa	Low-middle SDI	0.577452676
**Tajikistan**	TJK	Central Asia	Low-middle SDI	0.538654316
**Timor-Leste**	TLS	Southeast Asia	Low-middle SDI	0.51372636
**Zambia**	ZMB	Eastern Sub-Saharan Africa	Low-middle SDI	0.505086432
**Zimbabwe**	ZWE	Southern Sub-Saharan Africa	Low-middle SDI	0.475632182
**China**	CHN	East Asia	High-middle SDI	0.685967969
**Iran**	IRN	North Africa and Middle East	High-middle SDI	0.670415892
**Jordan**	JOR	North Africa and Middle East	High-middle SDI	0.731025449
**Libya**	LBY	North Africa and Middle East	High-middle SDI	0.709294185
**Sri Lanka**	LKA	Southeast Asia	High-middle SDI	0.689520933
**Trinidad and Tobago**	TTO	Caribbean	High-middle SDI	0.756858482

Supplementary Table 3: Socio-demographic Index (SDI) of countries excluded from analysis

Location Name	ISO3	Region	SDI Level (2019)	SDI (2019)
**Solomon Islands**	SLB	Oceania	Low SDI	0.406846203
**Cuba**	CUB	Caribbean	Middle SDI	0.667855786
**Fiji**	FJI	Oceania	Middle SDI	0.664044301
**Grenada**	GRD	Caribbean	Middle SDI	0.66915426
**Saint Lucia**	LCA	Caribbean	Middle SDI	0.670409821
**Saint Vincent and the Grenadines**	VCT	Caribbean	Middle SDI	0.626784725
**Samoa**	WSM	Oceania	Middle SDI	0.640812792
**Tonga**	TON	Oceania	Middle SDI	0.635899444
**Cape Verde**	CPV	Western Sub-Saharan Africa	Low-middle SDI	0.524957239
**Federated States of Micronesia**	FSM	Oceania	Low-middle SDI	0.579853271
**Kiribati**	KIR	Oceania	Low-middle SDI	0.526513752
**Maldives**	MDV	Southeast Asia	Low-middle SDI	0.561816599
**Marshall Islands**	MHL	Oceania	Low-middle SDI	0.544149104
**Vanuatu**	VUT	Oceania	Low-middle SDI	0.484678789
**Venezuela**	VEN	Central Latin America	Low-middle SDI	0.606514719
**American Samoa**	ASM	Oceania	High-middle SDI	0.712008609
**Dominica**	DMA	Caribbean	High-middle SDI	0.72914183
**Malaysia**	MYS	Southeast Asia	High-middle SDI	0.737370877
**Seychelles**	SYC	Southeast Asia	High-middle SDI	0.723944512

Supplementary Table 4: Cooking Fuel input dataset

NID	ISO3	Start Year	End Year	Survey Series	Survey Module
20888	TLS	2003	2003	ADB_DHS	HH
18468	AFG	2006	2006	AFG/HEALTH_SURVEY_2006	HH
30394	AGO	2008	2009	AGO/INTEGRATED_SURVEY_ON_POPULATION_WELFARE	HHM
107340	LBY	2007	2007	ARAB_LEAGUE_PAPFAM	HHM
126909	MAR	2010	2011	ARAB_LEAGUE_PAPFAM	WN
9999	PSE	2006	2007	ARAB_LEAGUE_PAPFAM	HH
126911	SYR	2009	2009	ARAB_LEAGUE_PAPFAM	HH
151805	BEN	2011	2012	BEN/HOUSEHOLD_LIVING_CONDITIONS_SURVEY	HH

236156	BFA	2014	2014	BFA/CONTINUOUS_MULTISECTORAL_SURVEY_EMC	HHM
283269	BGD	2015	2015	BGD/INTEGRATED_HOUSEHOLD_SURVEY	HHM
153062	BGD	2011	2012	BGD/INTEGRATED_HOUSEHOLD_SURVEY_MALE	HH
336686	BOL	2016	2016	BOL_HHS	HHM
317285	BOL	2015	2015	BOL_HHS	HHM
1245	BOL	2000	2000	BOL/HH_SURVEY	HH
1259	BOL	2001	2001	BOL/HH_SURVEY	HHM
1278	BOL	2002	2002	BOL/HH_SURVEY	HH
32374	BOL	2003	2004	BOL/HH_SURVEY	HHM
32388	BOL	2005	2005	BOL/HH_SURVEY	HHM
148343	BOL	2006	2006	BOL/HH_SURVEY	WN
148344	BOL	2007	2007	BOL/HH_SURVEY	HHM
164634	BOL	2011	2011	BOL/HH_SURVEY	HHM
164635	BOL	2013	2013	BOL/HH_SURVEY	HH
283486	BOL	2014	2014	BOL/HH_SURVEY	HHM
93522	BRA	2009	2009	BRA/HOUSEHOLD_SAMPLE_SURVEY_PNAD	HH
93487	BRA	2008	2008	BRA/HOUSEHOLD_SAMPLE_SURVEY_PNAD	HH
1489	BRA	2003	2003	BRA/HOUSEHOLD_SAMPLE_SURVEY_PNAD	HH
238441	BRA	2014	2014	BRA/HOUSEHOLD_SAMPLE_SURVEY_PNAD	HH
106724	BRA	2011	2011	BRA/HOUSEHOLD_SAMPLE_SURVEY_PNAD	HH
156581	BRA	2012	2012	BRA/HOUSEHOLD_SAMPLE_SURVEY_PNAD	HH
156583	BRA	2013	2013	BRA/HOUSEHOLD_SAMPLE_SURVEY_PNAD	HH
281548	BRA	2015	2015	BRA/HOUSEHOLD_SAMPLE_SURVEY_PNAD	HH
1488	BRA	2002	2002	BRA/HOUSEHOLD_SAMPLE_SURVEY_PNAD	HH
93490	BRA	2007	2007	BRA/HOUSEHOLD_SAMPLE_SURVEY_PNAD	HH
1490	BRA	2004	2004	BRA/HOUSEHOLD_SAMPLE_SURVEY_PNAD	HH
1477	BRA	2001	2001	BRA/HOUSEHOLD_SAMPLE_SURVEY_PNAD	HHM
80311	BRA	2005	2005	BRA/HOUSEHOLD_SAMPLE_SURVEY_PNAD	HH
93528	BRA	2006	2006	BRA/HOUSEHOLD_SAMPLE_SURVEY_PNAD	HH
195010	BRA	2013	2013	BRA/NATIONAL_HEALTH_SURVEY_PNS	HHM
32244	BRA	2002	2005	BRA/RISK_FACTOR_MORBIDITY_NCD_SURVEY	HH
1175	BTN	2005	2005	BTN/CENSUS	HHM
151536	BWA	2009	2010	BWA_CWIS	HHM
22114	BWA	2004	2004	BWA/AIDS_IMPACT_SURVEY	HHM
134753	BWA	2013	2013	BWA/AIDS_IMPACT_SURVEY	HHM

21970	BWA	2006	2006	BWA/DEMOGRAPHIC_SURVEY	HH
22125	BWA	2007	2008	BWA/FAMILY_HEALTH_SURVEY	HH
4779	GTM	2008	2009	CDC_RHS	WN
10370	PRY	2004	2004	CDC_RHS	WN
27525	PRY	2008	2008	CDC_RHS	WN
27599	SLV	2002	2003	CDC_RHS	WN
314646	BLZ	2013	2013	CHILD_ACTIVITY_SURVEY	HH
283812	CHN	2010	2010	CHINA_FAMILY_PANEL_STUDIES	HH
283815	CHN	2012	2012	CHINA_FAMILY_PANEL_STUDIES	HH
2039	CMR	2001	2001	CMR/HH_SURVEY	HH
3100	COL	2007	2008	COL/NATIONAL_HEALTH_SURVEY_ENS	HH
68396	COL	2011	2011	COL/NATIONAL_QUALITY_OF_LIFE_SURVEY_ENCV	HH
68334	COL	2010	2010	COL/NATIONAL_QUALITY_OF_LIFE_SURVEY_ENCV	HH
3209	CRI	2001	2001	CRI/MULTIPURPOSE_HH_SURVEY	HHM
3212	CRI	2002	2002	CRI/MULTIPURPOSE_HH_SURVEY	HHM
3218	CRI	2003	2003	CRI/MULTIPURPOSE_HH_SURVEY	HHM
3215	CRI	2004	2004	CRI/MULTIPURPOSE_HH_SURVEY	HHM
3177	CRI	2005	2005	CRI/MULTIPURPOSE_HH_SURVEY	HHM
3180	CRI	2006	2006	CRI/MULTIPURPOSE_HH_SURVEY	HHM
3183	CRI	2007	2007	CRI/MULTIPURPOSE_HH_SURVEY	HHM
30434	CRI	2008	2008	CRI/MULTIPURPOSE_HH_SURVEY	HHM
30437	CRI	2009	2009	CRI/MULTIPURPOSE_HH_SURVEY	HHM
149360	CRI	2010	2010	CRI/NATIONAL_HOUSEHOLD_SURVEY_ENAHO	HHM
149366	CRI	2011	2011	CRI/NATIONAL_HOUSEHOLD_SURVEY_ENAHO	HHM
149372	CRI	2012	2012	CRI/NATIONAL_HOUSEHOLD_SURVEY_ENAHO	HHM
169717	CRI	2014	2014	CRI/NATIONAL_HOUSEHOLD_SURVEY_ENAHO	HHM
238431	CRI	2015	2015	CRI/NATIONAL_HOUSEHOLD_SURVEY_ENAHO	HHM
282883	CRI	2016	2016	CRI/NATIONAL_HOUSEHOLD_SURVEY_ENAHO	HHM
3455	DOM	2006	2006	DOM/HOUSEHOLD_SURVEY_ENHOGAR	HH
3441	DOM	2007	2007	DOM/HOUSEHOLD_SURVEY_ENHOGAR	HH
35583	DOM	2005	2005	DOM/HOUSEHOLD_SURVEY_ENHOGAR	HHM
95320	ECU	2010	2010	ECU/CENSUS	HH
46924	ECU	2005	2006	ECU/LIVING_CONDITIONS_SURVEY_ECV	HHM
153674	ECU	2012	2012	ECU/NATIONAL_HEALTH_AND_NUTRITION_SURVEY_ENSA NUT	HHM

323944	BOL	2016	2016	EDSA	HHM
34085	ETH	2004	2004	ETH/WELFARE_MONITORING_SURVEY	HH
365281	ETH	2015	2016	ETH/WELFARE_MONITORING_SURVEY	HH
4010	GHA	2001	2001	GHA/CHILD_LABOR_SURVEY	HHM
5009	HND	2004	2004	HND/SURVEY_OF_LIVING_CONDITIONS	HH
219201	IDN	2012	2012	IDN/FAMILY_LIFE_SURVEY_EAST	HH
43552	IDN	2009	2009	IDN/SOCIOECONOMIC_SURVEY_SUSENAS	HH
150884	IDN	2012	2012	IDN/SOCIOECONOMIC_SURVEY_SUSENAS	HH
151184	IDN	2013	2013	IDN/SOCIOECONOMIC_SURVEY_SUSENAS	HH
85265	IDN	2011	2011	IDN/SOCIOECONOMIC_SURVEY_SUSENAS	HH
65181	IND	2009	2010	IND/COVERAGE_EVALUATION_SURVEY	CH
23219	IND	2002	2004	IND/DISTRICT_LEVEL_HOUSEHOLD_SURVEY	HH
23258	IND	2007	2008	IND/DISTRICT_LEVEL_HOUSEHOLD_SURVEY	HH
165390	IND	2012	2014	IND/DISTRICT_LEVEL_HOUSEHOLD_SURVEY	HH
174154	IND	2010	2010	IND/LONGITUDINAL_AGING_STUDY_IN_INDIA	HH_HHM
225626	IND	2014	2014	IND/NATIONAL_SAMPLE_SURVEY	HHM
5285	IND	2004	2004	IND/NATIONAL_SAMPLE_SURVEY	HH_HHM
129770	IND	2011	2012	IND/NNMB_RURAL_SURVEY	HH
367347	BEN	2002	2002	IPUMS_CENSUS	HHM
367419	BEN	2013	2013	IPUMS_CENSUS	HHM
105403	BFA	2006	2006	IPUMS_CENSUS	HHM
1362	BOL	2001	2001	IPUMS_CENSUS	HHM
294205	BWA	2001	2001	IPUMS_CENSUS	HHM
105800	CMR	2005	2005	IPUMS_CENSUS	HHM
3029	COL	2005	2006	IPUMS_CENSUS	HHM
227111	CRI	2011	2011	IPUMS_CENSUS	HHM
151304	DOM	2010	2010	IPUMS_CENSUS	HHM
3549	ECU	2001	2001	IPUMS_CENSUS	HHM
105801	ECU	2010	2010	IPUMS_CENSUS	HHM
35578	EGY	2006	2006	IPUMS_CENSUS	HHM
38508	GHA	2000	2000	IPUMS_CENSUS	HHM
151306	GHA	2010	2010	IPUMS_CENSUS	HHM
367563	HND	2001	2001	IPUMS_CENSUS	HHM
106473	HTI	2003	2003	IPUMS_CENSUS	HHM

56573	IDN	2005	2005	IPUMS_CENSUS	HHM
39396	IRN	2006	2006	IPUMS_CENSUS	HHM
39450	JAM	2001	2001	IPUMS_CENSUS	HHM
35329	KHM	2008	2008	IPUMS_CENSUS	HHM
151310	LBR	2008	2008	IPUMS_CENSUS	HHM
367585	LSO	2006	2006	IPUMS_CENSUS	HHM
151311	MLI	2009	2009	IPUMS_CENSUS	HHM
40186	MWI	2008	2008	IPUMS_CENSUS	HHM
151312	NGA	2007	2007	IPUMS_CENSUS	HHM
151314	NGA	2009	2009	IPUMS_CENSUS	HHM
151315	NGA	2010	2010	IPUMS_CENSUS	HHM
56520	NIC	2005	2005	IPUMS_CENSUS	HHM
40907	PAN	2000	2000	IPUMS_CENSUS	HHM
106529	PAN	2010	2010	IPUMS_CENSUS	HHM
41267	PER	2007	2007	IPUMS_CENSUS	HHM
41296	PHL	2000	2000	IPUMS_CENSUS	HHM
367607	PHL	2010	2010	IPUMS_CENSUS	HHM
227167	PRY	2002	2002	IPUMS_CENSUS	HHM
41088	PSE	2007	2007	IPUMS_CENSUS	HHM
42432	RWA	2002	2002	IPUMS_CENSUS	HHM
367645	RWA	2012	2012	IPUMS_CENSUS	HHM
43167	SDN	2008	2008	IPUMS_CENSUS	HHM
11661	SLE	2004	2004	IPUMS_CENSUS	HHM
56476	SLV	2007	2007	IPUMS_CENSUS	HHM
106548	SSD	2008	2008	IPUMS_CENSUS	HHM
43231	THA	2000	2000	IPUMS_CENSUS	HHM
294807	TTO	2011	2011	IPUMS_CENSUS	HHM
43212	TZA	2002	2002	IPUMS_CENSUS	HHM
294725	TZA	2012	2012	IPUMS_CENSUS	HHM
43328	UGA	2002	2002	IPUMS_CENSUS	HHM
43412	VEN	2001	2001	IPUMS_CENSUS	HHM
43726	VNM	2009	2009	IPUMS_CENSUS	HHM
43152	ZAF	2001	2001	IPUMS_CENSUS	HHM
43158	ZAF	2007	2007	IPUMS_CENSUS	HHM
227194	ZAF	2011	2011	IPUMS_CENSUS	HHM

151325	ZMB	2000	2000	IPUMS_CENSUS	HHM
151326	ZMB	2010	2010	IPUMS_CENSUS	HHM
367747	ZWE	2012	2012	IPUMS_CENSUS	HHM
34524	IRQ	2006	2007	IRQ/HH_SOCIOECONOMIC_SURVEY	HHM
133219	KEN	2007	2007	KEN/AIDS_INDICATOR_SURVEY	HHM
133304	KEN	2012	2013	KEN/AIDS_INDICATOR_SURVEY	HH
157397	KEN	2010	2010	KEN/CHILD_LABOR_SURVEY	HHM
157635	KEN	2007	2007	KEN/HH_HEALTH_EXPENDITURE_UTILIZATION_SURVEY	HH
7375	KEN	2005	2006	KEN/KIHBS	HH
57990	KEN	2007	2007	KEN/MALRIA_INDICATORY_SURVEY	HH
164729	KHM	2013	2013	KHM/INTERCENSAL_POPULATION_SURVEY	HH
30963	KHM	2003	2005	KHM/SOCIO_ECONOMIC_SURVEY	HH
165631	KHM	2007	2008	KHM/SOCIO_ECONOMIC_SURVEY	HH
31143	KHM	2009	2009	KHM/SOCIO_ECONOMIC_SURVEY	HH
327852	MWI	2016	2017	LSMS_ISA	HHM
3133	COG	2009	2009	DHS_AIS	HH
4837	GUY	2005	2005	DHS_AIS	HH
8906	MOZ	2009	2009	DHS_AIS	HH
157060	MOZ	2015	2015	DHS_AIS	HHM
12630	TZA	2003	2004	DHS_AIS	HH
12644	TZA	2007	2008	DHS_AIS	HH
77395	TZA	2011	2012	DHS_AIS	HH
55973	UGA	2011	2011	DHS_AIS	HH
13544	VNM	2005	2005	DHS_AIS	HH
157018	AFG	2015	2016	DHS	HHM
218555	AGO	2015	2016	DHS	HH
30431	BDI	2010	2011	DHS	HH
286766	BDI	2016	2017	DHS	HHM
18950	BEN	2001	2001	DHS	HH
18959	BEN	2006	2006	DHS	HH
79839	BEN	2011	2012	DHS	HH
19088	BFA	2003	2003	DHS	HH
19133	BFA	2010	2011	DHS	HH
18902	BGD	2004	2004	DHS	HH
18913	BGD	2007	2007	DHS	HH

55956	BGD	2011	2012	DHS	HH
157021	BGD	2014	2014	DHS	HH
19001	BOL	2003	2004	DHS	HH
19016	BOL	2008	2008	DHS	HH
18533	CIV	2011	2012	DHS	HH
19211	CMR	2004	2004	DHS	HH
19274	CMR	2011	2011	DHS	HH
19381	COD	2007	2007	DHS	HH
76878	COD	2013	2014	DHS	HH
19391	COG	2005	2005	DHS	HH
56151	COG	2011	2012	DHS	HH
19359	COL	2000	2000	DHS	HH
19324	COL	2004	2005	DHS	HH
21281	COL	2009	2010	DHS	HH
218566	COL	2015	2016	DHS	HHM
76850	COM	2012	2013	DHS	HH
19444	DOM	2002	2002	DHS	HH
19456	DOM	2007	2007	DHS	HH
77819	DOM	2013	2013	DHS	HH
19511	EGY	2000	2000	DHS	HH
19529	EGY	2003	2003	DHS_INTERIM	HH
19521	EGY	2005	2005	DHS	HH
19539	ERI	2002	2002	DHS	HH
19571	ETH	2000	2000	DHS	HH
19557	ETH	2005	2005	DHS	HH
21301	ETH	2010	2011	DHS	HH
218568	ETH	2016	2016	DHS	HH
19579	GAB	2000	2001	DHS	HH
76706	GAB	2012	2012	DHS	HH
19627	GHA	2003	2003	DHS	HH
21188	GHA	2008	2008	DHS	HH
157027	GHA	2014	2014	DHS	HH
218572	GHA	2017	2017	DHS_SPECIAL	HH
19683	GIN	2005	2005	DHS	HH
69761	GIN	2012	2012	DHS	HH

77384	GMB	2013	2013	DHS	HH
157031	GTM	2014	2015	DHS	HH
21348	GUY	2009	2009	DHS	HH
19728	HND	2005	2006	DHS	HH
95440	HND	2011	2012	DHS	HH
19708	HTI	2000	2000	DHS	HH
19720	HTI	2005	2006	DHS	HH
65118	HTI	2012	2012	DHS	HH
218574	HTI	2016	2017	DHS	HH
20011	IDN	2002	2003	DHS	HH
20021	IDN	2007	2007	DHS	HH
76705	IDN	2012	2012	DHS	HH
19950	IND	1998	2000	DHS	HH
19963	IND	2005	2006	DHS	HH
157050	IND	2015	2016	DHS	HH
20073	JOR	2002	2002	DHS	HH
20083	JOR	2007	2007	DHS	HH
21206	JOR	2009	2009	DHS_INTERIM	HH
77517	JOR	2012	2012	DHS	HH
20145	KEN	2003	2003	DHS	HH
21365	KEN	2008	2009	DHS	HH
157057	KEN	2014	2014	DHS	HH
77518	KGZ	2012	2012	DHS	HH
19156	KHM	2000	2000	DHS	HH
19167	KHM	2005	2006	DHS	HH
30379	KHM	2010	2011	DHS	HH
157024	KHM	2014	2014	DHS	HH
20191	LBR	2006	2007	DHS	HH
77385	LBR	2013	2013	DHS	HH
20167	LSO	2004	2005	DHS	HH
21382	LSO	2009	2010	DHS	HH
157058	LSO	2014	2014	DHS	HH
20361	MAR	2003	2004	DHS	HH
20223	MDG	2003	2004	DHS	HH
21409	MDG	2008	2009	DHS	HH

20315	MLI	2001	2001	DHS	HH
20274	MLI	2006	2006	DHS	HH
77388	MLI	2012	2013	DHS	HH
157061	MMR	2015	2016	DHS	HH
20394	MOZ	2003	2004	DHS	HH
55975	MOZ	2011	2011	DHS	HH
20322	MRT	2000	2001	DHS	HH
20252	MWI	2000	2000	DHS	HH
20263	MWI	2004	2005	DHS	HH
21393	MWI	2010	2010	DHS	HH
218581	MWI	2015	2016	DHS	HH
20417	NAM	2000	2000	DHS	HH
20428	NAM	2006	2007	DHS	HH
150382	NAM	2013	2013	DHS	HH
20499	NER	2006	2006	DHS	HH
74393	NER	2012	2012	DHS	HH
20567	NGA	2003	2003	DHS	HH
21433	NGA	2008	2008	DHS	HH
77390	NGA	2013	2013	DHS	HH
20487	NIC	2001	2001	DHS	HH
20450	NPL	2001	2001	DHS	HH
20462	NPL	2006	2006	DHS	HH
21240	NPL	2011	2011	DHS	HH
286782	NPL	2016	2017	DHS	HHM
20595	PAK	2006	2007	DHS	HH
77521	PAK	2012	2013	DHS	HH
20649	PER	2000	2000	DHS	HH
275090	PER	2003	2008	DHS	HH
270404	PER	2009	2009	DHS	HH
270469	PER	2010	2010	DHS	HH
270470	PER	2011	2011	DHS	HH
270471	PER	2012	2012	DHS	HH
210182	PER	2014	2014	DHS	HHM
21421	PHL	2008	2008	DHS	HH
142943	PHL	2013	2013	DHS	HH

337877	PHL	2017	2017	DHS	HH
44870	PNG	2006	2007	MACRO_DHS	HH
20722	RWA	2000	2000	DHS	HH
20740	RWA	2005	2005	DHS	HH
21222	RWA	2007	2008	DHS_INTERIM	HH
56040	RWA	2010	2011	DHS	HH
157063	RWA	2014	2015	DHS	HH
26855	SEN	2005	2005	DHS	HH
56063	SEN	2010	2011	DHS	HH
111432	SEN	2012	2013	DHS	HH
191270	SEN	2014	2014	DHS	HH
218592	SEN	2015	2015	DHS	HH
286772	SEN	2016	2016	DHS	HH
353526	SEN	2017	2017	DHS	HH
21258	SLE	2008	2008	DHS	HH
131467	SLE	2013	2013	DHS	HH
26866	STP	2008	2009	DHS	HH
20829	SWZ	2006	2007	DHS	HH
157025	TCD	2014	2015	DHS	HH
77515	TGO	2013	2014	DHS	WN
74460	TJK	2012	2012	DHS	HH
341838	TJK	2017	2017	DHS	HH
21274	TLS	2009	2010	DHS	HH
20875	TZA	2004	2005	DHS	HH
21331	TZA	2009	2010	DHS	HH
218593	TZA	2015	2016	DHS	HH
20993	UGA	2000	2001	DHS	HH
21014	UGA	2006	2006	DHS	HH
56021	UGA	2011	2011	DHS	HH
286780	UGA	2016	2016	DHS	HH
112500	YEM	2013	2013	DHS	HH
21102	ZMB	2001	2002	DHS	HH
21117	ZMB	2007	2007	DHS	HH
77516	ZMB	2013	2014	DHS	HH
21163	ZWE	2005	2006	DHS	HH

55992	ZWE	2010	2011	DHS	HH
157066	ZWE	2015	2015	DHS	HH
56099	AFG	2010	2010	DHS_SPECIAL	HH
21173	GHA	2007	2008	DHS_SPECIAL	HH
21173	GHA	2007	2008	DHS_SPECIAL	WN
137351	IDN	2012	2012	DHS_SPECIAL	HH
20040	IDN	2002	2003	MACRO_DHS_SP	HHM
21039	UZB	2002	2002	DHS_SPECIAL	HH
672	AGO	2007	2007	DHS_MIS	HH
56169	AGO	2011	2011	DHS_MIS	HH
108080	BDI	2012	2013	DHS_MIS	HHM
188785	BFA	2014	2014	DHS_MIS	HH
286788	GHA	2016	2016	DHS_MIS	HH
58006	KEN	2010	2010	DHS_MIS	HH
34279	LBR	2008	2009	DHS_MIS	HH
56828	LBR	2011	2011	DHS_MIS	HH
286768	LBR	2016	2016	DHS_MIS	HHM
69806	MDG	2011	2011	DHS_MIS	HH
111438	MDG	2013	2013	DHS_MIS	HH
218580	MDG	2016	2016	DHS_MIS	HH
218587	MLI	2015	2015	DHS_MIS	HH
77387	MWI	2012	2012	DHS_MIS	HH
157059	MWI	2014	2014	DHS_MIS	HH
30991	NGA	2010	2010	DHS_MIS	HH
218590	NGA	2015	2015	DHS_MIS	HH
77391	RWA	2012	2013	DHS_MIS	HH
350836	RWA	2017	2017	DHS_MIS	HHM
11540	SEN	2008	2009	DHS_MIS	HH
286773	SLE	2016	2016	DHS_MIS	HH
359318	TGO	2017	2017	DHS_MIS	HHM
350798	TZA	2017	2017	DHS_MIS	HH
13109	UGA	2009	2010	DHS_MIS	HH
157065	UGA	2014	2015	DHS_MIS	HH
150485	MAR	2009	2010	MAR/HOUSEHOLD_AND_YOUTH_SURVEY	HHM

8684	MEX	2002	2003	MEX/NATIONAL_PERFORMANCE_EVALUATION_SURVEY_ENED	HH
23982	MEX	2006	2006	MEX/SURVEY_DEMOGRAPHIC_DYNAMICS_ENADID	HH
8618	MEX	2005	2006	MEX/SURVEY_HEALTH_AND_NUTRITION_ENSANUT	HH
8618	MEX	2005	2006	MEX/SURVEY_HEALTH_AND_NUTRITION_ENSANUT	HH
8618	MEX	2005	2006	MEX/SURVEY_HEALTH_AND_NUTRITION_ENSANUT	HH
81748	MEX	2011	2012	MEX/SURVEY_HEALTH_AND_NUTRITION_ENSANUT	HH
165610	MEX	2012	2012	MEX/SURVEY_INCOME_AND_HOUSEHOLD_EXPENDITURE_ENIGH	HH
165610	MEX	2012	2012	MEX/SURVEY_INCOME_AND_HOUSEHOLD_EXPENDITURE_ENIGH	HH
93321	MEX	2010	2010	MEX/SURVEY_INCOME_AND_HOUSEHOLD_EXPENDITURE_ENIGH	HH
25358	MEX	2008	2008	MEX/SURVEY_INCOME_AND_HOUSEHOLD_EXPENDITURE_ENIGH	HH
25335	MEX	2006	2006	MEX/SURVEY_INCOME_AND_HOUSEHOLD_EXPENDITURE_ENIGH	HH
25317	MEX	2005	2005	MEX/SURVEY_INCOME_AND_HOUSEHOLD_EXPENDITURE_ENIGH	HH
25293	MEX	2004	2004	MEX/SURVEY_INCOME_AND_HOUSEHOLD_EXPENDITURE_ENIGH	HH
25273	MEX	2002	2002	MEX/SURVEY_INCOME_AND_HOUSEHOLD_EXPENDITURE_ENIGH	HH
25254	MEX	2000	2000	MEX/SURVEY_INCOME_AND_HOUSEHOLD_EXPENDITURE_ENIGH	HH
141910	MMR	2003	2003	MMR/MULTIPLE_INDICATOR_CLUSTER_SURVEY	HH
134132	NAM	2011	2011	NAM/CENSUS	HHM
134371	NAM	2009	2010	NAM/HH_INCOME_AND_EXPENDITURE_SURVEY	HHM
24890	NGA	2006	2007	NGA/GENERAL_HOUSEHOLD_SURVEY	HHM
24915	NGA	2007	2008	NGA/GENERAL_HOUSEHOLD_SURVEY	HHM
151719	NGA	2008	2010	NGA/LIVING_STANDARDS_SURVEY	HHM
126952	NIC	2011	2012	NIC/DHS_ENDESA	HH
9951	PAK	2004	2005	PAK/SOCIAL_AND_LIVING_MEASUREMENT_SURVEY	HHM
265082	PAK	2014	2015	PAK/SOCIAL_AND_LIVING_MEASUREMENT_SURVEY	HHM
303663	PER	2015	2015	PER/DEMOGRAPHIC_AND_FAMILY_HEALTH_SURVEY_ENDES	HH
358824	PER	2017	2017	PER/DEMOGRAPHIC_AND_FAMILY_HEALTH_SURVEY_ENDES	HH
44275	PER	2000	2000	PER/NATIONAL_HH_SURVEY_ENAHO	HH
44315	PER	2000	2000	PER/NATIONAL_HH_SURVEY_ENAHO	HH

44470	PER	2001	2001	PER/NATIONAL_HH_SURVEY_ENAHO	HH
44696	PER	2002	2002	PER/NATIONAL_HH_SURVEY_ENAHO	HH
44748	PER	2003	2004	PER/NATIONAL_HH_SURVEY_ENAHO	HH
49279	PER	2004	2004	PER/NATIONAL_HH_SURVEY_ENAHO	HH
49429	PER	2006	2006	PER/NATIONAL_HH_SURVEY_ENAHO	HH
33702	PER	2009	2009	PER/NATIONAL_HH_SURVEY_ENAHO	HH
33829	PER	2010	2010	PER/NATIONAL_HH_SURVEY_ENAHO	HH
265406	PER	2014	2014	PER/NATIONAL_HH_SURVEY_ENAHO	HH
265409	PER	2015	2015	PER/NATIONAL_HH_SURVEY_ENAHO	HH
283383	PER	2016	2016	PER/NATIONAL_HH_SURVEY_ENAHO	HH
126396	PHL	2011	2011	PHL/FNRI	HH
243566	PRY	2014	2014	PRY/PERMANENT_HH_SURVEY_EPH	HH
243564	PRY	2013	2013	PRY/PERMANENT_HH_SURVEY_EPH	HH
243562	PRY	2012	2012	PRY/PERMANENT_HH_SURVEY_EPH	HH
336799	PRY	2016	2016	PRY/PERMANENT_HH_SURVEY_EPH	HH
286233	PRY	2015	2015	PRY/PERMANENT_HH_SURVEY_EPH	HH
367995	PRY	2017	2017	PRY/PERMANENT_HH_SURVEY_EPH	HH
243548	PRY	2011	2011	PRY/PERMANENT_HH_SURVEY_EPH	HH
243537	PRY	2010	2010	PRY/PERMANENT_HH_SURVEY_EPH	HH
41856	PRY	2009	2009	PRY/PERMANENT_HH_SURVEY_EPH	HH
41851	PRY	2008	2008	PRY/PERMANENT_HH_SURVEY_EPH	HH
41844	PRY	2007	2007	PRY/PERMANENT_HH_SURVEY_EPH	HH
41837	PRY	2006	2006	PRY/PERMANENT_HH_SURVEY_EPH	HH
41830	PRY	2005	2005	PRY/PERMANENT_HH_SURVEY_EPH	HH
41823	PRY	2004	2004	PRY/PERMANENT_HH_SURVEY_EPH	HH
10377	PRY	2003	2003	PRY/PERMANENT_HH_SURVEY_EPH	HH
10373	PRY	2002	2002	PRY/PERMANENT_HH_SURVEY_EPH	HH
10040	PSE	2007	2008	PSE/CENSUS	HHM
264956	IDN	2014	2015	RAND_FLS/IDN	HHM
6464	IDN	2007	2007	RAND_FLS/IDN	HHM
6111	IDN	2000	2000	RAND_FLS/IDN	HH
58185	RWA	2006	2006	RWA/COMPREHENSIVE_FOOD_SECURITY_AND_VULNERABILITY_ASSESSMENT	HH
151436	RWA	2012	2012	RWA/COMPREHENSIVE_FOOD_SECURITY_AND_VULNERABILITY_ASSESSMENT	CH
151437	RWA	2010	2011	RWA/INTEGRATED_LIVING_CONDITIONS_SURVEY_EICV	HHM

58218	RWA	2008	2008	RWA/VISION_2020_UMURENGE_PROGRAM_BASELINE_SURVEY	HHM
30368	SSD	2009	2009	SDN/NATIONAL_BASELINE_HOUSEHOLD_SURVEY	HH
30349	SSD	2009	2009	SDN/NORTH_NATIONAL_BASELINE_HH_SURVEY	HH
165017	SLV	2013	2013	SLV/MULTIPURPOSE_HH_SURVEY_EHPM	HH
238389	SLV	2014	2014	SLV/MULTIPURPOSE_HH_SURVEY_EHPM	HHM
137328	SLV	2012	2012	SLV/MULTIPURPOSE_HH_SURVEY_EHPM	HH
240604	MEX	2014	2014	SURVEY_DEMOGRAPHIC_DYNAMICS_ENADID	HHM
31740	TZA	2000	2001	TZA/HH_BUDGET_SURVEY	HH
31887	TZA	2007	2007	TZA/HH_BUDGET_SURVEY	HH
280228	TZA	2014	2014	TZA/INTEGRATED_LABOR_FORCE_SURVEY_ILFS	HH
23687	UGA	2005	2006	UGA/HOUSEHOLD_SURVEY	HH
56830	AFG	2010	2011	UNICEF_MICS	HH
1981	BDI	2005	2005	UNICEF_MICS	HH
206075	BEN	2014	2014	UNICEF_MICS	HH
1927	BFA	2006	2006	UNICEF_MICS	HH
951	BGD	2006	2006	UNICEF_MICS	HH
151086	BGD	2012	2013	UNICEF_MICS	HH
1089	BLZ	2006	2006	UNICEF_MICS	HH
76699	BLZ	2011	2011	UNICEF_MICS	HH
40028	BTN	2010	2010	UNICEF_MICS	HH
2223	CAF	2006	2006	UNICEF_MICS	HH
82832	CAF	2010	2011	UNICEF_MICS	HH
26433	CIV	2006	2006	UNICEF_MICS	HH
218611	CIV	2016	2016	UNICEF_MICS	HH
2053	CMR	2000	2000	UNICEF_MICS	HH
2063	CMR	2006	2006	UNICEF_MICS	HH
244455	CMR	2014	2014	UNICEF_MICS	HH
26998	COD	2010	2010	UNICEF_MICS	HH
234733	COG	2014	2015	UNICEF_MICS	HH
3114	COM	2000	2000	UNICEF_MICS	HH
125596	CRI	2011	2011	UNICEF_MICS	HH
3404	DJI	2006	2006	UNICEF_MICS	HH
200697	DOM	2014	2014	UNICEF_MICS	HH
210614	DZA	2012	2013	UNICEF_MICS	HH

159617	EGY	2013	2014	UNICEF_MICS	HH
4694	GHA	2006	2006	UNICEF_MICS	HH
160576	GHA	2007	2008	UNICEF_MICS	HH
56241	GHA	2010	2011	UNICEF_MICS	HH
63993	GHA	2011	2011	UNICEF_MICS	HH
303458	GIN	2016	2016	UNICEF_MICS	HH
3935	GMB	2005	2006	UNICEF_MICS	HH
91506	GMB	2010	2010	UNICEF_MICS	HH
4818	GNB	2006	2006	UNICEF_MICS	HH
174049	GNB	2014	2014	UNICEF_MICS	HH
27215	GNB	2010	2010	UNICEF_MICS	WN
4916	GUY	2000	2000	UNICEF_MICS	HH
4926	GUY	2006	2007	UNICEF_MICS	HH
200598	GUY	2014	2014	UNICEF_MICS	HH
7028	IRQ	2006	2006	UNICEF_MICS	HH
76707	IRQ	2011	2011	UNICEF_MICS	HH
141336	JAM	2011	2011	UNICEF_MICS	HH
7387	KEN	2000	2000	UNICEF_MICS	HH
155335	KEN	2007	2007	UNICEF_MICS	HH
7401	KEN	2008	2008	UNICEF_MICS	HH
56420	KEN	2009	2009	UNICEF_MICS	HH
7540	KGZ	2005	2006	UNICEF_MICS	HH
162283	KGZ	2014	2014	UNICEF_MICS	HH
7629	LAO	2006	2006	UNICEF_MICS	HH
103973	LAO	2011	2012	UNICEF_MICS	HH
375362	LAO	2017	2017	UNICEF_MICS	HHM
125594	MDG	2012	2012	UNICEF_MICS	HH
264590	MEX	2015	2015	UNICEF_MICS	HH
270627	MLI	2009	2010	UNICEF_MICS	HH
248224	MLI	2015	2015	UNICEF_MICS	HH
90696	MMR	2009	2010	UNICEF_MICS	HHM
8788	MNG	2000	2000	UNICEF_MICS	HH
8777	MNG	2005	2005	UNICEF_MICS	HH
76704	MNG	2010	2010	UNICEF_MICS	HH
150866	MNG	2013	2013	UNICEF_MICS	HH

189048	MNG	2012	2012	UNICEF_MICS	HH
27031	MOZ	2008	2009	UNICEF_MICS	HH
8115	MRT	2007	2007	UNICEF_MICS	HH
152783	MRT	2011	2011	UNICEF_MICS	HH
7919	MWI	2006	2006	UNICEF_MICS	HH
161662	MWI	2013	2014	UNICEF_MICS	HH
9516	NGA	2007	2007	UNICEF_MICS	HH
76703	NGA	2011	2011	UNICEF_MICS	HH
218613	NGA	2016	2017	UNICEF_MICS	HH
39999	NPL	2010	2010	UNICEF_MICS	HH
162317	NPL	2014	2014	UNICEF_MICS	HH
324470	PRY	2016	2016	UNICEF_MICS	HH
125591	PSE	2010	2010	UNICEF_MICS	HH
161590	PSE	2014	2014	UNICEF_MICS	HH
12243	SDN	2000	2000	UNICEF_MICS	HH
153643	SDN	2010	2010	UNICEF_MICS	HH
200617	SDN	2014	2014	UNICEF_MICS	HH
27044	SEN	2000	2000	UNICEF_MICS	HH
287639	SEN	2015	2016	UNICEF_MICS	HH
11649	SLE	2005	2005	UNICEF_MICS	HH
76700	SLE	2010	2010	UNICEF_MICS	HH
218619	SLE	2017	2017	UNICEF_MICS	HH
200636	SLV	2014	2014	UNICEF_MICS	HH
11774	SOM	2006	2006	UNICEF_MICS	HH
91508	SOM	2011	2011	UNICEF_MICS	HH
91507	SOM	2011	2011	UNICEF_MICS	HH
12232	SSD	2000	2000	UNICEF_MICS	HH
32189	SSD	2010	2010	UNICEF_MICS	HH
27055	STP	2000	2000	UNICEF_MICS	HH
214640	STP	2014	2014	UNICEF_MICS	HH
12289	SUR	2006	2006	UNICEF_MICS	HH
81203	SUR	2010	2010	UNICEF_MICS	HH
12320	SWZ	2000	2000	UNICEF_MICS	HH
30325	SWZ	2010	2010	UNICEF_MICS	HH
200707	SWZ	2014	2014	UNICEF_MICS	HH

12399	SYR	2006	2006	UNICEF_MICS	HH
76701	TCD	2010	2010	UNICEF_MICS	HH
12896	TGO	2006	2006	UNICEF_MICS	HH
40021	TGO	2010	2010	UNICEF_MICS	HH
12732	THA	2005	2006	UNICEF_MICS	HH
148649	THA	2012	2012	UNICEF_MICS	HH
296646	THA	2015	2016	UNICEF_MICS	HH
331377	THA	2016	2016	UNICEF_MICS	HH
12595	TJK	2000	2000	UNICEF_MICS	HH
12608	TJK	2005	2005	UNICEF_MICS	HH
13064	TKM	2006	2006	UNICEF_MICS	HH
264583	TKM	2015	2016	UNICEF_MICS	HH
12950	TTO	2006	2006	UNICEF_MICS	HH
76709	TUN	2011	2012	UNICEF_MICS	HH
13445	UZB	2006	2006	UNICEF_MICS	HH
13719	VNM	2006	2006	UNICEF_MICS	HH
57999	VNM	2010	2011	UNICEF_MICS	HH
152735	VNM	2013	2014	UNICEF_MICS	HH
13816	YEM	2006	2006	UNICEF_MICS	HH
35493	ZWE	2009	2009	UNICEF_MICS	HH
152720	ZWE	2014	2014	UNICEF_MICS	HH
151568	AGO	2011	2011	WB_CWIQ	HHM
1855	BFA	2003	2003	WB_CWIQ	HH
22950	BFA	2005	2005	WB_CWIQ	HH
18499	BFA	2007	2007	WB_CWIQ	HH
23017	GHA	2003	2003	WB_CWIQ	HH
9522	NGA	2006	2006	WB_CWIQ	HH
31797	TZA	2005	2005	WB_CWIQ	HH
31831	TZA	2006	2007	WB_CWIQ	HH
31786	TZA	2004	2004	WB_CWIQ	HHM
4679	GHA	2005	2006	WB_LSMS	HHM
165101	GHA	2012	2013	WB_LSMS	HH
46317	MWI	2004	2005	WB_LSMS	HH
9422	NIC	2001	2001	WB_LSMS	HH
44645	NIC	2005	2005	WB_LSMS	HH

46480	NPL	2003	2004	WB_LSMS	HH
94168	NPL	2010	2011	WB_LSMS	HHM
10224	PAN	2003	2003	WB_LSMS	HH
46517	PAN	2008	2008	WB_LSMS	HH
12863	TLS	2001	2001	WB_LSMS	HHM
46682	TLS	2007	2008	WB_LSMS	HH
235215	ETH	2013	2014	WB_LSMS_ISA	HH
286657	ETH	2015	2016	WB_LSMS_ISA	HH
260407	MLI	2014	2015	WB_LSMS_ISA	HHM
224223	MWI	2013	2013	WB_LSMS_ISA	HH
94140	NER	2011	2012	WB_LSMS_ISA	HH
27297	TZA	2008	2009	WB_LSMS_ISA	HH
81005	TZA	2010	2011	WB_LSMS_ISA	HH
224096	TZA	2012	2013	WB_LSMS_ISA	HHM
311265	TZA	2014	2016	WB_LSMS_ISA	HHM
299064	LKA	2012	2012	WB_STEP_HH_SURVEY	HH
299045	VNM	2012	2012	WB_STEP_HH_SURVEY	HH
111485	GHA	2007	2008	WHO_SAGE	HH
111488	ZAF	2007	2008	WHO_SAGE	HH
244480	YEM	2014	2014	YEM/COMPREHENSIVE_FOOD_SECURITY_SURVEY_CFSS	HH
22882	YEM	2005	2006	YEM/HH_BUDGET_SURVEY	HH
249499	YEM	2012	2013	YEM/NATIONAL_SOCIAL_PROTECTION_MONITORING_SURVEY_NSPMS	HHM
12146	ZAF	2011	2011	ZAF/CENSUS	HH
280803	ZAF	2016	2016	ZAF/COMMUNITY_SURVEY	HHM
25100	ZAF	2007	2007	ZAF/COMMUNITY_SURVEY	HHM
115481	ZAF	2002	2002	ZAF/HH_SURVEY	HHM
11787	ZAF	2003	2003	ZAF/HH_SURVEY	HHM
11788	ZAF	2004	2004	ZAF/HH_SURVEY	HHM
11789	ZAF	2005	2005	ZAF/HH_SURVEY	HHM
115486	ZAF	2006	2006	ZAF/HH_SURVEY	HHM
11790	ZAF	2007	2007	ZAF/HH_SURVEY	HHM
115488	ZAF	2008	2008	ZAF/HH_SURVEY	HHM
115489	ZAF	2009	2009	ZAF/HH_SURVEY	HH
115490	ZAF	2010	2010	ZAF/HH_SURVEY	HH

115491	ZAF	2011	2011	ZAF/HH_SURVEY	HH
238485	ZAF	2014	2014	ZAF/HH_SURVEY	HH
265084	ZAF	2015	2015	ZAF/HH_SURVEY	HH
238483	ZAF	2013	2013	ZAF/HH_SURVEY	HH
317089	ZAF	2016	2016	ZAF/HH_SURVEY	HH
11848	ZAF	2005	2006	ZAF/INCOME_AND_EXPENDITURE_SURVEY	HH
265153	ZAF	2014	2015	ZAF/NATIONAL_INCOME_DYNAMICS_STUDY	HH
369644	ZAF	2017	2017	ZAF/NATIONAL_INCOME_DYNAMICS_STUDY	HH
14027	ZMB	2002	2003	ZMB/LCMS	HHM
14063	ZMB	2004	2005	ZMB/LCMS	HH
14105	ZMB	2006	2006	ZMB/LCMS	HH
58660	ZMB	2010	2010	ZMB/LCMS	HH
286783	PAK	2017	2018	DHS	HH
237943	IDN	2015	2015	IDN/INTERCENSAL_POPULATION_SURVEY_SUPAS	HH
165186	IDN	2014	2014	IDN/SOCIOECONOMIC_SURVEY_SUSENAS	HH
238332	IDN	2015	2015	IDN/SOCIOECONOMIC_SURVEY_SUSENAS	HH
282087	IDN	2016	2016	IDN/SOCIOECONOMIC_SURVEY_SUSENAS	HH
395694	IDN	2017	2017	IDN/SOCIOECONOMIC_SURVEY_SUSENAS	HH
356955	JOR	2017	2018	DHS	HH
218565	BEN	2017	2018	DHS	HHM
369294	CHN	2016	2016	CHINESE_FAMILY_PANEL_STUDIES_CFPS/2016	HH
286781	IDN	2017	2017	DHS	HH
393799	ZAF	2016	2016	ZAF/HH_SURVEY	HH
364181	ECU	2013	2014	ECU_LIVING_CONDITIONS_SURVEY_ECV	HH
141521	PAK	2011	2011	PAK_NATIONAL_NUTRITION	HHM
400338	LBR	2016	2017	LBR_HH_INCOME_EXPENDITURE	HHM
385708	IRQ	2018	2018	UNICEF_MICS	HH
165290	BTN	2012	2013	BTN/NATIONAL_HEALTH_SURVEY	HH
31050	KHM	2006	2007	KHM/SOCIO_ECONOMIC_SURVEY	HHM
24143	SDN	2006	2006	ARAB_LEAGUE_PAPFAM	HH
24143	SSD	2006	2006	ARAB_LEAGUE_PAPFAM	HH
327591	TZA	2016	2017	ICAP_PHIA	HH
151566	AGO	2005	2006	WB_CWIQ	HHM
287629	MWI	2015	2016	MWI_ICAP_PHIA	HH
409558	TUN	2018	2018	UNICEF_MICS	HH

413556	BFA	2017	2018	DHS_MIS	HH
408226	KGZ	2018	2018	UNICEF_MICS	HH
415531	SWZ	2016	2017	ICAP_PHIA	HH
400526	PAK	2017	2018	UNICEF_MICS	HH
413934	MOZ	2018	2018	DHS_MIS	HH
12865	TLS	2002	2002	UNICEF_MICS	HH
21622	GHA	2003	2003	WHO_WHS	HHM
77393	SLE	2013	2013	DHS_MIS	HH
27885	ZAF	2008	2008	ZAF/NATIONAL_INCOME_DYNAMICS_STUDY	HHM
60405	CHN	2007	2010	WHO_SAGE	HHM
339653	MEX	2016	2016	MEX/SURVEY_INCOME_AND_HOUSEHOLD_EXPENDITURE_ ENIGH	HH
326837	LKA	2016	2016	DHS	HHM
18815	LKA	2006	2007	DHS	HHM
90707	LKA	2000	2000	MACRO_DHS	HH
396957	GIN	2018	2018	DHS	HH
398033	MLI	2018	2018	DHS	HH
81416	IRN	2010	2010	IRN/MULTIPLE_INDICATOR_DEMOGRAPHIC_HEALTH_SURV EY	HH
264910	BLZ	2015	2016	UNICEF_MICS	HH
267343	MRT	2015	2015	UNICEF_MICS	HH
286769	MWI	2017	2017	DHS_MIS	HH
286785	TLS	2016	2016	DHS	HH
336042	MNG	2016	2016	UNICEF_MICS	HH
335994	MNG	2016	2016	UNICEF_MICS	HH
332558	TTO	2011	2011	UNICEF_MICS	HH
22108	BTN	2007	2007	BTN/LIVING_STANDARDS	HH
404407	MEX	2018	2018	MEX/ENADID	HH
400219	NPL	2016	2016	NPL_RISK_VULNERABILITY_ASSESSMENT	HHM
403435	BOL	2012	2012	BOL/HOUSEHOLD_SURVEY	HH
124467	COL	2004	2005	COL/NATIONAL_SURVEY_ON_NUTRITION_SITUATION_ENSI N	HHM
407869	PER	2018	2018	PER/DEMOGRAPHIC_AND_FAMILY_HEALTH_SURVEY_END ES	HH
403655	PRY	2018	2018	PRY/PERMANENT_HOUSHEOLD_SURVEY_EPH	HH
148346	BOL	2009	2009	BOL/HH_SURVEY	HHM

413667	PER	2007	2008	DHS	HH
413666	PER	2003	2006	DHS	HH
400535	TZA	2014	2015	TZA/MEASURING_LIVING_STANDARDS_WITHIN_CITIES	HHM
148345	BOL	2008	2008	BOL/HH_SURVEY	HHM
424884	GMB	2018	2018	UNICEF_MICS	HH
317305	THA	2015	2016	UNICEF_MICS	HH
317305	THA	2015	2016	UNICEF_MICS	HH
317305	THA	2015	2016	UNICEF_MICS	HH
317305	THA	2015	2016	UNICEF_MICS	HH
317305	THA	2015	2016	UNICEF_MICS	HH
317305	THA	2015	2016	UNICEF_MICS	HH
317305	THA	2015	2016	UNICEF_MICS	HH
317305	THA	2015	2016	UNICEF_MICS	HH
317305	THA	2015	2016	UNICEF_MICS	HH
317305	THA	2015	2016	UNICEF_MICS	HH
317305	THA	2015	2016	UNICEF_MICS	HH
317305	THA	2015	2016	UNICEF_MICS	HH
317305	THA	2015	2016	UNICEF_MICS	HH
317305	THA	2015	2016	UNICEF_MICS	HH
426238	PNG	2016	2018	DHS	HH
399853	MDG	2018	2018	UNICEF_MICS	HH
427952	MNG	2018	2018	UNICEF_MICS	HH
427983	SUR	2018	2018	UNICEF_MICS	HH
431951	ZWE	2019	2019	UNICEF_MICS	HH
284177	ZMB	2015	2015	ZMB/LCMS	HHM

Supplementary Table 5: Covariates used in mapping

A variety of socioeconomic and environmental variables were used to estimate cooking fuel type use. Where available, the finest spatiotemporal resolution of gridded datasets was used. In addition to the geospatial covariates detailed below, the last three covariates listed are country level covariates: *healthcare access and quality index, lag distributed income per capita,* and *proportion of the population with access to adequate sanitation.*

Covariate	Temporal Resolution	Justification	Source	Reference
**Distance from rivers or lakes >= 50 sq. km**	Static	Related to availability of biofuels	Natural Earth Data (derived)	Available at: http://www.naturalearthdata.com/downloads/10m-physical-vectors/10m-rivers-lake-centerlines/ AND http://www.worldwildlife.org/pages/global-lakes-and-wetlands-database
**Night-time lights**	Annual	Related to development	NOAA DMSP satellite program (derived)	Available at: https://www.ngdc.noaa.gov/eog/dmsp/downloadV4composites.html
**Elevation**	Static	Related to availability of biofuels	NOAA GlOBE	Available at: https://www.ngdc.noaa.gov/mgg/topo/gltiles.html
**Agricultural land**	Static	Related to availability of biofuels	Earth Stat Project	Ramankutty, N., Evan, A.T., Monfreda, C., & Foley, J.A. Farming the planet. Part 1: Geographic distribution of global agricultural lands in the year 2000. Global Biogeochemical Cycle 22, GB1003 (2008), doi: 10.1029/2007GB002952.<br><br>Available at: http://www.earthstat.org/data-download/
**Enhanced vegetation index**	Annual	Related to availability of biofuels	MODIS, pre-processed by Oxford	Weiss, D. J. et al. An effective approach for gapfilling continental scale remotely sensed timeseries. Isprs J. Photogramm. Remote Sens. 98, 106–118 (2014).<br><br>USGS & NASA. Vegetation indices 16-Day L3 global 500m MOD13A1 dataset. Available at: https://lpdaac.usgs.gov/dataset_discovery/modis/modis_products_table/mcd43b4. (Accessed: 25th July 2017) Huete, A., Justice, C. & van Leeuwen, W. MODIS vegetation index (MOD 13) algorithm theoretical basis document. (1999).
**Fertility**	Annual	Related to development	WorldPop (derived)	Lloyd, C. T., Sorichetta, A. & Tatem, A. J. High resolution global gridded data for use in population studies. Sci. Data 4, sdata20171 (2017).<br><br>Available at: http://www.worldpop.org.uk/data/get_data/. (Accessed: 25th July 2017)
**Urban or rural**	Annual	Related to availability of clean fuels and technologies	European Commission/GHS	Pesaresi, M. et al. Operating procedure for the production of the Global Human Settlement Layer from Landsat data of the epochs 1975, 1990, 2000, and 2014. JRC Technical Report EUR 27741 EN; doi:10.2788/253582 (online)<br><br>Available at: http://ghsl.jrc.ec.europa.eu/data.php
**Nutrient yield**	Static	Related to availability of biofuels	Herrero et al.	Herrero, M. et al. Farming and the geography of nutrient production for human use: a transdisciplinary analysis. Lancet Planet. Health 1, e33–e42 (2017).

**Irrigation**	Static	Related to availability of biofuels	University of Frankfurt and FAO	Siebert, S., Doll, P., Hoogeveen, J., Faures, J.-M., Frenken, K., & Feick, S. Development and validation of the global map of irrigation areas. Hydrology and Earth System Sciences 9, 535-547 (2005).<br><br>Goethe-Universität. Generation of a digital global map of irrigation areas. Available at: https://www.unifrankfurt.de/45218039/Global_Irrigation_Map. (Accessed: 25th July 2017) Also from: http://www.fao.org/nr/water/aquastat/irrigationmap/index10.stm
**Land cover**	Annual	Related to availability of clean fuels and technologies	MODIS	Available at: https://lpdaac.usgs.gov/dataset_discovery/modis/modis_products_table/mcd12q1
**Average land surface temperature**	Annual	Related to availability of biofuels	MODIS	Weiss, D. J. et al. An effective approach for gapfilling continental scale remotely sensed timeseries. Isprs J. Photogramm. Remote Sens. 98, 106–118 (2014).<br><br>Available at: https://modis.gsfc.nasa.gov/data/dataprod/mod11.php

Covariate	Temporal Resolution	Justification	Source	Reference
**Precipitation**	Annual	Related to availability of biofuels	Beck et al.	Beck, H.E., A.I.J.M. van Dijk, V. Levizzani, J. Schellekens, D.G. Miralles, B. Martens, & A. de Roo. MSWEP: 3-hourly 0.25 global gridded precipitation (1979-2015) by merging gauge, satellite, and reanalysis data. Hydrology and Earth System Sciences 21(1), 589-615 (2017).<br><br>Available at: https://data.princetonclimate.com/opendap
**Tassled cap brightness**	Annual	Related to availability of biofuels	MODIS	Lobser, S.E. & Cohen, W.B. MODIS tasselled cap: land cover characteristics expressed through transformed MODIS data. International Journal of Remote Sensing 28(22), 5079-5101 (2007).<br><br>C. Schaaf, Z. Wang. (2015). MCD43A1 MODIS/Terra+Aqua BRDF/Albedo Model Parameters Daily L3 Global - 500m V006. NASA EOSDIS Land Processes DAAC. http://doi.org/10.5067/MODIS/MCD43A1.006<br>Available at: https://modis.gsfc.nasa.gov/data/dataprod/mod43.php
**Tassled cap wetness**	Annual	Related to availability of biofuels	MODIS	Lobser, S.E. & Cohen, W.B. MODIS tasselled cap: land cover characteristics expressed through transformed MODIS data. International Journal of Remote Sensing 28(22), 5079-5101 (2007).<br><br>C. Schaaf, Z. Wang. (2015). MCD43A1 MODIS/Terra+Aqua BRDF/Albedo Model Parameters Daily L3 Global - 500m V006. NASA EOSDIS Land Processes DAAC. http://doi.org/10.5067/MODIS/MCD43A1.006<br>Available at: https://modis.gsfc.nasa.gov/data/dataprod/mod43.php

**Population**	Annual	Related to development	WorldPop	Lloyd, C. T., Sorichetta, A. & Tatem, A. J. High resolution global gridded data for use in population studies. Sci. Data 4, sdata20171 (2017).<br><br>World Pop. Get data. Available at: http://www.worldpop.org.uk/data/get_data/. (Accessed: 25th July 2017)
**Healthcare access and quality index**	Annual	Related to development	Barber et al.	Barber, R. M. et al. Healthcare Access and Quality Index based on mortality from causes amenable to personal health care in 195 countries and territories, 1990-2015: a novel analysis from the Global Burden of Disease Study 2015. Lancet 390, 231-266 (2017).
**Lag distributed income per capita**	Annual	Related to development	Global Burden of Disease Study	Institute for Health Metrics and Evaluation (IHME). Findings from the Global Burden of Disease Study 2017. Seattle, WA: IHME, 2018.
**Concentration of Particulate Matter 2.5**	Annual	Related to target indicator	van Donkelaar et al (derived)	van Donkelaar, A., Martin, R.V., Brauer, M., & Boys, B.L. Use of Satellite Observations for Long-Term Exposure Assessment of Global Concentrations of Fine Particulate Matter. *Environmental Health Perspectives***123**(2), 135-143 (2015). Available at: https://ehp.niehs.nih.gov/1408646/#tab1
**Deforestation**	Annual	Related to availability of biofuels	University of Maryland's Global Forest Change project, based on Landsat data archive	Hansen, M. C. *et al.* High-Resolution Global Maps of 21st-Century Forest Cover Change. *Science*. **342**, 850-853 (2013).
**Landcover classes**	Annual	Related to development	ESA-CCI project	Available at: https://cds.climate.copernicus.eu/cdsapp#!/dataset/satellite-land-cover?tab=overview

Supplementary Table 6a-b: Covariates used in ensemble modelling via stacked generalisation, stratified by modelling region

Each ‘TRUE’ indicates that a specific covariate was used in the model for a specific modelling region. Table **a)** presents the first 10 modelling regions. Table **b)** presents the remaining 11 modelling regions.

a)

Covariate	Andean South America	Central America and the Caribbean	China	Central Sub-Saharan Africa	Eritrea, Djibouti, Yemen	Eastern Sub-Saharan Africa	Middle East	Mongolia	Nigeria
**Travel time to nearest settlement**	TRUE	TRUE	TRUE	TRUE	TRUE	TRUE	TRUE	TRUE	TRUE
**aridity**	TRUE	FALSE	TRUE	TRUE	FALSE	TRUE	TRUE	FALSE	FALSE
**Diurnal temperature range**	TRUE	FALSE	TRUE	TRUE	TRUE	TRUE	TRUE	TRUE	FALSE
**Frost day frequency**	TRUE	TRUE	FALSE	TRUE	TRUE	TRUE	TRUE	FALSE	FALSE
**Potential Evapotranspiration**	TRUE	TRUE	TRUE	FALSE	TRUE	TRUE	FALSE	TRUE	FALSE
**Average daily mean temperature**	FALSE	FALSE	FALSE	TRUE	FALSE	FALSE	FALSE	FALSE	TRUE
**deforestation**	TRUE	TRUE	TRUE	TRUE	FALSE	TRUE	FALSE	TRUE	TRUE
**Dependency ratio of dependents to working age adults**	FALSE	FALSE	TRUE	TRUE	TRUE	TRUE	TRUE	TRUE	TRUE
**Distance from rivers or lakes >= 50 sq km**	TRUE	TRUE	TRUE	TRUE	FALSE	TRUE	TRUE	TRUE	TRUE
**Nighttime lights**	FALSE	TRUE	TRUE	FALSE	TRUE	FALSE	TRUE	FALSE	TRUE
**elevation**	FALSE	TRUE	TRUE	FALSE	FALSE	TRUE	TRUE	TRUE	TRUE
**Landcover classes**	TRUE	TRUE	TRUE	TRUE	TRUE	TRUE	TRUE	TRUE	TRUE
**escroplandarea**	TRUE	TRUE	TRUE	TRUE	TRUE	TRUE	TRUE	TRUE	TRUE
**evi_v6**	TRUE	TRUE	TRUE	TRUE	TRUE	TRUE	TRUE	TRUE	FALSE
**fertility**	TRUE	TRUE	FALSE	TRUE	TRUE	TRUE	TRUE	TRUE	FALSE
**Urbanicity**	TRUE	TRUE	TRUE	TRUE	TRUE	TRUE	TRUE	TRUE	TRUE
**haqi**	FALSE	FALSE	TRUE	FALSE	FALSE	TRUE	TRUE	TRUE	TRUE
**nutrient yield**	TRUE	TRUE	TRUE	TRUE	TRUE	TRUE	TRUE	TRUE	FALSE
**irrigation**	TRUE	TRUE	TRUE	TRUE	TRUE	TRUE	TRUE	TRUE	TRUE

**landcover**	TRUE	TRUE	TRUE	FALSE	TRUE	TRUE	TRUE	TRUE	TRUE
**ldi_pc**	TRUE	TRUE	FALSE	TRUE	FALSE	TRUE	TRUE	FALSE	TRUE
**Average Land Surface Temperature**	TRUE	TRUE	FALSE	FALSE	TRUE	FALSE	FALSE	FALSE	TRUE
**Precipitation**	FALSE	TRUE	TRUE	TRUE	TRUE	TRUE	TRUE	TRUE	TRUE
**Concentration of Particulate Matter 2.5**	TRUE	TRUE	TRUE	TRUE	TRUE	TRUE	TRUE	TRUE	FALSE
**tcb_v6**	TRUE	TRUE	TRUE	TRUE	TRUE	FALSE	TRUE	TRUE	TRUE
**tcw_v6**	TRUE	FALSE	TRUE	FALSE	FALSE	TRUE	FALSE	TRUE	FALSE
**Population**	TRUE	TRUE	TRUE	TRUE	TRUE	TRUE	TRUE	TRUE	TRUE

b)

Covariate	North Africa	Oceania	Southeast Asia	South Asia	Southern Sub-Saharan Africa	Central Asia	Tropical South America	Western Sub-Saharan Africa	South Africa
**Travel time to nearest settlement**	TRUE	TRUE	TRUE	TRUE	TRUE	TRUE	TRUE	TRUE	TRUE
**aridity**	FALSE	TRUE	TRUE	FALSE	FALSE	TRUE	FALSE	FALSE	FALSE
**Diurnal temperature range**	TRUE	FALSE	TRUE	TRUE	TRUE	TRUE	TRUE	FALSE	FALSE
**Frost day frequency**	TRUE	TRUE	TRUE	TRUE	TRUE	FALSE	TRUE	TRUE	TRUE
**Potential Evapotranspiration**	TRUE	TRUE	TRUE	TRUE	TRUE	FALSE	TRUE	FALSE	FALSE
**Average daily mean temperature**	FALSE	TRUE	FALSE	FALSE	FALSE	FALSE	TRUE	TRUE	FALSE
**deforestation**	FALSE	TRUE	TRUE	TRUE	TRUE	FALSE	TRUE	TRUE	TRUE
**Dependency ratio of dependents to working age adults**	FALSE	FALSE	TRUE	TRUE	FALSE	TRUE	FALSE	TRUE	TRUE
**Distance from rivers or lakes >= 50 sq km**	TRUE	TRUE	TRUE	TRUE	TRUE	TRUE	TRUE	TRUE	TRUE
**Nighttime lights**	TRUE	FALSE	TRUE	TRUE	TRUE	FALSE	TRUE	FALSE	TRUE

**elevation**	TRUE	TRUE	TRUE	TRUE	TRUE	TRUE	TRUE	TRUE	TRUE
**Landcover classes**	TRUE	TRUE	TRUE	TRUE	TRUE	TRUE	TRUE	TRUE	TRUE
**escroplandarea**	TRUE	TRUE	TRUE	TRUE	TRUE	TRUE	TRUE	TRUE	TRUE
**evi_v6**	FALSE	TRUE	TRUE	TRUE	TRUE	TRUE	TRUE	FALSE	TRUE
**fertility**	TRUE	TRUE	TRUE	TRUE	TRUE	TRUE	TRUE	TRUE	TRUE
**Urbanicity**	TRUE	TRUE	TRUE	TRUE	TRUE	TRUE	TRUE	TRUE	TRUE
**haqi**	TRUE	TRUE	FALSE	TRUE	TRUE	FALSE	TRUE	TRUE	FALSE
**nutrient yield**	TRUE	TRUE	TRUE	TRUE	TRUE	TRUE	TRUE	TRUE	TRUE
**irrigation**	TRUE	TRUE	TRUE	TRUE	TRUE	TRUE	TRUE	TRUE	TRUE
**landcover**	FALSE	FALSE	TRUE	FALSE	TRUE	TRUE	FALSE	TRUE	TRUE
**ldi_pc**	FALSE	TRUE	TRUE	TRUE	FALSE	TRUE	FALSE	TRUE	TRUE
**Average Land Surface Temperature**	FALSE	FALSE	FALSE	FALSE	FALSE	FALSE	FALSE	FALSE	TRUE
**Precipitation**	TRUE	TRUE	TRUE	TRUE	TRUE	TRUE	TRUE	TRUE	TRUE
**Concentration of Particulate Matter 2.5**	TRUE	TRUE	TRUE	TRUE	TRUE	TRUE	TRUE	TRUE	TRUE
**tcb_v6**	FALSE	FALSE	FALSE	TRUE	TRUE	TRUE	TRUE	TRUE	TRUE
**tcw_v6**	TRUE	TRUE	TRUE	TRUE	FALSE	TRUE	FALSE	FALSE	FALSE
**Population**	TRUE	TRUE	TRUE	TRUE	TRUE	TRUE	TRUE	TRUE	TRUE

Supplementary Table 7: Fitted parameters by region for model of Solid Fuel Use

Lower, median, and upper quantiles (0.025%, 0.50%, 0.975%) are displayed for the main parameters by region. The first four columns provide information on the fixed effects: the intercept (int) and the covariates (gam, gbm, and enet) corresponding to the predicted ensemble rasters. Fitted values for the spatio-temporal field hyperparameters and the precisions (inverse variance) for our random effects are shown in the next four columns.

	Quantiles	int	gam	gbm	xgboost	Nominal Range	Nominal Variance	Ar1 ρ	precis	Country Random Effect Precision
**Andean South America**	0.025	-1.58485	-0.00068	0.08620	0.83292	0.84010	0.87280	0.59480	2.20950	0.35370
	0.5	-0.86580	0.02452	0.11619	0.85927	0.99670	0.93520	0.60280	3.19800	1.39030
	0.975	-0.14735	0.04972	0.14615	0.88561	1.13830	0.97870	0.60830	5.19730	4.70690
**Central America and the Caribbean**	0.025	-0.63052	0.04751	0.10272	0.69755	1.12050	0.93050	0.43840	2.75290	0.63980
	0.5	-0.11545	0.09848	0.16087	0.74063	1.31580	0.98090	0.44920	4.14210	1.76390
	0.975	0.39920	0.14940	0.21901	0.78367	1.47420	1.04530	0.46810	6.89890	4.82600
**China**	0.025	-0.33666	-0.09953	-0.37279	1.18108	5.45100	0.29750	4153.28410	12.54080	NA
	0.5	-0.12063	-0.01129	-0.25980	1.27109	7.90640	0.40100	20603.90910	57.67960	NA
	0.975	0.09519	0.07690	-0.14714	1.36121	12.50670	0.57670	91703.73690	220.36210	NA
**Central Sub-Saharan Africa**	0.025	1.38080	-0.03779	-0.31687	1.08055	2.85840	1.17350	0.71050	1.37830	0.32300
	0.5	2.12847	0.02503	-0.20675	1.18151	3.72010	1.44100	0.76220	3.03750	1.75460
	0.975	2.87562	0.08836	-0.09688	1.28240	4.97860	1.81470	0.79540	6.77940	8.64240
**Eritrea, Djibouti, and Yemen**	0.025	-1.92666	-0.03216	-0.51720	0.78475	2.56670	1.23210	0.14830	1.18240	1163.70890
	0.5	-1.05112	0.17834	-0.25479	1.07615	4.09890	1.62410	0.15490	10.72630	16039.60500
	0.975	-0.17760	0.38971	0.00724	1.36722	6.72600	2.21540	0.16470	46.47140	76312.09290
**Eastern Sub-Saharan Africa**	0.025	1.65691	0.03735	0.17986	0.67583	1.12480	0.76090	0.44880	0.89430	1.33090
	0.5	1.97228	0.06752	0.21961	0.71273	1.19110	0.78280	0.45640	1.14240	5.66250
	0.975	2.28739	0.09800	0.25933	0.74953	1.28730	0.81840	0.46060	1.37570	26.12950
**Middle East**	0.025	-3.74763	-0.07895	0.53827	0.24256	1.84990	2.17190	0.77020	0.03510	1139.92650
	0.5	-2.51122	-0.03559	0.66598	0.36715	2.25990	2.53560	0.84960	0.09680	14124.22670
	0.975	-1.27655	0.01337	0.79359	0.49137	2.59610	2.82120	0.90710	0.19890	70417.66600
**Mongolia**	0.025	0.43626	0.01661	-0.93653	1.53200	1.97440	0.71600	0.67810	3.03670	NA
	0.5	0.80808	0.11109	-0.78340	1.67242	3.11170	0.91340	0.80250	9.94220	NA
	0.975	1.17972	0.20572	-0.63059	1.81264	4.65920	1.17310	0.87440	21.42290	NA
**Nigeria**	0.025	0.94962	0.12214	-0.87223	1.36671	1.02520	0.73130	0.47810	1.58970	NA
	0.5	1.31404	0.21557	-0.71940	1.50385	1.23430	0.81860	0.49150	3.17030	NA

	Quantiles	int	gam	gbm	xgboost	Nominal Range	Nominal Variance	Ar1 ρ	precis	Country Random Effect Precision
	0.975	1.67817	0.30897	-0.56671	1.64086	1.51260	0.93390	0.50170	7.06070	NA
**North Africa**	0.025	-2.60989	0.02938	0.32645	0.55286	3.44670	1.44620	1.07860	0.00860	1279.38900
	0.5	-1.39878	0.04992	0.36344	0.58641	4.22000	1.70570	1.13920	0.06330	12656.89930
	0.975	-0.18897	0.07114	0.40023	0.61993	5.50710	2.09670	1.17960	0.18020	65596.20270
**Oceania**	0.025	0.20310	-0.01904	-0.00413	0.94406	1.07990	0.89370	0.50410	0.50370	1295.97220
	0.5	0.62047	0.00064	0.02642	0.97293	1.17200	0.92120	0.50890	0.95770	12718.42720
	0.975	1.03753	0.02035	0.05693	1.00178	1.24950	0.95900	0.51610	3.03050	69850.65680
**Southeast Asia**	0.025	0.26101	0.17272	-0.04960	0.68451	1.32550	0.82130	0.50130	0.08010	1269.42210
	0.5	0.78153	0.24449	0.01628	0.74068	1.57150	0.89440	0.51790	0.36290	13665.42090
	0.975	1.30165	0.31134	0.08317	0.79732	1.78310	0.96770	0.54250	0.76430	69249.11850
**South Asia**	0.025	0.93389	-0.01766	-1.44295	2.26529	0.83740	0.97750	0.30960	0.41740	2039.97530
	0.5	1.38598	0.04739	-1.37639	2.32899	0.95310	0.99930	0.31370	0.57560	14399.55650
	0.975	1.83772	0.11239	-1.30989	2.39264	1.02760	1.03390	0.31700	0.82640	62740.85950
**Southern Sub-Saharan Africa**	0.025	0.07039	0.44656	-0.73129	0.91923	0.45910	0.93520	0.32540	0.75520	1212.84440
	0.5	0.41526	0.55710	-0.60577	1.04872	0.62710	0.99640	0.33630	1.47970	12216.45070
	0.975	0.75985	0.66760	-0.48039	1.17807	0.77600	1.04740	0.34450	2.52000	66768.69320
**Central Asia**	0.025	-0.93006	-0.08087	-0.38585	1.22377	10.86020	1.16420	0.17930	0.75020	652.34450
	0.5	-0.27388	0.00292	-0.30124	1.29824	12.00080	1.28420	0.19010	0.87470	7236.75560
	0.975	0.38177	0.08674	-0.21671	1.37265	14.93530	141870.26620	195.34510	1.19530	91991.31560
**Tropical South America**	0.025	-0.31025	0.14782	0.17325	0.62631	2.49180	0.75620	17.62900	5.89530	0.23750
	0.5	0.51561	0.16671	0.19252	0.64076	2.70010	0.80770	18.21250	8.87290	1.01690
	0.975	1.34085	0.18559	0.21178	0.65520	2.97310	0.87270	19.09090	14.03920	3.05940
**Western-Sub Saharan Africa**	0.025	1.08673	-0.00911	0.26714	0.58305	1.93070	0.77510	0.52940	0.81450	1329.71270
	0.5	1.34195	0.03971	0.32913	0.63044	2.36570	0.83890	0.53940	1.08000	13907.71210
	0.975	1.59697	0.09127	0.39009	0.67779	2.70350	0.90480	0.54600	1.42260	72484.39230
**South Africa**	0.025	-0.55307	-0.04261	0.42272	0.54545	0.84230	0.46760	3.30410	14.98960	NA
	0.5	-0.42193	-0.01947	0.45087	0.56860	1.03310	0.51830	3.36150	19.83510	NA

	Quantiles	int	gam	gbm	xgboost	Nominal Range	Nominal Variance	Ar1 ρ	precis	Country Random Effect Precision
	0.975	-0.29090	0.00366	0.47899	0.59173	1.24440	0.58990	3.42720	30.36190	NA

Supplementary Table 8: Predictive metrics for SFU, aggregated to the national level (Admin 0)

YEAR	OOS	Median SS	Mean err.	RMSE	Corr.	95% Cov.
2000	FALSE	74,345.660	-0.029	0.077	0.928	0.944
2005	FALSE	84,999.073	0.000	0.052	0.986	0.957
2010	FALSE	110,729.798	-0.038	0.043	0.998	0.853
2015	FALSE	84,022.000	-0.071	0.099	0.971	0.736
2018	FALSE	96,822.000	-0.014	0.080	0.976	0.936
2000	TRUE	74,345.660	-0.025	0.076	0.928	0.944
2005	TRUE	84,926.073	-0.011	0.056	0.985	0.932
2010	TRUE	110,729.798	-0.039	0.044	0.998	0.803
2015	TRUE	84,022.000	-0.101	0.118	0.975	0.745
2018	TRUE	96,822.000	-0.029	0.073	0.983	0.946

Supplementary Table 9: Predictive metrics for SFU, aggregated to the first administrative level (Admin 1)

YEAR	OOS	Median SS	Mean err.	RMSE	Corr.	95% Cov.
2000	FALSE	10,066.000	-0.029	0.100	0.950	0.944
2005	FALSE	5,592.593	0.000	0.075	0.975	0.957
2010	FALSE	5,180.552	-0.038	0.055	0.993	0.853
2015	FALSE	5,192.436	-0.071	0.134	0.921	0.736
2018	FALSE	9,477.775	-0.014	0.092	0.974	0.936
2000	TRUE	9,937.514	-0.026	0.097	0.949	0.944
2005	TRUE	5,592.593	-0.011	0.082	0.971	0.932
2010	TRUE	5,180.552	-0.039	0.067	0.987	0.803
2015	TRUE	5,094.664	-0.101	0.140	0.944	0.745
2018	TRUE	9,477.775	-0.029	0.126	0.951	0.946

Supplementary Table 10: Predictive metrics for SFU, aggregated to the second administrative level (Admin 2)

YEAR	OOS	Median SS	Mean err.	RMSE	Corr.	95% Cov.
2000	FALSE	1,423.868	-0.029	0.124	0.933	0.944
2005	FALSE	764.000	0.000	0.117	0.947	0.957
2010	FALSE	333.046	-0.038	0.092	0.972	0.853
2015	FALSE	710.463	-0.071	0.175	0.870	0.736
2018	FALSE	159.633	-0.014	0.131	0.946	0.936
2000	TRUE	1,405.583	-0.026	0.127	0.924	0.944

2005	TRUE	763.358	-0.011	0.129	0.937	0.932
2010	TRUE	331.275	-0.039	0.111	0.957	0.803
2015	TRUE	708.381	-0.101	0.170	0.906	0.745
2018	TRUE	159.170	-0.029	0.154	0.926	0.946

6.0 Author Contributions

Managing the estimation or publications process

Brigette F Blacker, Simon I Hay, and Robert C Reiner Jr.

Writing the first draft of the manuscript

Joseph Jon Frostad, Simon I Hay, and Robert C Reiner Jr.

Primary responsibility for applying analytical methods to produce estimates

Joseph Jon Frostad

Primary responsibility for seeking, cataloguing, extracting, or cleaning data; designing or coding figures and tables

Joseph Jon Frostad, QuynhAnh P Nguyen, Mathew M Baumann, Kate E LeGrand, and Kimberly B Johnson.

Providing data or critical feedback on data sources

QuynhAnh P Nguyen, Kimberly B Johnson, Foad Abd-Allah, Amir Abdoli, Hassan Abolhassani, Niveen ME Abu-Rmeileh, Victor Adekanmbi, Muktar Beshir Ahmed, Fahad Mashhour Alanezi, Turki M Alanzi, Jacqueline Elizabeth Alcalde-Rabanal, Biresaw Wassihun Alemu, Amir Almasi-Hashiani,

Nelson J Alvis-Zakzuk, Saeed Amini, Dickson A Amugsi, Catalina Liliana Andrei, Tudorel Andrei, Fereshteh Ansari, Davood Anvari, Jalal Arabloo, Morteza Arab-Zozani, Marcel Ausloos, Yared Asmare Aynalem, Samad Azari, Atif Amin Baig, Kalpana Balakrishnan, Maciej Banach, Sanjay Basu, Neeraj Bedi, Derrick A Bennett, Catherine P Benziger, Sadia Bibi, Somayeh Bohlouli, Soufiane Boufous, Nicola Luigi Bragazzi, Dejana Braithwaite, Sharath Burugina Nagaraja, Florentino Luciano Caetano dos Santos, Joao Mauricio Castaldelli-Maia, Carlos A Castañeda-Orjuela, Franz Castro, Souranshu Chatterjee, Soosanna Kumary Chattu, Vijay Kumar Chattu, Dinh-Toi Chu, Natalie Maria Cormier, Vera Marisa Costa, Giovanni Damiani, Lalit Dandona, Rakhi Dandona, Aso Mohammad Darwesh, Sagnik Dey, Samath Dhamminda Dharmaratne, Meghnath Dhimal, Govinda Prasad Dhungana, Leila Doshmangir, Andre Rodrigues Duraes, Andem Effiong, Nazir Fattahi, Seyed-Mohammad Fereshtehnejad, Irina Filip, Takeshi Fukumoto, Abhay Motiramji Gaidhane, Mansour Ghafourifard, Ahmad Ghashghaee, Syed Amir Gilani, Ayman Grada, Yuming Guo, Rajat Das Gupta, Nima Hafezi-Nejad, Ahmed I Hasaballah, Soheil Hassanipour, Hadi Hassankhani, Mohamed I Hegazy, Reza Heidari-Soureshjani, Claudiu Herteliu, Sung Hwi Hong, Mehdi Hosseinzadeh, Mowafa Househ, Mohamed Hsairi, Segun Emmanuel Ibitoye, Seyed Sina Naghibi Irvani, Sheikh Mohammed Shariful Islam, Chidozie C D Iwu, Mihajlo Jakovljevic, Tahereh Javaheri, John S Ji, Jost B Jonas, Zubair Kabir, Rohollah Kalhor, Naser Kamyari, Umesh Kapil, Gbenga A Kayode, Chandrasekharan Nair Kesavachandran, Yousef Saleh Khader, Nauman Khalid, Maseer Khan, Md Nuruzzaman Khan, Khaled Khatab, Mona M Khater, Mahalaqua Nazli Khatib, Abdullah T Khoja, Gyu Ri Kim, Adnan Kisa, Sezer Kisa, Luke D Knibbs, Tufa Kolola, Parvaiz A Koul, Kewal Krishan, G Anil Kumar, Dian Kusuma, Van Charles Lansingh, Savita Lasrado, Shanshan Li, Shilpashree Madhava Kunjathur, Deborah Carvalho Malta, Borhan Mansouri, Mohammad Ali Mansournia, Francisco Rogerlândio Martins-Melo, Benjamin K Mayala, Man Mohan Mehndiratta, Walter Mendoza, Ritesh G Menezes, Endalkachew Worku Mengesha, Irmina Maria Michalek, Erkin M Mirrakhimov, Roya Mirzaei, Babak Moazen, Abdollah Mohammadian-Hafshejani, Shafiu Mohammed, Ali H Mokdad, Taklu Marama Mokonnon, Masoud Moradi, Maziar Moradi-Lakeh, Amin Mousavi Khaneghah, Satinath Mukhopadhyay, Christopher J L Murray, Ahamarshan Jayaraman Nagarajan, Mohsen Naghavi, Bruno Ramos Nascimento, Javad Nazari, Ionut Negoi, Henok Biresaw Netsere, Josephine W Ngunjiri, Huong Lan Thi Nguyen, Chukwudi A Nnaji, Jean Jacques Noubiap, Bogdan Oancea, Felix Akpojene Ogbo, Andrew T Olagunju, Bolajoko Olubukunola Olusanya, Jacob Olusegun Olusanya, Ahmed Omar Bali, Obinna E Onwujekwe, Mayowa O Owolabi, Mahesh P A, Jagadish Rao Padubidri, Adrian Pana, Anamika Pandey, Eun-Cheol Park, Fatemeh Pashazadeh Kan, Jenil R Patel, Sangram Kishor Patel, Alexandre Pereira, Hai Quang Pham, Khem Narayan Pokhrel, Maarten J Postma, Hadi Pourjafar, Zahiruddin Quazi Syed, Amir Radfar, Mohammad Hifz Ur Rahman, Amir Masoud Rahmani, Usha Ram, Chhabi Lal Ranabhat, Sowmya J Rao, Prateek Rastogi, Goura Kishor Rath, Priya Rathi, David Laith Rawaf, Salman Rawaf, Lal Rawal, Reza Rawassizadeh, Andre M N Renzaho, Bhageerathy Reshmi, Nima Rezaei, Jennifer Rickard, Leonardo Roever, Luca Ronfani, Enrico Rubagotti, Godfrey M Rwegerera, Basema Saddik, Ehsan Sadeghi, Rajesh Sagar, Marwa Rashad Salem, Abdallah M Samy, Milena M Santric-Milicevic, Sivan Yegnanarayana Iyer Saraswathy, Nizal Sarrafzadegan, Brijesh Sathian, David C Schwebel, Saeed Shahabi, Amira A Shaheen, Masood Ali Shaikh, Mehran Shams-Beyranvand, Morteza Shamsizadeh, Mohammed Shannawaz, Sara Sheikhbahaei, Ranjitha S Shetty, Wondimeneh Shibabaw Shiferaw, Jae Il Shin, K M Shivakumar, Soraya Siabani, Jasvinder A Singh, Mohammad Reza Sobhiyeh, Amin Soheili, Ireneous N Soyiri, Chandrashekhar T Sreeramareddy, Rizwan Suliankatchi Abdulkader, Rafael Tabarés-Seisdedos, Amir Taherkhani, Mohamad-Hani Temsah, Marcos Roberto Tovani-Palone, Bach Xuan Tran, Saif Ullah, Bhaskaran Unnikrishnan, Era Upadhyay, Bay Vo, Giang Thu Vu, Yasir Waheed, Yafeng Wang, Gelin Xu, Sanni Yaya, Vahid Yazdi-Feyzabadi, Naohiro Yonemoto, Mustafa Z Younis, and Chuanhua Yu.

Development of methods or computational machinery

QuynhAnh P Nguyen, Aniruddha Deshpande, Kirsten E Wiens, Muktar Beshir Ahmed, Amir Almasi-Hashiani, Davood Anvari, Somayeh Bohlouli, Sharath Burugina Nagaraja, Aso Mohammad Darwesh, Lucas Earl, Abhay Motiramji Gaidhane, Ahmad Ghashghaee, Yuming Guo, Reza Heidari-Soureshjani, Nathaniel J Henry, Mehdi Hosseinzadeh, Mowafa Househ, Bogdan Ileanu, Tahereh Javaheri, Mohammad Khammarnia, Mahalaqua Nazli Khatib, Adnan Kisa, Sezer Kisa, Shanshan Li, Shilpashree Madhava Kunjathur, Borhan Mansouri, Abdollah Mohammadian-Hafshejani, Ali H Mokdad, Christopher J L Murray, Mohsen Naghavi, Josephine W Ngunjiri, Ahmed Omar Bali, Zahiruddin Quazi Syed, Amir Masoud Rahmani, Chhabi Lal Ranabhat, Reza Rawassizadeh, Enrico Rubagotti, Abdallah M Samy, Bay Vo, Catherine A Welgan, and Robert C Reiner Jr.

Providing critical feedback on methods or results

QuynhAnh P Nguyen, Laurie B Marczak, Aniruddha Deshpande, Kirsten E Wiens, Kate E LeGrand, Foad Abd-Allah, Ibrahim Abdollahpour, Hassan Abolhassani, Lucas Guimarães Abreu, Michael R M Abrigo, Abdelrahman I Abushouk, Victor Adekanmbi, Khashayar Afshari, Anurag Agrawal, Muktar Beshir Ahmed, Ziyad Al-Aly, Fahad Mashhour Alanezi, Turki M Alanzi, Jacqueline Elizabeth Alcalde-Rabanal, Biresaw Wassihun Alemu, Vahid Alipour, Amir Almasi-Hashiani, Khalid A Altirkawi, Nelson Alvis-Guzman, Nelson J Alvis-Zakzuk, Saeed Amini, Fatemeh Amiri, Dickson A Amugsi, Robert Ancuceanu, Catalina Liliana Andrei, Tudorel Andrei, Fereshteh Ansari, Davood Anvari, Jalal Arabloo, Morteza Arab-Zozani, Mohammad Asghari Jafarabadi, Seyyed Shamsadin Athari, Seyyede Masoume Athari, Marcel Ausloos, Getinet Ayano, Yared Asmare Aynalem, Samad Azari, Ghasem Azarian, Ashish D Badiye, Atif Amin Baig, Kalpana Balakrishnan, Maciej Banach, Sanjay Basu, Michelle L Bell, Derrick A Bennett, Catherine P Benziger, Krittika Bhattacharyya, Zulfiqar A Bhutta, Sadia Bibi, Binyam Minuye Birihane, Somayeh Bohlouli, Soufiane Boufous, Nicola Luigi Bragazzi, Dejana Braithwaite, Sharath Burugina Nagaraja, Zahid A Butt, Florentino Luciano Caetano dos Santos, Josip Car, Rosario Cárdenas, Joao Mauricio Castaldelli-Maia, Carlos A Castañeda-Orjuela, Franz Castro, Ester Cerin, Souranshu Chatterjee, Soosanna Kumary Chattu, Vijay Kumar Chattu, Sarika Chaturvedi, Jee-Young Jasmine Choi, Dinh-Toi Chu, Sheng-Chia Chung, Saad M A Dahlawi, Haijiang Dai, Giovanni Damiani, Lalit Dandona, Rakhi Dandona, Aso Mohammad Darwesh, Jai K Das, Aditya Prasad Dash, Diego De Leo, Jan-Walter De Neve, Getu Debalkie Demissie, Sagnik Dey, Meghnath Dhimal, Govinda Prasad Dhungana, Daniel Diaz, Isaac Oluwafemi Dipeolu, Fariba Dorostkar, Leila Doshmangir, Hisham Atan Edinur, Ferry Efendi, Andem Effiong, Sharareh Eskandarieh, Ibtihal Fadhil, Irina Filip, Nataliya A Foigt, Morenike Oluwatoyin Folayan, Masoud Foroutan, Takeshi Fukumoto, Abhay Motiramji Gaidhane, Biniyam Sahiledengle Geberemariyam, Mansour Ghafourifard, Srinivas Goli, Alessandra C Goulart, Bárbara Niegia Garcia Goulart, Ayman Grada, Davide Guido, Yuming Guo, Rajat Das

Gupta, Rajeev Gupta, Reyna Alma Gutiérrez, Nima Hafezi-Nejad, Ahmed I Hasaballah, Soheil Hassanipour, Hadi Hassankhani, Khezar Hayat, Behzad Heibati, Nathaniel J Henry, Claudiu Herteliu, Sung Hwi Hong, Mehdi Hosseinzadeh, Mowafa Househ, Guoqing Hu, Segun Emmanuel Ibitoye, Bogdan Ileanu, Irena M Ilic, Milena D Ilic, Seyed Sina Naghibi Irvani, Sheikh Mohammed Shariful Islam, Chidozie C D Iwu, Jalil Jaafari, Mihajlo Jakovljevic, Tahereh Javaheri, Ravi Prakash Jha, John S Ji, Jost B Jonas, Ali Kabir, Zubair Kabir, Rohollah Kalhor, Naser Kamyari, Tanuj Kanchan, Neeti Kapoor, Gbenga A Kayode, Peter Njenga Keiyoro, Chandrasekharan Nair Kesavachandran, Yousef Saleh Khader, Nauman Khalid, Mohammad Khammarnia, Ejaz Ahmad Khan, Maseer Khan, Md Nuruzzaman Khan, Khaled Khatab, Mona M Khater, Mahalaqua Nazli Khatib, Maryam Khayamzadeh, Ardeshir Khosravi, Jagdish Khubchandani, Gyu Ri Kim, Yun Jin Kim, Ruth W Kimokoti, Adnan Kisa, Sezer Kisa, Luke D Knibbs, Tufa Kolola, Ai Koyanagi, Kewal Krishan, G Anil Kumar, Manasi Kumar, Dian Kusuma, Qing Lan, Savita Lasrado, Paolo Lauriola, Sonia Lewycka, Shanshan Li, Andrea Lobato-Cordero, Daiane Borges Machado, Shilpashree Madhava Kunjathur, Phetole Walter Mahasha, Mina Maheri, Azeem Majeed, Venkatesh Maled, Reza Malekzadeh, Deborah Carvalho Malta, Borhan Mansouri, Mohammad Ali Mansournia, Santi Martini, Francisco Rogerlândio Martins-Melo, Benjamin K Mayala, Sumi Mehta, Walter Mendoza, Ritesh G Menezes, Endalkachew Worku Mengesha, George A Mensah, Tuomo J Meretoja, Tomislav Mestrovic, Irmina Maria Michalek, Ted R Miller, Andreea Mirica, Erkin M Mirrakhimov, Maryam Mirzaei, Babak Moazen, Yousef Mohammad, Abdollah Mohammadian-Hafshejani, Shafiu Mohammed, Ali H Mokdad, Taklu Marama Mokonnon, Maziar Moradi-Lakeh, Rahmatollah Moradzadeh, Lidia Morawska, Simin Mouodi, Amin Mousavi Khaneghah, Christopher J L Murray, Ahamarshan Jayaraman Nagarajan, Mohsen Naghavi, Sanjeev Nair, Vinay Nangia, Bruno Ramos Nascimento, Javad Nazari, Ionut Negoi, Henok Biresaw Netsere, Josephine W Ngunjiri, Huong Lan Thi Nguyen, Chukwudi A Nnaji, Jean Jacques Noubiap, Bogdan Oancea, Felix Akpojene Ogbo, Andrew T Olagunju, Bolajoko Olubukunola Olusanya, Jacob Olusegun Olusanya, Ahmed Omar Bali, Obinna E Onwujekwe, Nikita Otstavnov, Stanislav S Otstavnov, Mahesh P A, Jagadish Rao Padubidri, Adrian Pana, Anamika Pandey, Eun-Cheol Park, Eun-Kee Park, Jenil R Patel, Sangram Kishor Patel, Hai Quang Pham, Meghdad Pirsaheb, Khem Narayan Pokhrel, Maarten J Postma, Hadi Pourjafar, Zahiruddin Quazi Syed, Navid Rabiee, Amir Radfar, Fakher Rahim, Mohammad Hifz Ur Rahman, Muhammad Aziz Rahman, Amir Masoud Rahmani, Usha Ram, Chhabi Lal Ranabhat, Sowmya J Rao, Prateek Rastogi, Priya Rathi, David Laith Rawaf, Salman Rawaf, Lal Rawal, Reza Rawassizadeh, Andre M N Renzaho, Bhageerathy Reshmi, Negar Rezaei, Nima Rezaei, Aziz Rezapour, Jennifer Rickard, Leonardo Roever, Godfrey M Rwegerera, Basema Saddik, Ehsan Sadeghi, Sahar Saeedi Moghaddam, Rajesh Sagar, Marwa Rashad Salem, Abdallah M Samy, Milena M Santric-Milicevic, Sivan Yegnanarayana Iyer Saraswathy, Brijesh Sathian, Thirunavukkarasu Sathish, Lauren E Schaeffer, David C Schwebel, Sadaf G Sepanlou, Saeed Shahabi, Amira A Shaheen, Izza Shahid, Masood Ali Shaikh, Ali S Shalash, Mehran Shams-Beyranvand, MohammadBagher Shamsi, Mohammed Shannawaz, Kiomars Sharafi, Aziz Sheikh, Sara Sheikhbahaei, Ranjitha S Shetty, Wondimeneh Shibabaw Shiferaw, Mika Shigematsu, Jae Il Shin, K M Shivakumar, Soraya Siabani, Tariq Jamal Siddiqi, Balbir Bagicha Singh, Jasvinder A Singh, Yitagesu Sintayehu, Amin Soheili, Muluken Bekele Sorrie, Ireneous N Soyiri, Chandrashekhar T Sreeramareddy, Leo Stockfelt, Mu'awiyyah Babale Sufiyan, Rizwan Suliankatchi Abdulkader, Rafael Tabarés-Seisdedos, Mohamad-Hani Temsah, Hamid Reza Tohidinik, Marcos Roberto Tovani-Palone, Eugenio Traini, Bach Xuan Tran, Saif Ullah, Bhaskaran Unnikrishnan, Era Upadhyay, Muhammad Shariq Usman, Santosh Varughese, Francesco S Violante, Bay Vo, Giang Thu Vu, Yasir Waheed, Yafeng Wang, Catherine A Welgan, Andrea Werdecker, Andrea Sylvia Winkler, Davood Yari, Sanni Yaya, Vahid Yazdi-Feyzabadi, Mekdes Tigistu Yilma, Naohiro Yonemoto, Mustafa Z Younis, Taraneh Yousefinezhadi, Chuanhua Yu, Yong Yu, Hasan

Yusefzadeh, Sojib Bin Zaman, Mohammad Zamani, Maryam Zamanian, Yunquan Zhang, Zhi-Jiang Zhang, Cong Zhu, Michael Brauer, and Robert C Reiner Jr.

Drafting the manuscript or revising it critically for important intellectual content

QuynhAnh P Nguyen, Mathew M Baumann, Laurie B Marczak, Kirsten E Wiens, Mohsen Abbasi-Kangevari, Foad Abd-Allah, Hassan Abolhassani, Lucas Guimarães Abreu, Niveen ME Abu-Rmeileh, Abdelrahman I Abushouk, Victor Adekanmbi, Muktar Beshir Ahmed, Jacqueline Elizabeth Alcalde-Rabanal, Amir Almasi-Hashiani, Nelson Alvis-Guzman, Nelson J Alvis-Zakzuk, Adeladza Kofi Amegah, Dickson A Amugsi, Robert Ancuceanu, Catalina Liliana Andrei, Tudorel Andrei, Ernoiz Antriyandarti, Jalal Arabloo, Morteza Arab-Zozani, Afsaneh Arzani, Mehran Asadi-Aliabadi, Mohammad Asghari Jafarabadi, Marcel Ausloos, Atif Amin Baig, Maciej Banach, Sanjay Basu, Neeraj Bedi, Michelle L Bell, Catherine P Benziger, Krittika Bhattacharyya, Sadia Bibi, Raaj Kishore Biswas, Somayeh Bohlouli, Nicola Luigi Bragazzi, Dejana Braithwaite, Sharath Burugina Nagaraja, Florentino Luciano Caetano dos Santos, Josip Car, Felix Carvalho, Joao Mauricio Castaldelli-Maia, Carlos A Castañeda-Orjuela, Franz Castro, Ester Cerin, Souranshu Chatterjee, Soosanna Kumary Chattu, Vijay Kumar Chattu, Pankaj Chaturvedi, Sarika Chaturvedi, Simiao Chen, Dinh-Toi Chu, Vera Marisa Costa, Haijiang Dai, Giovanni Damiani, Claudio Alberto Dávila-Cervantes, Jan-Walter De Neve, Edgar Denova-Gutiérrez, Samath Dhamminda Dharmaratne, Meghnath Dhimal, Govinda Prasad Dhungana, Daniel Diaz, Leila Doshmangir, Andem Effiong, Maha El Tantawi, Nelsensius Klau Fauk, Seyed-Mohammad Fereshtehnejad, Eduarda Fernandes, Irina Filip, Nataliya A Foigt, Morenike Oluwatoyin Folayan, Masoud Foroutan, Takeshi Fukumoto, Abhay Motiramji Gaidhane, Biniyam Sahiledengle Geberemariyam, Mansour Ghafourifard, Ahmad Ghashghaee, Tiffany K Gill, Alessandra C Goulart, Bárbara Niegia Garcia Goulart, Ayman Grada, Mohammed Ibrahim Mohialdeen Gubari, Rajat Das Gupta, Rajeev Gupta, Reyna Alma Gutiérrez, Nima Hafezi-Nejad, Randah R Hamadeh, Ahmed I Hasaballah, Mohamed I Hegazy, Claudiu Herteliu, Sung Hwi Hong, Mostafa Hosseini, Mowafa Househ, Segun Emmanuel Ibitoye, Olayinka Stephen Ilesanmi, Irena M Ilic, Milena D Ilic, Seyed Sina Naghibi Irvani, Sheikh Mohammed Shariful Islam, Chidozie C D Iwu, Jalil Jaafari, Mihajlo Jakovljevic, Ravi Prakash Jha, John S Ji, Jost B Jonas, Ali Kabir, Umesh Kapil, Gbenga A Kayode, Chandrasekharan Nair Kesavachandran, Ejaz Ahmad Khan, Maseer Khan, Md Nuruzzaman Khan, Khaled Khatab, Mona M Khater, Mahalaqua Nazli Khatib, Abdullah T Khoja, Jagdish Khubchandani, Gyu Ri Kim, Yun Jin Kim, Adnan Kisa, Sezer Kisa, Luke D Knibbs, Parvaiz A Koul, Ai Koyanagi, Kewal Krishan, Manasi Kumar, Dian Kusuma, Carlo La Vecchia, Ben Lacey, Faris Hasan Lami, Van Charles Lansingh, Savita Lasrado, Paul H Lee, Daiane Borges Machado, Shilpashree Madhava Kunjathur, Venkatesh Maled, Afshin Maleki, Reza Malekzadeh, Deborah Carvalho Malta, Borhan Mansouri, Francisco Rogerlândio Martins-Melo, Pallab K Maulik, Walter Mendoza, Ritesh G Menezes, Tuomo J Meretoja, Tomislav Mestrovic, Irmina Maria Michalek, Ted R Miller, Molly K Miller-Petrie, Babak Moazen, Yousef Mohammad, Abdollah Mohammadian-Hafshejani, Shafiu Mohammed, Ali H Mokdad, Taklu Marama Mokonnon, Lorenzo Monasta, Maziar Moradi-Lakeh, Paula Moraga, Abbas Mosapour, Simin Mouodi, Amin Mousavi Khaneghah, Satinath Mukhopadhyay, Sandra B Munro, Christopher J L Murray, Ahamarshan Jayaraman Nagarajan, Mohsen Naghavi, Sanjeev Nair, Vinay Nangia, Bruno Ramos Nascimento, Vinod C Nayak, Ionut Negoi, Josephine W Ngunjiri, Huong Lan Thi Nguyen, Jean Jacques Noubiap, Felix Akpojene Ogbo, In-Hwan Oh, Andrew T

Olagunju, Bolajoko Olubukunola Olusanya, Jacob Olusegun Olusanya, Obinna E Onwujekwe, Nikita Otstavnov, Stanislav S Otstavnov, Mahesh P A, Jagadish Rao Padubidri, Adrian Pana, Eun-Cheol Park, Jenil R Patel, Alexandre Pereira, Hai Quang Pham, Thomas Pilgrim, Khem Narayan Pokhrel, Maarten J Postma, Zahiruddin Quazi Syed, Navid Rabiee, Amir Radfar, Fakher Rahim, Mohammad Hifz Ur Rahman, Muhammad Aziz Rahman, Chhabi Lal Ranabhat, Sowmya J Rao, Davide Rasella, David Laith Rawaf, Salman Rawaf, Lal Rawal, Andre M N Renzaho, Bhageerathy Reshmi, Nima Rezaei, Jennifer Rickard, Leonardo Roever, Luca Ronfani, Morteza Rostamian, Godfrey M Rwegerera, Basema Saddik, Rajesh Sagar, Amirhossein Sahebkar, Marwa Rashad Salem, Abdallah M Samy, Milena M Santric-Milicevic, Nizal Sarrafzadegan, Thirunavukkarasu Sathish, Lauren E Schaeffer, David C Schwebel, Sadaf G Sepanlou, Izza Shahid, Mehran Shams-Beyranvand, Sara Sheikhbahaei, Mika Shigematsu, K M Shivakumar, Tariq Jamal Siddiqi, Balbir Bagicha Singh, Jasvinder A Singh, Ireneous N Soyiri, Chandrashekhar T Sreeramareddy, Mu'awiyyah Babale Sufiyan, Carolyn B Swope, Takahiro Tabuchi, Mohamad-Hani Temsah, Kavumpurathu Raman Thankappan, Hamid Reza Tohidinik, Marcos Roberto Tovani-Palone, Bach Xuan Tran, Aristidis Tsatsakis, Saif Ullah, Bhaskaran Unnikrishnan, Era Upadhyay, Muhammad Shariq Usman, Sahel Valadan Tahbaz, Francesco S Violante, Giang Thu Vu, Yasir Waheed, Yuan-Pang Wang, Andrea Werdecker, Andrea Sylvia Winkler, Gelin Xu, Seyed Hossein Yahyazadeh Jabbari, Davood Yari, Vahid Yazdi-Feyzabadi, Mekdes Tigistu Yilma, Naohiro Yonemoto, Sojib Bin Zaman, Maryam Zamanian, Michael Brauer, Simon I Hay, and Robert C Reiner Jr.

Extracting, cleaning, or cataloging data; designing or coding figures and tables

QuynhAnh P Nguyen, Mathew M Baumann, Kate E LeGrand, Kimberly B Johnson, Muktar Beshir Ahmed, Saeed Amini, Fatemeh Amiri, Somayeh Bohlouli, Lucas Earl, Takeshi Fukumoto, Abhay Motiramji Gaidhane, Ahmed I Hasaballah, Jalil Jaafari, Md Nuruzzaman Khan, Mahalaqua Nazli Khatib, Borhan Mansouri, Seyyede Momeneh Mohammadi, Ali H Mokdad, Christopher J L Murray, Mohsen Naghavi, Javad Nazari, Zahiruddin Quazi Syed, Enrico Rubagotti, Abdallah M Samy, Emma Elizabeth Spurlock, and Catherine A Welgan.

Management of the overall research enterprise

QuynhAnh P Nguyen, Brigette F Blacker, Laurie B Marczak, Lalit Dandona, Ali H Mokdad, Christopher J L Murray, Mohsen Naghavi, and Simon I Hay.

Local Burden of Disease 2017 Household Air Pollution II Collaborators

Joseph Jon Frostad, QuynhAnh P Nguyen, Mathew M Baumann, Brigette F Blacker, Laurie B Marczak, Aniruddha Deshpande, Kirsten E Wiens, Kate E LeGrand, Kimberly B Johnson, Mohsen Abbasi-Kangevari, Amir Abdoli, Hassan Abolhassani, Lucas Guimarães Abreu, Michael R M Abrigo, Niveen ME Abu-Rmeileh, Victor Adekanmbi, Anurag Agrawal, Muktar Beshir Ahmed, Ziyad Al-Aly, Fahad Mashhour Alanezi, Jacqueline Elizabeth Alcalde-Rabanal, Vahid Alipour, Khalid A Altirkawi, Nelson Alvis-Guzman, Nelson J Alvis-Zakzuk, Adeladza Kofi Amegah, Saeed Amini, Fatemeh Amiri, Dickson A Amugsi, Robert Ancuceanu, Catalina Liliana Andrei, Tudorel Andrei, Ernoiz Antriyandarti, Davood Anvari, Jalal Arabloo, Morteza Arab-Zozani, Seyyed Shamsadin Athari, Marcel Ausloos, Getinet Ayano, Yared Asmare Aynalem, Samad Azari, Ashish D Badiye, Atif Amin Baig, Kalpana Balakrishnan, Maciej Banach, Sanjay Basu, Neeraj Bedi, Michelle L Bell, Derrick A Bennett, Krittika Bhattacharyya, Zulfiqar A Bhutta, Sadia Bibi, Somayeh Bohlouli, Soufiane Boufous, Nicola Luigi Bragazzi, Dejana Braithwaite, Sharath Burugina Nagaraja, Zahid A Butt, Florentino Luciano Caetano dos Santos, Josip Car, Rosario Cárdenas, Felix Carvalho, Joao Mauricio Castaldelli-Maia, Carlos A Castañeda-Orjuela, Ester Cerin, Soosanna Kumary Chattu, Vijay Kumar Chattu, Pankaj Chaturvedi, Sarika Chaturvedi, Simiao Chen, Dinh-Toi Chu, Sheng-Chia Chung, Saad M A Dahlawi, Giovanni Damiani, Lalit Dandona, Rakhi Dandona, Aso Mohammad Darwesh, Jai K Das, Aditya Prasad Dash, Claudio Alberto Dávila-Cervantes, Diego De Leo, Jan-Walter De Neve, Getu Debalkie Demissie, Edgar Denova-Gutiérrez, Sagnik Dey, Samath Dhamminda Dharmaratne, Meghnath Dhimal, Govinda Prasad Dhungana, Daniel Diaz, Isaac Oluwafemi Dipeolu, Fariba Dorostkar, Leila Doshmangir, Andre Rodrigues Duraes, Hisham Atan Edinur, Ferry Efendi, Maha El Tantawi, Sharareh Eskandarieh, Ibtihal Fadhil, Nazir Fattahi, Nelsensius Klau Fauk, Seyed-Mohammad Fereshtehnejad, Morenike Oluwatoyin Folayan, Masoud Foroutan, Takeshi Fukumoto, Abhay Motiramji Gaidhane, Mansour Ghafourifard, Ahmad Ghashghaee, Syed Amir Gilani, Tiffany K Gill, Alessandra C Goulart, Bárbara Niegia Garcia Goulart, Ayman Grada, Mohammed Ibrahim Mohialdeen Gubari, Davide Guido, Yuming Guo, Rajat Das Gupta, Rajeev Gupta, Reyna Alma Gutiérrez, Nima Hafezi-Nejad, Randah R Hamadeh, Ahmed I Hasaballah, Soheil Hassanipour, Khezar Hayat, Behzad Heibati, Reza Heidari-Soureshjani, Nathaniel J Henry, Claudiu Herteliu, Mehdi Hosseinzadeh, Mohamed Hsairi, Guoqing Hu, Segun Emmanuel Ibitoye, Olayinka Stephen Ilesanmi, Irena M Ilic, Milena D Ilic, Seyed Sina Naghibi Irvani, Sheikh Mohammed Shariful Islam, Chidozie C D Iwu, Jalil Jaafari, Mihajlo Jakovljevic, Tahereh Javaheri, Ravi Prakash Jha, John S Ji, Jost B Jonas, Ali Kabir, Zubair Kabir, Rohollah Kalhor, Naser Kamyari, Tanuj Kanchan, Umesh Kapil, Neeti Kapoor, Gbenga A Kayode, Peter Njenga Keiyoro, Yousef Saleh Khader, Nauman Khalid, Ejaz Ahmad Khan, Maseer Khan, Md Nuruzzaman Khan, Khaled Khatab, Mona M Khater, Mahalaqua Nazli Khatib, Maryam Khayamzadeh, Jagdish Khubchandani, Gyu Ri Kim, Yun Jin Kim, Ruth W Kimokoti, Adnan Kisa, Sezer Kisa, Luke D Knibbs, Parvaiz A Koul, Ai Koyanagi, Kewal Krishan, G Anil Kumar, Manasi Kumar, Dian Kusuma, Carlo La Vecchia, Ben Lacey, Faris Hasan Lami, Qing Lan, Savita Lasrado, Paolo Lauriola, Paul H Lee, Sonia Lewycka, Shanshan Li, Daiane Borges Machado, Phetole Walter Mahasha, Mina Maheri, Azeem Majeed, Afshin Maleki, Reza Malekzadeh, Deborah Carvalho Malta, Borhan Mansouri, Mohammad Ali Mansournia, Natalie Maria Martinez, Santi Martini, Francisco Rogerlândio Martins-Melo, Benjamin K Mayala, Man Mohan Mehndiratta, Walter Mendoza, Ritesh G Menezes, Endalkachew Worku Mengesha, Tuomo J Meretoja, Tomislav Mestrovic, Irmina Maria Michalek, Erkin M Mirrakhimov, Maryam Mirzaei, Roya Mirzaei, Babak Moazen, Yousef Mohammad, Abdollah Mohammadian-Hafshejani, Shafiu Mohammed, Ali H Mokdad, Lorenzo Monasta, Maziar Moradi-Lakeh, Paula Moraga, Lidia Morawska, Abbas Mosapour, Simin Mouodi, Amin Mousavi Khaneghah, Satinath Mukhopadhyay, Sandra B Munro, Christopher J L Murray, Ahamarshan Jayaraman Nagarajan, Mohsen Naghavi, Sanjeev Nair, Vinay Nangia, Bruno Ramos Nascimento, Javad Nazari, Ionut Negoi, Henok Biresaw Netsere, Josephine W Ngunjiri, Huong Lan Thi Nguyen, Jean Jacques Noubiap, Bogdan Oancea, Felix Akpojene Ogbo, In-Hwan Oh, Andrew T Olagunju, Bolajoko Olubukunola Olusanya, Jacob Olusegun Olusanya, Ahmed Omar Bali, Obinna E Onwujekwe, Nikita Otstavnov, Stanislav S Otstavnov, Mayowa O Owolabi, Mahesh P A, Anamika Pandey, Eun-Cheol Park, Eun-Kee Park, Sangram Kishor Patel, Hai Quang Pham, Thomas Pilgrim, Meghdad Pirsaheb, Khem Narayan Pokhrel, Maarten J Postma, Zahiruddin Quazi Syed, Navid Rabiee, Amir Radfar, Fakher Rahim, Mohammad Hifz Ur Rahman, Muhammad Aziz Rahman, Amir Masoud Rahmani, Chhabi Lal Ranabhat, Sowmya J Rao, Davide Rasella, Prateek Rastogi, Goura Kishor Rath, David Laith Rawaf, Salman Rawaf, Lal Rawal, Reza Rawassizadeh, Andre M N Renzaho, Bhageerathy Reshmi, Negar Rezaei, Nima Rezaei, Aziz Rezapour, Jennifer Rickard, Leonardo Roever, Luca Ronfani, Morteza Rostamian, Enrico Rubagotti, Godfrey M Rwegerera, Basema Saddik, Ehsan Sadeghi, Sahar Saeedi Moghaddam, Rajesh Sagar, Amirhossein Sahebkar, Biniyam Sahiledengle, Marwa Rashad Salem, Abdallah M Samy, Milena M Santric-Milicevic, Sivan Yegnanarayana Iyer Saraswathy, Brijesh Sathian, Thirunavukkarasu Sathish, David C Schwebel, Sadaf G Sepanlou, Saeed Shahabi, Amira A Shaheen, Izza Shahid, Masood Ali Shaikh, Ali S Shalash, Mehran Shams-Beyranvand, Mohammed Shannawaz, Kiomars Sharafi, Aziz Sheikh, Sara Sheikhbahaei, Ranjitha S Shetty, Wondimeneh Shibabaw Shiferaw, Mika Shigematsu, Jae Il Shin, K M Shivakumar, Soraya Siabani, Tariq Jamal Siddiqi, Balbir Bagicha Singh, Jasvinder A Singh, Yitagesu Sintayehu, Muluken Bekele Sorrie, Ireneous N Soyiri, Emma Elizabeth Spurlock, Chandrashekhar T Sreeramareddy, Leo Stockfelt, Mu'awiyyah Babale Sufiyan, Rizwan Suliankatchi Abdulkader, Rafael Tabarés-Seisdedos, Takahiro Tabuchi, Amir Taherkhani, Mohamad-Hani Temsah, Kavumpurathu Raman Thankappan, Marcos Roberto Tovani-Palone, Eugenio Traini, Saif Ullah, Bhaskaran Unnikrishnan, Era Upadhyay, Sahel Valadan Tahbaz, Santosh Varughese, Francesco S Violante, Bay Vo, Giang Thu Vu, Yasir Waheed, Yuan-Pang Wang, Catherine A Welgan, Andrea Werdecker, Seyed Hossein Yahyazadeh Jabbari, Sanni Yaya, Vahid Yazdi-Feyzabadi, Mekdes Tigistu Yilma, Naohiro Yonemoto, Mustafa Z Younis, Taraneh Yousefinezhadi, Chuanhua Yu, Yong Yu, Sojib Bin Zaman, Yunquan Zhang, Zhi-Jiang Zhang, Michael Brauer, Simon I Hay, Robert C Reiner Jr.

Affiliations

Institute for Health Metrics and Evaluation (J J Frostad MPH, Q P Nguyen BS, M M Baumann BS, B F Blacker MPH, L B Marczak PhD, K E LeGrand MPH, K B Johnson MS, Prof L Dandona MD, Prof R Dandona PhD, Prof S D Dharmaratne MD, N M Martinez MPSA, B K Mayala PhD, T Mestrovic PhD, A H Mokdad PhD, Prof C J L Murray DPhil, Prof M Naghavi PhD, E E Spurlock MPH, C A Welgan BS, Prof M Brauer DSc, Prof S I Hay FMedSci, R C Reiner Jr PhD), Department of Health Metrics Sciences, School of Medicine (Prof R Dandona, Prof S D Dharmaratne, A H Mokdad, Prof C J L Murray, Prof M Naghavi, Prof S I Hay, R C Reiner Jr), University of Washington, Seattle, WA, USA; Department of Epidemiology, Emory University, Atlanta, GA, USA (A Deshpande MPH); Department of Epidemiology (K E Wiens PhD), Department of Radiology and Radiological Science (N Hafezi-Nejad MD, S Sheikhbahaei MD), Johns Hopkins University, Baltimore, MD, USA; Social Determinants of Health Research Center (M Abbasi-Kangevari MD), Injury Prevention and Safety Promotion Research Center (T Yousefinezhadi PhD), Shahid Beheshti University of Medical Sciences, Tehran, Iran (M Khayamzadeh MD); Zoonoses Research Center, Jahrom University of Medical Sciences, Jahrom, Iran (A Abdoli PhD); Research Center for Immunodeficiencies (H Abolhassani PhD, Prof N Rezaei PhD), Multiple Sclerosis Research Center (S Eskandarieh PhD), School of Medicine (N Hafezi-Nejad MD), Department of Environmental Health Engineering (Prof A Maleki PhD), Digestive Diseases Research Institute (Prof R Malekzadeh MD, S G Sepanlou MD), Department of Epidemiology and Biostatistics (M Mansournia PhD), Water Quality Research Center (R Mirzaei PhD), Metabolomics and Genomics Research Center (F Rahim PhD), Non-communicable Diseases Research Center (N Rezaei PhD, S Saeedi Moghaddam MSc), Endocrinology and Metabolism Research Institute (N Rezaei PhD), Tehran University of Medical Sciences, Tehran, Iran; Department of Biosciences and Nutrition, Karolinska University Hospital, Huddinge, Sweden (H Abolhassani PhD); Department of Pediatric Dentistry (Prof L G Abreu PhD), Department of Maternal and Child Nursing and Public Health (Prof D C Malta PhD), Department of Clinical Medicine (Prof B R Nascimento PhD), Clinical Hospital (Prof B R Nascimento PhD), Federal University of Minas Gerais, Belo Horizonte, Brazil; Department of Research, Philippine Institute for Development Studies, Quezon City, Philippines (M R M Abrigo PhD); Institute of Community and Public Health, Birzeit University, Ramallah, Palestine (Prof N M Abu-Rmeileh PhD); Department of Obstetrics & Gynecology, University of Texas, Galveston, TX, USA (V Adekanmbi PhD); Trivedi School of Biosciences, Ashoka University, Sonipat, India (Prof A Agrawal PhD); Section of General Internal Medicine, Baylor College of Medicine, Houston, TX, USA (Prof A Agrawal); Department of Epidemiology, Jimma University, Jimma, Ethiopia (M B Ahmed MPH); Australian Center for Precision Health, University of South Australia, Adelaide, SA, Australia (M B Ahmed); John T Milliken Department of Internal Medicine, Washington University in St Louis, St Louis, MO, USA (Z Al-Aly MD); Clinical Epidemiology Center, US Department of Veterans Affairs, St Louis, MO, USA (Z Al-Aly); Environmental Health Department (S M A Dahlawi PhD), Forensic Medicine Division (Prof R G Menezes MD), Imam Abdulrahman Bin Faisal University, Dammam, Saudi Arabia (F M Alanezi PhD); Center for Health Systems Research (J E Alcalde-Rabanal PhD), Center for Nutrition and Health Research (E Denova-Gutiérrez DSc), National Institute of Public Health, Cuernavaca, Mexico; Health Management and Economics Research Center (V Alipour PhD, J Arabloo PhD, A Rezapour PhD), Department of Health Economics (V Alipour), Hospital Management Research Center (S Azari PhD), Department of Medical Laboratory Sciences (F Dorostkar PhD), Minimally Invasive Surgery Research Center (A Kabir MD), Comprehensive Research Laboratory (R Mirzaei PhD), Preventive Medicine and Public Health Research Center (M Moradi-Lakeh MD), Iran University of Medical Sciences, Tehran, Iran; Pediatric Intensive

Care Unit (K A Altirkawi MD, M Temsah MD), Internal Medicine Department (Y Mohammad MD), King Saud University, Riyadh, Saudi Arabia; Research Group in Hospital Management and Health Policies (Prof N Alvis-Guzman PhD), Department of Economic Sciences (N J Alvis-Zakzuk MSc), Universidad de la Costa (University of the Coast), Barranquilla, Colombia; Research Group in Health Economics, University of Cartagena, Cartagena, Colombia (Prof N Alvis-Guzman PhD); National Health Observatory (N J Alvis-Zakzuk MSc), Colombian National Health Observatory (C A Castañeda-Orjuela MD), National Institute of Health, Bogota, Colombia; Department of Biomedical Science, University of Cape Coast, Cape Coast, Ghana (A K Amegah PhD); Department of Health Services Management, Khomein University of Medical Sciences, Khomein, Iran (S Amini PhD); Department of Radiology and Nuclear Medicine (F Amiri MSc), Research Center for Environmental Determinants of Health (N Fattahi PhD, Prof M Pirsaheb PhD, Prof E Sadeghi PhD, K Sharafi PhD), Substance Abuse Prevention Research Center (B Mansouri PhD), Department of Rehabilitation and Sports Medicine (M Mirzaei MSc), Department of Health Education and Health Promotion (S Siabani PhD), Kermanshah University of Medical Sciences, Kermanshah, Iran; Maternal and Child Wellbeing, African Population and Health Research Center, Nairobi, Kenya (D A Amugsi PhD); Department of Pharmacy (Prof R Ancuceanu PhD), Department of Cardiology (C Andrei PhD), Department of General Surgery (I Negoi PhD), Carol Davila University of Medicine and Pharmacy, Bucharest, Romania; Department of Statistics and Econometrics (Prof T Andrei PhD, Prof M Ausloos PhD, Prof C Herteliu PhD), Bucharest University of Economic Studies, Bucharest, Romania; Agribusiness Study Program, Sebelas Maret University, Surakarta, Indonesia (E Antriyandarti DrAgrSc); Department of Parasitology, Mazandaran University of Medical Sciences, Sari, Iran (D Anvari PhD); Department of Parasitology, Iranshahr University of Medical Sciences, Iranshahr, Iran (D Anvari); Social Determinants of Health Research Center, Birjand University of Medical Sciences, Birjand, Iran (M Arab-Zozani PhD); Department of Immunology, Zanjan University of Medical Sciences, Zanjan, Iran (S Athari PhD); School of Business (Prof M Ausloos PhD), Department of Health Sciences (P H Lee PhD), University of Leicester, Leicester, UK; School of Indigenous Studies, University of Western Australia, Perth, WA, Australia (G Ayano MSc); School of Public Health, Curtin University, Perth, WA, Australia (G Ayano); Department of Nursing, Debre Berhan University, Debre Berhan, Ethiopia (Y A Aynalem MSc, W S Shiferaw MSc); Department of Forensic Science, Government Institute of Forensic Science, Nagpur, India (A D Badiye MSc, N Kapoor MSc); Unit of Biochemistry (A A Baig PhD), Universiti Sultan Zainal Abidin (Sultan Zainal Abidin University), Kuala Terengganu, Malaysia; Department of Environmental Health Engineering, Sri Ramachandra Medical College and Research Institute, Chennai, India (Prof K Balakrishnan PhD); Department of Hypertension, Medical University of Lodz, Lodz, Poland (Prof M Banach PhD); Polish Mothers' Memorial Hospital Research Institute, Lodz, Poland (Prof M Banach); Center for Primary Care (S Basu PhD), Division of General Internal Medicine (Prof A Sheikh MD), Harvard University, Boston, MA, USA; School of Public Health (S Basu PhD), Department of Primary Care and Public Health (J Car PhD, Prof A Majeed MD, Prof S Rawaf MD), Imperial College Business School (D Kusuma DSc), WHO Collaborating Centre for Public Health Education and Training (D L Rawaf MD), Imperial College London, London, UK; School of Public Health, Dr D Y Patil University, Mumbai, India (Prof N Bedi MD); Department of Epidemiology (M Khan MD), Jazan University, Jazan, Saudi Arabia (Prof N Bedi MD); School of the Environment (Prof M L Bell PhD), Yale School of Public Health—Social and Behavioral Sciences (E E Spurlock MPH), Yale University, New Haven, CT, USA; Nuffield Department of Population Health (D A Bennett PhD, B Lacey PhD), Nuffield Department of Clinical Medicine (N J Henry BS), Centre for Tropical Medicine and Global Health (S Lewycka PhD), The George Institute for Global Health (Prof S Yaya PhD), University of Oxford, Oxford, UK; Department of Statistical and Computational Genomics, National Institute of Biomedical Genomics, Kalyani, India (K Bhattacharyya MSc); Department of Statistics, University of Calcutta, Kolkata, India (K Bhattacharyya MSc); Centre for Global Child Health, University of Toronto, Toronto, ON, Canada (Prof Z A Bhutta PhD); Centre of Excellence in Women & Child Health (Prof Z A Bhutta), Division of Women and Child Health (J K Das MD), Aga Khan University, Karachi, Pakistan; Institute of Soil and Environmental Sciences, University of Agriculture, Faisalabad, Faisalabad, Pakistan (S Bibi PhD, S Ullah PhD); Department of Veterinary Medicine, Islamic Azad University, Kermanshah, Iran (S Bohlouli PhD); Transport and Road Safety Research Centre, University of New South Wales, Sydney, NSW, Australia (S Boufous PhD); University of Genoa, Genoa, Italy (N L Bragazzi PhD); Department of Epidemiology, University of Florida, Gainesville, FL, USA (D Braithwaite PhD); Cancer Population Sciences Program, University of Florida Health Cancer Center, Gainesville, FL, USA (D Braithwaite); Department of Community Medicine, Employee State Insurance Post Graduate Institute of Medical Sciences and Research, Bangalore, India (Prof S Burugina Nagaraja MD); School of Public Health and Health Systems, University of Waterloo, Waterloo, ON, Canada (Z A Butt PhD); Al Shifa School of Public Health, Al Shifa Trust Eye Hospital, Rawalpindi, Pakistan (Z A Butt); Institute of Microengineering, Federal Polytechnic School of Lausanne, Lausanne, Switzerland (F Caetano dos Santos PhD); Centre for Population Health Sciences, Nanyang Technological University, Singapore, Singapore (J Car PhD); Department of Health Care, Metropolitan Autonomous University, Mexico City, Mexico (Prof R Cárdenas DSc); Research Unit on Applied Molecular Biosciences (UCIBIO), University of Porto, Porto, Portugal (Prof F Carvalho PhD); Department of Psychiatry (Prof J Castaldelli-Maia PhD, Y Wang PhD), Center for Clinical and Epidemiological Research (A C Goulart PhD), Department of Internal Medicine (A C Goulart PhD), Department of Pathology and Legal Medicine (M R Tovani-Palone PhD), University of São Paulo, São Paulo, Brazil; Epidemiology and Public Health Evaluation Group, National University of Colombia, Bogota, Colombia (C A Castañeda-Orjuela MD); Mary MacKillop Institute for Health Research, Australian Catholic University, Melbourne, VIC, Australia (Prof E Cerin PhD); School of Public Health, University of Hong Kong, Hong Kong, China (Prof E Cerin); Department of Public Health, Texila American University, Georgetown, Guyana (S Chattu PhD); Department of Community Medicine, Datta Meghe Institute of Medical Sciences, Sawangi, India (V Chattu MD); Saveetha Medical College, Saveetha University, Chennai, India (V Chattu); Center for Cancer Epidemiology, Tata Memorial Hospital, Navi Mumbai, India (Prof P Chaturvedi MD); Department of Head Neck Surgery, Tata Memorial Hospital, Mumbai, India (Prof P Chaturvedi); Department of Research, Dr D Y Patil University, Pune, India (S Chaturvedi PhD); Heidelberg Institute of Global Health, Heidelberg University, Heidelberg, Germany (S Chen DSc, J De Neve MD, B Moazen MSc); Center for Biomedicine and Community Health, VNU-International School, Hanoi, Viet Nam (D Chu PhD); Department of Health Informatics (S Chung PhD), Division of Psychology and Language Sciences (M Kumar PhD), University College London, London, UK; Health Data Research UK, London, UK (S Chung PhD); IRCCS Istituto Ortopedico Galeazzi (G Damiani MD), Department of Clinical Sciences and Community Health (Prof C La Vecchia MD), University of Milan, Milan, Italy; Department of Dermatology, Case Western Reserve University, Cleveland, OH, USA (G Damiani); Department of Research (A Pandey PhD), Public Health Foundation of India, Gurugram, India (Prof L Dandona MD, Prof R Dandona PhD, G Kumar PhD); Indian Council of Medical Research, New Delhi, India (Prof L Dandona MD); Department of Information Technology (A M Darwesh PhD), Department of Computer Science (M Hosseinzadeh PhD), Diplomacy and Public Relations Department (A Omar Bali PhD), University of Human Development, Sulaymaniyah, Iraq; Asian Institute of Public Health University, Bhubaneswar, India (Prof A P Dash DSc); Department of Population and Development, Latin American Faculty of Social Sciences Mexico, Mexico City, Mexico (C A Dávila-Cervantes PhD); Australian Institute for Suicide Research and Prevention, Griffith University, Mount Gravatt, QLD, Australia (Prof D De Leo DSc); Institute of Public health (G D Demissie MPH), School of Nursing (H B Netsere MS), University of Gondar, Gondar, Ethiopia; Centre for Atmospheric Sciences, Indian Institute of Technology Delhi, New Delhi, India (S Dey PhD); Department of Community Medicine, University of Peradeniya, Peradeniya, Sri Lanka (Prof S D Dharmaratne MD); Health Research Section, Nepal Health Research Council, Kathmandu, Nepal (M Dhimal PhD); Department of Microbiology, Far Western University, Mahendranagar, Nepal (G P Dhungana MSc); Center of Complexity Sciences, National Autonomous University of Mexico, Mexico City, Mexico (Prof D Diaz PhD); Faculty of Veterinary Medicine and Zootechnics, Autonomous University of Sinaloa, Culiacán Rosales, Mexico (Prof D Diaz PhD); Health Promotion and Education (I O Dipeolu PhD), Department of Health Promotion and Education (S E Ibitoye MPH), Department of Community Medicine (O S Ilesanmi PhD), Department of Medicine (Prof M O Owolabi DrM), University of Ibadan, Ibadan, Nigeria; Department of Health Policy and Management (L Doshmangir PhD), Department of Medical Surgical Nursing (M Ghafourifard PhD), Tabriz University of Medical Sciences, Tabriz, Iran; School of Medicine (Prof A R Duraes PhD), Institute of Collective Health (Prof D Rasella PhD), Federal University of Bahia, Salvador, Brazil; Department of Internal Medicine (Prof A R Duraes PhD), Escola Bahiana de Medicina e Saúde Pública (Bahiana School of Medicine and Public Health), Salvador, Brazil; School of Health Sciences (H A Edinur PhD), Universiti Sains Malaysia (University of Science Malaysia), Kubang Kerian, Malaysia; Community Health Nursing (F Efendi PhD), Faculty of Public Health (S Martini PhD), Universitas Airlangga (Airlangga University), Surabaya, Indonesia; School of Nursing and Midwifery, La Trobe University, Melbourne, VIC, Australia (F Efendi PhD, M Rahman PhD); Pediatric Dentistry and Dental Public Health Department, Alexandria University, Alexandria, Egypt (Prof M El Tantawi PhD); Division of Non-communicable Diseases, Ministry of Public Health and Population, Dubai, United Arab Emirates (I Fadhil PhD); Torrens University Australia, Adelaide, SA, Australia (N K Fauk MSc); Institute of Resource Governance and Social Change, Kupang, Indonesia (N K Fauk MSc); Department of Neurobiology, Karolinska Institute, Stockholm, Sweden (S Fereshtehnejad PhD); Division of Neurology (S Fereshtehnejad), School of International Development and Global Studies (Prof S Yaya PhD), University of Ottawa, Ottawa, ON, Canada; Department of Child Dental Health, Obafemi Awolowo University, Ile-Ife, Nigeria (Prof M O Folayan FWACS); Department of Medical Parasitology (M Foroutan PhD), Faculty of Medicine (M Foroutan PhD), Department of Biostatistics (N Kamyari PhD), Abadan University of Medical Sciences, Abadan, Iran; Department of Dermatology, Kobe University, Kobe, Japan (T Fukumoto PhD); Department of Community Medicine (Prof A M Gaidhane MD, Prof Z Quazi Syed PhD), Global Evidence Synthesis Initiative (Prof M Khatib PhD), Datta Meghe Institute of Medical Sciences, Wardha, India; School of Public Health (A Ghashghaee BSc), Institute for Prevention of Non-communicable Diseases (R Kalhor PhD), Health Services Management Department (R Kalhor PhD), Qazvin University of Medical Sciences, Qazvin, Iran; Faculty of Allied Health Sciences, University of Lahore, Lahore, Pakistan (Prof S Gilani PhD); Afro-Asian Institute, Lahore, Pakistan (Prof S Gilani);

Adelaide Medical School (T K Gill PhD), Centre for Heart Rhythm Disorders (J Noubiap MD), University of Adelaide, Adelaide, SA, Australia; Postgraduate Program in Epidemiology, Federal University of Rio Grande do Sul, Porto Alegre, Brazil (Prof B N G Goulart DSc); Department of Dermatology (A Grada MD), Health Informatic Lab (T Javaheri PhD), Department of Computer Science (R Rawassizadeh PhD), Boston University, Boston, MA, USA; Department of Family and Community Medicine, University Of Sulaimani, Sulaimani, Iraq (M I M Gubari PhD); UO Neurologia, Salute Pubblica e Disabilità, Fondazione IRCCS Istituto Neurologico Carlo Besta (Neurology, Public Health and Disability Unit, Carlo Besta Neurological Institute), Milan, Italy (D Guido PhD); Department of Epidemiology and Preventive Medicine (Prof Y Guo PhD), School of Public Health and Preventive Medicine (S Li PhD), School of Clinical Sciences at Monash Health (S Zaman MPH), Monash University, Melbourne, VIC, Australia; Department of Epidemiology, Binzhou Medical University, Yantai City, China (Prof Y Guo PhD); Department of Epidemiology and Biostatistics, University of South Carolina, Columbia, SC, USA (R Gupta MPH); Centre for Non-communicable Diseases and Nutrition, BRAC University, Dhaka, Bangladesh (R Gupta MPH); Department of Preventive Cardiology, Eternal Heart Care Centre & Research Institute, Jaipur, India (Prof R Gupta MD); Department of Medicine, Mahatma Gandhi University Medical Sciences, Jaipur, India (Prof R Gupta); Department of Epidemiology and Psychosocial Research, Ramón de la Fuente Muñiz National Institute of Psychiatry, Mexico City, Mexico (R A Gutiérrez PhD); Department of Family and Community Medicine, Arabian Gulf University, Manama, Bahrain (Prof R R Hamadeh PhD); Department of Zoology and Entomology, Al Azhar University, Cairo, Egypt (A I Hasaballah PhD); Gastrointestinal and Liver Diseases Research Center (S Hassanipour PhD), Caspian Digestive Disease Research Center (S Hassanipour), Department of Environmental Health Engineering (J Jaafari PhD), Guilan University of Medical Sciences, Rasht, Iran; Institute of Pharmaceutical Sciences, University of Veterinary and Animal Sciences, Lahore, Pakistan (K Hayat MS); Department of Pharmacy Administration and Clinical Pharmacy, Xian Jiaotong University, Xian, China (K Hayat MS); Center for Environmental and Respiratory Health Research, University of Oulu, Oulu, Finland (B Heibati PhD); Department of Nursing (R Heidari-Soureshjani MSc), Department of Clinical Biochemistry (A Mosapour PhD), Tarbiat Modares University, Tehran, Iran; School of Business, London South Bank University, London, UK (Prof C Herteliu PhD); Institute of Research and Development, Duy Tan University, Da Nang, Viet Nam (M Hosseinzadeh PhD); Faculty of Medicine of Tunis, University Tunis El Manar, Tunis, Tunisia (Prof M Hsairi MPH); Department of Epidemiology and Health Statistics, Central South University, Changsha, China (Prof G Hu PhD); Department of Community Medicine (O S Ilesanmi PhD), Department of Medicine (Prof M O Owolabi DrM), University College Hospital, Ibadan, Ibadan, Nigeria; Faculty of Medicine (I M Ilic PhD, Prof M M Santric-Milicevic PhD), School of Public Health and Health Management (Prof M M Santric-Milicevic), University of Belgrade, Belgrade, Serbia; Department of Epidemiology (Prof M D Ilic PhD), University of Kragujevac, Kragujevac, Serbia Prof M D Ilic PhD); Independent Consultant, Tabriz, Iran (S N Irvani MD); Institute for Physical Activity and Nutrition, Deakin University, Burwood, VIC, Australia (S Islam PhD); Sydney Medical School (S Islam PhD), School of Public Health (L D Knibbs PhD), School of Veterinary Science (B B Singh PhD), University of Sydney, Sydney, NSW, Australia; School of Health Systems and Public Health, University of Pretoria, Pretoria, South Africa (C C D Iwu MPH); Institute of Advanced Manufacturing Technologies, Peter the Great St Petersburg Polytechnic University, St Petersburg, Russia (Prof M Jakovljevic PhD); Institute of Comparative Economic Studies, Hosei University, Tokyo, Japan (Prof M Jakovljevic); Department of Community Medicine, Dr Baba Saheb Ambedkar Medical College & Hospital, Delhi, India (R P Jha MSc); Department of Community Medicine, Banaras Hindu University, Varanasi, India (R P Jha); Vanke School of Public Health, Tsinghua University, Beijing, China (J S Ji DSc); Institute of Molecular and Clinical Ophthalmology Basel, Basel, Switzerland (Prof J B Jonas MD); Department of Ophthalmology, Heidelberg University, Mannheim, Germany (Prof J B Jonas MD); School of Public Health, University College Cork, Cork, Ireland (Z Kabir PhD); Department of Forensic Medicine and Toxicology, All India Institute of Medical Sciences, Jodhpur, India (T Kanchan MD); Department of Epidemiology, Biostatistics and Clinical Research (Prof U Kapil MD), Department of Radiation Oncology (Prof G K Rath MD), Department of Psychiatry (Prof R Sagar MD), All India Institute of Medical Sciences, New Delhi, India; International Research Center of Excellence, Institute of Human Virology Nigeria, Abuja, Nigeria (G A Kayode PhD); Julius Centre for Health Sciences and Primary Care (G A Kayode PhD), Institute for Risk Assessment Sciences (E Traini MSc), Utrecht University, Utrecht, Netherlands; Open, Distance and eLearning Campus (Prof P N Keiyoro PhD), Department of Psychiatry (M Kumar PhD), University of Nairobi, Nairobi, Kenya; Department of Public Health, Jordan University of Science and Technology, Irbid, Jordan (Prof Y S Khader PhD); School of Food and Agricultural Sciences, University of Management and Technology, Lahore, Pakistan (N Khalid PhD); Department of Epidemiology and Biostatistics, Health Services Academy, Islamabad, Pakistan (E A Khan MPH); Department of Population Science, Jatiya Kabi Kazi Nazrul Islam University, Mymensingh, Bangladesh (M Khan PhD); Faculty of Health and Wellbeing, Sheffield Hallam University, Sheffield, UK (K Khatab PhD); College of Arts and Sciences, Ohio University, Zanesville, OH, USA (K Khatab); Department of Medical Parasitology (M M Khater MD), Public Health and Community Medicine Department (M R Salem MD), Cairo University, Cairo, Egypt; The Iranian Academy of Medical Sciences, Tehran, Iran (M Khayamzadeh MD); Department of Public Health, New Mexico State University, Las Cruces, NM, USA (Prof J Khubchandani PhD); Institute of Health Services Research (G Kim PhD, Prof E Park PhD), Department of Preventive Medicine (Prof E Park PhD), College of Medicine (Prof J Shin MD), Yonsei University, Seoul, South Korea; School of Traditional Chinese Medicine, Xiamen University Malaysia, Sepang, Malaysia (Y Kim PhD); Department of Nutrition, Simmons University, Boston, MA, USA (R W Kimokoti MD); School of Health Sciences, Kristiania University College, Oslo, Norway (Prof A Kisa PhD); Department of Global Community Health and Behavioral Sciences, Tulane University, New Orleans, LA, USA (Prof A Kisa); Department of Nursing and Health Promotion, Oslo Metropolitan University, Oslo, Norway (S Kisa PhD); Department of Internal and Pulmonary Medicine, Sheri Kashmir Institute of Medical Sciences, Srinagar, India (Prof P A Koul MD); Biomedical Research Networking Center for Mental Health Network, San Juan de Dios Sanitary Park, Sant Boi de Llobregat, Spain (A Koyanagi MD); Catalan Institution for Research and Advanced Studies, Barcelona, Spain (A Koyanagi MD); Department of Anthropology, Panjab University, Chandigarh, India (Prof K Krishan PhD); Faculty of Public Health, University of Indonesia, Depok, Indonesia (D Kusuma DSc); National Institute for Health Research Oxford Biomedical Research Centre, Oxford, UK (B Lacey PhD); Department of Community and Family Medicine, University of Baghdad, Baghdad, Iraq (F H Lami PhD); Division of Cancer Epidemiology and Genetics, National Cancer Institute, Rockville, MD, USA (Q Lan PhD); Department of Otorhinolaryngology, Father Muller Medical College, Mangalore, India (S Lasrado MS); International Society Doctors for the Environment, Arezzo, Italy (P Lauriola MD); Oxford University Clinical Research Unit, Wellcome Trust Asia Programme, Hanoi, Viet Nam (S Lewycka PhD); Center for Integration of Data and Health Knowledge (D B Machado PhD), Oswald Cruz Foundation (FIOCRUZ), Salvador, Brazil; Centre for Global Mental Health, London School of Hygiene & Tropical Medicine, London, UK (D B Machado); Grants, Innovation and Product Development Unit, South African Medical Research Council, Cape Town, South Africa (P W Mahasha PhD); Department of Public Health, Urmia University of Medical Science, Urmia, Iran (M Maheri PhD); Environmental Health Research Center, Kurdistan University of Medical Sciences, Sanandaj, Iran (Prof A Maleki PhD); Non-communicable Disease Research Center (Prof R Malekzadeh MD, S G Sepanlou MD), Health Policy Research Center (S Shahabi PhD), Shiraz University of Medical Sciences, Shiraz, Iran; Indonesian Public Health Association, Surabaya, Indonesia (S Martini PhD); Campus Caucaia, Federal Institute of Education, Science and Technology of Ceará, Caucaia, Brazil (F R Martins-Melo PhD); ICF International, Demographic and Health Surveys Program, Rockville, MD, USA (B K Mayala PhD); Department of Neurology, Janakpuri Super Specialty Hospital Society, New Delhi, India (Prof M Mehndiratta MD); Department of Neurology, Govind Ballabh Institute of Medical Education and Research, New Delhi, India (Prof M Mehndiratta); Peru Country Office, United Nations Population Fund, Lima, Peru (W Mendoza MD); Department of Reproductive Health and Population Studies, Bahir Dar University, Bahir Dar, Ethiopia (E W Mengesha MPH); Breast Surgery Unit, Helsinki University Hospital, Helsinki, Finland (T J Meretoja MD); University of Helsinki, Helsinki, Finland (T J Meretoja); University Centre Varazdin, University North, Varazdin, Croatia (T Mestrovic PhD); Woman-Mother- Child Department, Lausanne University Hospital, Lausanne, Switzerland (I Michalek PhD); Internal Medicine Programme, Kyrgyz State Medical Academy, Bishkek, Kyrgyzstan (Prof E M Mirrakhimov PhD); Department of Atherosclerosis and Coronary Heart Disease, National Center of Cardiology and Internal Disease, Bishkek, Kyrgyzstan (Prof E M Mirrakhimov); Institute of Addiction Research, Frankfurt University of Applied Sciences, Frankfurt, Germany (B Moazen MSc); Department of Epidemiology and Biostatistics, Shahrekord University of Medical Sciences, Shahrekord, Iran (A Mohammadian-Hafshejani PhD); Health Systems and Policy Research Unit (S Mohammed PhD), Department of Community Medicine (M B Sufiyan MD), Ahmadu Bello University, Zaria, Nigeria; Department of Health Care Management, Technical University of Berlin, Berlin, Germany (S Mohammed PhD); Clinical Epidemiology and Public Health Research Unit, Burlo Garofolo Institute for Maternal and Child Health, Trieste, Italy (L Monasta DSc, L Ronfani PhD, E Traini MSc); Computer, Electrical, and Mathematical Sciences and Engineering Division, King Abdullah University of Science and Technology, Thuwal, Saudi Arabia (P Moraga PhD); International Laboratory for Air Quality and Health, Queensland University of Technology, Brisbane, QLD, Australia (Prof L Morawska PhD); Department of Clinical Biochemistry (A Mosapour PhD), Social Determinants of Health Research Center (S Mouodi PhD), Babol University of Medical Sciences, Babol, Iran; Department of Fruit and Vegetable Product Technology, Prof Wacław Dąbrowski Institute of Agricultural and Food Biotechnology State Research Institute, Warsaw, Poland (Prof A Mousavi Khaneghah PhD); Department of Endocrinology & Metabolism, Institute of Post-Graduate Medical Education and Research and Seth Sukhlal Karnani Memorial Hospital, Kolkata, India (Prof S Mukhopadhyay MD); Scientific Communications Department, Invitae, Boulder, CO, USA (S B Munro PhD); Research and Analytics Department, Initiative for Financing Health and Human Development, Chennai, India (A J Nagarajan MTech); Department of Research and Analytics, Bioinsilico Technologies, Chennai, India (A J Nagarajan); Department of Pulmonary Medicine, Government Medical College Trivandrum, Trivandrum, India (S Nair MD); Health Action by People, Trivandrum, India (S Nair); Suraj Eye Institute, Nagpur, India (V Nangia MD); Department of Pediatrics, Arak University of Medical

Sciences, Arak, Iran (J Nazari MD); Department of General Surgery (I Negoi PhD), Emergency Hospital of Bucharest, Bucharest, Romania; College of Medicine and Health Sciences, Bahir Dar University, Gondar, Ethiopia (H B Netsere MS); Department of Biological Sciences, University of Embu, Embu, Kenya (J W Ngunjiri DrPH); Institute for Global Health Innovations, Duy Tan University, Hanoi, Viet Nam (H L T Nguyen MPH); Administrative and Economic Sciences Department (Prof B Oancea PhD), University of Bucharest, Bucharest, Romania; Translational Health Research Institute, Western Sydney University, Sydney, NSW, Australia (F A Ogbo PhD); Department of Preventive Medicine, Kyung Hee University, Dongdaemun-gu, South Korea (I Oh PhD); Department of Psychiatry and Behavioural Neurosciences (A T Olagunju MD), Population Health Research Institute (T Sathish PhD), McMaster University, Hamilton, ON, Canada; Department of Psychiatry, University of Lagos, Lagos, Nigeria (A T Olagunju MD); Centre for Healthy Start Initiative, Lagos, Nigeria (B O Olusanya PhD, J O Olusanya MBA); Department of Pharmacology and Therapeutics, University of Nigeria Nsukka, Enugu, Nigeria (Prof O E Onwujekwe PhD); Laboratory of Public Health Indicators Analysis and Health Digitalization, Moscow Institute of Physics and Technology, Dolgoprudny, Russia (N Otstavnov BA, S S Otstavnov PhD); Department of Project Management, National Research University Higher School of Economics, Moscow, Russia (S S Otstavnov PhD); Department of Respiratory Medicine, Jagadguru Sri Shivarathreeswara Academy of Health Education and Research, Mysore, India (Prof M P A DNB); Department of Medical Humanities and Social Medicine, Kosin University, Busan, South Korea (Prof E Park PhD); Department of Poverty, Gender and Youth, Population Council, New Delhi, India (S K Patel PhD); Center of Excellence in Behavioral Medicine, Nguyen Tat Thanh University, Ho Chi Minh City, Viet Nam (H Q Pham MD, G T Vu BA); Department of Cardiology, University of Bern, Bern, Switzerland (T Pilgrim MD); HIV and Mental Health Department, Integrated Development Foundation Nepal, Kathmandu, Nepal (K N Pokhrel PhD); University Medical Center Groningen (Prof M J Postma PhD), School of Economics and Business (Prof M J Postma PhD), University of Groningen, Groningen, Netherlands; School of Engineering, Macquarie University, Sydney, NSW, Australia (N Rabiee PhD); Pohang University of Science and Technology, Pohang, South Korea (N Rabiee); College of Medicine, University of Central Florida, Orlando, FL, USA (A Radfar MD); Department of Community Medicine, Maharishi Markandeshwar Medical College & Hospital, Solan, India (M Rahman PhD); School of Nursing and Healthcare Professions, Federation University Australia, Berwick, VIC, Australia (M Rahman); Future Technology Research Center, National Yunlin University of Science and Technology, Yunlin, Taiwan (A Rahmani PhD); Research Department, Policy Research Institute, Kathmandu, Nepal (C L Ranabhat PhD); Health and Public Policy Department, Global Center for Research and Development, Kathmandu, Nepal (C L Ranabhat PhD); Department of Oral Pathology, Sharavathi Dental College and Hospital, Shimogga, India (S Rao MDS); Department of Forensic Medicine and Toxicology (Prof P Rastogi MD), Kasturba Medical College (Prof B Unnikrishnan MD), Manipal Academy of Higher Education, Mangalore, India; University College London Hospitals, London, UK (D L Rawaf MD); Academic Public Health England, Public Health England, London, UK (Prof S Rawaf MD); School of Health, Medical and Applied Sciences, CQ University, Sydney, NSW, Australia (L Rawal PhD); School of Medicine and Translational Health Research Institute, Western Sydney University, Campbelltown, NSW, Australia (Prof A M N Renzaho PhD); Department of Health Information Management (B Reshmi PhD), Department of Community Medicine (R S Shetty MD), Manipal Academy of Higher Education, Manipal, India; Network of Immunity in Infection, Malignancy and Autoimmunity, Universal Scientific Education and Research Network, Tehran, Iran (Prof N Rezaei PhD); Department of Surgery, University of Minnesota, Minneapolis, MN, USA (J Rickard MD); Department of Surgery, University Teaching Hospital of Kigali, Kigali, Rwanda (J Rickard); Department of Clinical Research, Federal University of Uberlândia, Uberlândia, Brazil (L Roever PhD); School of Medicine, Gonabad University of Medical Sciences, Gonabad, Iran (M Rostamian PhD); African Genome Center, Mohammed VI Polytechnic University (UM6P), Ben Guerir, Morocco (E Rubagotti PhD); Center for Research in Congenital Anomalies and Rare Diseases, ICESI University (Centro de Investigaciones en Anomalías Congénitas y Enfermedades Raras, Universidad Icesi), Cali, Colombia (E Rubagotti); Department of Internal Medicine, University of Botswana, Gaborone, Botswana (G M Rwegerera MD); Sharjah Institute for Medical Research (B Saddik PhD), University of Sharjah, Sharjah, United Arab Emirates; Applied Biomedical Research Center and Biotechnology Research Center, Mashhad University of Medical Sciences, Mashhad, Iran (A Sahebkar PhD); Department of Public Health, Madda Walabu University, Bale Robe, Ethiopia (B Sahiledengle MPH); Department of Entomology (A M Samy PhD), Department of Neurology (Prof A S Shalash PhD), Ain Shams University, Cairo, Egypt; Independent Consultant, Thiruvananthapuram, India (S Y Saraswathy PhD); Geriatric and Long Term Care Department, Hamad Medical Corporation, Doha, Qatar (B Sathian PhD); Faculty of Health & Social Sciences, Bournemouth University, Bournemouth, UK (B Sathian PhD); Department of Psychology (D C Schwebel PhD), School of Medicine (Prof J A Singh MD), University of Alabama at Birmingham, Birmingham, AL, USA; Public Health Division, An-Najah National University, Nablus, Palestine (A A Shaheen PhD); Department of Internal Medicine, Ziauddin University, Karachi, Pakistan (I Shahid MBBS); Independent Consultant, Karachi, Pakistan (M A Shaikh MD); School of Medicine, Alborz University of Medical Sciences, Karaj, Iran (M Shams-Beyranvand MSc); Symbiosis Medical College for Women, Symbiosis International University, Pune, India (M Shannawaz PhD); Centre for Medical Informatics, University of Edinburgh, Edinburgh, UK (Prof A Sheikh MD); National Institute of Infectious Diseases, Tokyo, Japan (M Shigematsu PhD); Department of Public Health Dentistry, Krishna Institute of Medical Sciences Deemed to be University, Karad, India (Prof K M Shivakumar PhD); School of Health, University of Technology Sydney, Sydney, NSW, Australia (S Siabani PhD); Department of Medicine, Dow University of Health Sciences, Karachi, Pakistan (T J Siddiqi MB); School of Public Health & Zoonoses, Guru Angad Dev Veterinary & Animal Sciences University, Ludhiana, India (B B Singh PhD); Medicine Service, US Department of Veterans Affairs, Birmingham, AL, USA (Prof J A Singh MD); Department of Midwifery, Dire Dawa University, Dire Dawa, Ethiopia (Y Sintayehu MSc); Department of Public Health, Arba Minch University, Arba Minch, Ethiopia (M B Sorrie MPH); Hull York Medical School, University of Hull, Hull, UK (I N Soyiri PhD); Division of Community Medicine, International Medical University, Kuala Lumpur, Malaysia (C T Sreeramareddy MD); Occupational and Environmental Medicine Department, University of Gothenburg, Gothenburg, Sweden (L Stockfelt PhD); National Institute of Epidemiology, Indian Council of Medical Research, Chennai, India (R Suliankatchi Abdulkader MD) Department of Medicine, University of Valencia, Valencia, Spain (Prof R Tabarés-Seisdedos PhD); Carlos III Health Institute, Biomedical Research Networking Center for Mental Health Network, Madrid, Spain (Prof R Tabarés-Seisdedos PhD); Cancer Control Center, Osaka International Cancer Institute, Osaka, Japan (T Tabuchi MD); Research Center for Molecular Medicine, Hamadan University of Medical Sciences, Hamadan, Iran (A Taherkhani PhD); Department of Public Health and Community Medicine, Central University of Kerala, Kasaragod, India (Prof K R Thankappan MD); Modestum, London, UK (M R Tovani-Palone PhD); Amity Institute of Biotechnology, Amity University Rajasthan, Jaipur, India (E Upadhyay PhD); Clinical Cancer Research Center, Milad General Hospital, Tehran, Iran (S Valadan Tahbaz PhD, S Yahyazadeh Jabbari MD); Department of Microbiology, Islamic Azad University, Tehran, Tehran, Iran (S Valadan Tahbaz PhD); Department of Nephrology, Christian Medical College and Hospital, Vellore, India (Prof S Varughese FRCP); Department of Medical and Surgical Sciences, University of Bologna, Bologna, Italy (Prof F S Violante MD); Occupational Health Unit, Sant'Orsola Malpighi Hospital, Bologna, Italy (Prof F S Violante); Faculty of Information Technology, HUTECH University, Ho Chi Minh City, Viet Nam (B Vo PhD); Foundation University Medical College, Foundation University Islamabad, Islamabad, Pakistan (Prof Y Waheed PhD); Demographic Change and Aging Research Area, Federal Institute for Population Research, Wiesbaden, Germany (A Werdecker PhD); Health Services Management Research Center, Kerman University of Medical Sciences, Kerman, Iran (V Yazdi-Feyzabadi PhD); Department of Health Management, Policy, and Economics, Kerman University of Medical Sciences, Kerman, Iran (V Yazdi-Feyzabadi); Department of Public Health, Wollega University, Nekemte, Ethiopia (M T Yilma MPH); Department of Neuropsychopharmacology, National Center of Neurology and Psychiatry, Kodaira, Japan (N Yonemoto PhD); Department of Public Health, Juntendo University, Tokyo, Japan (N Yonemoto PhD); Department of Health Policy and Management, Jackson State University, Jackson, MS, USA (Prof M Z Younis PhD); School of Business & Economics, University Putra Maylisia, Kuala Lumpur, Malaysia (Prof M Z Younis PhD); Department of Epidemiology and Biostatistics (Prof C Yu PhD), School of Medicine (Z Zhang PhD), Wuhan University, Wuhan, China; School of Public Health and Management, Hubei University of Medicine, Shiyan, China (Y Yu MS); Maternal and Child Health Division, International Centre for Diarrhoeal Disease Research, Bangladesh, Dhaka, Bangladesh (S Zaman MPH); School of Public Health, Hubei Province Key Laboratory of Occupational Hazard Identification and Control, Wuhan University of Science and Technology, Wuhan, China (Y Zhang PhD); School of Population and Public Health, University of British Columbia, Vancouver, BC, Canada (Prof M Brauer DSc).
